# Kinase domain-targeted isolation of defense-related receptor-like kinases (RLK/Pelle) in *Platanus* × *acerifolia*: phylogenetic and structural analysis

**DOI:** 10.1186/1756-0500-7-884

**Published:** 2014-12-08

**Authors:** Massimo Pilotti, Angela Brunetti, Paolo Uva, Valentina Lumia, Lorenza Tizzani, Fabio Gervasi, Michele Iacono, Massimo Pindo

**Affiliations:** Plant Pathology Research Center, CRA-PAV Agricultural Research Council, V. C.G. Bertero 22, 00156 Rome, Italy; CRS4 Bioinformatics Laboratory POLARIS Science and Technology Park, 09010 Pula, Cagliari, Italy; Fruit Tree Research Center, CRA-FRU Agricultural Research Council, V. Fioranello, 52, 00134 Rome, Italy; Roche Diagnostics SpA, V. G.B. Stucchi 110, 20052 Monza Milano, Italy; Research and Innovation Centre, Edmund Mach Foundation, V. E. Mach 1, 38010 San Michele a/A, Trento, Italy

**Keywords:** *Platanus* × *acerifolia*, RLK/Pelle, Pto-like, CrRLK1L, LRR XII, WAK-like, LRR X-BRI1, Relaxed purifying selection, Pathogen resistance

## Abstract

**Background:**

Plant receptor-like kinase (RLK/Pelle) family regulates growth and developmental processes and interaction with pathogens and symbionts.

Platanaceae is one of the earliest branches of Eudicots temporally located before the split which gave rise to Rosids and Asterids. Thus investigations into the RLK family in *Platanus* can provide information on the evolution of this gene family in the land plants.

Moreover RLKs are good candidates for finding genes that are able to confer resistance to *Platanus* pathogens.

**Results:**

Degenerate oligonucleotide primers targeting the kinase domain of stress-related RLKs were used to isolate for the first time 111 RLK gene fragments in *Platanus* × *acerifolia*. Sequences were classified as candidates of the following subfamilies: CrRLK1L, LRR XII, WAK-like, and LRR X-BRI1 group. All the structural features typical of the RLK kinase domain were identified, including the non-RD motif which marks potential pathogen recognition receptors (PRRs). The LRR XII candidates, whose counterpart in *Arabidopsis* and rice comprises non-RD PRRs, were mostly non-RD kinases, suggesting a group of PRRs. Region-specific signatures of a relaxed purifying selection in the LRR XII candidates were also found, which is novel for plant RLK kinase domain and further supports the role of LRR XII candidates as PRRs. As we obtained CrRLK1L candidates using primers designed on *Pto* of tomato, we analysed the phylogenetic relationship between CrRLK1L and Pto-like of plant species. We thus classified all non-solanaceous *Pto*-like genes as CrRLK1L and highlighted for the first time the close phylogenetic vicinity between CrRLK1L and Pto group. The origins of *Pto* from CrRLK1L is proposed as an evolutionary mechanism.

**Conclusions:**

The signatures of relaxed purifying selection highlight that a group of RLKs might have been involved in the expression of phenotypic plasticity and is thus a good candidate for investigations into pathogen resistance.

Search of *Pto*-like genes in *Platanus* highlighted the close relationship between CrRLK1L and *Pto* group. It will be exciting to verify if *sensu strictu Pto* are present in taxonomic groups other than Solanaceae, in order to further clarify the evolutionary link with CrRLK1L.

We obtained a first valuable resource useful for an in-depth study on stress perception systems.

**Electronic supplementary material:**

The online version of this article (doi:10.1186/1756-0500-7-884) contains supplementary material, which is available to authorized users.

## Background

In animals, cell surface receptor tyrosine kinases (RTKs) and serine/threonine kinase receptors (STRKs) are of primary importance for the perception of intercellular signals and the transduction of signals at an intracellular level. In general the domain composition of these receptors is defined by an extracellular ligand-binding domain, a single-pass trans-membrane domain and a cytoplasmic tyrosine or serine/threonine kinase domain [1, 2]. In the plant lineage, the counterpart of RTKs and STRKs is represented by the family of receptor-like kinases (RLKs), which share the same domain composition [3]. The RLK kinase domain, a serine-threonine kinase, belongs to the same gene family as the *Drosophila melanogaster* Pelle and mammalian interleukin receptor-associated kinases. Thus the term RLK/Pelle was established [3, 4].

With regard to RLK/Pelles, a striking difference between the land plant and non-plant eukaryote genomes is the size of this family, in fact 329 genes have been identified in moss (*Physcomitrella patens*), 610 in *Arabidopsis*, 647 in tomato, 1192 in poplar (*Populus trichocarpa*), 1070 in rice, 1 and 4 in the animals *Drosophila melanogaster* and *Homo sapiens* respectively, and none have been identified in fungal organisms [5, 6]. It has been suggested that one of the evolutionary reasons for the gene family’s expansion derives from the need to cope, as sessile organisms, with an ever-changing environment, including a multitude of mutating pathogenic and *would be*-pathogenic microbes [4, 5, 7]. This would account for the variety of RLK/Pelle types - 53 subfamilies categorized in *Arabidopsis* - for the genetic redundancy present in part of the subfamilies, and for the up-regulation of hundreds of RLK/Pelle members under biotic stress [3–5, 7].

Some members within the plant RLK/Pelle family, have been shown to be transmembrane receptors [8, 9] and it is likely that this is the main functional contribution of these proteins [4]. Two functional groups are recognized in plants: RLKs controlling growth and development processes, and RLKs regulating interactions with microbes, both pathogens and symbionts. The rice (*Oryza sativa*) Xa21 is one example of the latter class, which confers resistance to the bacterium *Xanthomonas oryzae* pv. *oryzae*[10]. Other examples include EFR (EF-TU RECEPTOR) of *Arabidopsis thaliana* which is a PAMP (Pathogen Associated Molecular Pattern) receptor [11], and *Arabidopsis* RFO1 (RESISTANCE TO FUSARIUM OXISPORUM 1) which confers broad spectrum resistance to diverse *Fusarium oxisporum* races and *Verticillium longisporum*[12, 13].

A functional dualism may involve BRI1 (BRASSINOSTEROID-INSENSITIVE1). This RLK/Pelle has an important role in the regulation of growth and development through the perception of brassinosteroids (BRs) [14, 15]. Its involvement in stress responses is suggested by the fact that BRs are also strongly related in stress signaling [16]. Furthermore, a tomato BRI1 ortholog, SR160 (SYSTEMIN RECEPTOR 160), is able to bind systemin, a solanaceous specific protein which induces systemic wound responses following attack by feeding insects [17] although it does not represent the functional systemin receptor [18, 19].

A lack of extracellular domain is a characteristic of another class of the plant RLK/Pelle family, the receptor-like cytoplasmic kinases (RLCKs) [4]. Pto (for *Pseudomonas syringae* pv. *tomato*) is a well-known member of this class and confers resistance to the bacterial pathogen, *P. syringae* pv *tomato* in wild tomato species, according to a host genotype/pathogen race specificity fashion [20]. Great interest in *Pto* orthologs in plant species has arisen from the fact that the overexpression of *Pto* has been shown to confer a broad spectrum resistance in tomato [21].

Platanaceae is a tree family that is considered as one of the earliest branches of Eudicots [22] (http://www.mobot.org/MOBOT/research/APweb/.). In contrast, *Platanus* × *acerifolia* (Ait.) Willd. is a species of this family which originated in the 17th century from a cross between individuals belonging to the *P. occidentalis* L. species group and *P. orientalis* L. [23, 24]. Therefore, *P*. × *acerifolia* (*Pac*) combines genomic contributions from the two parental species, which evolved separately for long geological periods [24]. The high haploid chromosome number, n =21, suggests that the current Platanaceae had ancient polyploid origins [25] which adds to the overall complexity. Given these features, investigations into the RLK/Pelle family in *Pac* can provide information on the nature of this gene family in the Dicots, especially when compared with the *Arabidopsis* counterpart.

Platanaceae suffer from several diseases including canker stain, a vascular disease caused by the fungus *Ceratocystis platani* (J.M. Walter) Engelbr. & T. C. Harr., and antrachnose, caused by the fungus *Apiognomonia veneta* (Sacc. et Speg.), which are particularly destructive and have heavily conditioned the diffusion and cultivation of *Platanus* spp. [26, 27]. Thus unravelling the resistance response of *Platanus* to pathogens is necessary in order to sustain and speed up current time-consuming genetic selection programmes [24, 28]. The plant RLK/Pelle family is thus a good candidate for finding genes that are able to confer resistance to pathogens.

The aim of this study was to isolate RLK/Pelle genes in *Pac* which, based on knowledge of *Arabidopsis*, play a potential role in pathogen perception and signal transduction. We successfully applied a PCR-based strategy, which targeted a representative region of the kinase domain.

We isolated for the first time RLK/Pelle gene fragments in *Pac*, with an uninterrupted open reading frame (ORF). Sequence analyses clearly showed that the genes were, in most cases, candidates of the following RLK/Pelle subfamilies: CrRLK1L, LRR XII, WAK-like, LRR X-BRI1 group. A number of structural motifs and the analysis of non-synonymous versus synonymous substitution rates, confirmed the phylogenetic identification and provided an insight into their potential functions. In fact, the detection in *Pac* of signals of a completely relaxed purifying selection in sub-regions of the kinase domain, is another important finding of this study.

As we isolated CrRLK1L-L using primers designed on *Pto* of tomato, we also wished to clarify the relationships between Pto-like of several plant species available in the GenBank, the *bona fide* Pto family of solanaceous species and CrRLK1L. As a result a close phylogenetic relationship was revealed between Pto and CrRLK1L.

## Methods

### Plant material, nucleic acid extraction and primer design

Genomic DNA was isolated from young leaves collected in the spring from a potted *Pac* tree (accession MS.12) using a Plant Midi Kit (Qiagen, Hilden, Germany) according to the manufacturer’s instructions.

Degenerate primers were designed in order to isolate RLK/Pelle candidate genes for pathogen perception and signal transduction: 1) known R genes (*Pto*, *Xa-21*, *Xa-26*, *RFO1*); 2) PAMP receptor genes (*EFR*); 3) the whole BRI1 gene group. The primers were designed within the kinase catalytic domain. They were generated from the nucleotide stretches conserved in the model gene and several plant homologs, available from the NCBI GenBank database. In the case of *RFO1*, a WAK-like gene, collected sequences were used to infer a phylogenetic tree in order to identify clades of homologous WAK-like genes from which distinct sets of primers were generated. We then used a similar strategy in order to search for *BRI1*-like genes.

All the information regarding primer is reported in Additional file 1.

### Amplification and cloning of gene fragments

PCRs were performed in a 50-μl reaction volume containing each dNTP at 200 μM, each primer at 1 μM, 2.5U of Platinum *Taq* DNA Polymerase High Fidelity (Invitrogen), 2 mM MgSO_4_, and 100–200 ng of genomic DNA as a template, in the buffer supplied by the manufacturer. The initial denaturation step was carried out at 94°C for 2 min, followed by 35 amplification cycles (94°C for 1 min, annealing at 47°C for 50 s, an extension at 68°C for 1 min) and a final extension step at 68°C for 15 min. Analysis of PCR products, cloning and sequencing was as previously described [29]. A variable number of clones *per* cloned DNA band were sequenced in both directions (Bio Fab Research) (Additional file 2). RLK/Pelle-like sequences were deposited in the GenBank database [GenBank: EU722764-EU722869, EU722871-EU722900, HQ425329-30] (Additional file 3).

### Alignments and phylogenetic analyses

Clone sequences were trimmed of vector and primer sequences and were then considered for further analysis. To select gene fragments representing the kinase-catalytic region, the amino acid sequences were compared with UniProt using blastp [30]. Multiple sequence alignments were performed with MAFFT [31]. Pairwise local alignments were performed using the EMBOSS Pairwise Alignment suite [32] (gap open: 10; gap extend: 0.5; matrix: Blosum62).

Since we isolated portions of the full length ORF, we classified the *Pac* sequences according to the nomenclature by Shiu and Bleecker [4] or the name of the gene for which a potential orthology relation was evident, followed by the code -L (= − like).

Phylogenetic analysis was based on the MAFFT alignment of the amino acid sequences of the kinase-catalytic domain. Trees were inferred using the Neighbor-joining (NJ) method [33], with 1,000 bootstrap replications. The evolutionary distances were computed using the Poisson correction method with rate uniformity among sites [34] and were in the units of the number of amino acid substitutions per site. Analyses were conducted in Mega version 6 [35].

Phylogenetic analyses were also conducted with two maximum-likelihood (ML) methods: i) Whelan And Goldman substitution model - WAG [36], with 1,000 bootstrap replications and rate uniformity among sites (analyses were conducted in Mega version 6). ii) ML approach implemented in the TREE-PUZZLE software package [37], with the following parameters: VT (variable time) substitution model [38], gamma-distributed rates of heterogeneity among sites, amino acid frequencies inferred from the data set, branch lengths estimated without the molecular-clock assumption, 50,000 puzzling steps to infer branch support values for the relative majority consensus tree.

To classify *Pac* sequences we made a phylogenetic comparison with representative members of all the subfamilies of the *Arabidopsis* RLK/Pelle complement [4]. Then sequences were divided into homology groups and each group was compared with the closest whole RLK/Pelle subfamily.

We also performed a comparative phylogenetic analysis in order to shed light on the relations between the *Pto* group [39], *Pto*-like of *Solanaceae*, *Pto*-like of other dicot and monocot plants, CrRLK1L, and the Pto-primer-derived sequences obtained in this work.

Apart from *Arabidopsis*, genes from nine additional full genome-sequenced species and partial sequences from a number of plant species were included in the phylogenies for comparison. All the reference sequences and those used to root the trees are reported in Additional file 4.

In the phylogenetic trees, sequence names contain: I) the acronym of the protein or, secondly, the locus tag or the accession number, II) the Latin name of the species, and III) the RLK/Pelle subfamily to which the protein belongs. Latin names were abbreviated as follows: Arab. thal. = *Arabidopsis thaliana*, Caps. = *Capsicum*, Cath. = *Catharantus*, Frag. = *Fragaria*, Mu. = *Musa*, Nicot. tab. = *Nicotiana tabacum*, Ory. = *Oryza*, Phas. = *Phaseolus*, Pop. = *Populus*, Pot. = *Potentilla*, Ric. = *Ricinus*, Sol.pimp. = *Solanum pimpinellifolium*, Sol.lyc. = *Solanum lycopersicum*, Vit. = *Vitis*, Physc.patens = *Physcomitrella patens*.

### Domain detection and determination of other structural features

Domain detection was performed using Pfam [40] and SMART [41].

The phylogenetic classification at the subfamily level was validated by comparing some representative *Pac* sequences with long isotigs generated from a 454 GS-FLX transcriptome data set of the same species, though using a different genotype (Pilotti, Brunetti, Iacono and Pindo, unpublished data) (Additional file 5). Putative extracellular domains of the isotigs were identified as described above.

Serine-threonine and tyrosine protein kinase motifs [42] were searched for and a consensus was determined by using the motif-based sequence analysis tool MEME [43].

We compared the activation segment of Pto and BRI1 with the corresponding region of the deduced protein of the entire *Pac* RLK/Pelle-like sequence complement and the corresponding RLK/Pelle subfamilies of *Arabidopsis*.

### Non-synonymous versus synonymous substitution rates

The ratio of non-synonymous (Ka) to synonymous (Ks) nucleotide substitutions was calculated for pairwise comparisons using a 75 bp sliding window with a step size of 15 bp. The analysis was based on codon-delimited nucleotide alignments and performed with JCoDA [44] which uses the PAML (yn00) suite [45] and the Yang and Nielsen substitution model [46]. JCoDA was also used to identify the individual sites under positive selection (site by site): the site models M7 and M8, which account for transitional rate bias and unequal codon frequencies, were applied, and the differences were assessed with a likelihood ratio test (d.f. = 2). The tree topology required by the two models was inferred from the amino acid sequence by the maximum likelihood approach using the Phylip package [47], with a JTT substitution matrix [48] and rate heterogeneity approximated using a discrete γ-distribution with four categories. The posterior probabilities of individual sites belonging to the class Ka/Ks >1 were calculated using the Bayes empirical Bayes approach implemented in PAML.

Codon-delimited alignments were also used to map those positions that were subject to non-synonymous variations in order to: i) identify those that were shared among the different phylogenetic LRR XII-L groups of *Pac* and LRR XII of *Arabidopsis*, ii) to describe the amino acid variability at each position.

## Results

### Amplification pattern and oligonucleotide efficiency

Generally PCRs based on degenerate oligonucleotide pairs always gave a clear amplification pattern with minimum or no smearing. Evident expected-sized DNA bands were always present and a variable number of clones were sequenced for each band. In some cases additional non expected bands of variable intensity were also present but these were not investigated further.

Nucleotide sequences of the cloned amplicons revealed that oligonucleotide pairs worked highly efficiently at priming the amplification of target genes. Amplicons from 16 oligonucleotide pairs-based PCRs, out of 23, yielded RLK/Pelle-like sequences with maximum efficiency (100%) (Additional file 2). A total of 111 out of 136 RLK/Pelle candidates showed an uninterrupted ORF. These sequences represent the focus of this paper. Their length is indicated in Table 1. A total of 26 RLK/Pelle candidates showed interrupted ORFs due to frameshift indels – probably pseudogenes or truncated genes - and were not analysed further.

**Table 1:** **RLK/Pelles obtained in** ***Pac***

Homologous RLK/Pelle subfamily	No	Subdomain spanning	Length (nt)	non-RD kinases	Atypical kinases
CrRLK1L	28	I → IX (27)	495–538	-	-
		II → IX (1)			
LRR-XII	34	I → VIII (33)	442–508	31	2
		I → VII/VIII (1)			
WAK-like	28	I → VIII/IX( 7)	503–780	1	1
		I → IX (17)			
		I → XI (4)			
LRR X-BRI1 group	7	I → IX/X (4)	628–876	-	-
		I → XI (3)			
Uncertain: RKF3	3	I → VIII/IX	549–582	-	-
L-Lectin	4	I → VIII	494	-	-
LRR-VII	2	I → VIII	460	2	2
SD-2	2	I → VIII/IX	490, 492	2	-
Uncertain:	3	I/II → VIII	448	3	-
Thaumatin/LRK10L-2/					
GDPD/CRPK1-like-2					

### Phylogenetic analyses for RLK/Pelle subfamily identification

By using reference sequences of full genome-sequenced species, we initially verified that highly/well supported clades did not change between phylogenies based on the partial kinase domain and those inferred with the complete kinase domain or the full ORF. A comparison with CrRLK1L and WAK-like phylogenies in other studies [49, 50] further confirmed the robustness of our methodology (data not shown).

A highly supported clustering showed that close homologs of CrRLK1L, LRR XII, WAK-like and LRR X-BRI1 group, had been isolated (Additional file 6A and B).

**Figure 1:**
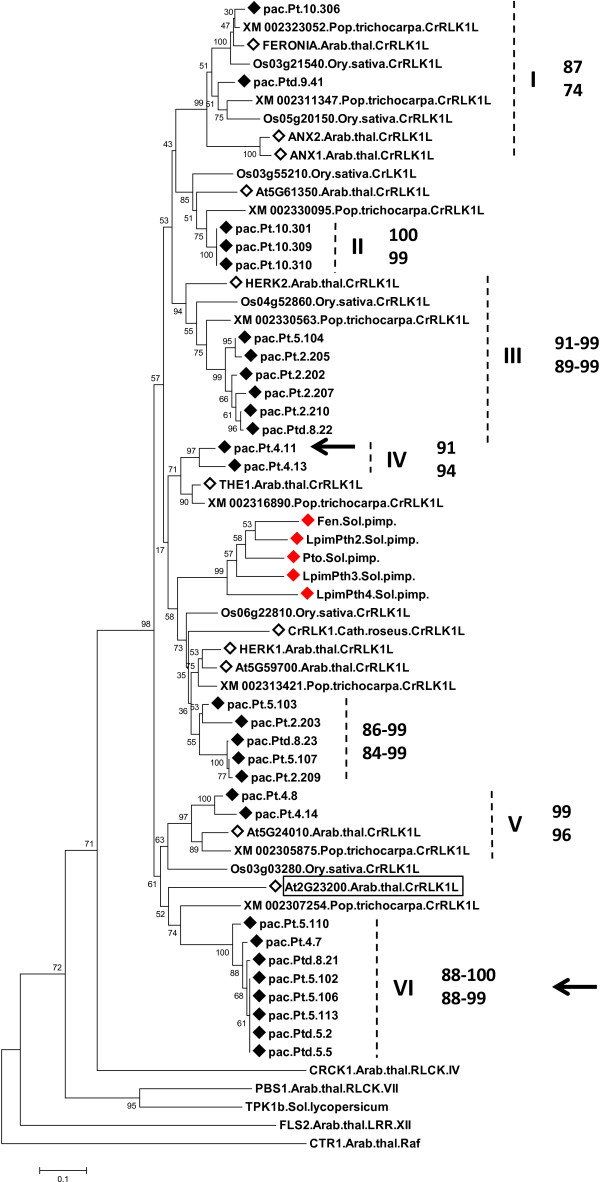
**Neighbor-joining tree of the aa sequences of** ***Pac*** **obtained with** ***Pto*** **primers.** *Pac* sequences: pac, ♦. As a comparison we included representative CrRLK1L sequences of *Arabidopsis* and *Catharanthus roseus* (*◊*), poplar and rice, Pto and paralogs of *Solanum piminellifolium* (red colored rhombus). Black arrows indicate *Pac* sequences sharing Thr204 of Pto, which is the main structural determinant of Pto for the specific recognition of AvrPto [59, 62]. The rectangular frame indicates the *Arabidopsis* CrRLK1L member that directly binds to the effector AvrPto of *Pseudomonas syringae* pv *tomato*[63]. The kinase domain region spanned subdomain I through IX. The percentage of replicate trees in which sequences clustered together in the bootstrap test (1000 replicates) are shown for each node. The tree is drawn to scale with branch length in the units of the number of amino acid substitution per site (scale at the bottom). Roman numerals indicate groups with a high bootstrap. Arabic numerals indicate amino acid (upper) and nucleotide (lower) identity scores in *Pac* sequences within each group. Abbreviations are explained in the text.

CrRLK1L candidates (CrRLK1L-L) were obtained with *Pto* primers. All clustered with a maximum bootstrap value with CrRLK1L of *Arabidopsis* and rice (Figure 1, Additional file 6 and Additional file 7) [50]. Specifically these candidates fell into the largest group of CrRLK1L, previously defined as CRPK1-like1 [4]. In the highly-supported CRPK1-like1 clade, Pto and its relatives of *L. pimpinellifolium* were also included, and clustered together with a maximum bootstrap value. The specific Pto clade did not include any CrRLK1L. The unknown sequences of poplar, which were used for comparison, were all included in the highly/well-supported subgroups headed by *Pac* sequences and the CrRLK1L reference sequences (Figure 1, Additional file 6 and Additional file 7). Using SMART and Pfam predictive tools, we identified the poplar sequences as CrRLK1L (see “Re-classification of plant *Pto*-like genes”).

**Figure 2:**
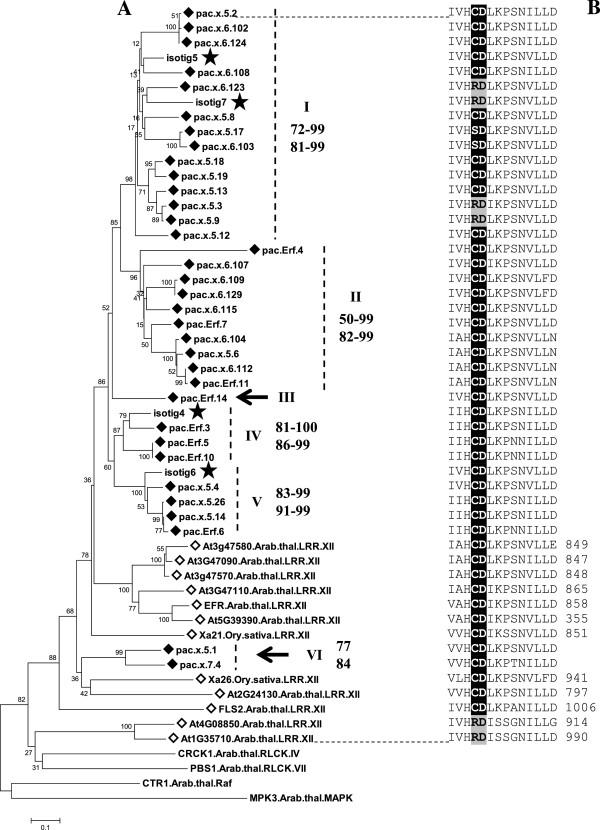
**LRR XII-L of** ***Pac*** **compared with** ***Arabidopsis*** **RLK/Pelle subfamily LRR XII. A** Neighbor-joining tree of the aa sequences of LRR XII-L of *Pac* (pac, ♦), the complete set of LRR XII of *Arabidopsis* (*◊*), two well-known members of LRR XII of rice – Xa21 and Xa26 (*◊*), and isotigs extracted from a 454 transcriptome dataset of *Pac* which have LRR signatures located upstream the kinase domain (black star) (Additional file 5). Analysis was based on the kinase domain region spanning subdomain I/II up to the beginning of VIII. See caption of Figure 1 for additional details on the representation of the tree. **B** Multiple alignment of the RD motif (shaded grey) and non-RD motif (shaded black) corresponding to the sequences included in the tree.

With regard to the sequences obtained with *Xa21*/*Xa26*/*EFR* primers, phylogenetic analyses clearly showed that they were LRR XII candidates (LRR XII-L) (Additional file 6, Figure 2). Founding members of LRR XII are *FLS2* and *EFR* in *Arabidopsis* and *Xa21* in rice [7, 11]. In general, homologs of the different species did not strictly group at low hierarchical levels (Figure 2). This characteristic of the LRR XII subfamily has been previously shown in genome wide analyses [7].

The use of *Xa21*/*Xa26*/*EFR* primers also yielded a few sequences which were homologous with other RLK/Pelle subfamilies (Additional file 6 and Additional file 8).

**Figure 3:**
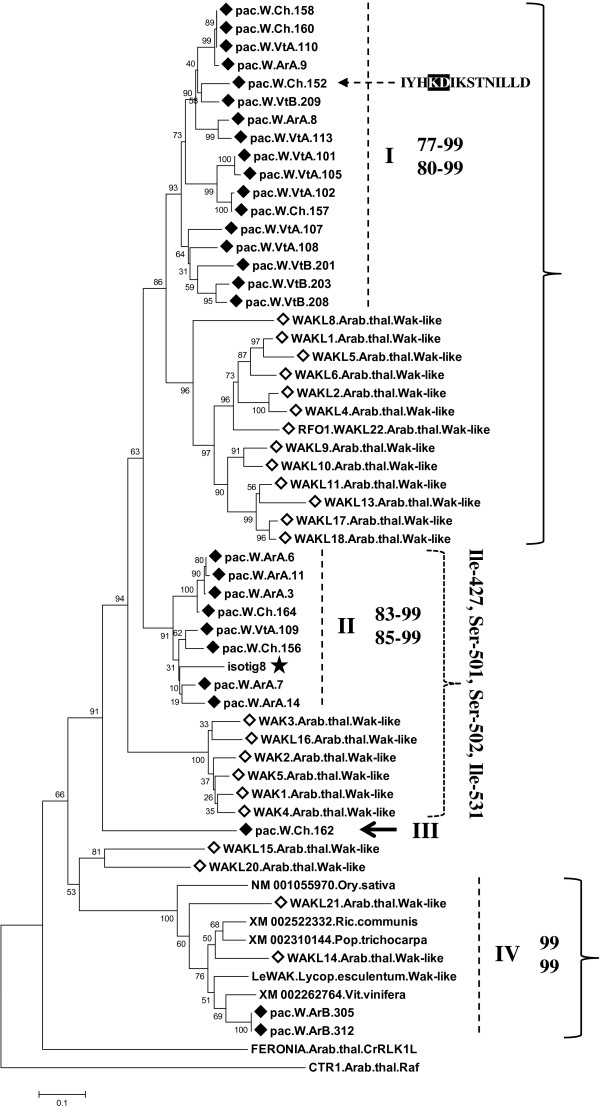
**Neighbor-joining tree of** ***Pac*** **WAK-like candidates and** ***Arabidopsis*** **RLK/Pelle subfamily Wak-like.** Analysis was based on the aa sequences of: *Pac* (pac ♦) obtained with Wak-like primers; the complete Wak-like subfamily of *Arabidopsis* (*◊*), other sequences from plant species and one isotig extracted from a 454 transcriptome dataset of *Pac*, which has the typical Wak-like extracellular domains (black star) (Additional file 5). The *Pac* sequence containing the non-RD motif is evidenced by a dashed arrow and the motif is shaded black. The kinase domain region spanned subdomain II up to the beginning of IX. The brackets indicate a likely genetic correspondence between *Pac* and *Arabidopsis* clades (uncertain in the case of the dotted bracket). Residues next to the brackets of clade II are shared by all the members of clade II and of WAK1-headed clade of *Arabidopsis* and differentiate these sequences from all the other *Arabidopsis* and *Pac* WAK-like (−L) (see Additional file 9). See caption of Figure 1 for additional details on the representation of the tree.

The phylogenetic analyses of sequences obtained with *WAK* primers clearly identified most of these sequences as WAK-like-L (Additional file 6, Figure 3). The topology of the tree depicted in Figure 3 suggested that the gene complement isolated in *Pac* was at least partially representative of the *Arabidopsis* subfamily structure. In fact, sequences of the two species clustered within four highly supported groups, of which three were species-specific. On the other hand, the group marked by WAKL14 and WAKL21 of *Arabidopsis* and LeWAK of tomato [51], included members from different species (included *Pac*) with a high statistical support (Figure 3). Multiple alignment of the kinase domain portion spanned by *Pac* sequences, enabled us to identify four conserved residues, which in the *Arabidopsis* WAK-like were exclusive to the WAK1 clade: Ile-427, Ser-501, Ser-502, Ile-531 (numbering refers to WAK1). Of the *Pac* sequences, only those of clade II shared all these residues (Additional file 9). Of the sequences obtained with *WAK* primers (thirty-one), three showed a homology with RFK3 (RECEPTOR-LIKE KINASE IN FLOWERS 3, subfamily RFK3-Like) of *Arabidopsis* and were not analysed further.

**Figure 4:**
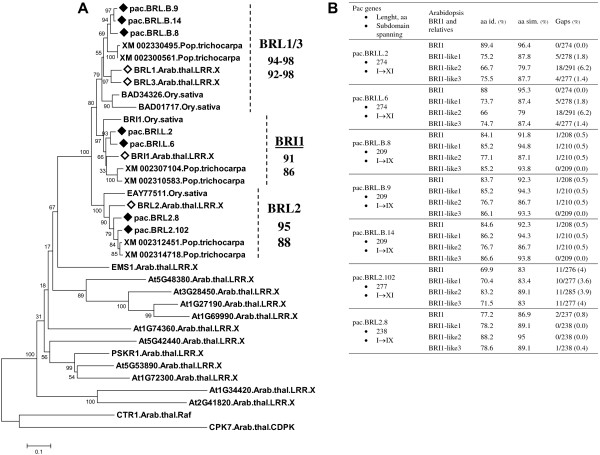
**LRR X-BRI1-L group of** ***Pac*** **compared with** ***Arabidopsis*** **RLK/Pelle subfamily LRR X. A**: Neighbor-joining tree of the aa sequences of: *Pac* (pac, ♦) which were obtained with BRI1 (−like) primers; the complete LRR X subfamily of *Arabidopsis*, which includes the BRI1 group (*◊*) [4] and other homologous sequences of rice and poplar. Analysis was based on the kinase domain region spanning subdomain I up to the beginning of X. See caption of Figure 1 for additional details on the representation of the tree. **B** Identity and similarity values and the number of gaps derived from the pairwise comparison of the *Pac* sequences with the *Arabidopsis* members of the BRI1 group.

Regarding the sequences obtained with *BRI1*(−like) primers, phylogenies clearly classified the *Pac* sequences as BRI1 group candidates (LRR X-BRI1-L) (Additional file 6, Figure 4). In fact sequences strictly clustered in the three highly/well supported sub-clades headed by BRI1, BRL1/BRL3 and BRL2 of *Arabidopsis*, together with poplar and rice orthologs. The percentage of sequence identity, similarity and the number of gaps of these sequences compared to *Arabidopsis* BRI1(−like) proteins agree with the phylogeny results (Figure 4).

All ML analyses - Mega 6 and TREE-PUZZLE implemented - confirmed results of the NJ analyses (Additional file 10).

To further confirm the reliability of the phylogenetic classification at the subfamily level, we compared selected sequences of *Pac*, for each homology group, with ten isotigs generated from a 454 transcriptome dataset derived from *P*. × *acerifolia*. Nearly all the isotigs spanned the whole region represented by PCR RLK/Pelle fragments and matched them with a high/maximum nucleotide identity score. In the isotigs all the putative extracellular domains typical of CrRLK1L, LRR XII, WAK-Like and LRR X were detected thus confirming the phylogenetic identification (Additional file 5).

### Re-classification of plant *Pto*-like genes

The apparent failure to collect potential orthologs of *Pto* gene family members, prompted us to investigate the existence of *sensu strictu Pto* orthologs (or close homologs) in plant species using NJ and ML phylogenetic analyses. We used two approaches.

**Figure 5:**
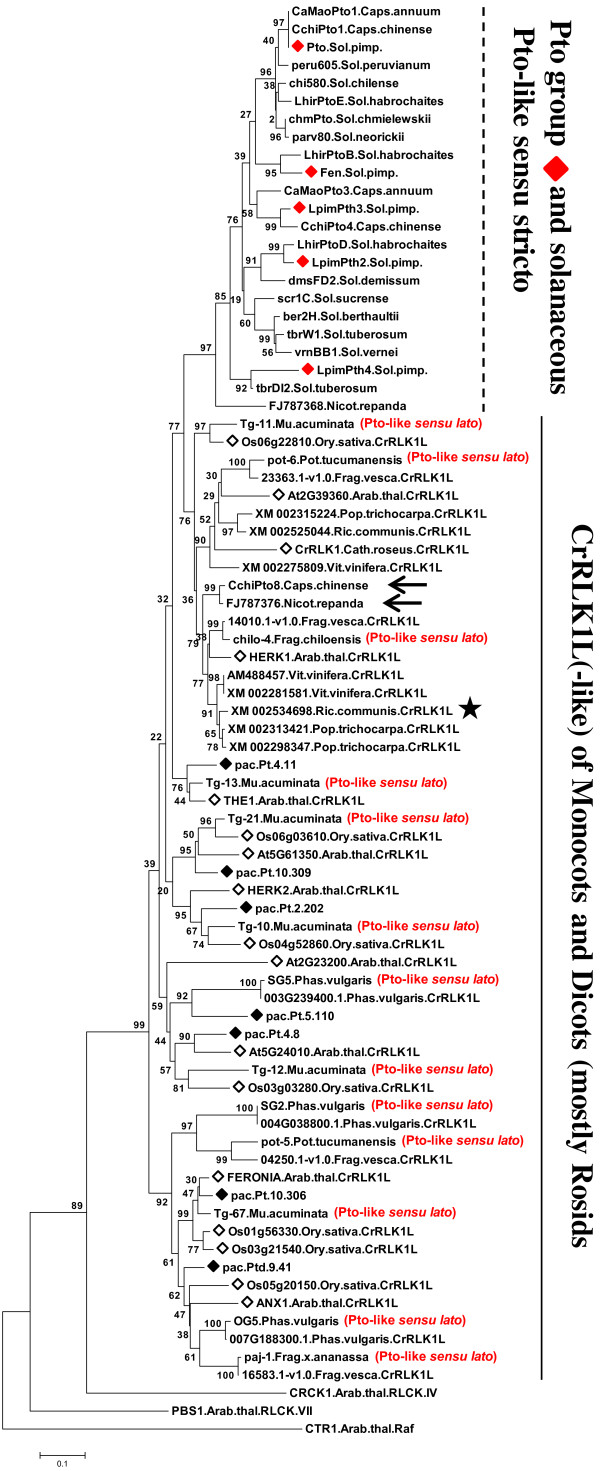
**Phylogenetic relationship between Pto(−like) (** ***sensu strictu*** **and** ***sensu latu*** **) and the RLK/Pelle subfamily CrRLK1L.** The Neighbor-joining analysis was based on the aa sequences of: Pto and paralogs of *Solanum pimpinellifolium* and the other solanaceous Pto-like (i.e. Pto-like *sensu strictu*); representative CrRLK1L of *Arabidopsis* (*◊*), rice (*◊*) and *Catharanthus roseus* (*◊*); Pto best blastp matches (namely CrRLK1L, based on domain composition) of *Arabidopsis*, rice, grapevine, poplar and castor bean; Pto-like fragments of non-solanaceous species (*Phaselus vulgaris*, cultivated and wild strawberry and *Musa acuminata*) (namely CrRLK1L-like, i.e. Pto-like *sensu latu*); best blastp matches from *F. vesca* and *P. vulgaris* (namely CrRLK1L) obtained using Pto-like fragments from the same species, as query. Representative CrRLK1L-like of *Pac* obtained with *Pto*-primer were also included (pac, ♦). Black arrows indicate the only solanaceous *so-called* Pto-like sequences, which clustered within a CrRLK1L-headed clade. The black star indicates a CrRLK1L of castor bean lacking the extracellular domain typical of the subfamily. The kinase domain region spanned subdomain I up to the beginning of IX. See caption of Figure 1 for additional details on the representation of the tree.

In the first approach we compared Pto locus and Pto-like of solanaceous species with Pto-like partial sequences of non-solanaceous species (including *Pac*) and the best Pto blastp matches of some fully sequenced non-solanaceous species. As *F. vesca* and *P.vulgaris* genomes have been also fully sequenced, we also incuded the best matches obtained from these genomes using Pto-like partial sequences of *P. vulgaris* and cultivated/wild strawberries as query. Representative CrRLK1L were included as control. All these sequences, clustered together with a high support (Figure 5; Additional file 6A). Within this clade, the branching pattern determined several highly/well supported groups among which one included the *Pto* locus and the vast majority of Pto-like sequences of Solanaceae. The other clades were headed by CrRLK1L sequences and contained all Pto-like partial sequences of non-solanaceous species, the best Pto and Pto-like blastp matches of fully sequenced non-solanaceous species and a few sequences of Solanaceae (Figure 5). For the sake of legibility, the phylogenetic tree in Figure 5 contains representative sequences of those listed in Additional file 4, which were all tested in a preliminary analysis with the same results. The best Pto blastp matches of *Arabidopsis* and rice corresponded to CrRLK1L members. Those of poplar, grapevine and castor bean and the best matches from *F. vesca* and *P. vulgaris*, obtained using non solanaceous Pto-like as query, were unknown genes with a C-terminal kinase domain, and 1 or 2 N-terminal malectin domains (Di-glucose binding within endoplasmic reticulum). Malectin is a membrane-anchored endoplasmic reticulum protein which is highly conserved in animals and recognizes glucose oligomers [52]. The same domain composition was detected in the *Arabidopsis* CrRLK1L, confirming the identity of poplar, grapevine, castor bean, bean and strawberry proteins as CrRLK1L. Results of NJ analysis were confirmed by the ML pylogenetic analyses - Mega6 and TREE-PUZZLE implemented (Additional file 11).

In the second approach we compared the complete CrRLK1L subfamily of those species in which this gene set had been characterised (i.e. arabidopsis, poplar, tomato, and rice) with *Pto* locus (see Additional file 4 for sequence sources). Moss (*Physcomitrella patens*) was also included in the analysis as a representative of basal lineages of land plant. CrRLK1L sequences of moss were retrieved from NCBI GenBank using CrRLK1L of *Arabidopsis* and Pto as query. The vast majority of the sequences grouped together in highly supported multi-species sub-clades confirming that in CrRLK1L, orthologs are more similar than paralogs. On the contrary Pto and paralogs and the other ortologs from tomato database formed a highly supported *Solanaceae*-specific clade. The phylogenetic analysis was performed with NJ and two ML methods, which gave the same results (Additional file 12).

Representative non-solanaceous Pto-like partial sequences were each compared with close CrRLK1L relatives and with Pto. Identities and similarity values were always clearly higher with the CrRLK1L relatives than with Pto. In line with this, the number of gaps was lower in CrRLK1L than with Pto. The same analysis, performed on representative *Pac* CrRLK1L-L, gave similar results (Table 2).

**Table 2:** **Comparison of non-solanaceous Pto-like partial sequences with Pto and close relatives of CrRLK1L**

Pto-like genes	Close phylogenetic relative Pto	aa id.(%)	aa sim.(%)	Gaps (%)
chilo-4 *Fragaria chiloensis*	At.HERK1 CrRLK1L	91.3	95.6	0/183 (0.0)
	Pto	70.8	81.1	5/185 (2.7)
pot-6 *Potentilla tucumanensis*	Cr.CrRLK1	80.6	91.9	3/186 (1.6)
	Pto	67.0	80.5	5/185 (2.7)
paj-1 *Fragaria* × *ananassa*	At.ANX1 CrRLK1L	80.4	91.3	1/184 (0.5)
	Pto	63.4	79	6/186 (3.2)
Pot-5 *Potentilla tucumanensis*	At.FERONIA CrRLK1L	75.0	83.7	0/184 (0.0)
	Pto	64.0	75.3	6/186 (3.2)
SG5 *Phaseolus vulgaris*	At2G23200 CrRLK1L	65.3	80.0	2/170 (1.2)
	Pto	60.2	73.7	5/171 (2.9)
OG5 *Phaseolus vulgaris*	At.ANX1 CrRLK1L	84.3	91.3	2/172 (1.2)
	Pto	63.4	78.5	3/172 (1.7)
SG2 *Phaseolus vulgaris*	At.FERONIA CrRLK1L	77.8	87.7	1/171 (0.6)
	Pto	60.7	75.7	7/173 (4.0)
Tg-10 *Musa acuminata*	Os04g52860 CrRLK1L	88.2	93.5	0/170 (0.0)
	Pto	64.0	69.7	5/172 (2.9)
Tg-11 *Musa acuminata*	Os06g22810 CrRLK1L	91.2	95.3	0/170 (0.0)
	Pto	68.6	80.8	5/172 (2.9)
Tg-12 *Musa acuminata*	Os10g39010 CrRLK1L	73.0	86.8	5/174 (2.9)
	Pto	62.2	77.3	5/172 (2.9)
Tg-13 *Musa acuminata*	At.THE1 CrRLK1L	93.5	98.2	0/170 (0.0)
	Pto	69.8	82.0	5/172 (2.9)
Tg-21 *Musa acuminata*	Os06g03610 CrRLK1L	92.9	96.5	0/170 (0.0)
	Pto	65.5	79.5	5/171 (2.9)
Tg-67 *Musa acuminata*	At.FERONIA CrRLK1L	95.3	98.2	0/171 (0.0)
	Pto	61.8	75.7	6/173 (3.5)
pac.Pt.10.306	At.FERONIA CrRLK1L	94.9	98.3	0/178 (0.0)
	Pto	65.2	77.3	8/181 (4.4)
pac.Pt.10.309	At5G61350 CrRLK1L	83.6	91.8	7/183 (3.8)
	Pto	68.5	81.5	5/178 (2.8)
pac.Ptd.9.41	At.ANX1 CrRLK1L	83.1	93.8	0/177 (0.0)
	Pto	68.2	80.4	5/179 (2.8)
pac.Pt.2.202	HERK2 CrRLK1L	85.9	94.4	0/177 (0.0)
	Pto	66.5	81.0	5/179 (2.8)
pac.Pt.4.11	At.THE1 CrRLK1L	89.1	93.9	0/165 (0.0)
	Pto	71.1	80.1	5/166 (3.0)
pac.Pt.4.8	At5G24010 CrRLK1L	86.6	95.7	0/164 (0.0)
	Pto	62.3	80.2	5/167 (3.0)
pac.Pt.5.110	At2G23200 CrRLK1L	67.9	81.0	4/168 (2.4)
	Pto	63.2	74.9	13/171 (7.6)

Pto primer-derived *Pac* sequences (pac) are included. At = *Arabidopsis thaliana*; Os = *Oryza sativa*; Cr = *Catharanthus roseus*.

### Structural features of the kinase domain: typical, atypical, RD and non-RD kinases

The RLK/Pelle-like fragments of *Pac* spanned subdomain I to subdomain VIII-XI (Table 1). Invariant residues of kinases were all conserved except in five sequences (belonging to LRR XII-L, WAK-like-L and LRR VII-L), which were therefore classified as atypical kinases [53] (for details see Additional file 13).

Based on the crucial role of the RD motif in the catalysis [54], we assessed the nature of *Pac* sequences as RD or non-RD kinases, by verifying the presence/absence of the conserved Arg (R) immediately preceding the invariant catalytic Asp (D) in subdomain VIB of the catalytic domain (Additional file 13). Most possessed the RD-Arg. Thirty-nine sequences out of 111 (35%) showed the feature of non-RD kinases. Of the 34 LRR XII-L sequences, 31 were non-RD kinases, and the vast majority (29) had a Cys in substitution of Arg (Figure 2). A single WAK-like-L sequence was also a non-RD kinase (Figure 3). Seven additional non-RD kinases were found among the RLK-like belonging to the unwanted subfamilies (Additional file 8). Features of the non-RD motif of *Pac* sequences were strongly conserved in the reference sequences of the other plant species, monocot and dicot, with which they grouped (Figure 2, Additional file 8). Specifically *Arabidopsis* LRR XII were nearly all non-RD, with a Cys substituting Arg.

In the CrRLK1L(−L), WAK-like(−L) and LRR XII(−L) groups of *Pac* and *Arabidopsis*, the MEME-based consensus motifs were clearly different from the tyrosine motif and instead there was a clear vicinity with the serine/threonine or the dual specifity motif (Additional file 14).

### Analysis of the activation segment

In protein kinases, the activation segment is the catalytic region and is made up of two recognizable regions: the T-loop in which regulatory autophosphorylation often occurs and the P + 1 loop, which forms the primary binding site for the substrate [55]. The function of several residues, in the activation segment of Pto and BRI1, has been clarified [56, 57]. Our objective was to compare the activation segment of Pto and BRI1 with the corresponding regions in the *Pac* and *Arabidopsis* CrRLK1L(−L), WAK-like(−L) and BRI1(−L). The LRR XII(−L) sequences were not analyzed as they did not span the entire region of the activation segment.

**Figure 6:**
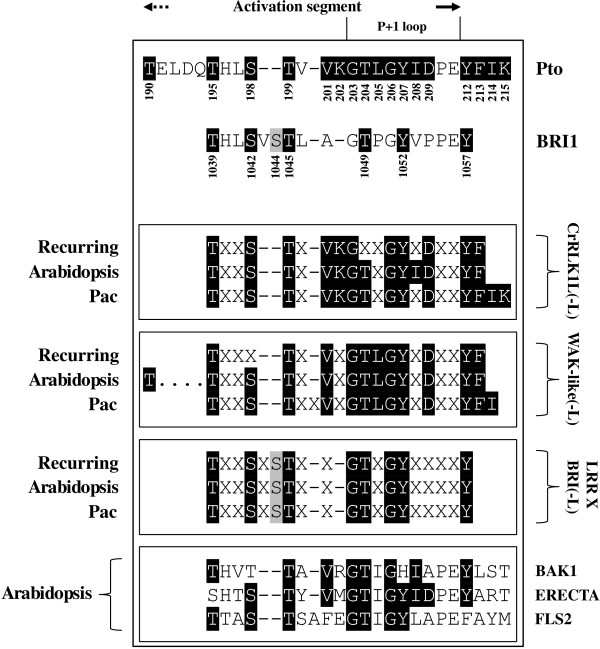
**Functional consensus derived from Pto and BRI1 activation segments inferred from structure/function studies of other authors.** These consensus motifs are compared with counterparts found in the activation segment of RLK/Pelle subfamilies of *Arabidopsis* and *Pac* separately. A consensus motif is also reported for each subfamily, which includes both species and only the most frequent residues of the Pto and BRI1 functional consensus (recurring). ERECTA, FLS2 and BAK1 are also included for comparison. Black-shaded residues define the Pto functional consensus and identities with the other RLK/Pelles. BRI1 consensus residues shared with Pto consensus motif, are shaded black. One residue was found to be exclusive of BRI1 and is shaded grey. In *Arabidopsis* and *Pac* RLK/Pelle subfamilies, residues that do not fall within the consensus positions are not reported (X). The consensus motifs were derived from alignments presented in Additional files 16, 17 and 18, and manually refined.

We identified a motif spanning the activation segment of Pto (Pto motif_190–215_) and BRI1 (BRI1 motif_1039–1057_) and composed of residues that are crucial for their function (Figure 6, Additional file 15), [56–64]. When the two motifs were aligned, six out of seven key residues of the BRI1 motif coincided with key residues of the Pto motif_190–215_ at the corresponding positions (Figure 6, Additional file 15). Specifically alignment of BRI1 Thr-1045 was ambiguous. As this residue is involved in phosphorilation and is coupled to a serine (Ser-1044) similarly to Pto Thr-199, we decided to locate the two threonines in corresponding positions by re-locating the gaps as needed (Figure 6, Additional file 15). As a consequence we choose to consider BRI1 Ser-1044 an insertion, with BRI1 Ser-1042 and Pto Ser-198 located in corresponding positions (Figure 6, Additional file 15). Alternatively BRI1 Ser-1042 may be considered an insertion with Pto Ser-198 and BRI1 Ser-1044 located in corresponding positions (data not shown).

A search for the motifs in the above cited sequences, highlighted that many or all residues recurred in the sequence complements (Figure 6, Additional files 16, 17 and 18). The motifs were also partially conserved in BAK1, ERECTA and FLS2, which were included in the analysis for comparison (Figure 6).

Given the results of the phylogenetic reconstruction and the analyses of structural features (RD, non-RD, typical, atypical motifs and the activation segment), we defined the RLK/Pelle-like of *Pac* as *bona fide* RLK/Pelles.

### Non-synonymous versus synonymous substitution rates, non-synonymous positions and their amino acid variability

**Figure 7:**
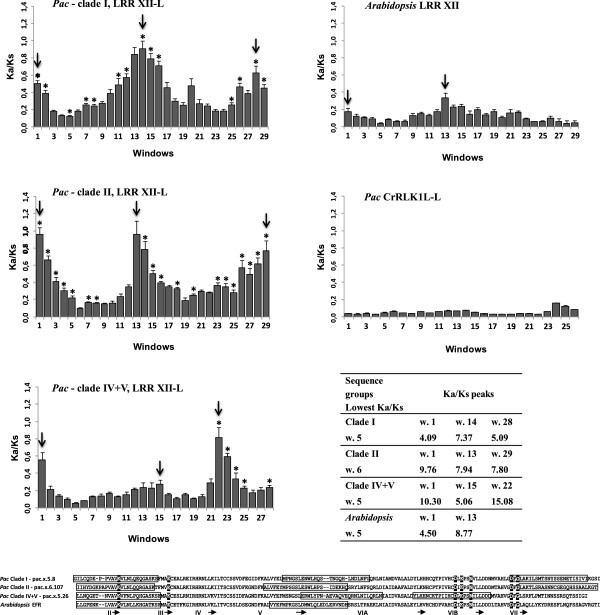
**Kinase domain sub-regions in the LRR XII-L of** ***Pac*** **show signatures of relaxed purifying selection.** Ka/Ks calculation was based on a sliding-window analysis (windows 75 bp, step 15 bp). With regard to the clades of Pac, refer to Figure 2. The *Arabidopsis* LRR XII and the CrRLK1L(−L) of *Pac*, were included in the analysis for comparison and control respectively. The results are shown as average Ka/Ks ratios per window. Vertical bars indicate standard errors of the mean. For each group a representative sequence is shown below in order to map in a frame the windows with the highest Ka/Ks values (arrows in the graphs). The sequences are aligned. Invariant residues of kinases are shaded black. Roman numerals under the alignment identify the beginning of the kinase subdomain motifs and the arrows define the end (see [106]). The asterisks indicate the windows in which Ka/Ks values are significantly higher than those of the corresponding windows of *Arabidopsis* (Wilcoxon- test *pvalue* < 0.0001 and fold increase >2). The table reports the fold change increase in the average Ka/Ks values of each sequence group in evidently relaxed windows (w) compared to those with the lowest values. All the comparisons, except for w.1 vs w.5 in *Arabidopsis*, were significant with our stringent criteria.

In a preliminary work we calculated Ka/Ks values in sequence pairs within CrRLK1L(−L), WAK-like(−L) and LRR XII(−L) groups, for *Pac* and *Arabidopsis* separately. The analysis revealed clear signatures of purifying selection in both species. Interestingly in *Pac*, the distribution of Ka/Ks values of LRR XII-L was shifted in higher values - in any case less than 1 - compared with *Arabidopsis*. A significant increase was also evident in the LRR XII(−L) compared with WAK-Like(−L) and CrRLK1L(−L). These preliminary findings pushed us to focus on the LRR XII(−L) group by running a window-based Ka/Ks analysis in three *Pac* sequence sub-groups separately (clades I, II and IV + V, Figures 2 and 7). The analysis showed an acceleration toward Ka/Ks values higher than 1 in the first, central and final portions of the sequences, which corresponded, in the three groups, to the same sub-domains (Figure 7, Additional file 19). With regard to the average values of Ka/Ks ratio per window, none were significantly larger than 1 (at *pvalue* <0.05). This is in line with a complete relaxation of purifying selection. In the LRR XII of *Arabidopsis*, the analysis was performed in two groups of sequences, one of which comprised AT4G08850 and AT1G35710 which did not align with the others according to a codon-delimited fashion. In *Arabidopsis* average Ka/Ks values ranged from 0.01 to 0.34, highlighting a signature of strong purifying selection. However the pattern of average Ka/Ks values was similar to that observed in *Pac* (Figure 7, Additional file 19). In CrRLK1L-L, the average values ranged from 0.03 to 0.16, thus showing the strongest signature of purifying selection (Figure 7, Additional file 19). In many windows of *Pac* sequences, Ka/Ks values were significantly higher than those of the corresponding windows of *Arabidopsis*. Significant differences were also detected by comparing windows with the highest and the lowest values within each sequence group of *Pac* (see the table in Figure 7), in line with a differential action of the selection in different regions of the kinase domain. With regard to this type of comparison, a significant difference was also detected in *Arabidopsis* (see the table in Figure 7), (Wilcoxon-test *pvalue* <0.0001 and fold increase >2).

In the site by site analysis, the likelihood ratio test did not indicate an overall significantly better fit of the M8 site model (positive selection) versus the M7 model (P > 0.05). The Bayes empirical Bayes method revealed a total of six sites with Ka/Ks ratio >1, in the three sequence groups of *Pac*, although with small posterior probability values (range 56–73%) when accounting for the standard error.

**Figure 8:**
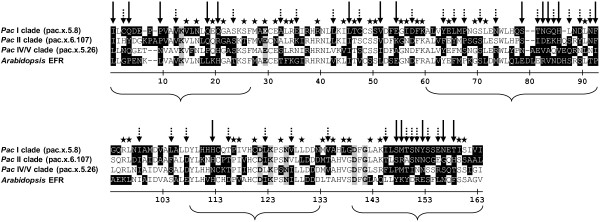
**Amino acid positions of the LRR XII kinase domain subject to non-synonymous nucleotide variations.** Sequences of phylogenetic clades of LRR XII(−L) of *Pac* and LRR XII of *Arabidopsis* were included in the analysis. Positions subject to non-synonymous variations are shaded black. Sequences in the alignment are representatives of each group. Long arrows show non-synonymous positions shared among all sequence groups. Short dotted arrows show non-synonymous positions shared among three groups of sequences. Stars above the alignments show non-synonymous positions that are exclusive to a single group of sequences. The invariant residues of the kinase superfamily (from III to IX) are in bold and shaded grey. The brackets indicate the regions where purifying selection is more relaxed, based on the analysis presented in Figure 7.

A sequence analysis of each subgroup of LRR XII(−L) and of *Arabidopsis* LRR XII showed that of the 158 positions analysed (excluding gaps), 126 positions were subject to non-synonymous variations, of which 88 (70%) were shared among two or more sequence groups, and 19 (24%) were present in all the sequence groups (Figure 8). The amino acids recurring in all the shared positions and for each group of sequences are listed in Additional file 20.

## Discussion

Genome-wide analysis for the characterization of gene families in fully-sequenced species is invaluable for an in-depth comprehension of the information contained in the genomes (see for example: [4, 65, 66]). It is also a necessary support for gene candidate analyses such as those based on domain-targeted isolations of gene groups, in species lacking genome sequencing. We thus based our study on a detailed comparison with RLK/Pelles of fully-sequenced species, especially *Arabidopsis*, and devised a method for the correct identification of RLK/Pelle gene fragments.

In this study we present some substantial advances on plant RLK/Pelles. We isolated for the first time a set of RLK/Pelles in *Pac*. Interestingly, the analysis of sequence data also revealed: i) a novel phylogenetic relationship between the *Pto* group and the CrRLK1L RLK/Pelle subfamily; ii) clear signatures of relaxed purifying selection in the LRR XII-L, which had not been previously revealed for plant RLK kinase domain.

The representativeness of the kinase domain for RLK/Pelle classification has been widely shown [3–5, 7, 67, 68]. Kinase-domain targeted isolation of RLK/Pelles has also been found to be successful [69–75]. In this work we confirmed its representativeness for a correct classification, by matching the cloned sequences with long 454 isotigs from *Pac*, which displayed the expected extracellular domain signatures (Additional file 5).

### CrRLK1L candidates and the relationship between CrRLK1L and Pto

All the analyses highlighted our apparent failure to collect *sensu strictu Pto*-like genes in *Pac* and showed that the analysed non-solanaceous plant genomes lack a *bona fide Pto* gene group. Interestingly, in the phylogenetic analyses, Pto group was closest to CrRLK1L than any other RLK subfamily. Note that i) best matches of Pto, belonging to six fully-sequenced dicot species and to rice, were revealed as CrRLK1L; ii) all non-solanaceous Pto-like partial sequences were classified as CrRLK1L-like; in this regard it is explicative the high closeness of Pto-like partial sequences from bean and cultivated/wild strawberries to CrRLK1L extracted from bean and *F. vesca* genomes (Figure 5, Additional file 11). All the dicot species that we used in the Pto/CrRLK1L comparative analysis were Rosids, except the solanaceous species which belong to Asterids, and *Pac*, whose formation predates the split giving rise to Rosids and Asterids (http://www.mobot.org/MOBOT/research/APweb/). All this clearly points to the restriction of *Pto* gene group to a range of taxa within Dicots, possibly due to a relatively more recent origin than other ubiquitous RLK/Pelle subfamilies. On the other hand a more precise evaluation of the taxonomic restriction of *Pto*-like genes is needed and would require sequencing additional plant taxa representative of Asterids. The taxonomic restriction of an RLK group is not surprising as species-specific RLK/Pelle subfamilies have previously been reported [5, 7].

The CrRLK1L subfamily is an ancient group of RLK/Pelles with a conserved extracellular domain and whose formation predates the divergence of vascular plants [5, 50]. This was confirmed by our phylogenies which clearly showed that orthologs are more similar than paralogs, and suggests that events giving rise to family members occurred prior to the monocot-dicot split and after the divergence rate was low (Figures 1 and 5, Additional files 7 and 12).

Strong evidence thus suggests that *Pto* genes evolved from ancient CrRLK1L, through the loss of the extracellular domain: i) the ubiquity of the CrRLK1L in the land plant and the conservation of this family over a long geological period [5, 50]; ii) the restriction of *Pto* and its paralogs to asterid species (i.e. Solanaceae, based on current knowledge) (Figure 5, Additional files 11 and 12); iii) the close vicinity between the kinase domain of the two RLK/Pelle groups (Figures 1 and 5, Additional files 6 and 12) [6]. Interestingly, the structure of the *Pto* group is also similar to CrRLK1L in that orthologs are more similar than paralogs [76]. Recently the tomato RLK/Pelle family has been characterised and the phylogenetic comparison with *Arabidopsis* showed that a CrRLK1L subfamily exists in tomato [6]. Interestingly we noted that Pto group was included in the CrRLK1L clade. Though this was not discussed by the authors it represents a full confirmation of the results of our phylogenies [6]. Phylogenetic vicinity between the kinase domains of RLK and RLCK subfamilies has been reported, and domain reassortment has been repeatedly emphasized as an ancient mechanism of RLK/Pelle evolution in the land plant lineage [4, 5, 77]. CrRLK1L is one of the *Arabidopsis* RLK/Pelle subfamilies which has corresponding RLPs (receptor–like proteins, i.e. proteins resembling the extracellular domain of RLKs), suggesting that these RLKs and their RLPs gave rise or were each derived from their counterpart [4]. In this scenario, our conclusion on the formation of RLCKs - *Pto* and paralogs - from RLKs - CrRLK1L - is thus in agreement with the accepted RLK/Pelle gene evolution model.

The vicinity between *Pto* group and CrRLK1L raises the question if they have to be classified as *bona fide* CrRLK1L or represent the founding members of a novel RLCK/Pelle subfamily, related to CrRLK1L. This will be a matter of investigation and debate in the future.

The analysis of the activation segment was also useful for shedding light on the relationship between CrRLK1L(−like) and Pto (Figure 6). The functional motifs identified in the activation segment of BRI1 and Pto (Figure 6, Additional file 15), were partially overlapping and were conserved among a wide range of RLK/Pelles. This highlights an interesting point which fits the special case of CrRLK1L/Pto well: the conservation of functional residues can be useful for supporting identification at a high hierarchical level but it is not sufficient to suggest a relationship of putative orthology, even when this conclusion is sustained by phylogenetic vicinity - as is the case of the non-solanaceous Pto-like vs solanaceous Pto group [73, 74].

The recovery of a CrRLK1L(−L) gene set in *Pac*, is still an interesting finding, also in the context of defense-related RLK/Pelles. For example THE1 mediates the response of plant cells to the perturbation of cellulose synthesis by triggering growth inhibition, ectopic lignin accumulation and the expression of a set of defense-related genes. THE1 is thus considered as a sensor of cell wall integrity and a role in pathogen defense has been postulated [78, 79]. Another suggestion for the role of CrRLK1Ls in pathogen defense is that, in *Arabidopsis*, the bacterial effector AvrPto directly binds to the CrRLK1L member AT2G23200, suggesting a significant, yet undiscovered role, in innate immunity [63].

### LRR XII candidates: nonRD motif and relaxed purifying selection highlight a potential link with pathogen resistance

In *Arabidopsis* and rice the LRR XII subfamily includes PRRs functioning as PAMP receptors and R genes [10, 11, 80] and it has been suggested that this is the general functional contribution of the subfamily. An LRR XII-L group was clearly identified in *Pac* (Figure 2, Additional file 6). The wide nucleotide diversity range among all *Pac* sequences (nucleotide score: 62–98) and a comparison, in the phylogenetic tree, of the branching pattern of *Pac* and *Arabidopsis* sequences, suggest that an undetermined number of paralogs had been isolated (Figure 2).

An important feature shared by the *Arabidopsis* and *Pac* sequence complements is their nature as non-RD kinases and the high frequency of Cys in substitution of the RD-Arg (Figure 2), which strongly supports the phylogenetic identification. Non-RD kinases are present in the kinomes of a wide range of Eukaryotic organisms and a statistically significant and positive correlation was found between kinases that function in the innate immunity and the non-RD motif [81]. This finding, together with the fact that *Arabidopsis* and rice LRR XII contain PRRs, suggests that we have identified a group of genes that is potentially involved in the perception of pathogens.

Further support for this hypothesis is that we also detected signatures of a relaxed purifying selection in *Pac* sequences (Figure 7). This finding is new for plant RLK/Pelles. In fact in *Arabidopsis* and rice, signatures of positive selection were detected in the extracellular domains [7, 82, 83]. In contrast, the kinase domain has been shown to be under a strong purifying selection [7].

Interestingly, only specific regions of the kinase domains were subject, in *Pac*, to an evidently relaxed purifying selection and the substitutions occurring in the the non-synonymous positions were largely shared by the different sequence groups of *Pac* (Figures 7 and 8, Additional file 20). This suggests the action of a selective pressure that tends to keep specific non-silent variations in regions which might be relevant for the fitness. Indeed these regions span zones which are crucial for the function of the kinase catalytic core: i) I-II subdomain, which is involved in anchoring the ATP phosphates; ii) VII-VIII subdomain, the activation segment; iii) V-VIA subdomain, which is the site of catalysis [84, 85]. The fact that the strength of purifying selection can be differentially modulated in the distinct functional regions of a protein coding gene is well known (e.g. [86, 87]), however it is novel for plant RLK/Pelles.

Purifying selection generally eliminates any mutations in order to preserve the exact function of essential genes. On the other hand, a relaxation of purifying selection and positive selection are considered as two non-mutually exclusive forces that drive the protein evolutionary rate towards divergence. In particular in the relaxed purifying selection, random mutations affect genes that are dispensable or redundant, therefore there is no stringent constraint to impede their retention. By chance, these mutations may turn out to be beneficial for morphological and functional specification [88–90]. In fact a relaxed selective constraint plays a role in the expression of phenotypic plasticity, which is one of the most important ways by which organisms adaptively interact with the environment [90]. Specifically, the evolution of caste systems in polyphenic social insects [90], the fast evolution of lineage-specific genes in humans [91] and a variation in freezing tolerance among natural accessions of *Arabidopsis*[92] have all been attributed to a relaxed purifying selection as the main driving force.

It is well known that effectors of pathogenic bacteria target the kinase domain of several RLK/Pelles with a role in plant immunity, giving rise to an “arms race” ending with host resistance or susceptibility [20, 62, 63, 93–96]. Thus in *Pac*, a relaxation of purifying selection in the kinase domain of LRR XII-L, may have enabled the recruitment of variants which are useful for the resistance response of *Pac* to some microbial (would be) pathogens. This might be an exciting research theme for the future.

### WAK-like and BRI1 group candidates

WAK-like proteins link the extracellular matrix to the cytoplasm for appropriate signal transmission [97]. Wak-like have been found to be involved in many functions including pathogen resistance [12, 98, 99] and development [100, 101]. Despite our initial aim to isolate a single WAK-like member - namely an RFO1 homolog - a WAK-Like-L gene complement was isolated in *Pac* (Additional file 6) and seemed highly representative of the phylogenetic structure of the *Arabidopsis* counterpart (Figure 3). Importantly, results of WAK-like phylogeny agree exactly with the phylogenetic comparison between WAK-like of *Arabidopsis* and rice, in which sequences clustered in a species-specific manner and with only one multi-species clade headed by *Arabidopsis* WAKL14 and WAKL21 (Figure 3) [67]. However it remains difficult to predict the level of completeness of the gene complement isolated in *Pac*. In fact, compared with the *Arabidopsis* genome, an extensive expansion of the gene family has occurred both in rice and in poplar suggesting a lineage-independent evolution [5, 67]. In any case the formation of species-specific and multi-species clades, in the *Arabidopsis*/*Pac* (this work) and *Arabidopsis*/rice phylogenies [67], suggests that opposing evolutionary forces shaped this RLK/Pelle subfamily, which produced both genetic divergence and conservation. This is in line with the involvement of these RLKs in very different functions, i.e. development and pathogen resistance, which exploit genetic conservation and diversification, respectively.

In the light of our results, as RFO1 belongs to a species-specific clade, phylogeny-based identification of the potential RFO1 homologs/orthologs was not possible. However based on the branching pattern of the phylogenetic tree, clade I appears to be the group of genes in which it might be possible to search for homologs with RFO1-related functions, i.e. resistance to vascular pathogens (Figure 3). This is particularly important in *Platanus* species which are hyper-susceptible to the vascular pathogen *C. platani*[26]. In *Arabidopsis*, a number of additional WAK-like are involved in stress responses, namely WAK1, WAK2, WAKL5 and WAKL7, which were found to be inducible by salicylic acid (SA) and by wounding, and to protect plants from detrimental effects during pathogen responses, such as high levels of SA [98, 100, 101]. In terms of finding orthologs of these WAK-like, clade II of *Pac* might be a candidate source, as it shares a 4-residue motif exclusively with the WAK1-headed clade, suggesting a genetic correlation (Additional file 9).

The topology of LRR X-BRI1-L phylogeny was the same as that found by Cano-Delgado et al. [102] based on full-length proteins of BRI1(−like) from plant species, which confirms once more that the kinase domain is of primary importance in RLK/Pelle subfamily identification and phylogeny. Similarly to CrRLK1(−L) phylogeny, the three major clades included the potential orthologs from different dicot and monocot species, implying that diversification within this gene group predates the monocot/dicot split (Figure 4). Our analyses suggest that potential orthologs of BRI1 group members were isolated in *Pac*. In *Arabidopsis*, BRLs are expressed in the vasculature where they have a role in provascular cell growth and contribute to establishing the xylem/phloem pattern through perception of the BR signal [102, 103]. In addition, BRLs also seem to be important in stress responses. In fact, after confirmation that BRI1 was not the functional receptor of systemin [18, 19] a BRL was suspected to be the true systemin receptor since BRLs match the highly specific localization of prosystemin and systemin perception in the vasculature [104, 105]. Several *Pac* diseases target vascular tissues, cambium and living bark, and the pathogens gain entry through wounds [27]. Therefore, LRR X-BRI1-L of *Pac* are important candidates in the study of the host response to wound pathogens, such as *C. platani*.

## Conclusions

The study of the RLK/Pelles of *Pac* led to interesting findings, highlighting some aspects of *Pac* as a model species. This is also supported by the representative position of *Platanus* within Dicot phylogeny, one of the earliest branches, temporally located before the split which gave rise to the main groups, Rosids and Asterids, which comprise the majority of cultivated Dicots (http://www.mobot.org/MOBOT/research/APweb/.).

In addition the region-specific signatures of relaxed purifying selection in the non-RD LRR XII-L genes, highlights that this gene group is a good candidate for investigations into pathogen resistance.

With regard to CrRLK1L/Pto, it will be exciting to verify in which taxonomic groups, apart from solanaceae, *sensu strictu Pto* genes are currently present in order to further clarify the evolutionary link with CrRLK1L.

Above all, the main objective of our work in *Pac* was to create the first valuable resource to analyze the RLK kinome expression of genotypes that resist or are overwhelmed by pathogens. We believe that this represents the first step in an in-depth study on stress perception systems and also in the identification of useful molecular markers for genetic mapping and selecting resistant genotypes.

### Availability of supporting data

The data set supporting the results of this article is available in the NCBI GenBank repository, (see also Additional file 3 for accession numbers and the text of nt and aa sequences).

## Electronic supplementary material

Additional file 1: **Degenerate primers successfully used for PCR amplifications of kinase domain of RLK/Pelle genes.** (PDF 129 KB)

Additional file 2: **Primer pairs used to isolate RLK/Pelle gene fragments and efficiency of the gene fishing process.** (PDF 14 KB)

Additional file 3: ***Platanus*** **×** ***acerifolia*** **(** ***Pac*** **) RLK/Pelles: NCBI GenBank accession numbers, subfamily classification and sequence text.** Sequences in **A** and **B** were obtained in this work, those in **C** were used for comparison. In **D** nucletide and amino acidic sequences are listed. (PDF 319 KB)

Additional file 4: **Reference sequences included in the phylogenies for comparison and to root the trees.** Where RLK subfamilies or generic terms (i.e. Pto-like) are cited, all sequences reported in the source were tested in the phylogenies, except pseudogenes or putative proteins with truncated kinase domains. (PDF 23 KB)

Additional file 5: **The extracellular domain of** ***Platanus*** **×** ***acerifolia*** **(** ***Pac*** **) RLK/Pelles.** Pairwise comparison between selected gene fragments obtained in this work, and the overlapping RLK/Pelle isotigs extracted from a 454 FLX dataset of a *Platanus* × *acerifolia* (*Pac*) transcriptome (Pilotti M., Brunetti A., Iacono M. and Pindo M. unpublished data). **A** Spanning regions and identity values. **B** The isotigs display a region 5′-upstream the kinase domain, which includes putative extracellular domains typical of the RLK/Pelle subfamilies dealt with in this work (output data from Pfam). This is a confirmation of the phylogenetic identification of the gene fragments. A SMART analysis predicted in a confident manner transmembrane domains in all the isotigs, which were located upstream the kinase domain and downstream the putative extracellular domains. (PDF 110 KB)

Additional file 6: **Phylogenetic classification of RLK/Pelles of** ***Platanus*** **×** ***acerifolia*** **(** ***Pac*** **).** The analyses were based on the Neighbor-joining method and show a general view of the relationship of the aa sequences of RLK/Pelles of *Pac* (pac, ♦ in black), Pto group (♦ in red) and other kinases. **A**. Contains representative sequences of the following species/groups: *Pac*, Pto group (red rhombus), Pto-like partial sequences from non-solanaceous species (white rhombus with a red outline), RLK/Pelle subfamilies of *Arabidopsis*[4], other families of the receptor kinase group – receptor tyrosine kinase (RTK) and Raf [3] - and other eukaryotic protein kinases (ePKs). The tree is rooted with a bacterial protein kinase [APH(3′)III also named aminoglycoside 3′-phosphotransferase]. **B**. Contains all *Pac* sequences reported in this work and their strict *Arabidopsis* homologs according to what emerged from **A**. A kinase domain region spanning subdomains I/II to the beginning of VIII, was used to infer the trees. See caption of Figure 1 and Methods for details on the representation of the trees. (PDF 90 KB)

Additional file 7: **The CrRLK1L-L of** ***Platanus*** **×** ***acerifolia*** **(** ***Pac*** **).** Neighbor-joining analysis to compare CrRLK1L-L of *Pac* (pac, ♦), with the complete CrRLK1L subfamily of *Arabidopsis* and rice; Pto and paralogs of *Solanum pimpinellifolium* were also included (red rhombus); The subdivision into CRPK1-like 1 and CRPK1-like 2 is from Shiu and Bleecker [4]. aa sequences were used to infer the tree. Analysis was based on the kinase domain region spanning subdomain I through to IX. See caption of Figure 1 for details on the representation of the tree. Arrows indicate *Arabidopsis* CrRLK1L which were functionally characterised. (PDF 47 KB)

Additional file 8: **Unwanted RLK/Pelles of** ***Platanus*** **×** ***acerifolia*** **(** ***Pac*** **).** Sequences were obtained with primers aimed at isolating members of LRR XII subfamily. **A** Neighbor-joining tree based on the aa sequences of *Pac* (pac, ♦), the nearest RLK/Pelle members of *Arabidopsis* (*◊*) and other unknown, homologous sequences of rice and poplar. Analysis was based on the kinase domain region spanning subdomains I/II to the beginning of VIII. See caption of Figure 1 for details on the representation of the tree. **B** Multiple alignment of RD motif (shaded grey), non-RD motif (shaded black) and an atypical variant of the non-RD-Asp (shaded black). **C** Phylogenetic analysis was ambiguous in determining the homology relationship of pac.Erf.2, pac.Erf.9 and pac.Erf.13 with the *Arabidopsis* RLK/Pelle subfamilies GDPD, LRK10L-2, CRPK1-like2 (i.e. CrRLK1L) and Thaumatin. The table thus shows the identity and similarity values and number of gaps derived from the pairwise comparison of these sequences with one *Arabidopsis* relative for each subfamily which, in preliminary analyses, were the nearest sequences. (PDF 172 KB)

Additional file 9: **Wak1-clade-specific amino acid motif helps to identify close homologs in** ***Platanus*** **×** ***acerifolia*** **(** ***Pac*** **).** Amino acidic residues (shaded black) conserved among sequences of Wak1-headed clade of *Arabidopsis* (RLK/Pelle subfamily WAK-like) and of the II phylogenetic clade of Wak-like-L of *Pac* (refer to phylogeny of Figure 3). This motif suggests a genetic correlation between the two sequence groups. (PDF 130 KB)

Additional file 10: **Classification of** ***Pac*** **sequences based on maximum-likelihood (ML) phylogenetic analyses.** Analyses were performed on the same dataset alignments used for crucial Neighbor-joining trees presented in this work (aa sequences). A1 and A2 match with Additional file 6B (all *Pac* sequences compared with homologous *Arabidopsis* RLK Pelles); B matches with Additional file 7 (*Arabidopsis* and rice CrRLK1L vs *Pac* CrRLK1L-L); C matches with Figure 2 (*Arabidopsis* LRR XII vs *Pac* LRR XII-L); D matches with Figure 3 (*Arabidopsis* WAK-like vs *Pac* WAK-like-L); E with Figure 4 (*Arabidopsis* LRR X vs *Pac* LRR X-BRI1-L group). Editing matches that of counterpart figures. All trees were inferred with Mega 6 except the one represented in A2 that was TREE PUZZLE-implemented. With regard to Mega 6 analyses, the percentage of trees in which the associated sequences clustered together in the bootstrap test (1,000 replicates) is shown for each node; trees are drawn to scale in the number of substitutions per site (scale at the bottom). TREE-PUZZLE analysis was conducted with 50,000 puzzling steps and quartet puzzling support values are shown for each node. (PDF 161 KB)

Additional file 11: **Phylogenetic relationship between Pto (−like) (** ***sensu strictu*** **and** ***sensu latu*** **) and the subfamily CrRLK1L by two methods of maximum-likelihood analysis.** Analysis was performed on the same dataset alignment used for the analysis presented in Figure 5. The following aa sequences were included: Pto locus and solanaceous Pto-like; CrRLK1L of *Arabidopsis*, rice and *Catharanthus roseus*; Pto best blastp matches (namely CrRLK1L, based on domain composition) of *Arabidopsis*, rice, grapevine, poplar and castor bean; Pto-like partial sequences (namely CrRLK1L-like, i.e. Pto-like *sensu latu*) of non-solanaceous species (i.e. *Phaselus vulgaris*, cultivated and wild strawberry and *Musa acuminata*); best blastp matches from *F. vesca* and *P. vulgaris* (namely CrRLK1L) obtained using Pto-like partial sequences as query. Representative *Pac* sequences obtained with *Pto*-primer (CrRLK1L-like) were also included (pac). The arrows indicate solanaceous *so-called* Pto-like sequences, which clustered within a CrRLK1L-headed clade. The star indicates a CrRLK1L-like of castor bean lacking the extracellular domain typical of the subfamily. **A**: tree obtained with Mega 6; the percentage of trees in which the associated sequences clustered together in the bootstrap test (1,000 replicates) is shown for each node; trees are drawn to scale in the number of substitutions per site (scale at the bottom). **B**: tree obtained with TREE-PUZZLE; analysis was conducted with 50,000 puzzling steps and quartet puzzling support values are shown for each node. See Methods for details. (PDF 115 KB)

Additional file 12: **Phylogenetic analyses (NJ and ML) to compare the complete CrRLK1L subfamily (CRPK1-like 1) of different plant species and Pto group.** We included the complete CRPK1-like 1 aa sequence set of *Arabidopsis*, poplar, tomato (*Solanum lycopersicum*), rice and moss, and Pto and paralogs of *Solanum pimpinellifolium* (see Additional file 4 for sequence sources)*.* The NJ tree is presented in **A**, the ML trees, Mega6 and TREE-PUZZLE-implemented, are presented in **B** and **C** respectively. Analyses were based on the complete kinase domain region. With regard to Mega 6, the percentage of trees in which the associated sequences clustered together in the bootstrap test (1,000 replicates) is shown for each node; trees are drawn to scale in the number of substitutions per site (scale at the bottom). With regard to TREE-PUZZLE analysis was conducted with 50,000 puzzling steps and quartet puzzling support values are shown for each node. See Methods for additional details. (PDF 220 KB)

Additional file 13: **The molecular role of some invariant residues of the kinase domain.** Substitutions in *Platanus* × *acerifolia* (*Pac*) sequences (atypical and non-RD motifs) are listed. (PDF 82 KB)

Additional file 14: **Serine, threonine and tyrosine phosphorilation specificity motifs of RLK/Pelles of** ***Platanus*** **×** ***acerifolia*** **(** ***Pac*** **) and** ***Arabidopsis*** **.** The motifs are compared with phosphorilation specificity motifs of protein kinase previously inferred [42]. Consensus was determined using MEME (a motif-based sequence analysis tool). (PDF 128 KB)

Additional file 15: **Crucial residues of the activation segments of Pto and BRI1.** The relevant literature is cited in the text. (PDF 24 KB)

Additional file 16: **Important residues of CrRLK1L located in the kinase domain region spanning subdomains VIB to VIII.** The alignment shows the functional consensus derived from the Pto and BRI1 activation segment (subdomains VII and VIII) (Figure 6, Additional file 15) and its conservation in CrRLK1L(−L) subfamilies of *Arabidopsis* and *Platanus* × *acerifolia* (*Pac*). Black-shaded residues define the Pto functional consensus and the identities with CrRLK1L(−L). BRI1 consensus residues shared with Pto consensus motif, are shaded black, the residue exclusive of BRI1 consensus is shaded grey. Shaded-grey gaps have been inserted in order to match sequences with BRI1 consensus. The figure also shows the RD/non-RD motif and the regions that are useful to infer the ser/thr, tyr and the dual phosphorylation specificities of protein kinases [42] (Additional file 14) [red-shaded residues (subdomain VIb) and the P + 1 loop (subdomain VIII)]. (PDF 166 KB)

Additional file 17: **Important residues of Wak-like located in the kinase domain region spanning subdomains VIB to VIII.** The alignment shows the functional consensus derived from the Pto and BRI1 activation segment (subdomains VII and VIII) (Figure 6, Additional file 15) and its conservation in the WAK-like(−L) subfamilies of *Arabidopsis* and *Platanus* × *acerifolia* (*Pac*). Black-shaded residues define the Pto functional consensus and the identities with WAK-like(−L). BRI1 consensus residues shared with Pto consensus motif, are shaded black, the residue exclusive of BRI1 consensus is shaded grey. Shaded-grey gaps have been inserted in order to match sequences with BRI1 consensus. The figure also shows the RD and non-RD motif and the regions that are useful to infer the ser/thr, tyr and the dual phosphorylation specificities of protein kinases [42] (Additional file 14) [red-shaded residues (subdomain VIb) and the P + 1 loop (subdomain VIII)]. (PDF 165 KB)

Additional file 18: **Important residues of LRR X-BRI1 group located in the kinase domain region spanning subdomains VIB to VIII.** The alignment shows the functional consensus derived from the Pto and BRI1 activation segment (subdomains VII and VIII) (Figure 6, Additional file 15) and its conservation in the LRR X-BRI1(−L) group of *Arabidopsis* and *Platanus* × *acerifolia* (*Pac*). Black-shaded residues define the Pto functional consensus and the identities with LRR X-BRI1(−L). BRI1 consensus residues shared with Pto consensus motif, are shaded black, the residue exclusive of BRI1 consensus is shaded grey. The figure also shows the RD motif and the regions that are useful to infer the ser/thr, tyr and the dual phosphorylation specificities of protein kinases [42] (Additional file 14) [red-shaded residues (subdomain VIb) and the P + 1 loop (subdomain VIII)]. (PDF 169 KB)

Additional file 19: **Ka/Ks values obtained for each window in the sliding window analysis of** ***Platanus*** **×** ***acerifolia*** **(** ***Pac*** **) LRR XII-L.** CrRLK1L-L of Pac and LRR XII of *Arabidopsis* are included for comparison. (PDF 61 KB)

Additional file 20: **Amino acid variability at the kinase domain positions subject to non-synonymous variation.** Analysis focuses on phylogenetic clades of LRR-XII-L of *Platanus* × *acerifolia* (*Pac*) and LRR-XII of *Arabidopsis* (refer Figure 2). The three distinct tables present the data regarding the sharing of the variable positions among four, three and two distinct group of sequences. (PDF 183 KB)
