# Supplementary material for: Kinase domain-targeted isolation of defense-related receptor-like kinases (RLK/Pelle) in Platanus × acerifolia: phylogenetic and structural analysis
Source: BMC Res Notes. 2014 Dec 8;7:884. doi: 10.1186/1756-0500-7-884 (PMC4295470; doi:10.1186/1756-0500-7-884)
Supplement: Supplementary file 1 — Additional file 1: Degenerate primers successfully used for PCR amplifications of kinase domain of RLK/Pelle genes. (PDF 129 KB) [file 13104_2014_3456_MOESM1_ESM.pdf]

Additional file 1. Degenerate primers successfully used for PCR amplifications of kinase domain of RLK/Pelle genes.

Primer name [a, b]	Annealing site [c]	Sequence (5'→3') [d]	Deg. degree	Targeted amino acid sequence [e]
Xa21.F.I [α]	Subd. I	GGMASYTTYGGKACTGTDTAYAA	1/192	GXFGTVYX
Xa21. F.II [α]	Subd. I	GGDTCWGGMASYTTYGGWTCTGT	1/192	GSGXFGSV
Xa21.F.Ispec [α]	Subd. I	GGMTSMTTTGGMTCAGTATAYAA	1/32	GXFGSVYX
Xa21.R.I-gl [α]	Subd. VIII	CCATAYTCTGGWGCDRCATAKCC	1/48	GYXAPEYG
Pto.F.IB [β]	Subd. I	GGYGSMTTYGGGAWGGTTTWYARRGG	1/512	GXFGXVXXG
Pto.F.II [β, γ]	Subd. I	GGAKWGGGTGSMTTTGGRAMRGTTTA	1/128	GXGXFGXVX
Pto.F.IA.n [β]	Subd. I	GGACWKGGYGKMTTYGGGAWGGTTT	1/128	GXGXFGXVX
Pto.F.IB.n [β, δ]	Subd. I	GGYGKMTTYGGGAWGGTTTWYARRGG	1/512	GXFGXVXXG
Pto.F.II.pl [γ]	Subd. I	GGACWTGGTGTCTTTGGRAMRGTTTA	1/16	GXGVFGXVX
Pto.R.I [δ]	Subd. IX	ACWACACCRAAWGAATAMACRTC	1/32	DVYSFGVV
Pto.R.II.A [β]	Subd. IX	GCRGRYCTAGCRCAAAGWACKTC	1/64	XVLCARXA
Pto.R.II.B [γ]	Subd. IX	GCMGRCCTWGCACAMARWACYTC	1/128	EVXCARXA
WAK1.F [ε]	Subd. I	GGCYAWGGAACRGTBTACAAAGG	1/24	GXGTVYKG
WAK1.R [ε]	Subd. IX	TCCATWAGGACDACYCCRAARCTAT	1/48	SFGVVLMX
WAK.Ar.A.F [η]	< Subd. I	AGAGYTDGARAAAGCYACYGADAAC	1/144	XEXEKATXN
WAK.Ar.A.R [η]	Subd. IX	CCRAARCTATARACATCACTYTTNTC	1/16	XKSDVYSFG
WAK.Ar.B.F [θ]	< Subd. I	CYGGCARAGAAATCRYSAAAGCMAC	1/64	XGXEIXKAT
WAK.Ar.B.R [θ]	Subd. IX	CACAACCCCGAAGCTRTACACRTCGC	1/4	DVYSFGVV
WAK.Ar.C.F [κ]	< Subd. I	GAGARAGCDACMRATRGTTTCTCYG	1/96	EXATXXFSX
WAK.Ar.C.R [κ]	Subd. IX	ACVACYCCRAAGCTRTASACRTCRCTC	1/192	SDVYSFGVV
WAK.Vt.A.F [λ]	Subd. I	GRRTTMTTGGWCRWGGARGYCAAGG	1/128	XXXGXGXQG
WAK.Vt.A.R [λ]	Subd. IX	AACWATTCCAAARCTATAKACATCAC	1/8	DVYSFGXV
WAK.Vt.B.F [μ]	< Subd. I	GARGAGYTGSARARRGCMACAGAT	1/128	EEXXXATD
WAK.Vt.B.R [μ]	Subd. XI	MAAYTTSTTTCATKGTTGGYCTTTT	1/32	KRPTMKXXX
EFR.F.I [μ]	Subd. I	WTTYGGYWMWGTRTWYAAAGG	1/512	FGXVXKG
EFR.R.I [μ]	Subd. VIII	CCATAYTCTGGHGCKGCATAKCCRAT	1/48	IGYAAPEYG
EFR.R.II [μ]	Subd. IX	ATYCCRWAGCTGTAMAYATCTCC	1/32	GDXYSXGX
Xa26.F [ν]	Subd. I	GGVKYYGGAAGYTTTGGWAAWGTTT	1/192	GXGSFGXVX
Xa26.R.II [ν]	Subd. IX	ATGATSCCRWARCTGAAYACATCRCT	1/64	SDVFSXGIX
BRI1.F [π]	< Subd. I	GRAARCTYACNTTYGCDGATCTTCT	1/192	KLTFADLL
BRI1.R [π]	Subd. XI	CKGCTTGDATYTCCTTRAACWTNGCC	1/192	AXFKEIQAX
BRL1.F [χ]	< Subd. I	TWGAAGCBACMAAYGGBTTYAG	1/144	EATNGFX
BRL1.R [χ]	Subd. X	CCCAYCCNACMAGRTTRTTRTCWTC	1/256	XDNNLVGWX
BRL2.FII [ψ]	< Subd. I	ACAACATGGAARATTGACAARGAGAAAGA	1/4	TTWKIDKEKX
BRL2.FIII [ω]	< Subd. I	CTCATTGARGCYACCAAYGGCTTCTCWGC	1/16	LIEATNGFSA
BRL2.RI [ψ]	Subd. X	CACCCAHCCCACCAARTTRGTGTCKCC	1/24	GDTNLVGWV
BRL2.RII [ω]	Subd. XI	CCAGGYATCAGCTCYCTCAACATGGCYAC	1/8	VAMLRELXPG

[a] Primer codes contain the reference gene acronyms (*Xa21*, *Xa26*, *EFR*, *Pto*, *BRI1*) or the structural gene class acronyms (WAK kinase, which include *RFO1*) from which they were generated; forward and reverse primers are indicated by the letters F and R, respectively, within each code. [b] Forward primers were paired with reverse primers showing the same Greek letter. [c] < =upstream. [d] Degenerate IUPAC 1-letter code abbreviations are used. [e] X represents a variable residue as computed by the Emboss Transeq tool.
