# Supplementary material for: Kinase domain-targeted isolation of defense-related receptor-like kinases (RLK/Pelle) in Platanus × acerifolia: phylogenetic and structural analysis
Source: BMC Res Notes. 2014 Dec 8;7:884. doi: 10.1186/1756-0500-7-884 (PMC4295470; doi:10.1186/1756-0500-7-884)
Supplement: Supplementary file 2 — Additional file 2: Primer pairs used to isolate RLK/Pelle gene fragments and efficiency of the gene fishing process. (PDF 14 KB) [file 13104_2014_3456_MOESM2_ESM.pdf]

Additional file 2. Primer pairs used to isolate RLK/Pelle gene fragments and efficiency of the gene fishing process.

Primer pairs		RLK / n.Seq. [a]	Primer pairs		RLK / n.Seq. [a]
For	Rev		For	Rev	
Xa21.F.I	Xa21.R.I-gl	26/26	Pto.F.IB	Pto.R.IIA	5/5
Xa21. F.II	Xa21.R.I-gl	17/17	Pto.F.II	Pto.R.IIA	5/5
Xa21.F.Ispec	Xa21.R.I-gl	1/4	Pto.F.II	Pto.R.II.B	4/5
Xa26.F	Xa26.R.II	7/8	Pto.F.IA.n	Pto.R.II.A	7/7
EFR.F.I	EFR.R.I	13/13	Pto.F.IB.n	Pto.R.I	6/6
EFR.F.I	EFR.R.II	3/8	Pto.F.IBn	Pto.R.II.A	9/10
WAK1.F	WAK1.R	14/14	Pto.F.II.pl	Pto.R.II.B	5/5
WAK.Ar.A.F	WAK.Ar.A.R	11/11	BRI1.F	BRI1.F	12/12
WAK.Ar.B.F	WAK.Ar.B.R	3/10	BRL1.F	BRL1.R	8/12
WAK.Ar.C.F	WAK.Ar.C.R	13/13	BRL2.FII	BRL2.RI	12/12
WAK.Vt.A..F	WAK.Vt.A.R	13/13	BRL2.FIII	BRL2.RII	18/18
WAK.Vt.B.F	WAK.Vt.B.R	13/13			

[a] Ratio of the number of RLK/Pelle gene fragments which were obtained out of the total number of sequenced PCR clones.
