# Supplementary material for: Kinase domain-targeted isolation of defense-related receptor-like kinases (RLK/Pelle) in Platanus × acerifolia: phylogenetic and structural analysis
Source: BMC Res Notes. 2014 Dec 8;7:884. doi: 10.1186/1756-0500-7-884 (PMC4295470; doi:10.1186/1756-0500-7-884)
Supplement: Supplementary file 3 — Additional file 3: Platanus × acerifolia ( Pac ) RLK/Pelles: NCBI GenBank accession numbers, subfamily classification and sequence text. Sequences in A and B were obtained in this work, those in C were used for comparison. In D nucletide and amino acidic sequences are listed. (PDF 319 KB) [file 13104_2014_3456_MOESM3_ESM.pdf]

Additional file 3. *Platanus* × *acerifolia* (*Pac*) RLK/Pelles: NCBI GenBank accession numbers, subfamily classification and sequence text. Sequences in A and B were obtained in this work, those in C were used for comparison. In D nucletide and amino acidic sequences are listed.

A

Continuous ORF	Acc. Nos.	RLK/Pelle subfamily
pac.x.5.1	EU722764	LRR XII-like
pac.x.5.2	EU722765	LRR XII-like
pac.x.5.3	EU722766	LRR XII-like
pac.x.5.4	EU722767	LRR XII-like
pac.x.5.6	EU722768	LRR XII-like
pac.x.5.8	EU722769	LRR XII-like
pac.x.5.9	EU722770	LRR XII-like
pac.x.5.12	EU722771	LRR XII-like
pac.x.5.13	EU722772	LRR XII-like
pac.x.5.14	EU722773	LRR XII-like
pac.x.5.17	EU722774	LRR XII-like
pac.x.5.18	EU722775	LRR XII-like
pac.x.5.19	EU722776	LRR XII-like
pac.x.5.26	EU722777	LRR XII-like
pac.x.5.39	EU722778	LRR VII-like
pac.x.5.43	EU722779	LRR VII-like
pac.x.6.102	EU722780	LRR XII-like
pac.x.6.103	EU722781	LRR XII-like
pac.x.6.104	EU722782	LRR XII-like
pac.x.6.107	EU722783	LRR XII-like
pac.x.6.108	EU722784	LRR XII-like
pac.x.6.109	EU722785	LRR XII-like
pac.x.6.112	EU722786	LRR XII-like
pac.x.6.115	EU722787	LRR XII-like
pac.x.6.123	EU722788	LRR XII-like
pac.x.6.124	EU722789	LRR XII-like
pac.x.6.129	EU722790	LRR XII-like
pac.x.7.4	EU722791	LRR XII-like
pac.x6.52	EU722792	L-Lectin-like
pac.x6.53	EU722793	L-Lectin-like
pac.x6.54	EU722794	L-Lectin-like
pac.x6.55	EU722795	L-Lectin-like
pac.pt.4.7	EU722796	CrRLK1L-like
pac.pt.4.8	EU722797	CrRLK1L-like
pac.pt.4.11	EU722798	CrRLK1L-like
pac.pt.4.13	EU722799	CrRLK1L-like
pac.pt.4.14	EU722800	CrRLK1L-like
pac.pt.5.102	EU722801	CrRLK1L-like

pac.pt.5.103	EU722802	CrRLK1L-like
pac.pt.5.104	EU722803	CrRLK1L-like
pac.pt.5.106	EU722804	CrRLK1L-like
pac.pt.5.107	EU722805	CrRLK1L-like
pac.pt.5.110	EU722806	CrRLK1L-like
pac.pt.5.113	EU722807	CrRLK1L-like
pac.pt.2.202	EU722808	CrRLK1L-like
pac.pt.2.203	EU722809	CrRLK1L-like
pac.pt.2.205	EU722810	CrRLK1L-like
pac.pt.2.207	EU722811	CrRLK1L-like
pac.pt.2.209	EU722812	CrRLK1L-like
pac.pt.2.210	EU722813	CrRLK1L-like
pac.pt.10.301	EU722814	CrRLK1L-like
pac.pt.10.306	EU722815	CrRLK1L-like
pac.pt.10.309	EU722816	CrRLK1L-like
pac.pt.10.310	EU722817	CrRLK1L-like
pac.ptd.5.2	EU722818	CrRLK1L-like
pac.ptd.5.5	EU722819	CrRLK1L-like
pac.ptd.8.21	EU722820	CrRLK1L-like
pac.ptd.8.22	EU722821	CrRLK1L-like
pac.ptd.8.23	EU722822	CrRLK1L-like
pac.ptd.9.41	EU722823	CrRLK1L-like
pac.Erf.2	EU722824	Uncertain
pac.Erf.3	EU722825	LRR XII-like
pac.Erf.4	EU722826	LRR XII-like
pac.Erf.5	EU722827	LRR XII-like
pac.Erf.6	EU722828	LRR XII-like
pac.Erf.7	EU722829	LRR XII-like
pac.Erf.9	EU722830	Uncertain
pac.Erf.10	EU722831	LRR XII-like
pac.Erf.11	EU722832	LRR XII-like
pac.Erf.13	EU722833	Uncertain
pac.Erf.14	EU722834	LRR XII-like
pac.Erf.2B.210	EU722835	SD-2-like
pac.Erf.2B.211	EU722836	SD-2-like
pac.W.ArA.3	EU722837	Wak-like-like
pac.W.ArA.4	EU722838	Uncertain
pac.W.ArA.5	EU722839	Uncertain
pac.W.ArA.6	EU722840	Wak-like-like
pac.W.ArA.7	EU722841	Wak-like-like
pac.W.ArA.8	EU722842	Wak-like-like
pac.W.ArA.9	EU722843	Wak-like-like
pac.W.ArA.11	EU722844	Wak-like-like
pac.W.ArA.12	EU722845	Uncertain
pac.W.ArA.14	EU722846	Wak-like-like
pac.W.ArB.305	EU722847	Wak-like-like
pac.W.ArB.312	EU722848	Wak-like-like

pac.W.VtA.101	EU722849	Wak-like-like
pac.W.VtA.102	EU722850	Wak-like-like
pac.W.VtA.105	EU722851	Wak-like-like
pac.W.VtA.107	EU722852	Wak-like-like
pac.W.VtA.108	EU722853	Wak-like-like
pac.W.VtA.109	EU722854	Wak-like-like
pac.W.VtA.110	EU722855	Wak-like-like
pac.W.VtA.113	EU722856	Wak-like-like
pac.W.VtB.201	EU722857	Wak-like-like
pac.W.VtB.203	EU722858	Wak-like-like
pac.W.VtB.208	EU722859	Wak-like-like
pac.W.VtB.209	EU722860	Wak-like-like
pac.W.Ch.152	EU722861	Wak-like-like
pac.W.Ch.156	EU722862	Wak-like-like
pac.W.Ch.157	EU722863	Wak-like-like
pac.W.Ch.158	EU722864	Wak-like-like
pac.W.Ch.160	EU722865	Wak-like-like
pac.W.Ch.162	EU722866	Wak-like-like
pac.W.Ch.164	EU722867	Wak-like-like
pac.BRI.L.2	EU722868	LRR X BRI group-like
pac.BRI.L.6	EU722869	LRR X BRI group-like
pac.BRL.B.8	EU722871	LRR X BRI group-like
pac.BRL.B.9	EU722872	LRR X BRI group-like
pac.BRL.B.12	EU722873	LRR X BRI group-like
pac.BRL.B.14	EU722874	LRR X BRI group-like
pac.BRL2.102	HQ425329	LRR X BRI group-like
pac.BRL2.8	HQ425330	LRR X BRI group-like

B

Interrupted ORF	Acc. Nos.
pac.x.5.5-St	EU722875
pac.x.5.7-St	EU722876
pac.x.5.11-St	EU722877
pac.x.5.27-St	EU722878
pac.x.6.105-St	EU722879
pac.x.6.111-st	EU722880
pac.x.6.130-St	EU722881
pac.Pt.4.9-St	EU722882
pac.Pt.5.105-St	EU722883
pac.Pt.10.308-St	EU722884
pac.Erf.8-st	EU722885
pac.Erf.2B.205-st	EU722886
pac.W.VtA.103-st	EU722887
pac.W.VtA.104-st	EU722888

pac.W.VtA.106-st	EU722889
pac.W.VtA.111-st	EU722890
pac.W.VtA.114-st	EU722891
pac.W.VtB.205-st	EU722892
pac.W.VtB.206-st	EU722893
pac.W.VtB.211-st	EU722894
pac.W.VtB.212-st	EU722895
pac.W.VtB.214-st	EU722896
pac.W.Ch.151-st	EU722897
pac.W.Ch.154-st	EU722898
pac.W.Ch.159-st	EU722899
pac.W.Ch.163-st	EU722900

C

Long ORF	Acc. Nos.	RLK/Pelle subfamily
Isotig1	KF208644	CrRLK1L
Isotig2	KF208645	CrRLK1L
Isotig3	KF208646	CrRLK1L
Isotig4	KF208647	LRR XII
Isotig5	KF208648	LRR XII
Isotig6	KF208649	LRR XII
Isotig7	KF208650	LRR XII
Isotig8	KF208651	Wak-like
Isotig9	KF208652	LRR X-BRI1
Isotig10	KF208653	LRR X-BRL

D

nt sequences

```
>pac.x.5.1
AGGGGTACTATCTGACCGGACTATTGGTGCTATAAAGGTTTTCAATTTGGAATTTCTAGC
AGCATTTAAGAGTTTTGATGCAGAATGTGAAGTAATGCGTAATATATGCCACCGGAACCT
TGTCAAAATCATAAGTAGTTGCTCCAATACTGAATTTAGAGCATTGATTCTTGAGTACAT
GCCCAATGGAAGCCTCGAGAAGTGGTTGTATTCTTACAACTATTATCTGGATTTCATGCA
AAGACTGGACATTATGTTAGATGTGGCATCTGCACTGGAATATCTTCATCACGATTATTC
AACAGCAGTGGTACATTGTGACTTGAAGCCAAGCAATGTCTTACTAGATGAAGACATGGT
TGGGCATGTATGTGATTTCGGCATCGCAAAGCTCTTGCCAGAAAATGAATCTACCAAACA
AACAAAGACATTAGGGACATTG
>pac.x.5.2
AGGAATTCTTCATCAAGATAAACTACCTGTTGCTGTGAAGGTACTCAATCTACAAGAACG
GGGGGCTTCGAAGAGTTTCATGGCTGAATGTGAGATCTTGAGAGAAATTCGGCATCGAAA
CCTTCTCAAGATATTAGCTTCTTGCTCAACTATTGACTTTCAAGGTAATGATTTTAAAGC
TCTTGTTTTTGAGCTCATGCCTAACGGGAGTCTTGAAAGGTGGTTGCACCCAAGTACAGA
TGGACAACATTTGTTGAATTATTTATGCTTGGGCAAGAGATTAGACATAGCAATTGATAT
CGCTTCTGCTTTGGATTATCTTCATCACCATAACCAAACTCCAATTGTTCACTGTGATCT
AAAGCCAAGCAACATTCTTCTTGATGATAGCATGGCCGCTCATATGGGTGATTTTGGGTT
AGCAAAGTTCCTTTCTAAAGACACCAACTTTTCTGATAAAAATGAAACTAGTTCAATAGC
CATAAAGGGTTCTATT
```

>pac.x.5.3
AGGGATTCTTCCTCGAGATGAGCTACCTGTTGCTGTAAAGGTACTCAACCTTCAAGAATG
CGGGGCTTCCAAGAGTTTCATGGCTGAATGTGAAGCCTTGAGAGCAATTCGGCACCGGAA
CCTCCTCAAGATCTTAACATCTTGCTCAGGTGTGGACTTTGAAGGTAACGATTTCAAAGC
TCTTGTTTTTGAGCTCATGCATAATGGGAGTCTTGAAAACTGGTTGCACCCAACTATAGA
TGGACGACATCTATTGAATAATTTGAGGTTTGGGCAGAGATTAAATATAGCAATTGATGT
CGCTTCTGCTTTGGATTATCTTCACCACCATTGCCAAACTCCAATTGTTCACCGCGATAT
AAAGCCAAGCAATGTCCTTCTTGATGATGACATGACTGCCCATGTTGGTGATTTTGGGTT
GGCAAAGTTCCTTTCTAATCCCACAAACAATTCCAGAAAATTTGAAAGTAGTTCGGTTGC
CTTAAGAGGATCTATT
>pac.x.5.4
AGGACTTCTAAATCAGGGTGAAACAATTGTTGCAGTGAAGGTACTCAACCTTCAACAATA
CGGATCCTATAAGAGCTTCATGGCTGAGTGCGAATCCTTGAGGAACATCCAGCATCGGAA
TCTCGTCAAGGTCATAACTTCCTGCTCAAGTATAGATTTTGAGGGCAATGATTTCAAAGC
TCTAGTTTACGAGTTCATGAGTAATGGAAGTTTAGAGAGGTGGTTACATCCAAATGCAGA
AGATGCACAAGTTGAACAGAGGAATTTAAATCTTCTTCAAAGGCTTAACATTGCCATTGA
TGTTGCCTGTGCACTGGATTATCTTCATCACAACTCTAAAACACCAATTGTTCATTGTGA
TCTAAAGCCAAGCAACGTTCTCCTTGATGACGACATGGTTGCTCATGTGGGTGATTTTGG
TTTATCAAGATTCCTTCCAATGACAATTAATAATTCCTCTCGAAGTCAAACCAGCTCTAT
GGGAATA
>pac.x.5.6
AGGAATGGTTCATCAGGATGGAAGACTAGTATCGGTTGCTGTGAAGGTATTGAACCTTCA
ACAACAAGAAGCATCCAAGACTTTTATGGCCGAATGCAAAGCATTGAGGAATGTCCGGCA
TCGAAATCTCCTCAAGATCATTACTTCTTGTTCGAGTGCGGATTTTAAGGGTAATGAGTT
TAAGGCTCTAGTTTTGGAGTTCATGCCTAATGGGAGTCTAGAGAGTTGGTTGCACCCAAG
GATAGATGGACAACTTCAATTGAGATACTTGAGCATTAGTCAAAGATTGAATATAGCAAT
TGATGTTGCTTTAGCATTGGATTATCTTCACAACCATTGTCAAATTCCTATTGCTCATTG
TGATCTAAAACCAAGCAATGTTCTTCTTAATGATGACATGACTGCCCATGTTGGTGACTT
TGGACTAGCCAAATTCCTTTATAGGGCTTCAAACAATTGTGGTGAAGGTCAACACAGTTC
AGCTGCACTTAAGGGAACTATT
>pac.x.5.8
AGGAATTCTTTGTCAAGATGAACCACCTGTTGCTGTGAAGGTACTCAACCTTCAAGAACA
GGGGGCTTCCAAGAGTTTCATGGCTGAATGTGAAGCTTTAAGAGAGATTCGACATCGAAA
CCTCCTTAAGATCTTAACTTGCTGCTCAAGTGTGGACTTTGAAGGTATTGATTTCAAAGC
TCTTGTTTATGAGCTCATGCCTAATGGGAGTCTTGAAAATTGGTTGCACCAAAGTACAAA
TGGACAACATCTGAATGATTTGAACTTCGGCCAGAGGTTAAACATAGCGATGGATGTCGC
TTTGGCTTTGGATTATCTTCATCACCACTGCCAAACCCCTATTGTTCACTGTGATTTAAA
GCCAAGCAATGTTCTTCTCGATGATAACATGGTGGCCCATCTCGGTGATTTTGGGTTAGC
AAAGATCCTTTCTATGACCTCCAACTATTCCAGTGAAAATGAAACTATTTCAATTGTCAT
AAAGGGATCTATT
>pac.x.5.9
AGGGATTCTTCCTCGAGATGAGCTACCTGTTGCTGTAAAGGTACTCAACCTTCAAGAATA
CGGGGCTTCCAAGAGTTTCATGGCTGAATGTGAAGCCTTGAGAGCAATTCGGCACCGGAA
CCTCCTCAAGATCTTAACATCTTGCTCAGGTGTGGACTTTGAAGGTAACGATTTCAAAGC
TCTTGTTTTTGAGCTCATGCATAATGGGAGTCTTGAAAACTGGTTGCACCCAACTATAGA
TGGACAACATCTATTGAATAATTTGAGGTTTGGGCAGAGATTAAATATAGCAATTGATGT
CGCTTCTGCTTTGGATTATCTTCACCACCATTGCCAAACTCCAATTGTTCACCGCGATCT
AAAGCCAAGCAATGTCCTTCTTGATGATGACATGACTGCCCATGTTGGTGATTTTGGGTT
GGCAAAGTTCCTTTCTAATCCCACAAACAATTCCAGAAAATTTGAAAGCAGTTCGGTTGC
CTTAAGAGGGTCTATT
>pac.x.5.12
AGGAATTCTTCGTCAGGATGAACAACCTATTGCTGTGAAGGTAGTCAACCTTCAAGAACA
AGGGGCTTCCAAGAGTTTTATGGCCGAATGTGAAGCTTTGAGAGAAATTCGACATCGAAA
TCTCCTTAAGATCTTAACTTCCTGCTCAAGTGTGGACTTTCAAGGTAATGATTTCAAAGC
TCTTGTTTTTGAGCTCATGCCTAATGGGAGTCTTGAAAACTGGTTGCATCCAAAACCAAA
TGAACAAAATCAATTGAATAAGTTGAATCTTAGACAGAGATTAAACATATCAATTGATGT
CGCTTCTGCTTTGGATTATCTTCATCATCAGTGCCAAACCCCTATTGTTCATTGTGATTT
AAAGCCAAGCAATGTTCTTCTTGATGATGACATGACTGCCCATGTTGGTGATTTTGGGTT
GGCAAAGTTCCTTTCTAATCCCACAAACAATTCCAGAAAATTTGAAAGCAGTTCGGTTGC
CTTAAGAGGGTCTATT
>pac.x.5.13
AGGTAATCTTCCTCGAGATGATCTACCTGTTGCTGTAAAGGTACTCAACCTTCAAGAATG
TGGGGCTTCCAAGAGTTTCATGGCTGAATGTGAAGCCTTGAGAGCAATTCGGCATCGGAA
CCTCGTCAAGATCTTAACATCTTGCTCAAGTGTGGACTTTGAAGGTAACGATTTCAAAGC
TCTTGTTTTTGAGCTCATGCATAATGGGAGTCTTGAAAACTGGTTGCACCCAACTTTAGA
TGGGCGACATCTATTGAATAATTTGAGGTTTGGGCAGAGATTAAATATAGCAATTGATGT
CGCTTCCGCTTTGGATTATCTTCACCACCATTGCCAAACTCCAATTGTTCACTGCGATCT
AAAACCAAGCAATGTTCTTCTTGATGATGACATGACTGCCCATGTTGGTGATTTTGGGTT
GGCAAAGTTCCTTTTTAATCCCACAAACAATTCCAGAAAAAATGAAAGTAGTTCGGTTGC
CTTAAGAGGATCTATT

>pac.x.5.14
AGGACTTCTAAATCAGGGTGAAACAAATGTTGCAGTGAAGGTATTCAACCTTCCACAACA
TGGAGCCTCTAAGAGTTTCATGGCTGAGTGTGAATCCTTGAGAAACATCCGGCATCGGAA
TCTCGTCAAGGTTATAACTTCCTGCTCGAGTATAGATTTTGCGGGCAATGATTTCAAAGC
TCTAGTTTACGAGTTTATGAGTAATGGAAGTTTAGAGAGGTGGTTATATCCAAATGCAGA
AGTGGCACAAGTTGAACAAAGGAATTTAAATATTCTTCAAAGACTTAACATTGCCATTGA
TGTTGCCTCTGCACTAGATTATCTTCATCACAACTGTAAAACACCAATCATTCATTGTGA
TCTAAAGCCAAGCAACATTCTCCTTGATGACGACATGGTTGCTCACGTGGGTGATTTTGG
TTTATCAAGATTCCTCCCAATGACGATTAATAATTCTCAAAGTCAAACCAGCTCTATTGG
AATAAAAGGATCCATT
>pac.x.5.17
AGGGATTCTTCATCAGAATGAACTACCTATTGCTGTGAAGGTATTCAATCTTCTGGAACA
TGGGGCTTCCAAGAGTTTCATGACCGAATGTGAAGCTTTGAGAGAGATTCGGCATCGAAA
CCTCCTCAAGATCTTAACTTCTTGCTCAAGTACCGACTTTGAAGGTAATGACTTCAAAGC
TCTTGTTTTTGTGCTCATGCCTAATGGGAGTCTTGAAAACTGGTTGCACCAAAGTACATA
TGGACGACATCAAAATTATTTGAACTTTGACCAGAGATTAAACATAGCAATTGACGTCGC
ATCTGCTTTGGACTATCTTCATCACCAGTGCCAAACCCCTATTGTTCACTCTGATCTAAA
GCCAAGCAATGTTCTTCTTGACGATGACATGGTTGCCCATGTCAGTGATTTTGGGTTAGC
AAAGTTCATTTCTAAGCCCACCAAATATTCTGGTAACAATAAAACTAGTTCAGTTGTCAT
AAAGGGATCTATT
>pac.x.5.18
AGGGATTCTTCCTCGAGATGATCTACCTGTTGCTGTAAAGGTACTCAACCTTCAAGAATG
TGGGGCTTCCAAGAGTTTCATGGCTGAATGTGAAGCCTTGAGAGCAATTCGGCATCGGAA
CCTCGTCAAGATCTTAACATCTTGCTCAAGTGTGGACTTTGAAGGTAACGATTTCAAAGC
TCTTGTTTTTGAGCTCATGCATAATGGGAGTCTTGAAAACTGGTTGCACCCAACTTTAGA
TGGGCAACATCTATTGAATAATTTGAGGTTTGGGCAGAGATTAAATATAGCAATTGATGT
CGCTTCTGCTTTGGATTATCTTCACCACCATTGCCAAACTCTAATTGTTCACTGTGACCT
AAAACCAAGCAATGTTCTTCTTGATGATGACATGACTGCCCATGTGGGTGATTTTGGGTT
AGCAAAATTCATTTCAAAGCCTACCAACTATTTTGGTAAAAATGACACTAGTTCATCAAT
TGCTATAAAAGGATCTATC
>pac.x.5.19
AGGGATTCTTCCTCGAGATGATCTACCTGTTGCTGTAAAGGTACTCAACCTTCAAGAATG
TGGGGCTTCCAAGAGTTTCATGGCTGAATGTGAAGCCTTGAGAGCAATTCGGCATCGGAA
CCTCGTCAAGATCTTAACATCTTGCTCAAGTGTGGACTTTGAAGGTAACGATTTCAAAGC
TCTTGTTTTTGAGCTCATGCATAATGGGAGTCTTGAAAACTGGTTGCACCCAACTTTAGA
TGGGCAACATCTATTGAATAATTTGAGGTTTGGGCAGAGATTAAATATAGCACTTGATGT
CGCTTCTGCTTTGGATTATCTTCAGTACCATTGCCAAACTCCAATTGTTCACTGCGATTT
AAAACCAAGCAATGTTCTTCTTGATGATGACATGACTGCCCATGTGGGTGATTTTGGGTT
AGCAAAGATCCTTTCTAAACCCACCAGCTACACTGGGAAAAATGAAACTAGTTCACTTGC
CATAAAGGGATCAATT
>pac.x.5.26
AGGACTTCTAAATCAGGGTGAAACAAATGTTGCAGTGAAGGTATTCAACCTTCCACAACA
TGGAGCCTCTAAGAGTTTCATGGCTGAGTGTGAATCCTTGAGAAACATCCGGCATCGGAA
TCTCGTCAAGGTTATAACTTCCTGCTCGAGTATAGATTTTGCGGGCAATGATTTCAAAGC
TCTAGTTTACGAGTTTATGAGTAATGGAAGTTTAGAGAGGTGGTTATATCCAAATGCAGA
AGTGGCACAAGTTGAACAAAGGAATTTAAATATTCTTCAAAGACTTAACATTGCCATTGA
TGTTGCCTCTGCACTAGATTATCTTCATCACAACTGTAAAACACCAATCATTCATTGTGA
TCTAAAGCCAAGCAACATTCTCCTTGATGACGACATGGTTGCTCACGTGGGTGATTTTGG
TTTATCAAGATTCCTCCCAATGACAATTAATAATTCTTCTCGAAGTCAAACCAGCTCTAT
TGGAATAAAAGGATCCATT
>pac.x.5.39
GGCTTCTCTAGGTGGAGAAAAGAGGATTTTGGCCATTAAGAAGCTTGTAACCTCTAACAT
AATCCAATATCCAGAAGATTTTGACAGAGAAGTTAGAATTCTAGGAAAAGCTATGCACCC
AAATCTGATGACACTCAAAGGGTACTACTGGACTCCTCAGTTGCAGCTTCTGATATCTGA
TTATGCTCCCAATGGAAGCTTGCACTCCAAACTCCATGAAAGGTCCCCGTCCACCCCCTTC
TCTTTCTTGGGCCAACCGATTCAAAATTGTGCTTGGAACAGCAAAAGGACTTGCTCATCT
GCATCAGTCTTTCCGTCCACCAATCATACACTACAATGTCAAACCAAGCAATATCTTGCT
TGATGAGAATTACAATCCAAAGATCTCCGACTTTGGACTGGCAAGGCTTCTTCCCAAGCT
CGACAAGCATGTGATAAATAGCAGGTTTCAGAGTGCCTTA
>pac.x.5.43
GGCTTCTCTAGGTGGAGAAAAGAGGATTTTGGCCATTAAGAAGCTTGTAACCTCTAACAT
AATCCAATATCCAGAAGATTTTGACAGAGAAGTTAGAATTCTAGGAAAAGCTATGCACCC
AAATCTGATGACGCTCAAAGGGTACTACTGGACTCCTCAGTTGCAGCTTCTGATATCTGA
TTATGCTCCCAATGGAAGCTTGCACTCCAAACTCCATGAAAGGTCCCCGTCCGCCCCCTTC
TCTTTCTTGGGCCAACCGATTCAAAATTGTGCTTGGAACAGCAAAAGGACTTGCTCATCT
GCATCAGTCTTTCCGTCCACCAATCATACACTACAATGTCAAACCAAGCAATATCTTGCT
TGATGAGAATTACAATCCAAAGATCTCCGACTTTGGACTGGCAAGGCTTCTTCCCAAGCT
CGACAAGCATGTGATAAATAGCAGGTTTCAGAGTGCCTTA
>pac.x.6.102
GTATAAAGGAATTCTTCATCAAGATAAACTACCTGTTGCTGTGAAGGTACTCAATCTACA

AGAACGGGGGGCTTCGAAGAGTTTCATGGCTGAATGTGAGATCTTGAGAGAAATTCGGCA
TCGAAACCTTCTCAAGATATTAGCTTCTTGCTCAACTATTGACTTTCAAGGTAATGATTT
TAAAGCTCTTGTTTTTGAGCTCATGCCTAACGGGAGTCTTGAAAGATGGTTGCACCCAAG
TACAGATGGACAACATTTGTTGAATTATTTATGCTTGGGCAAGAGATTAGACATGGCAAT
TGATATCGCTTCTGCTTTGGATTATCTTCATCACCATAACCAAACTCCAATTGTTCACTG
TGATCTAAAGCCAAGCAACATTCTTCTTGATGATAGCATGACCGCTCATATGGGTGATTT
TGGGTTAGCAAAGTTCCTTTCTAAAGACACCAACTTTTCTGATAAAAATGAAACTAGTTC
AATAGCCATAAAGGGTTCTATT
>pac.x.6.103
GTATAAAGGAATCCTTCATCGAGATGAATCTCCTATTGCTGTGAAGGTATTCAATCTTCT
GGAACATGGGGCTTCCAAGAGTTTCATGACCGAATGTGAAGCTTTGAGAGAGATTCGGCA
TCGAAACCTCCTCAAGATCTTAACTTCTTGCTCAAGTACCGACTTTGAAGGTAATGACTT
CAAAGCTCTTGTTTTTGTGCTCGTGCCTAATGGGAGTCTTGAAAACTGGTTGCACCAAAG
TACATATGGACGACATCAAAATTATTTGAACTTTGACCAGAGATTAAACATAGCAATTGA
CGTCGCATCTGCTTTGGACTATCTTCATCACCAGTGCCAAACCCCTATTGTTCACTCTGA
TCTAAAGCCAAGCAATGTTCTTCTTGACGATGGCATGGTTGCCCATGTCAGTGATTTTGG
GTTAGCAAAGTTCATTTCTAAGCCCACCAAATATTCTGGTAACAATAAAACTAGTTCAGT
TGTCATAAAGGGATCTATT
>pac.x.6.104
ATATAAAGGAATGGTTCATCAGGATGGAAGACTAGTATCGGTTGCTGTGAAGGTATTGAA
CCTTCAACAACAAGAAGCATCCAAGACTTTTATGGCCGAATGCAAAGCATTGAGGAATGT
CCGGCATCGAAATCTCCTCAAGATCATTACTTCTTGTTCGAGTGCGGATTTTAAGGGTAA
TGAGTTTAAGGCTCTAGTTTTGGAGTTCATGCCTAATGGGAGTCTAGAGAGTTGGTTGCA
CCCAAGGATAGATGGACAACTTCAATTGAGATACTTGAGCATTAGTCAAAGATTGAATAT
AGCAATTGATGTTGCTTTAGCATTGGATTATCTTCACAACCATTGTCAAATTCCTATTGC
TCATTGTGATCTAAAACCAAGCAATGTTCTTCTTAATGATGACATGACTGCCCATGTGGG
TGACTTTGGACTCGCCAAGTTCCTTTCTAGGGCTACCAGCAATTCTGGTGAAGGTCAACA
CAGCTCAGTTGCAATTAAGGGAACTATT
>pac.x.6.107
ATATAAAGGAATTATCCATTATGATGGAAAACCAGCACCGGTTGCTGTGAAGGTTTTGAA
CCTTCAACAAAGAGGAGCTTCCAAAACTTTCATGGTTGAATGCAATGCCTTGAGAAAAAT
CAGGCACCGAAATCTCCTCAAGATCATAACTTCTTGCTCCAGTGTGGATTTTAAGGGTAA
TGACTTTAAGGCTCTAGTTTTTGAATACATGCCTAGTGGAAGTCTGGAGAGCTGGTTGCA
CCCAAGTATAGATGAAAAACATCAATCAAGATATTTGAACTTTAGCCAAAGATTAGACAT
AGCAATTGATGCTGCTTTTGCATTGGATTATCTTAAAAACCATTGCCCAACACCGATCGT
TCATTGTGATATAAAACCAAGCAACGTTCTTCTTGATGATGACATGACCGCTCATGTGGG
TGACTTCGGACTAGCCAAGACCCTTTCTAGGGCTTCAAACAATTGTGGTGAAGGTCAACA
CAGTTCAGCTGCACTTAAGGGAACTATT
>pac.x.6.108
GTATAAAGGCATTCTTTGTCAAGATGAACAACCTGTTGCTATAAAGGTTCTTATCCTTCA
GGAAAGGGGGGCTTCCAAGAGTTTTATGGCCAAATGTGAAGCCTTAAGAGAAATTCAGCA
TCGAAACCTCCTCAAAATCTTCACTTCCTGCTCAACTGTGGACTTTGAAGGTAATTTTTT
CAGAGCTTTTGTTTTCGAGTTCCTGCCTAATGGGAGTCTTGAAAACTGGTTGCACCCAAG
TACCGATGGACAACATCTATTGAAGAATTTGAGCTTTAGCCAGAGATTAAACATAGCAAT
TGATGTTGCTTTTACTTTGGATTATCTACATCACCATTGCCAAACCCCAATTGTTCATTG
TGATCTAAAGCCAAGCAATATTCTTCTTGATGATGACATGACCACCCATGTGGGTGATTT
TGGGTTAGCAAAATTCCTATCTAAACCAACCAATTATTCCCGTAAGGATGAAACTAGTTC
AGTTGCCATAAAGGGATCTATC
>pac.x.6.109
TTATAAAGGAATTTTTCATCAAGATGGAAGAGCAATATTGATCGCTGTGAAGGTATTGAA
CCTTCTACAACGAAAAGCATCTAAGACCTTCATGGCCGAATGCAAAGCATTAAGGAACAT
CCGGCATCGAAATCTACTCAAGATCTTAACTTCTTGCTCAAGTATAGATTTTAAGGGTAA
TGATTTCAAGGCTCTAGTTTTCGAGTTCATGCCTAATAGGAGTCTAGAGAGTTGGTTACA
TCCAAGTGTAAACGAACACCATCAATTAAGGTGCCTGAATTTTAGCCAAAGACTAAACGT
AGCAATCGATGTTGCTTTTGCATTGGATTATCTTCAAAACCATTGCCCAACACCTATTGT
TCATTGTGATCTAAAACCAAGCAATGTTCTTTTTGATGATGACATGACTGCCCATGTGGG
TGACTTTGGACTAGCCAAGTTCCTTTCTATGGCTACCGACAATTCTGGTGAAAGTCAATA
CAGTTCAATTGCAATTAAGGGAACTATT
>pac.x.6.112
ATATAAAGGAATGGTTCATCAGGATGGAAGACTAGTATCGGTTGCTGTGAAGGTATTGAA
CCTTCAACAACAAGAAGCATCCAAGACTTTTATGGCCGAATGCAAAGCATTGAGGAATGT
CCGGCATCGAAATCTCCTCAAGATCATTACTTCTTGTTCGAGTGCGGATTTTAAGGGTAA
TGAGTTTAAGGCTCTAGTTTTGGAGTTCATGCCTAATGGGAGTCTAGAGAGTTGGTTGCA
CCCAAGGATAGATGGACAACTTCAATTGAGATACTTGAGCATTAGTCAAAGATTGAATAT
AGCAATTGATGTTGCTTTAGCATTGGATTATCTTCACAACCATTGTCAAATTCCTATTGC
TCATTGTGATCTAAAACCAAGCAATGTTCTTCTTAATGATGACATGACTGCCCATGTTGG
TGACTTTGGACTAGCCAAATTCCTTTATAGGGCTACCAGCAATTCTGCTGAGGGTCAAAA
CAGTTTGGCTACAATTAAAGGAACTATT
>pac.x.6.115
ATATAAAGGAATTCTTCATCAAGATGGAAGATCGATATTAGTTGCTGTGAAGGTATTGAA

CCTTCAACAACGAGATGGATTCAAGAGTTTCATGACCGAATGCAAAGCATTAAGGAATAT
CCGACATCGAAATCTGGTCAAGATCTTAACTTCCTGCTCGAGTATAGATTTTAAAGATAA
TGATTTCAAGGCTTTGGTTTTTGAGTTCATGCCTAATGGAAGTCTAGAGAGCTGGTTGCA
CCCAAGTACAGAAGGACACCATCAATCAAGATACTTGAATTTTAGCCAAAGACTGAATAT
AGCAATCGATGTTGCTTTTGCATTGGATTATCTTCATAACCATTGCTCAACACCTATTGT
TCATTGTGATCTAAAACCAAGCAATGTTCTTCTTGATGATGACATGACCGCCCATGTAGG
TGACTTTGGGCTTGCCAAGATCCTTTCTAGAGCTACTAGCAATTCTGGTGAAGGTCAACA
CAGTTCAGCTGCATTAAAGGGAACTATT
>pac.x.6.123
GTATAAAGGGATTCTTCATCAGGATGAACTATTTGTTGCTGTGAAGGAATTCAACCTTCA
AGAATGGGGGGCTTCCAAGAGTTTCATGGCCGAATGTGAAGCCTTGAGAGAGATCCGGCA
TCAAAACTTACTCAAGATCTTAACTGCTTGCTCAAGCATAGACATTGAAGGTAATGATTT
AAAAGCTCTTGTTTCCAAGCTCATGCCTAATGGGAGTCTTGAAAACTGGTTGCACCCAAA
TACATATGGAGGACATCTACTCAATAATTTGAGCTTTGGTAAGAGATTAAACATAGCAAT
TGACGTTGCTTCTGCTTTGGATTATCTTCATCACCATTGCCAAACTCCAATTGTTCACCG
TGACCTAAAACCAAGCAATGTTCTTCTTGATGATGACATGATTGCCCATGTGGGTGATTT
TGGGTTAGCAAAGTTCCTTTCGAAGCTCGACAACTATTCTGGTAAAAATGAAACTAGTTC
AATTGCTATAAAAGGATCCATC
>pac.x.6.124
GTATAAAGGAATTCTTCATCAAGATAAACTACCTGTTGCTGTGAAGGTACTCAATCTACA
AGAACGGGGGGCTTCGAAGAGTTTCATGGCTGAATGTGAGATCTTGAGAGAAATTCGGCA
TCGAAACCTTCTCAAGATATTAGCTTCTTGCTCAACTATTGACTTTCAAGGTAATGATTT
TAAAGCTCTTGTTTTTGAGCTCATGCCTAACGGGAGTCTTGAAAGGTGGTTGCACCCAAG
TACAGATGGACAACATTTGTTGAATTATTTATGCTTGGGCAAGAGATTAGACATAGCAAT
TGATATCGCTTCTGCTTTGGATTATCTTCATCACCATAACCAAACTCCAATTGTTCACTG
TGATCTAAAGCCAAGCAACATTCTTCTTGATGATAGCATGACCGCTCATATGGGTGATTT
TGGGTTAGCAAAGTTCCTTTCTAAAGACACCAACTTTTCTGATAAAAATGAAACTAGTTC
AATAGCCATAAAGGGTTCTATT
>pac.x.6.129
TTATAAAGGAATTTTTCATCAAGATGGAAGAGCAATATTGATCGCTGTGAAGGTATTGAA
CCTTCTACAACGAAAAGCATCTAAGACCTTCATGGCCGAATGCAAAGCATTAAGGAACAT
CCGGCATCGAAATCTACTCAAGATCTTAACTTCTTGCTCAAGTATAGATTTTAAGGGTAA
TGATTTCAAGGCTCTAGTTTTCGAGTTCATGCCTAATGGGAGTCTAGAGAGTTGGTTACA
TCCAAGTGTAAACGAACACCATCAATTAAGGTGCCTGAATTTTAGCCAAAGACTAAACGT
AGCAATCGATGTTGCTTTTGCATTGGATTATCTTCAAAACCATTGCCCAACACCTATTGT
TCATTGTGATCTAAAACCAAGCAATGTTCTTTTTGATGATGACATGACTGCCCATGTGGG
TGACTTTGGACTAGCCAAGTTCCTTTCTATGGCTACCGACAATTCTGGTGAAAGTCAATA
CAGTTCAATTGCAATTAAGGGAACTATT
>pac.x.7.4
AGGGCTATTGTCTGACGGGACTGTTTCTGCCGTAAAGGTTTTCAACTCGGAATTCCAAGG
AGTATTTAAGAGTTTTAATGTGGAATGTGAAGTATACCGCAATATTCGCCACCGAAACCT
TATCAAAATCATAAGTAGTTGCTCCAATATTGAATTTAGAGCATTGATTCTCGAGTACAT
GCCTAATGGAAGCCTTGAAAAGTGGTTGTATTCTCAGAATTATTGTTTAAATGTCTTGCA
AAGACTGGACATTATGATAGATGTTGCATTTGCATTGGAATATCTCCATCACGGTTATTC
AACATTAGTGGTGCATTGTGACTTAAAGCCAACAAACATCTTATTAGATGAAGACATGGT
TGGGCATGTAGGTGACTTTGGCATCGCAAAGCTCTTCGCAGAAAATGCATCTATCGTACA
AACCGAGACTCTAGGGACACTG
>pac.x6.52
ATAAAGGAACTCTATCGAATAACACAGAGGTCGCAGTCAAATGCGTAAGCCATGATTCGG
AGCAAGGGCTAAGAGAATTCATGGCGGAGATCTCAAGCATGGGCAGACTCCAACACAAGA
ACTTAGTCCACATGAAGGGTTGGTGCAGGAAAGGAAACGAGCTGATGCTGGTCTACAATT
ACATGTCCAACGGCAGCCTCAACCGTTGGATCTTTGATGAGCCCAAGAAGCTCTTGGGGT
GGAAGGAACGACGCAGAGTACTAGTTGATGTAGCTGAGGGCTTGAATTATCTGCACCACG
GATGGGATCAGGTGGTCCTTCACCGTGACATAAAGTCCAGCAACATATTATTGGATTCCG
AGTTCCGTGGTCGGTTAGGAGACTTTGGGCTTGCCAAGTTGTACCAACACGGGAAGGAAC
CGGGTACGACCCGACTGGTGGGTACGCTTGGGTACCTGGCGCCAGAGTTGGTGAATGTTG
GAGCTACGGCGGCG
>pac.x6.53
ATAAAGGAACTCTATCGAATAACACAGAGGTCGCAGTCAAATGCGTAAGCCATGATTCGG
AGCAAGGGCTAAGAGAATTCATGGCGGAGATCTCAAGCATGGGCAGACTCCAACACAAGA
ACTTAGTCCACATGAAAGGTTGGTGCAGGAAAGGAAACGAGCTGATGCTGGTCTACAATT
ACATGTCCAACGGCAGCCTCAACCGTTGGATCTTTGATGAGCCCAAGAAGCTCTTGGGGT
GGAAGGAACGACGCAGAGTACTAGTTGATGTAGCTGAGGGCTTGGATTATCTGCACCACG
GATGGGATCAGGTGGTCCTTCACCGTGACATAAAGTCCAGCAACATATTATTGGATTCCG
AGTTCCGTGGTCGGTTAGGAGGCTTTGGGCTTGCCAAGTTGTACCAACACGGGAAGGAAC
CGGGTACGACCCGACTGGTGGGTACGCTTGGGTACCTGGCGCCAGAGTTGGTGAATGTTG
GAGCTACGGCGGCG
>pac.x6.54
ATAAAGGAACTCTATCGAATAACACAGAGGTCGCAGTCAAATGCGTAAGCCATGATTCGG
AGCAAGGGCTAAGAGAATTCATGGCGGAGATCTCAAGCATGGGCAGGCTCCAACACAAGA

ACTTAGTCCACATGAAAGGTTGGTGCAGGAAAGGAAACGAGCTGATGCTGGTCTACGATT
ACATGTCCAACGGCAGCCTCAACCGTTGGATCTTTGATGAGCCCAAGAAGCTCTTGGGGT
GGAAGGAACGACGCAGAGTACTAGTTGATGTAGCTGAGGGCTTGAATTATCTGCACCACG
GATGGGATCAGGTGGTCCTTCACCGTGACATAAAGTCCAGCAACATATTATTGGATTCCG
AGTTCCGTGGTCGGTTAGGAGACTTTGGGCTTGCCAAGTTGTACCAACACGGGAAGGAAC
CGGGTACGACCCGACTGGTGGGTACGCTTGGGTACCTGGCGCCAGAGTTGGTGAATGTTG
GAGCTACGGCGGCG
>pac.x6.55
ATAAAGGAACTCTATCGAATAACACAGAGGTCGCAGTCAAATGCGTAAGCCATGATTCGG
AGCAAGGGCTAAGAGAATTCATGGCGGAGATCTCAAGCATGGGCAGGCTCCAACACAAGA
ACTTAGTCCACATGAAAGGTTGGTGCAGGAAAGGAAACGAGCTGATGCTGGTCTACGATT
ACATGTCCAACGGCAGCCTCAACCGTTGGATCTTTGATGAGCCCAAGAAGCTCTTGGGGT
GGAAGGAACGACGCAGAGTACTAGTTGATGTAGCTGAGGGCTTGAATTATCTGCACCACG
GATGGGATCAGGTGGTCCTTCACCGTGACATAAAGTCCAGCAACATATTATTGGATTCCG
AGTTCCGTGGTCGGTTAGGAGACTTTGGGCTTGCCAAGTTGTACCAACACGGGAAGGAAC
CGGGTACGACCCGACTGGTGGGTACGCTTGGGTACCTGGCGCCGGAGTTGGTGAACGTTG
GAGCTACGGCGGCG
>pac.pt.4.7
GTGCTCAAGAACAAAATGACAGTCGCTGTGAAACGAAGTGACCCGGGGGGAGATCAGGGC
CTTCTTGAATTCCAGAGAGAGATCATCATCTTATCCAGCATTCGCCATCGCCATCTAGTG
TCACTAATTGGGTATTGTGACGAGCGGTCAGAGATGATACTTGTTTATGAATTCATGGAG
AAGGGTACACTGAGAAGTCATCTTTACAATTCGGATTTGCCTAGTTTGTCATGGAAGCAG
AGACTTGATATATGCATTGGTTCCGCAAGAGGTCTTCATTACCTTCACACTGGCGTAGCA
GGAGGGATCATTCACCGTGATATCAAGTCGACTAACATCTTGCTTGATGAGAATTATGTT
GCTAAGGTTGCAGATTTCGGTCTTTCAAGGGAAGTGCCTGTTGAGCATAGCCGTGGCGGC
TATGTAAGCACAGCAGTAAAAGGCACCTTCGGGTATCTTGATCCAGAGTATTTTAAACGG
CAACAACTTACTGAGAAGTCC
>pac.pt.4.8
GGTGCTTAGAGACAATACAAAAATTGCAGTGAAGCGATGTGTACCGGGCTCTAAGCAGGG
CCTACCGGAATTCCAGACAGAGATCACGGTCTTGTCGAAGATTCGCCACCGACATCTTGT
TGCCCTTGTTGGGTACTGTGAAGAACAATCGGAGATGATACTGGTCTATGAGTACATGGA
GAAAGGACCATTAAGAAATCATCTATATGGTTCAGAGTTGCCACCTTTGTCTTGGAAGCA
GAGGCTTGAAATATGCATAGGTTCAGCCAGGGGTCTTCACTACCTTCATACAGGTTCAGC
CCAGGGAATCATCCATCGTGATGTTAAATCAACCAACATTTTGCTTGATGAGACTTATGT
GGCCAAGGTTGCTGATTTCGGTCTGTCGAGGTCTGGCCCATGCATGGATGAGACTCATGT
TAGCACTGGTGTGAAGGGCAGTTTTGGTTATCTGGACCCAGAGTATTTTAAACGGCAACA
GCTGACAGATAAATCG
>pac.pt.4.11
GACACTTGAAGATGGGACCAAGGTGGCTGTCAAGAGAGGAAACCCTAGATCTGAACAAGG
TCTTGCCGAGTTCCGGACTGAAATTGAGATGTTATCCAAGCTCCGCCACCGTCACCTTGT
CTCTCTGATTGGATACTGTGATGAAAGATCAGAGATGATCCTGGTTTATGAGTACATGGC
AAATGGGACCCTACGGAGCCATCTTTATGGGACAGACCTCCCATCTCTCTCATGGAGGCA
AAGACTAGAGATTTGCATTGGAGTTGCTAGGGGGGTTGCATTATCTCCACACGGAAGCAGC
TCAAAGCATAATTCACCGAGATGTGAAGTCCGCCAACATTCTCCTAAATGAGAACTTTGT
AGCAAAGGTTGCTGACTTTGGCATTTCAAAGATAGTTCCTGCTCTGGACCAGACCCATGT
GAGCACTGTTGTTAAGGGTACCTTTGGATACCTGGACCCTGAGTACTTTAGGAGGCAACA
ACTTACTGAGAAGTCC
>pac.pt.4.13
ACACTGGAAGACGGGACCAAGGTGGCTGTCAAGAGAGGGAACCCAAGATCTGGACAAGGT
CTTACTGAGTTCCAGACTGAAATTGAGTTGTTATCTAAGCTCCGCCATCGCCACCTAGTC
TCTCTGATTGGATACTGTAACGAAGGATCAGAGATGATCCTGGTTTATGAGTACATGGCA
AATGGGACCCTACGGAGACATCTTTGTGGGGCAGACCTTCCATCTCTCTCATGGAGGCAA
AGACTAGAGATTTGCATTGGAGCTGCTAAGGGGTTGCATTATCTCCACACGGGAGCAGCT
CAAAGCATAATTCACCGCGATGTGAAGTCTGCCAACATTCTCCTAAATGAGAACTTTGTA
GCAAAGGTTGCTGACTTTGGCATTTCAAAGATAGTTCCTGCTCTGGACCAGACCCATGTT
AGCACTGTTGTTAAGGGTAGCTTTGGATACCTGGACCCTGAGTACTTTAGGAGGAAACAA
CTTACTGAGAAGTCC
>pac.pt.4.14
GGTGCTTAGAGACAATACAAAAATTGCAGTGAAGCGATGTGTACCGGGCTCTGAGCAGGG
CGTACCAGAATTCCAGACAGAGATCACGGTCTTGTCGAAGATTCGCCACCAACATCTTGT
TGCCCTTATTGGGTACTGTGAAAAACAGTCGGAGATGATACTGGTCTATGAGTACATGGA
GAAAGGACCATTAAGAAATATTCTATATGGTTCAGAGTTGCCACCTTTGTCTTGGAAGCA
GAGTCTTGAAATATGCATAGGTTCAGCCAGGGGTCTTCACTACCTTCACACAGGTTCAGC
CCAGGGAATCATCCATCGTGACGTTAAATCAACCAACATTTTGCTTGATGAGAATTATGT
GACCAAGGTTGCTGATTTCGGCCTGTCGAGGTCTGGCCCCTGCGTGGACGAGACTCATGT
TAGCACTGGTGTGAAGGGCAGTTTTGGTTATCTGGACCCAGAGTATTTTAAACGGCAACA
GCTGACAGATAAATCG
>pac.pt.5.102
GGTGCTCAAGAACAAAATGACAGTCGCTGTGAAACGAAGTGACCCGGGGGGAGATCAGGG
CCTTCTTGAATTCCAGAGAGAGATCATCATCTTATCCAGCATTCGCCATCGCCATCTAGT

GTCACTAATTGGGTATTGTGACGAGCGGTCAGAGATGATACTTGTTTATGAATTCATGGA
GAAGGGTACACTGAGAAGTCATCTTTACAATTCGGATTTGCCTAGTTTGTCATGGAAGCA
GAGACTTGATATATGCATTGGTTCCGCAAGAGGTCTTCATTACCTTCACACTGGCGTAGC
AGGAGGGATCATTCACCGTGATATCAAGTCGACTAACATCTTGCTTGATGAGAATTATGT
TGCTAAGGTTGCAGATTTCGGTCTTTCAAGGGAAGTGCCTGTTGAGCATAGCCGTGGCGG
CTATGTAAGCACAGCCGTAAAAGGCACCTTCGGGTATCTTGATCCAGAGTATTTTAGACA
TCAACGGTTGACAGAGAAGTCAGATGTGTACTCCTTTGGTGTGGTGCTTCTT
>pac.pt.5.103
AGTCCTGGGAGATGGTACAAAGGTTGCAGTAAAGAGGGGTAATCCCCTATCCCAACAGGG
TCTTGCAGAGTTTCGGACTGAAATCGAAATGTTGTCTCAATTCCGCCACCGCCATCTGGT
CTCACTGATTGGGTATTGTGATGAGCGGAAAGAAATGATCCTAGTTTATGAGTACATGGA
AAATGGGACCCTTAAGAGTCATTTGTATGGCTCAAATCTTCCCAGCTTGAGTTGGAAGCA
GAGGCTCGAGATATGCATTGGATCAGCCAGAGGACTTCACTACCTTCATACTGGTTCTAC
AAAGGCAATTATCCACCGTGATGTTAAGTCTGCCAACATCTTACTTGATGAGAATCTCAT
GGCCAAGGTTGCTGATTTTGGGCTTTCCAAGACAGGTCCTGAGATCGATCAGACCCATGT
CAGCACCGCAGTCAAAGGGAGTTTTGGGTATCTCGATCCGGAGTATTTCAGAAGGCAGCA
ACTGACTGAGAAGTCAGATGTCTACTCATTCGGAGTGGTTTTGTTC
>pac.pt.5.104
TGAAATTGATGATGGCACCCTGGTGGCAATCAAGCGGGCCCATCCGCAATCTCAACAAGG
CCTAAGAGAATTTGAGACAGAGATTGAGATGCTGTCGAAGCTCAGGCATAGGCATCTTGT
CTCCATGATTGGATTCTGCGAGGAACAGAATGAGATGATCTTGGTGTATGAGTATATGGC
AAATGGAACCCTTAGGAGTCATCTCTTTGGGAGTGATCTCCCACCCTTAACATGGAAGCA
GCGGTTAGACGTGTGCATAGGCGCTGCCCGTGGACTCCATTACCTGCACACGGGTGCAGA
GAGGGGTATAATCCATAGGGATGTTAAGACAACCAACATACTTCTGGATGAGAATTTTGT
GGCAAAGATGGCTGATTTTGGGTTGTCAAAAACAGGTCCTGCATTGGAGCATACCCATGT
TAGTACTGCAGTAAAGGGAAGCTTTGGATATCTTGATCCAGAATATTTCCGAAAGCAACA
TTTGACTGAGAAGTCTGATGTTTATTCTTTTGGTGTAGTACTGTTT
>pac.pt.5.106
GTGCTCAAGAACAAAATGACAGTCGCTGTGAAACGAAGTGACCCGGGGGGAGATCAGGGC
CTTCTTGAATTCCAGAGAGAGATCATCATCTTATCCAGCATTCGCCATCGCCATCTAGTG
TCACTAATTGGGTATTGTGACGAGCGGTCAGAGATGATACTTGTTTATGAATTCATGGAG
AAGGGTACACTGAGAAGTCATCTTTACAATTCGGATTTGCCTAGTTTGTCATGGAAGCAG
AGACTTGATATATGCATTGGTTCCGCAAGAGGTCTTCATTACCTTCACACTGGCGTAGCA
GGAGGGATCATTCACCGTGATATCAAGTCGACTAACATCTTGCTTGATGAGAATTATGTT
GCTAAGGTTGCAGATTTCGGTCTTTCAAGGGAAGTGCCTGTTGAGCATAGCCGTGGCGGC
TATGTAAGCACAGCAGTAAAAGGCACCTTCGGGTATCTTGATCCAGAGTATTTTAGACAT
CAACGGTTGACAGAGAAGTCAGATGTGTACTCCTTTGGTGTGGTGCTTCTT
>pac.pt.5.107
GAGTTATGGAAGATGGTACAAAGGTTGCAGTAAAGAGGAGTAATCCCCTGTCCCAACAGG
GTCTTGCAGAGTTTCGGACTGAAATCGAAATGTTGTCTCAATTCCGCCACCGTCATCTGG
TCTCACTGATTGGGTATTGTGATGAGCAGAAAGAGATGATCCTAGTTTATGAGTACATGG
AGAATGGGACACTCAATAGTCATTTGTATGGCTCCAATTTTCCCAGCTTGAGTTGGAAGC
AGAGGCTAGAAATATGCATTGGATCAGCCAGAGGACTTCACTACCTTCACACTGGTTTTC
CGAAGGATATTATCCACCGTGATGTTAAGTCTGCCAACATCTTACTTGATGAGAATCTCA
TGGCCAAGGTTGCTGATTTTGGGCTTTCCAAGACAGGTCCTGGGATCGATCAGACCCATG
TCAGCACGGCGGTTAAGGGGAGTTTTGGATATCTCGATCCGGAATATTTCATAATGCAAC
GACTGACCGAGAAATCGGATGTCTACTCATTTGGGGTGCTTTTGTTT
>pac.pt.5.110
AAACGAAGTGAACCGGGACGAGATCAGGGCCTTCTCGAATTCCAGAGAGAAATCATCATT
TTATCCAACATTCGCCATCGCCATCTTGTGTCACTAATTGGGTTTTGTGACGAGCAATCG
GAGATGATACTCGTTTGTGAATTCATGGAAAAGGGAACACTGAGGAGTCATCTTTACAAT
TCGGATTTGCCTAGTTTGTCATGGAAGCAGAGACTTGATATATGCATTGGTTCCGCAAGA
GGTCTTCATTACCTTCACACTGGCGTAGCAGGAGGGATCATTCACCGTGATATCAAGTCG
ACTAACATCTTGCTTGATGAGAATTATGTTGCTAAGGTTGCAGATTTCGGTCTTTCAAGG
GAAGTGCCTGTTGAGCATAGCCGTGGCGGCTATGTAAGCACAGCAGTAAAAGGCACCTTC
GGGTATCTTGATCCAGAGTATTTTAGACATCAACGGTTGACAGAGAAGTCAGATGTGTAC
TCCTTTGGTGTGGTGCTTCTT
>pac.pt.5.113
GGTGCTCAAGAACAAAATGACAGTCGCTGTGAAACGAAGTGACCCGGGGGGAGATCAGGG
CCTTCTTGAATTCCAGAGAGAGATCATCATCTTATCCAGCATTCGCCATCGCCATCTAGT
GTCACTAATTGGGTATTGTGACGAGCGGTCAGAGATGATACTTGTTTATGAATTCATGGA
AAAGGGAACACTGAGAAGTCATCTTTACAATTCGGATTTGCCTAGTTTGTCATGGAAGCA
GAGACTTGATATATGCATTGGTTCCGCAAGAGGTCTTCATTACCTTCACACTGGCGTAGC
AGGAGGGATCATTCACCGTGATATCAAGTCGACTAACATCTTGCTTGATGAGAATTATGT
TGCTAAGGTTGCAGATTTCGGTCTTTCAAGGGAAGTGCCTGTTGAGCATAGCCGTGGCGG
CTATGTAAGCACAGCAGTAAAAGGCACCTTCGGGTATCTTGATCCAGAGTATTTTAGACA
TCAACGGTTGACAGAGAAGTCAGATGTGTACTCCTTTGGTGTGGTGCTTCTT
>pac.pt.2.202
TCAAGGGTGAAATTGATGATGGCACCCTGGTGGCAATCAAGCGGGCCCATCCGCAATCTC
AACAAGGCCTAAGAGAATTTGAGACAGAGATTGAGATGCTGTCGAAGCTCAGGCATAGGC

ATCTTGTCTCCATGATTGGATTCTGCGAGGAACAGAATGAGATGATCTTGGTGTATGAGT
ATATGGCAAATGGAACCCTTAGGAGTCATCTCTTTGGGGGTGATCTCCCACCATTAACAT
GGAAGCGACGATTAGATGTATGCATCGGTGCAGCACGTGGACTCCACTACCTCCACACAG
GGGCAGAGAGGAGTACAATCCATAGGGATGTTAAGACAACCAACATACTATTGGATGAGA
ACTTTGTTGCAAAGATGGCTGATTTCGGGTTGTCAAAAACCGGCCCTTCATTGGATCATA
CCCATGTTAGTACTGCAGTAAAGGGGAGCTTTGGATATCTTGATCCTGAATACTTCCGAA
GGCAACAATTGACTGAGAAATCTGATGTTTATTCTTTTGGTGTAGTACTGTTT
>pac.pt.2.203
ACAAGGGAGTTCTGAGAGATGGTACGAAGGTGGCAGTGAAAAGGGGTAATCCACTGTCCC
AACAGGGCTTTGCAGAGTTCTGGACTGAAATTGAAATGTTATCTCAGCTTCGCCACCGCC
ATCTGGTCTCAATGATCGGTTACTGTGATGAGCAAAAAGAGATGATCCTTGTTTATGAGT
ACATGGAAAATGGCACCCTCAGGAATCATCTTTATGGCTCAGAACTTCCCATCTTGAGTT
GGAAGCAGAGGCTTGAGATATGCATTGAATCGGCCAGAGGACTTCATTACCTACATACAG
GTTCCACAAAGGCAATCATTCACCGTGATGTTAAGTCGGCCAACATCTTACTTGATGAGA
ATCTCATGGCCAAGGTTGCTGATTTTGGGCTTTCAAAGGCAGGTCCCGAGATCGACCAGA
CCCATGTCAGCACTGCAGTTAAAGGGAGTTTTGGGTACCTTGATCCAGAGTATTTCAGAA
GGCAACAGCTGACTGAGAAATCAGATGTGTACTCATTCGGGGTAGTTTTGTTT
>pac.pt.2.205
TCAAGGGTGAAATTGATGATGGCACCCTGGTGGCAATCAAGCGGGCCCATCCGCAATCTC
AACAAGGCCTAAGAGAATTTGAGACAGAGATTGAGATGCTGTCGAAGCTCAGGCATAGGC
ATCTTGTCTCCATGATTGGATTCTGCGAGGAACAGAATGAGATGATCTTGGTGTATGAGT
ATATGGCAAATGGAACCCTTAGGAGTCATCTCTTTGGGAATGATCTCCCACCCTTAACAT
GGAAGCAGCGGTTAGACGTGTGCATAGGCGCTGCCCGTGGACTCCATTACCTGCACACGG
GTGCAGAGAGGGGTATAATCCATAGGGATGTTAAGACAACCAACATACTTCTGGATGAGA
ATTTTGTGGCAAAGATGGCTGATTCTGGGTTGTCAAAAACAGGTCCTGCATTGGAGCATA
CCCATGTTAGTACTGCAGTAAAGGGAAGCTTTGGATATCTTGATCCAGAATATTTCCGAA
AGCAACATTTGACTGAGAAGTCTGATGTTTATTCTTTTGGTGTAGTACTGTTT
>pac.pt.2.207
ATAGGGGTGAAATTGATGATGGCACCCTAGCGGCAATCAAGCGGGCACATCCACAATCTG
AACAAGGGCTGGCAGAATTTGAGACAGAGATTGAGATGCTTTCGAAGCTGAGGCATAGGC
ACCTGGTCTCCATGATTGGATTCTGTGAGGAGCAGAATGAGATGATCTTGGTGTATGAGT
ACATGGCAAATGGGACCCTTAGGAGTCATCTATTTGGGGGTGATCTCCCACCATTAACAT
GGAAGCGACGATTAGATGTATGCATCGGTGCAGCACGTGGACTCCACTACCTCCACACAG
GGGCAGAGAGGAGTACAATCCATAGGGATGTTAAGACAACCAACATACTTCTGGATGAGA
ATTTTGTGGCAAAGATGGCTGATTTTGGGTTGTCAAAAACAGGTCCTGCATTGGAGCATA
CCCATGTTAGTACTGCAGTAAAGGGAAGCTTTGGATATCTTGATCCAGAATATTTCCGAA
AGCAACATTTGACTGAGAAGTCTGATGTTTATTCTTTTGGTGTAGTACTGTTT
>pac.pt.2.209
ACAAGGGAGTTATGGAAGATGGTACAAAGGTTGCAGTAAAGAGGAGTAATCCCCTGTCCC
AACAGGGTCTTGCAGAGTTTCGGACTGAAATCGAAATGTTGTCTCAATTCCGCCACCGTC
ATCTGGTCTCACTGATTGGGTATTGTGATGAGCAGAAAGAGATGATCCTAGTTTATGAGT
ACATGGAGAATGGGACACTCAATAGTCATTTGTATGGCTCCAATTTTCCCAGCTTGAGTT
GGAAGCAGAGGCTAGAAATATGCATTGGATCAGCCAGAGGACTTCACTACCTTCACACTG
GTTTTCCGAAGGATATTATCCACCGTGATGTTAAGTCTGCCAACATCTTACTTGATGAGA
ATCTCATGGCCAAGGTTGCTGATTTTGGGCTTTCCAAGACAGGTCCTGGGATCGATCAGA
CCCATGTCAGCACGGCGGTTAAGGGGAGTTTTGGATATCCCGATCCGGAATATTTCATAA
TGCAACGACTGACCGAGAAATCGGATGTCTACTCATTTGGGGTGCTTTTGTTG
>pac.pt.2.210
ATAGGGGTGAAATTGATGATGGCACCCTAGCGGCAATCAAGCGGGCACATCCACAATCTG
AACAAGGGCTGGCAGAATTTGAGACAGAGATTGAGATGCTTTCGAAGCTGAGGCATAGGC
ACCTAGTCTCCATGATTGGATTCTGTGAGGAGCAGAATGAGATGATCTTGGTGTATGAGT
ACATGGCAAATGGGACCCTTAGGAGTCATCTATTTGGGGGTGATCTCCCACCATTAACAT
GGAGGCGACGATTAGATGTATGCATCGGTGCAGCACGTGGACTCCACTACCTCCACACAG
GGGCAGAGAGGAGTACAATCCATAGGGATGTTAAGACAACCAACATACTCTTGGATGAGA
ACTTTGTTGCAAAGATGGCTGATTTCGGGTTGTCAAAAACCGGCCCTTCATTGGATCATA
CCCATGTTAGTACTGCAGTAAAGGGGAGCTTTGGATATCTTGATCCTGAATACTTCCGAA
GGCAACAATTGACTGAGAAATCTGATGTTTATTCTTTTGGTGTAGTACTGTTT
>pac.Pt.10.301
TCTTGGGGAATTGGAAGATGGGACTAAGCTCGCCATCAAGCGTGGAAATCCTAGATCCGA
GCAAGGAATTAACGAGTTCCAAACAGAAATTCAAATGCTCTCCAAGCTTAGACATCGCCA
CTTAGTGTCCCTAATTGGGTACTGTGACGAACAATCAGAGATGATCCTTGTCTATGAATA
TATGGCAAATGGTCCTCTCCGTGACCACATTTATGGTTCTACCCTCCCTACATTGTCATG
GAAACAACGACTAGAGATCTGCATTGGTGCTGCCCGAGGCCTGCATTATCTTCATACAGG
CGCTGCGCAGGGGATTATCCACAGAGACGTAAAAACCACAAACATTCTTCTTGATGAGAA
CTTGGTTGCTAAGGTCTCGGATTTTGGTCTGTCAAAGACTGCACCCACATTGGAGCAGAC
CCATGTTAGTACAGCCGTGAAAGGTAGTTTTGGATATTTGGATCCTGAATACTTTAGAAG
GCAGCAGCTGACAGAGAAGTCTGATGTGTACTCGTTTGGGGTTGTTCTTTTC
>pac.pt.10.306
CAAGGGCGAGATCGATGGTGCAAGCACTAAGGTTGCGATCAAGCGTGGAAACCCACTCTC
CGAGCAGGGGGTCCATGAATTCCAAACGGAGATTGAGATGCTCTCAAAGCTTAGACACCG

TCATTTGGTCTCACTGATTGGGTACTGTGAAGAGAACTGTGAAATGATTCTTGTTTACGA
TTACATGGCCCATGGGACTCTTCGCGAGCACCTCTACAAGACCCAAAAGCCTCCTCTCCC
ATGGAGACAGAGGCTTGAGATTTGCATTGGAGCTGCCAGGGGCCTACACTACCTCCACAC
TGGTGCCAAACACACTATCATCCATCGAGATGTTAAGACAACAAACATTCTTCTGGATGA
GAAGTGGGTAGCAAAAGTCTCAGATTTTGGATTGTCGAAGACAGGCCCAACATTGGATCA
TACGCATGTAAGTACTGTTGTGAAGGGTAGTTTTGGGTACTTGGATCCGGAGTATTTTAG
GCGGCAGCAATTGACAGACAAATCAGATGTGTACTCATTCGGAGTTGTTTTGTTT
>pac.pt.10.309
TCTTGGGGAATTGGAAGATGGGACTAAGCTCGCCATCAAGCGTGGAAATCCTAGATCCGA
GCAAGGAATTAACGAGTTCCAAACAGAAATTCAAATGCTCTCCAAGCTTAGACATCGCCA
CTTAGTGTCCCTAATTGGGTACTGTGACGAACAATCAGAGATGATCCTTGTCTATGAATA
TATGGCAAATGGTCCTCTCCGTGACCACATTTATGGTTCTACCCTCCCTACATTGTCATG
GAAACAACGACTAGAGATCTGCATTGGTGCTGCGCGAGGCCTGCATTATCTTCATACAGG
CGCTGCGCAGGGGATTATCCACAGAGACGTAAAAACCACAAACATTCTTCTTGATGAGAA
CTTGGTTGCTAAGGTCTCGGATTTTGGTCTGTCAAAGACTGCACCCACATTGGAGCAGAC
CCATGTTAGTACAGCCGTGAAAGGTAGTTTTGGATATTTGGATCCTGAATACTTTAGAAG
GCAGCAGCTGACAGAGAAGTCTGATGTGTACTCGTTTGGGGTTGTTCTTTTC
>pac.pt.10.310
TCTTGGGGAATTGGAAGATGGGACTAAGCTCGCCATCAAGCGTGGAAATCCTAGATCCGA
GCAGGGAATTAACGAGTTCCAAACAGAAATTCAAATGCTCTCCAAGCTTAGACATCGCCA
CTTAGTGTCCCTAATTGGGTACTGTGACGAACAATCAGAGATGATCCTTGTCTATGAATA
TATGGCAAATGGTCCTCTCCGTGACCACATTTATGGTTCTACCCTCCCCACATTGTCATG
GAAACAACGACTAGAGATCTGCATTGGTGCTGCGCGAGGCCTGCATTATCTTCATACAGG
CGCTGCGCAGGGGATTATCCACAGAGACGTAAAAACCACAAACATTCTTCTTGATGAGAA
CTTGGTTGCTAAGGTCTCGGATTTTGGTCTGTCAAAGACTGCACCCACATTGGAGCAGAC
CCATGTTAGTACAGCCGTGAAAGGTAGTTTCGGATATTTGGATCCTGAATACTTTAGAAG
GCAGCAGCTGACAGAGAAGTCTGATGTGTACTCGTTTGGGGTTGTTCTTTTC
>pac.ptd.5.2
GGGTGCTCAAGAACAAAATGACAGTCGCTGTGAAACGAAGTGACCCGGGGGGAGATCAGG
GCCTTCTTGAATTCCAGAGAGAGATCATCATCTTATCCAGCATTCGCCATCGCCATCTAG
TGTCACTAATTGGGTATTGTGACGAGCGGTCAGAGATGATACTTGTTTATGAATTCATGG
AAAAGGGAACACTGAGAAGTCATCTTTACAATTCGGATTTGCCTAGTTTGTCATGGAAGC
AGAGACTTGATATATGCATTGGTTCCGCAAGAGGTCTTCATTACCTTCACACTGGCGTAG
CAGGAGGGATCATTCACCGTGATATCAAGTCGACTAACATCTTGCTTGATGAGAATTATG
TTGCTAAGGTTGCAGATTTCGGTCTTTCAAGGGAAGTGCCTGTTGAGCATAGCCGTGGCG
GCTATGTAAGCACAGCAGTAAAAGGCACCTTTGGGTATCTTGATCCAGAGTATTTTAGAC
ATCAACGGTTGACAGAGAAGTCAGATGTGTACTCCTTTGGTGTGGTGCTTCTT
>pac.ptd.5.5
GGTGCTCAAGAACAAAATGACAGTCGCTGTGAAACGAAGTGACCCGGGGGGAGATCAGGG
CCTTCTTGAATTCCAGAGAGAGATCATCATCTTATCCAGCATTCGCCATCGCCATCTAGT
GTCACTAATTGGGTATTGTGACGAGCGGTCAGAGATGATACTTGTTTATGAATTCATGGA
GAAGGGTACACTGAGAAGTCATCTTTACAATTCGGATTTGCCTAGTTTGTCATGGAAGCA
GAGACTTGATATATGCATTGGTTCCGCAAGAGGTCTTCATTACCTTCACACTGGCGTAGC
AGGAGGGATCATTCACCGTGATATCAAGTCGACTAACATCTTGCTTGATGAGAATTATGT
TGCTAAGGTTGCAGATTTCGGTCTTTCAAGGGAAGTGCCTGTTGAGCATAGCCGTGGCGG
CTATGTAAGCACAGCAGTAAAAGGCACCTTTGGGTATCTTGATCCAGAGTATTTTAGACA
TCAACGGTTGACAGAGAAGTCAGATGTGTACTCCTTTGGTGTGGTGCTTCTT
>pac.ptd.8.21
CAAAGGGGTGCTCAAGAACAAAACGACAGTCGCTGTGAAACGAAGTGACCCGGGGGGAGA
TCAGGGCCTTCTTGAATTCCAGAGAGAGATCATCATCTTATCCAGCATTCGTCATCGCCA
TCTAGTGCCACTAATTGGGTATTGTGACGAGCGGTCAGAGATGATACTTGTTTATGAATT
CATGGAAAAGGGAACACTGAGAAGTCATCTTTACAATTCGGATTTGCCTAGTTTGTCATG
GAAGCAGAGACTTGATATATGCATTGGTTCCGCAAGAGGTCTTCATTACCTTCACACTGG
CGTAGCAGGAGGGATCATTCACCGTGATATCAAGTCGACTAACATCTTGCTTGATGAGAA
TTATGTTGCTAAGGTTGCAGATTTCGGTCTTTCAAGGGAAGTGCCTGTTGAGCATAGCCG
TGGCGGCTATGTAAGCACAGCAGTAAAAGGCACCTTCGGGTATCTTGATCCAGAGTATTT
TAGACATCAACGGTTGACAGAGAAGTCAGATGTGTACTCCTTTGGTGTGGTGCTTCTT
>pac.ptd.8.22
TAGGGGTGAAATTGATGATGGCACCCTAGCGGCAATCAAGCGGGCACATCCACAATCTGA
ACAAGGGCTGGCAGAATTTGAGACAGAGATTGAGATGCTTTCGAAGCTGAGGCATAGGCA
CCTAGTCTCCATGATTGGATTCTGTGAGGAGCAGAATGAGATGATCTTGGTGTATGAGTA
CATGGCAAATGGGACCCTTAGGAGTCATCTATTTGGGGGTGATCTCCCACCATTAACATG
GAGGCGACGATTAGATGTATGCATCGGTGCAGCACGTGGACTCCACTACCTCCACACAGG
GGCAGAGAGGAGTACAATCCATAGGGATGTTAAGACAACCAACATACTCTTGGATGAGAA
CTTTGTTGCAAAGATGGCTGATTTCGGGTTGTCAAAAACCGGCCCTTCATTGGATCATAC
CCATGTTAGTACTACAGTAAAGGGGAGCTTTGGATATCTTGATCCTGAATACTTCCGAAG
GCAACAATTGACTGAGAAATCTGATGTTTATTCTTTTGGTGTAGTACTGTTT
>pac.ptd.8.23
CAAGGGAGTTATGGAAGATGGTACAAAGGTTGCAGTAAAGAGGAGTAATCCCCTGTCCCA
ACAGGGTCTTGCGGAGTTTCGGACTGAAATCGAAATGTTGTCTCAATTCCGCCACCGTCA

TCTGGTCTCACTGATTGGGTATTGTGACGAGCAGAAAGAGATGATCCTAGTTTATGAGTA
CATGGAGAATGGGACACTCAATAGTCATTTGTATGGCTCCAATTTTCCCAGCTTGAGTTG
GAAGCAGAGGCTAGAAATATGCATTGGATCAGCCAGAGGACTTCACTACCTTCATACTGG
TTTTCCGAAGGCTATTATCCACCGTGATGTTAAGTCTGCCAACATCTTACTTGATGAGAA
TCTCATGGCCAAGGTTGCTGATTTTGGGCTTTCCAAGACAGGTCCTGGGATCGATCAGAC
CCATGTCAGCACGGCGGTTAAGGGGAGTTTTGGATATCTCGATCCGGAATATTTCATAAT
GCAACGACTGACCGAGAAATCGGATGTCTACTCATTTGGGGTGCTTTTGTTT
>pac.ptd.9.41
TAAAGGAGTTGTTGATGGAGGGCTCAAGGTAGCCATTAAAAGAGCAAACCCATCTTCAGA
ACAGGGAGTTCATGAGTTCCAGACCGAAATTGAGATGCTTTCTAAGCTAAGACACAGGCA
TTTAGTCTCTCTGATTGGGTACTGTGAAGAAAATGAAGAGATGATCTTGGTTTATGATTA
CATGGCTTATGGGACTCTAAGGGAGCATCTCTATAAGAGCAACAAGCCTACTCTTTCATG
GAAACAGAGGCTAGAAATCTGTATCGGGGCTGCAAGGGGACTCCACTACCTTCATACTGG
TGCAAAGTACACCATCATCCACAGGGACGTAAAGACCACGAACATTCTCTTGGATGAGAA
CTGGGTAGCCAAAGTTTCAGATTTTGGGCTCTCAAAAACGGGTCCCAACCTCAACCAGAC
CCACGTTAGTACAGTGGTGAAGGGTAGTTTTGGCTACTTGGATCCTGAGTACTTCAGACG
CCAGCAATTGACAGAGAAATCTGATGTATACTCATTTGGGGTTGTTCTCTTT
>pac.Erf.2
TCAGCTTCGAAGTGGCCGTCATGTAGCGGTAAAGATGTTAGGCAAGTCCAAAGGTAATGG
CCAAGAATTCATCAATGAAGTCGCCACCATTGGGAGGATTCATCATGTTAATGTGGTGCG
ACTTATTGGATTCTGTTCAAACGGATCAAAACGGGCTCTGATTTATGAATTCATGCCTAA
TGGGTCTTTAGAAAAGTATACCTTTTCCCAAGAAAAAGAAGAAAGACTCTCACTGAGTTG
GGATAAAATGTATGAGATTGCCCTAGGAGTTGCTCGTGGGATTGAGTATTTACATCGAGG
ATGTGACATTCAAATTTTACATTTTGATATCAAGCCCCACAACATTCTTCTTGATGAGAA
CTTTGTTCCAAAGATTTCTGACTTTGGGCTCGCAAAATTCTATCCAATAAATGATAATAC
TATCACTCTTACTGCAGCACGAGGAACA
>pac.Erf.3
AACTCTTGATCATGGGGAAACAATTGTTGCAGTAAAGGTACTCAACCTTCAGCAACAAGG
AGCTTCCAAGAGTTTTATGGCGGAGTGTGAAGCCTTGAGAAATATTCGACACCGTAATCT
TGTCAAGATCTTAACCATTTGCTCAAGCATGGATTTCAAAGGAAATGATTTCAAAGCTCT
AGTTTTCGAGTTCATGCCTAATGGGAATTTGGAGAGATGGTTGCATCCTGAAACATATCA
TACACAAGATGAATTGGGAAATTTGAACTTCCTTCAAAGGCTAAACATTGCCATTGATGT
GGCTTCCGCTTTGGAATATCTTCATCACAACTGTCCAACACCAATCATCCATTGTGATCT
AAAGCCAAGCAACATTCTTCTTGATAATGACATGACTGCCCATGTGGGTGACTTTGGGTT
ATCAAGGCTCCATTTAGAGACGAGCAATAGATCTTCTCAAAATCAAACCAGCTCGATTGG
AATAAGAGGATCT
>pac.Erf.4
AATTCTTCATCAAGATGGAAGACCAGTAACATTTGCTCTGAAGGTGTTGAACGTTCAACA
ACAAGGAGCATCCAAGACTTTTATGGCAGAATGCAAAGCATTGAGAAATATTCGACATCG
AAATCTTCTCAAGATCATAACTTCTTGCTGAGCGTGGATTTTATGGGTAATGATTTCAAG
GCTCTAATTTTTGAGTTTATGACTAATGGGAATCTAGAGAGTTGGTTGCACCCAAATATC
GACGGACAACATCAATCTAGATACTTGAACATAAGCCAAAGACTGGACATGGCAATGGAT
GTTGCTTCTGCATTAAATTATCTTCATAACCAATGTCCAACACCAATTGTTCATTGTGAT
CTAAAACCAAGCAATGTTCTTCTTGATGATGATATGTCTGCCCATGTGGGTGACTTTGGA
CTTGCTAAGTTCCTTTTTAGGGCTCCCAGCGATTCTGGTGAAAGTCAATACAGTTCAGTT
GCAATTAAGGGTACT
>pac.Erf.5
AATTCTTGGTAACGGTGAAACAATTGTTGCAGTGAAGGTACTCAACCTTCAACAACAAGG
AGCTTCCAAGAGTTTCATGGCCGAGTGTGAAGCCTTGAGAAACATTCGACATCGAAATCT
TGTCAAGATCTTAACTACTTGCTCAAGCACTGATTTTGAAGGAAATGATTTTAAAGCTCT
AGTTTTTGAGTTCATGCCCAATGGGAGTTTGGAGAAATGGTTGCATCCGGGAGCAGATGA
TGCACAAGATGGATTGATGAATATAAACCTTCTCCAAAGGTTAAACATTGCAATTGATGT
AGCTTCTGCCTTGGAATATCTTCATCACCACTGTCAAATACCAATCATTCATTGTGATTT
AAAACCAAACAACATTCTTCTTGATAACGACATGACTGCCCATGTGGGTGATTTTGGGTT
ATCAAGGTTTCTTCCAGAGGCAACCAATGCATTTTCTCAAAACCATAGCAGCTCAATTGG
TATAAGAGGATCT
>pac.Erf.6
ACTTCTAAATCAGGGTGAAACAAATGTTGCAGTGAAGGTATTCAACCTTCCACAACATGG
AGCCTCTAAAAGTTTCATGGCTGAGTGTGAATCCTTGAGAAACATCCGGCATCGGAATCT
CGTCAAGGTTATAACTTCCTGCTCGAGTATAGATTTTGCGGGCAATGATTTCAAAGCTCT
AGTTTACGAGTTTATGAGTAATGGAAGTTTAGAGAGGTGGTTATATCCAAATGCAGAAGT
GGCACAAGTTGAACAAAGGAATTTAAATATTCTTCAAAGACTTAACACTGCCATTGATGT
TGCCTCTGCACTAGATTATCTTCATCACAACTGTAAAACACCAATCATTCATTGTGATCT
AAAGCCAAACAACATTCTCCTTGATGACGACATGGTTGCTCACGTGGGTGATTTTGGTTT
ATCAAGATTCCTCCCAATGACGATTAATAATTCTCAAAGTCAAACCAGCTCTATTGGAAT
AAAAGGATCC
>pac.Erf.7
AATATTTCATCAAGATGGAAGATCGGTATCAGTTGCTGTTAAGGTGTTGAACCTTCAACA
ACGAGAAGCATCCAAGACTTTCATGGCTGAATGCAAAGCATTAAGAAATATCAGGCATCG
AAATCTTCTCAAGATCTTAACTTCTTGTTCAAGTGTAGATTTTAAGGGTAATGATTTCAA

GGCTCTAGTCTTGGAGTTCATGTCTAATGAGAGTCTAGAGAGTTGGTTGCACCCAAATAT
AGATGAACACCATCAATCAAGATACTTGAATTTTAGCCAAAGACTGAACATAGCAATTGA
TGTTGCTCTTGCATTGGATTATCTTCATAACAATTGTCCAACACCTATTGTTCATTGTGA
TCTAAAACCAAGCAATGTTCTTCTTGATGATGAAATGACTGCCCATGTGGGTGACTTTGG
ACTCGCCAAGTTCCTTTCTAGGGCTACCAGCAATTCTGGTGAAGGTCAACACAGCTCAGT
TGCAATTAAGGGAACT
>pac.Erf.9
TCAGCTTCGAAGTGGCCATCATGTCGCGGTAAAGATGTTAGGCAAGTCCAAAGGTAATGG
CCAAGAATTCATCAATGAAGTCGCCACCATTGGGAGGATTCATCATGTTAATGTGGTGCA
ACTTATTGGATTCTGTTCAAACGGATCAAAACGGGCTCTGATTTATGAATTCATGCCTAA
TGGGTCTTTAGAAAAGTATACCTTTTCCCAAGAAAAAGAAGAAAGACTCTCACTGGGTTG
GGATAAAATGTATGAGATTGCCCTAGGAGTTGCTCGTGGGATTGAGTATTTACATCGAGG
ATGTGACATTCAAATTTTACATTTTGATATCAAGCCCCACAACATTCTTCTTGATGAGAA
CTTCGTTCCAAAGATTTCTGACTTTGGGCTCGCAAAATTCTATCCAATAAATGATAATAC
TATCACTCTTACTGCAGCACGAGGAACA
>pac.Erf.10
AATTCTTGGTAACGGTGAAACAATTGTTGCAGTGAAGGTACTCAACCTTCAACAACAAGG
AGCTTCCAAGAGTTTCATGGCCGAGTGTGAAGCCTTGAGAAACATTCGACATCGAAATCT
TGTCAAGATCTTAACTACTTGCTCAAGCACTGATTTTGAAGGAAATGATTTTAAAGCTCT
AGTTTTTGAGTTCATGCCCAATGGGAGTTTGGAGAAATGGTTGCATCCGGGAGCAGATGA
TGCACAAGATGGATTGATGAATATAAACCTTCTCCAAAGGTTAAACATTGCAATTGATGT
AGCTTCTGCCTTGGAATATCTTCATCACCACTGTCAAATACCAATCATTCATTGTGATTT
AAAACCAAACAACATTCTTCTTGATAACGACATGACTGCCCATGTGGGTGATTTTGGGTT
ATCAAGGTTTCTTCCTGAGGCAACCAATGCATTTTCTCAAAACCATAGCAGCTCAATTGG
TATAAGAGGATCT
>pac.Erf.11
AATGGTTCATCAGGATGGAAGACTAGTATCGGTTGCTGTGAAGGTATTGAACCTTCAACA
ACAAGAAGCATCCAAGACTTTTATGGCCGAATGCAAAGCATTGAGGAATGTCCGGCATCG
AAATCTCCTCAAGATCATTACTTCTTGTTCGAGTGCGGATTTTAAGGGTAATGAGTTTAA
GGCTCTAGTTTTGGAGTTCATGCCTAATGGGAGTCTAGAGAGTTGGTTGCACCCAAGGAT
AGATGGACAACTTCAATTGAGATACTTGAGCATTAGTCAAAGATTGAATATAGCAATTGA
TGTTGCTTTAGCATTGGATTATCTTCACAACCATTGTCAAATTCCTATTGCTCATTGTGA
TCTAAAACCAAGCAATGTTCTTCTTAATGATGACACGACTGCCCATGTTGGTGACTTTGG
ACTAGCCAAATTCCTTTATAGGGCTACCAGCAATTCTGCTGAGGGTCAAAACAGTTTGGC
TACAATTAAAGGAACT
>pac.Erf.13
GAGGCTTGTCGACGGACGTTTGGTTGCGGTTAAAGTCTTGAATGAATCCAAAGGTAAAGG
GGATGATTTCATTAACGAGGTCACTACCATCGGCAGGACAAACCACCATAATATTGTTCG
TCTATTGGGCTTCTGTTCTGAAGGGTCCAAAAGAGCTCTAATCTATGAATTCATGTCCAA
TGGATCTCTTGAAAAATTCATTTATGTTGACAAACCAAGGATGACACAACCTTTAGCATG
GGAAAACCTTTACCAAATTGCGATTGGCATTGCCAAGGGACTTGAGTACTTGCACCGTGG
TTGTAACACCCATATTTTACATCTTGACATAAAACCTCATAACATTCTCTTAGATGAGAA
ATTTTGTCCTAAGATCTCAGACTTTGGGTTAGCTAAACAATGCCTCACAGGAGAGAGTGC
AGTATCTTTGCATGGGGCTCGAGGGACT
>pac.Erf.14
AGTTCTACGGGAAGATGAAGCTGTTGTTGCCGTTAAGGTATTGAATCTTCAACAACATGG
AGCTTCCAAGAGTTTCATATCTGAATGCAAAACCCTGACAAACATTCGGCATCGTAATCT
TGTCAAGATCTTGACTGCCTGCTCAAGTGTGGATTTTAGGATGAATGATTTCAAAGCTCT
GGTTTTTGAGTTCATGTCAAATGGGAACCTAGACGAGTGGTTGCATCCAAGAATTGGTGG
GCAACATCAGCTCAAGAGTTTGAGCCTAATTCAAAGGTTAAACATAGCCATTGATGTGGC
TTCTGCACTGGAATATCTTCATTTCCATTGCCAAATTCCTATTGTTCATTGTGATCTAAA
ACCAAGCAATGTGCTTCTTGATGAAGATATGACTGCCCATGTTGGTGACTTCGGACTAGC
ACGGTTCCTCTTTGAAACATCCAATATTTCCTCCAAAAATCAAACCATATCGGCCGGGCT
AAAGGGTTCT
>pac.Erf.2B.210
AGCCTTATCAAATGGCCGCAGAGGGTCGTTGCTGTCAAAAGACTAGAGAATGTGGTGAAT
GGAGGAGAAAGGGAGTTTCAGAATGAGATGACATCCATTGGGAGAACTTATCATAGAAAT
CTAGTCCGATTGCTAGGTTATTGCCAAGATGGTCATCAAAGGCTTCTCGTGTACGAGTAC
ATGAGCAAAGGATCACTCGCGGACTTCCTCTTTAAACCCGATTGTCCCCCAAGTTGGGAC
GAAAGAGTGCGAATCGCTCTAGACATAGCTAGAGGTATCCTATATCTGCATGAAGAATGT
GAAACTCAAATCATCCACTGTGACATAAAACCTCAAAACATACTCATGGATGAGTACCAG
TGTGCGAAGATTGCCGATTTTGGACTCGCAAAGCTGCTGAAGCCAGACCAAACTAGGACT
TTCACTGGGATCCGAGGAACAAGAGGGTATGTTGCTCCCGAGTGGCATCGCAACTTGCCC
ATAACAGTGAAA
>pac.Erf.2B.211
AATCTTATCAAGTAGCCAGAAGGTTGTTGCTGTCAAAAGACTAGAGAATATGGTGAAAGA
AGGAGAAAATGAGTTTCAGAATGAGATGACAACGATCGGGAGAACTCATCACAGAAATCT
AGTCCGACTTCTAGGTTACTGCCATGAGGGTACTAATAGGCTTCTTGTGTACGAGTACAT
GAGCAACGGATCACTCGCGGACTTCCTCTTTAAATACGAAAGGCATCCAAATTGGGACGA
AAGAGTGGGAATTGCTCTACACATAGCTAGAGGTATCCTTTATTTGCATGAAGAGTGTGA

AACCCAGATCATTCATTGTGACATAAAACCTCAAAACATACTCATAGATGAGTATCGTTG
TGCAAAGATAGCCGACTTCGGACTTGCGAAACTGCTGAAGCCAAACCAAAGCATGACCTC
CACTGGGATCAGAGGGACAAGGGGGTATGTTGCGCCCGAGTGGCACCAGAACATGCCCAT
AACTGTGAAG
>pac.W.ArA.3
TATGCTGAGAACCAAATCCTTGGTCGTGGAGGCTATGGCACCGTATACAAAGGAATTTTA
GCAGACCACAGAACAGTGGCTGTTAAAAAATCCAAAATAGTGGATCAGAGCCAAATTGAG
CAATTCATAAATGAAGTTGTTATTCTCTCTCAGATCAACCATCGGAATGTGGTGAGACTT
CTAGGATGTTGTTTGGAGACTGAGGTCCCTCTACTAATTTATGAGTTTATCAACAATGGA
ACCCTCTCGCACCATATCCATGATGAGAGTCATATATCCTCAATTTCTTGGGGATGTCGT
TTGAGGATAGCTACAGAAACTGCGGAAGCACTTGCTTATCTACATTCTGCAGCTTCTCCA
CCAATCATTCACAGGGATATTAAGTCTACTAACATACTCTTGGATGAAGATTACACGGCT
AAAATAGCAGACTTTGGAGCTTCAAGGTTGGTTCCTCTTGATTACACACAATTGACTACA
TTAGTACAAGGGACGTTGGGGTACTTGGACCCAGAATATTTTCATTCAAGTCAATTAACT
>pac.W.ArA.4
TTCTCGGAGAAGAATTTAATCGGGGGTGGCGGGTTCGGAGTTGTGTATAAGGGGACCTTA
TCGGATGGAACTTGGGTTGCTGTGAAGAGGATCATAGAGTCAGATTTTCATGGTGATGCG
GAGTTCTGCAATGAAGTTGAGATCCTTAGCAATTTGAAGCACCGGAATCTCGTCCTGCTA
AGAGGCTGCTGTATGACAGATGAAGAGAGCAGCAAACAGAGGTATCTGGTGTACGAGTAT
ATGCCCAATGGGAACCTGAAGGATCATCTCTTCTTGTTAGACAAGCAACCGCTGAGTTGG
CCCCAAAGGAAGAGCATAATCTTGGATGTGGCCAAGGGGATTGCCTATCTTCATTATGGA
GTGAAACCTGCAATCTATCACAGAGACATCAAGCCCACTAACATACTCTTGGATGCAGAT
ATGAAAGCAAGAGTGGCAGATTTTGGGCTAGTCAAGCAGAGCAGAGAAGGACAGTCCCAG
CTCATCACCAGAGTCGCTGGCACACACGGCTACCTAGCTCCGGAATATGCCCTCTATGGG
CAGTTGACT
>pac.W.ArA.5
TTCTCTCAGAAGAATTTAATCGGGCGTGGCAGCTTTGGAGTTGTGTATAAGGGGATCCTA
CCGGATGGAACTTGGGTTGCTGTGAAAAAGATCACAGAGTCAGATTTTCAAGGTGATGCT
GAGTTCTGCAATGAAGTTGAGATCATTAGCAATTTGAAGCACCGGAATCTTGTCCCGCTT
AGAGGCTGCTGTGTGATAGATGGGGATTGTGATGAAGGGAGCGGCAAACAGAGGTATCTG
GTGTACGAGCATATGCCCTATGGGAACCTCGAGGATCATCTCTTCTTGTCGAACCCACAC
AGTGGAATATTCAACAAGCAACCTCTGAGTTGGCCCCAAAGAAAGAGCATAATCTTGGAT
GTGGCCAAGGGGATTGCCTATCTTCACTATGGAGTGAAACCTGCAATATATCATAGAGAC
ATCAAGCCTACGAACATACTTTTGGATGCAGATATGAAAGCAAGAGTGGCAGACTTTGGG
CTAGTCAAGCAAAGCAGAGAAGGACAGTCCCATCTCATCACCAAAGTTGCTGGCACACAT
GGCTACCTAGCTCCGGAGTATGCTCTCTATGGGCAGTTAACT
>pac.W.ArA.6
TATGCTGAGAACcAAATCCTTGGTCGTGGAGGCTATGGCACCGTATACAAAGGAATTTTA
GCAGACCACAGAACAGTGGCTGTTAAAAAATCCAAAATAGTGGATCAGAGCCAAATTGAG
CAATTCATAAATGAAGTTGTTATTCTCTCTCAGATCAACCATCGGAATGTGGTGAGACTT
CTAGGATGTTGTTTGGAGACTGAGGTCCCTCTACTAATTTATGAGTTTATCAACAATGGA
ACCCTCTCGCACCATATCCATGATGAGAGTCATATATCCTCAATTTCTTGGGGATGTCGT
TTGAGGATAGCCACAGAAACTGCGGAAGCACTTGCTTATCTACATTCTGCAGCTTCTCCA
CCAATCATTCACAGGGATATTAAGTCTACTAACATACTCTTGGATGAAGATTACACGGCT
AAAATAGCAGACTTTGGAGCTTCAAGGTTGGTTCCTCTTGATCACACACAATTAACTACA
TTAGTACAAGGGACGTTGGGGTACTTGGACCCAGAATATTTTCATTCAAGTCAATTAACT
>pac.W.ArA.7
TATGCTGAGAATCGAATCCTTGGTCGTGGAGGCTATGGTACCGTATACAAAGGAATTTTA
GCAGACCACAGAATAGTGGCTGTTAAAAAATCCAAAATAGTGGATGAGAGCCAAATTGAA
CAATTCATAAATGAGGTTGTTATTCTCTCCCAGATCAACCATAGGAATGTGGTGAGACTT
CTAGGTTGTTGTTTGGAGACTGAGGTTCCTTTATTAATATATGAGTTCATCAACAATGGA
ACCCTCTTCCATCATATCCATGATGAAGGTCATGTATCATCAATTTCATGGGGAAGTCGT
ATGAGGATAGCTGCAGAAACTGCAGGAGTACTTGCTTATCTTCACTCTGCAGCTTCTATA
CCAATCATTCATAGGGATATTAAGTCTACTAACATACTCTTAGACGAAAAGTACACAGCT
AAGGTGGCAGACTTTGGAGCTTCAAGGTTGGTTCCTTTAGATCAGACTCAATTGACTACA
TTAGTACAAGGAACGCTCGGTTACTTGGACCCAGAATACTTTAACTCGAGTCAACTAACG
>pac.W.ArA.8
TATAATGAGAACCGAATTCTTGGTCAAGGAAGTCAAGGTACGGTATATAAAGGCATGTTA
CCAGATGGGAGAATCGTAGCAATTAAGAAGTCCAAACTAGTAAATGAAGGCCAAATTGAG
CAATTCATTAATGAGCTTGCCATACTTTCCCATATCAACCACAGAAACGTGGTTAAATTG
TTAGGTTGTTGTTTGGAGACAGAAGTTCCTCTACTTGTTTACGAGTTTATTTCCAACGGA
ACCCTTTTCTATCATATCCATGAAAAAAGTGAAGAGTTCCTAAGTTCATGGGATAATCGC
TTAAGAATTGCTACAGAAGTTGCAGGTGCACTTGCTTACTTGCATTCAGCAGCTTCAATA
CCAATTTATCATAGAGACATCAAGTCTACCAACATACTCATAGATGGTAAATACACAGCT
AAAGTCTCAGACTTTGGTATTTCAAGGTCTGTTCCAATTGATAAAACACACCTAACTACA
TCAGTCCAAGGGACTTTTGGTTACATGGATCCAGAGTATTTCCAATCAAACCAATTCACA
>pac.W.ArA.9
TATAATGAGAACCGAATTCTTGGTCAAGGAGGCCAAGGTACAGTATACAAAGGTATGCTA
CCAGATGGAAGAATTGTAGCAATTAAGAAATCCAAAATAGTGAATGAGGGTCAAATCGAA
CAGTTCATTAATGAGGTTGTCATACTTTCTCATATCAACCACAAGAACGTGGTTAAATTG

TTGGGTTGTTGTTTGGAGACAGAAGTTCCTTTACTTGTTTATGAGTTCATCTCCAATGGA
ACCCTTTTCCATCACATCCATGTCCAAAGTGAGGAATTCCAATGTTCATGGGACAATCGG
TTAAGAATTGCTATAGAACTTGCAAATGCACTTGCCTATTTGCATTCAGCAGCTTCCATA
CCAATCTATCATAGAGATATCAAGTCTACCAACATACTATTAGATAGTAAATATACGGCT
AAAGTATCAGACTTTGGGATTTCAAGGTCCGTTCCAAACGATAAAACTCACTTAACCACA
TTAGTCCAAGGGACCTTTGGATACTTGGATCCAGAGTATTTCCAGTCAAGTCAATTCACA
>pac.W.ArA.11
TATGCTGAGAACcAAATCCTTGGTCGTGGAGGCTATGGCACCGTATACAAAGGAATTTTA
GCAGACCACAGAACAGTGGCTGTTAAAAAATCCAAAATAGTGGATCAGAGCCAAATTGAG
CAATTCATAAATGGAGTTGTTATTCTCTCTCAGATCAACCATCGGAATGTGGTGAGACTT
CTAGGATGTTGTTTGGAGACTGAGGTCCCTCTACTAATTTATGAGTTTATCAACAATGGA
ACCCTCTCGCACCATATCCATGATGAGAGTCATATATCCTCAATTTCTTGGGGATGTCGT
TTGAGGATAGCTACAGAAACTGCGGAAGCACTTGCTTATCTACATTCTGCAGCTTCTCCA
CCAATCATTCACAGGGATATTAAGTCTACTAACATACTCTTGGATGAAGATTACACGGCT
AAAATAGCAGACTTTGGAGCTTCAAGGTTGGTTCCTCTTGATCACACACAATTAACTACA
TTAGTACAAGGGACGTTGGGGTACTTGGACCCAGAATATTTCATTCAAGTCAATTAACT
>pac.W.ArA.12
TTCTCTCAGAAGAATTTAATCGGGCGTGGCAGCTTTGGAGTTGTGTATAAGGGGATCCTA
CCGGATGGAACTTGGGTTGCTGTGAAAAAGATCACAGAGTCAGATTTTCAAGGTGATGCT
GAGTTCTGCAATGAAGTTGAGATCATTAGCAATTTGAAGCACCGGAATCTTGTCCCGCTT
AGAGGCTGCTGTGTGATAGATGGGGATTGTGATGAAGGGAGCGGCAAACAGAGGTATCTG
GTGTACGAGCATATGCCCTATGGGAACCTCGAGGATCATCTCTTCTTGTCGAACCCACGC
AGTGGAATATTCAACAAGCAACCTCTGAGTTGGCCCCAAAGAAAGAGCATAATCTTGGAT
GTGGCCAAGGGGATTGCCTATCTTCACTATGGAGTGAAACCTGCAATATATCATAGAGAC
ATCAAGCCTACGAACATACTTTTGGATGCAGATATGAAAGCAAGAGTGGCAGACTTTGGG
CTAGTCAAGCAAAGCAGAGAAGGACAGTCCCATCTCATCACCAAAGTTGCTGGCACACAT
GGCTACCTAGCTCCGGAGTATGCTCTCTATGGACAGTTAACT
>pac.W.ArA.14
TATGCTGAGAACCGAATCCTTGGTCGTGGAGGCTATGGTACTGTATACAAAGGAATTTTA
GCAGACCACAGAATAGTGGCCGTTAAAAAATCCAAAATAGTGGATGAGAACCAAATTGAA
CAATTCATTAATGAGGTTGTTATTCTCTCCCAGATCAACCATAGGAATGTGGTAAGACTT
CTAGGTTGTTGTTTGGAGACTGAGGTTCCTTTACTAATTTATGAGTTCATCAATAATGGT
ACCCTATTCCACCATATCCATGATGAGGGTCATGTGTCATCAATTTCTTGGGAAATTCGT
TTGAGGATAGCTGCAGAAACTGCAGGAGCACTTGCCTATCTACACTCCGCAGTTTCTACA
CCAATCATTCATAGGGATATTAAGTCTGCTAACATACTCTTAGATGAAAATTACACATCT
AAAATAGCAGACTTTGGAGCTTCAAGGTTGGTTCCTTTAGATCAGACTCAATTGACTACA
TTAGTACAAGGGACGCTAGGTTACTTGGACCCAGAATACTTTCATTCGAGTCAATTAACG
>pac.W.ArB.305
GAATGGCTTCTCAGAGAAACAAAGGCTAGGAACTGGAGCCTATGGAACAGTTTATGCAGG
AAAGCTTCACAATGTCGACTGGGTTGCCATCAAAAAGATCAGACACCGAGACAATGATGG
CATTGAGCAAGTCATGAATGAGATCAAGCTCCTTTCATCGGTGAGCCACCCGAATCTAGT
CCGCCTCCTAGGCTGCTGTATAGAGAAGGGTGAACAGATCCTTGTGTATGAATTCATGCC
AAATGGAACTCTATCTCAGCATCTACAAAGGGAGATAAGCACTGGCCTCCCTTGGATGAC
TCGACTCACCATTGCCACCGAAACTGCTCACGCTATTGCCCATCTACACTCTGCTATTAA
CCCACCTATATTCCACAGAGACATCAAATCCAGCAACATCCTCTTAGACTATAACTACAA
TTCGAAAGTCGCTGACTTTGGTCTATCTAGACTCGGTATGACTGAGTCGTCCCACATCTC
CACAGCTCCACAAGGAACTCCAGGGTATCTAGACCCTCAGTATCATCAGAACTTTCACCT
TTCTGATAAAA
>pac.W.ArB.312
GAATGGCTTCTCGGAGAAACAAAGGCTAGGAACTGGAGCCTATGGAACAGTTTATGCAGG
AAAGCTTCACAATGTCGACTGGGTTGCCATCAAAAAGATCAGACACCGAGACAATGATGG
CATTGAGCAAGTCATGAATGAGATCAAGCTCCTTTCATCGGTGAGTCACCCGAATCTAGT
CCGCCTCCTAGGCTGCTGTATAGAGAAGGGTGAACAGATCCTTGTGTATGAATTCATGCC
AAATGGAACTCTATCTCAGCATCTACAAAGGGAGATAAGCACTGGCCTCCCTTGGATGAC
TCGCCTCACCATTGCCACCGAAACTGCTCACGCTATTGCCCATCTACACTCTGCTATTAA
CCCACCAATATTCCACAGAGACATCAAATCCAGCAACATCCTCTTAGACTATAACTACAA
TTCGAAAGTCGCTGACTTTGGTCTATCTAGACTCGGTATGACTGAGTCGTCCCACATCTC
CACAGCTCCACAAGGAACTCCAGGGTATCTAGACCCTCAGTATCATCAGAACTTTCACCT
TTCTGATAAAA
>pac.W.VtA.101
TACAGTATACAAAGGTATGTTAGTAGACGGAATGATTGTAGCAATTAAGAAGTCAAAAAT
AGTGGACGAAGGCCAAATCGAGCAATTCATTTATGAGGTTGTCATGCTTTCCCAAATCAA
CCACAGGAATGTGGTTAAATTATTAGGTTGTTGTTTGGAGACAGAAGTTCCTCTACTTGT
TTATGAGTTCATCTCCAAAGGAACCCTTTTCCACTATATCCATGACCAAAGTGAGGAGTT
CCCAAATTCTTGGGACAATCGCTTAAAAATTGCTACAGATGTTGCAACTGCACTTGCTTA
CTTGCATTCAGCAGCTTCGATGCCAATCTACCACAGAGATATAAAGTCGTCCAACATACT
GTTGGATGACAAATATATAGCTAAAATATCAGACTTTGGGATTTCAAGATCCATCCCAAC
AGATAAAACTCACTTGACCACATTAGTTCAGGGGACTTTGGGGTACTTGGATCCAGAATA
TTACCAATCAAGCCAATTTACAGAGAAAA
>pac.W.VtA.102

TACAGTATACAAAGGTATGTTAGTAGATGGAACAATTGTAGCAATTAAGAAGTCAAAAAT
AGTGGATGAAAGCCAGATTGAGCAATTCATTAATGAGGTTGTCATGCTTTCCCAGATCAA
CCACAGGAATGTGGTTAAATTATTAGGTTGTTGTTTGGAGGCAGAAGTTCCTCTACTTGT
TTACGAGTTCATCTCCAATGGAACCCTTTTCCATTATATTCATGACCAAAGTGAAAAGTT
CCCAAATTCTTGGGACAATCGCTTAAAAATTGCTACAGATGTTGCAGCTGCACTTGCCTA
CTTACATTCAGCAGCTTCGATACCAATCTACCATAGAGATATCAAGTCTTCCAACATACT
CCTGGATGATAAATATATAGCTAAAATATCAGACTTTGGGGCTTCAAGGTCCATCCCAAC
AGATAAAACTCACTTGACCACATTAGTTCAGGGGACTTTTGGGTACTTGGATCCAGAATA
TTTCCAATCAAACCAATTTACAGAAATAA
>pac.W.VtA.105
TACAGTATACAAAGGTATGTTAGTAGACGGAATGATTGTAGCAATTAAGAAGTCAAAAAT
AGTGGACGAAGGCCAAATCGAGCAATTCATTTATGAGGTTGTCATGCTTTCCCAAATCAA
CCACAGGAATGTGGTTAAATTATTAGGTTGTTGTTTGGAGACAGAAGTTCCTCTACTTGT
TTATGAGTTCATCTCCAAAGGAACCCTTTTCCACTATATCCATGACCAAAGTGAGGAGTT
CCCAAATTCTTGGGACAATCGCTTAAAAATTGCTACAGATGTTGCAACTGCACTTGCTTA
CTTGCATTCAGCAGCTTCGATGCCAATCTCCCACAGAGACATAAAGTCGTCCAACATACT
CTTGGATGACAAATATATAGCTAAAATATCAGACTTTGGGATTTCAAGATCCATCCCAAC
AGATAAAACTCACTTGACCACATTAGTTCAGGGGACTTTGGGGTACTTGGATCCAGAATA
TTACCAATCAGGCCAATTTACAGAGAAAA
>pac.W.VtA.107
TACAGTATACAAAGGTATGCTCCATTGTGGAAGAATTGTAGCAATTAAAAAGGCTAAATT
AGTTGATGAAGGCCAAGTCGAGCAATTCATTAATGAGGTTGTCATACTTTCCCAGATCAA
CCATAGAAATGTGGTTAAATTGTTAGGTTGTTGTTTGGAGACAAAAGTTCCTTTACTAGT
TTATGAGTTCATTTCCAATGGAACTCTTTTCTATCATATTCATGACCAAAGTGAGGAATT
CCCAATTTCATGGGTCAATTGTTTAAGAATTGCTACAGAAGTTGCTGGAGCACTTGCTTA
CCTGCATTCTGCTGCTTCTATACCAATCAATCATAGAGACATCAAGTCTACCAACATACT
TCTAGATGAAAAGTATAACGCCAAAGTATCAGACTTTGGGATTTCAAGGTCTGTTGCAAC
TGATAAAACTCATTTAACTACCACAGTGCAAGGGACTTTTGGATACTTTGACCCAGAATA
CTTTCAATCAAGCCAATTCACAGAGAAGA
>pac.W.VtA.108
TACAGTGTACAAAGGTATGTTGCAAGATGGAAGAAATGTAGCAACTAAGAAGTCCAGAAT
AGTTGATGAAGGCCAAATCGAGCAATTCATCAACGAGGTTGTCATACTTTCCCAAATCAA
CCATAGGAATGTGGTTAAATTATTAGGTTGTTGTTTGGAGACGGAAGTTCCTTTACTTGT
TTATGAGTTCATTTCCAATGGAACTCTTTTCTATCATATTCATGACCAAAGTGAGGAATT
CCCAATTTCATGGGTCAATTGTTTAAGAATTGCTACAGAAGTTGCTGGAGCACTTGCTTA
CTTGCATTCTGCTGCTTCTATACCAATCTATCATAGAGACATCAAGTCTACCAACATACT
TCTAGATGATAAGTGTAAAGCCAAAGTTGCAGATTTTGGAACTTCCAGGTCAGTCTCTGT
TGACAAAACTCATTTAACTACACTAGTGCAGGGGACCTTGGGGTACCTGGACCCAGAATA
CTTCCAATCAAGCCAATTCACAGATAAGA
>pac.W.VtA.109
AACAGTATATAAAGGAATTTTAGAGGACCATAGAATAGTGGCTATTAAAAAATCCAAATT
AGTGGATGAGAGCCAAATTGAACAATTCATCAATGAGGTTGTTATTCTCTCCCAGATCAA
CCATAGGAATGTGGTGAAACTTCTAGGTTGTTGTTTGGAGACTGAAGTACCTTTACTGAT
TTATGAGTTTATCAACAACGGAACCCTTTTCCACCATATCCATGATGAGGGTCATGTGTC
ATCAATTTCTTGGGAAAGTCGTTTGAGAATAGCTGCAGAAACTGCAGGAACACTTGCTTA
TTTGCATTCTGCAACTTCTATACCAATTATACATAGGGATATTAAGTCTACTAACATACT
CTTAGATGATAATTATACAGCTAAAGTAGCAGACTTTGGAGCTTCAAGGTTGGTTCCTTT
AGATCAAACTCAATTGACTACATTAGTACAGGGGACATTAGGGTACTTGGACCCAGAATA
CTTTATTTCAAGTCAATTAATAGAGAAGA
>pac.W.VtA.110
TACAGTATACAAAGGTATGCTACTAGATGGAAGAATTGTAGCAATTAAGAAATCCAAAAT
AGTGAATGAGGGTCAAATCGAACAGTTCATTAATGAGGTTGTCATACTTTCTCATATCAA
CCACAAGAACGTGGTTAAATTGTTGGGTTGTTGTTTGGAGACAGAAGTTCCTTTACTTGT
TTATGAGTTCATCTCCAATGGAACCCTTTTCCATCACATCCATGACCAAAGTGAGGAATT
CCAATGTTCATGGGACAATCGGTTAAGAATTGCTATAGAACTTGCAAATGCACTTGCCTA
TTTGCATTCAGCAGCTTCCATACCAATCTATCATAGAGATATCAAGTCTACCAACATACT
ATTAGATAGTAAATATACAGCTAAAGTATCAGACTTTGGGATTTCAAGGTCCGTTCCAAA
CGATAAAACTCACTTAACCACATTAGTCCAAGGGACCTTTGGATACTTGGATCCAGAGTA
TTTCCAATCAAGTCAATTCACAGAGAAAA
>pac.W.VtA.113
TACAGTATACAAAGGTATGTTAGTAGACGGAATGATTGTAGCAATTAAGAAGTCCAAAAT
AGTGAATGAAGGGCAAACTGAGCAGTTCATTAATGAGCTCGTCATTCTTTCACATATCAA
CCACAGGAACGTGGTTAAATTGTTAGGTTGTTGTTTGGAGACAGAAGTCCCTCTACTTGT
TTATGAGTTTATCTCTAATGGAACCCTCTTCCATCATATCCATGAAAAAAGTGAAGAGTT
CCTAAGTTCATGGGATAATCGCTTAAGAATTGCTATAGAAGTTGCAGGCGCACTTGCTTA
CTTGCATTCAGCAGCTTCCATACCAATTTATCATAGAGACATCAAGTCTACCAACGTACT
CATAGATGGTAAATATACAGCTAAAGTGTCAGACTTTGGTATTTCAAGGTCTGTTCCAAT
TGATAAAACCCACCTCACCACATCAGTTCAAGGGACATTTGGTTACATGGATCCAGAGTA
TTTCCAATCAAACCAATTCACAGAGAAAA
>pac.W.VtB.201

CATTATAATGATAATAGAATTCTTGGCCAAGGAGGCCAAGGTACAGTGTACAAAGGCATG
CTTCAAGATGGAAGAATAGTAGCAGTTAAAAAGGCTACAATGGTTGATGAAGGCCAAGTT
GAACAGTTCATTAATGAGGTTGTCATTCTTTCTCAAATCAACCATAGGAATGTGGTTAAA
TTGTTAGGTTGTTGTTTGGAAACAGAAGTTCCTCTACTTGTTTATGAGTTCATCTCTAAT
GGAACTCTTTTTTACCACATTCATGACCCGACTGAAGAGTTTGTTATGTCATGGGATAAT
CGCTTAAGAATCGCTACAGAAGTTGCAGGTGCACTTGCTTACTTGCATTCAGCAGCATCT
ATTCCAATCTATCATAGAGATGTCAAGTCTACCAACATACTCTTAGATGATAAGCACAGA
TCCAAGGTTTCAGACTTTGGAACTGCAAGATCAGTCTCAATCGACAAAACTCATTTAACT
ACAATCGTGCAAGGGACCTTAGGGTACTTGGACCCAGAATACTTCCAAACAAGCCAATTC
ACAGATAAGAGTGATGTTTATAGCTTTGGAGTTGTTCTTGTGGAGCTCCTAACAGGAGAG
AAGCCAATTTCCTTAATCAACTCCCAAGAAAGGAGAAACTTAGTCACACATTTTATTCAA
TTAACAAAAGAGAACCGTATCTTTGAGATTCTTGACGATCAAGTTGTTGAGGGTCGAAAA
GAGGAGATTGAGGCAGTCGTTAACATTGCAAAGAGGTGCTTAAATTTTAATGGAAAG
>pac.W.VtB.203
AATTATAATGAGAACAGAATTCTTGGCCAAGGAGGTCAAGGTACAGTATACAAAGGTATG
CTCCATTGTGGAAGAATTGTAGCAATTAAAAAGGCTAAATTAGTTGATGAAGGCCAAGTC
GAGCAATTCATTAATGAGGTTGTCATACTTTCCCAGATCAACCATAGAAATGTGGTTAAA
TTGTTAGGTTGTTGTTTGGAGACAGAAGTTCCTCTACTAGTTTATGAGTTCATTTCCAAT
GGAACACTTTTTCACCACATCCATAGCCAAACTGAGGATTTCCTTATGTCATGGGATAAT
CGCTTAAGAATCATTACAGAAGTTGCAGGTGCACTTGCTTACTTGCATTCATCAGTTTCG
ATGCCAATCTATCATAGAGATGTCAAGTCCACCAATATACTCTTAGATGATAAGTATAGA
GCAAAAGTTTCAGACTTTGGAACTTCAAGGTCAGTCCCTGTCGACAAAACTCACTTAACT
ACACTAGTGCAAGGGACCATGGGGTACTTGGATCCGGAATACTTTCAAACGAGCCAATTC
ACAGATAAGAGTGATGTTTATAGCTTTGGAGTAGTTCTTGTAGAGCTTCTAACGGGAGAG
AAGCCAATATCTTTAATTGGCTCACAAGAAAAGAGAAACCTAGTCACATATTTTATTCAA
TCAACTAAGAAAAACCGTCTCTTTGAGATTCTTGATGCTCAAATTGTAGAGGGTCAAAAA
GAGGAGCTTGAGGTAGTTGCTGGCCTTGCAAAGAGGTGCGTGACCTTGAATGGAAAG
>pac.W.VtB.208
CATTATAATGAGAACAGAATTCTTGGCCAAGGAGGTCAAGGTACAGTATACAAAGGTATG
CTCCATTGTGGAAGAATTGTAGCAATTAAAAAGGCTAAATTAGTTGATGAAGGCCAAGTC
GAGCAATTCATTAATGAGGTTGTCATACTTTCCCAGATCAACCATAGAAATGTGGTTAAA
TTGTTAGGTTGTTGTTTGGAGACAGAAGTTCCTCTACTAGTTTATGAGTTCATTTCCAAT
GGAACACTTTTTCACCACATCCATAGCCAAACTGAGGATTTCCTTATGTCATGGGATAAT
CGCTTAAGAATCATTACAGAAGTTGCAGGTGCACTTGCTTACTTGCATTCATCCGCTTCC
ATGCCAATCTACCATAGAGATATCAAGTCTACCAATATACTCTTAGATGATAAGTGTAAA
GCCAAAGTTGCAGATTTTGGAACTTCCAGGTCAGTCTCTGTTGACAAAACTCATTTAACT
ACACTAGTGCAGGGGACCTTGGGGTACCTGGACCCAGAATACTTCCAATCAAGCCAATTC
ACAGATAAGAGTGATGTTTATAGCTTTGGAGTAGTCCTTGTGGAGCTTCTAACAGGAGAG
AAACCAATTTCAGTCTTCGCACAAGAAAGGAGGAACCTAGTCACATATTTTATTCAGTCA
ATAAAAGAAAACCTTCTCTTTGAGATTCTTGATGATCGAATTATAGAAGGCCGGAAAGAG
GAGATCGAGGCAGTTGCTTCCCTTGCAAAGAGGTGCTTGAACTTGAATGGAAGG
>pac.W.VtB.209
CACTTTAATGAGAACCGAATTCTTGGTCAAGGAGGCCAAGGTACAGTGTACAAAGGCATG
TTAGTAAATGGAAGAATTGTAGCAGTTAAGAGGTCCAAAATAGTGAATGAAGGCCAAATC
GAGCAGTTCATTAATGAGGTAGTCATACTTTCTCATATCAATCATAGGAATGTGGTTAAA
TTATTAGGTTGTTGTTTGGAGACAGAAGTTCCTCTACTTGTTTATGAGTTTGTCTCCAAT
GGAACCCCTTTTTCATCATATCCATGACCAAAGTGAGGAGTTCCTAAGTTCATGGGATAAT
CGCTTAAGAATTGCTGCAGAAGCTGCAGGTGCACTTGCATACTTGCATTCAACAGCTTCC
ATACCAATCTATCATAGAGACATCAAGTCTACCAACATACTCTTGGATAGTAAATATACA
GCTAAAGTTTCAGACTTTGGGATTTCAAGATCCGTCCCAATTGATAAAACTCACTTAACC
ACATTAGTTCAAGGGACCTTTGGGTACTTGGATCCAGAGTATTTTCAATCAAGCCAATTT
ACAGAGAAGAGTGATGTTTATAGCTTTGGAGTAGTTATTGTGGAGCTTTTAACAGGTGAG
AAGCCAATTTCTTCTGTTAAATCAAAAGAAAAGATGAATTTAGCCACACATTTTCTTTCT
TCTATAAGGGAAAACTGTCTATTTGAGATTCTTGATGCTCAAGTTGTCAAAGAGGATAGA
AAAGAGGAGATTGAGATTGTTGCTAATCTTGCAAAGAGATGTTTGAACTTGAATGGGAGG
>pac.W.Ch.152
CATGTTAGGAGATGGAAGAATTGTAGCAGTTAAGAAGTCCAAAATAGTTAATGAATGCCA
AATTGAGCAATTCATTAATGAGGTTTTCATACTTTCTCATATCAATCACAGGAATGTTGT
TAAATTGTTAGGTTGTTGTTTGGAAACAGAAGTTCCTCTACTCGTTTATGAGTTTATCTC
CAATGGAACCCTCTTTCATCATATTCATGACCAAAGTGAGGAGTTCCTAAGTTCATGGGA
CAATCGGTTAAGAATTGCTGCAGAAGTTGCAGGTACACTTGCCTACTTGCATTCATCAGC
TTCCATACCAATCTATCATAAAGACATTAAGTCTACAAACATACTCTTAGATGGTAAATA
TACAGCTAAAGTGGCAGACTTTGGGACCTCAAGATCAATCCCAAACGATAAAACTCACTT
AACCACATTAGTTCAAGGGACCTTCGGATACTTGGATCCGGAGTATTTTCAATCAAGCCA
ATTCACAGAGAAGAGTGATGTTT
>pac.W.Ch.156
AATTTTAGAGGACCATAGAATAGTGGCTGTTAAAAAATCCAAATTAGTGGATGCGAGCCA
AATTGAACAATTCATAAATGAGGTTGTTATTCTCTCCCAGATCAACCATAGGAATGTGGT
GAGGCTTCTAGGCTGTTGTTTGGAGACTGAAGTACCTTTACTAATTTATGAGTTCATCAG
CAACGGAACCCTCTTCCACCATATCCATGACGAGGGTCATGTGTCCTCAATTTCATGGGA

AATTCGTTTGAGGATAGCTACTGAAACTGCAGGAACACTTGCTTATTTGCATTCTGCAAC
TTCTACACCAATTATTAATAGGGATATTAAGTCTACTAACATACTCTTAGACGATAATTA
CACAGCTAAAGTAGCAGACTTTGGAGCTTCAAGGTTAGTTCCTTTAGATCAAACTCAATT
GACTACGTTAGTACAAGGGACATTGGGGTACTTGGACCCAGAATACTTTCTTTCAAGTCA
ATTAACAGAGAAGAGTGATGTTT
>pac.W.Ch.157
TATGTTAGTAGATGGAACAATTGTAGCAATTAAGAAGTCAAAAATAGTGGATGAAAGCCA
GATTGAGCAATTCATTAATGAGGTTGTCATGCTTTCCCAGATCAACCACAGGAATGTGGT
TAAATTATTAGGTTGTTGTTTGGAGGCAGAAGTTCCTCTACTTGTTTACGAGTTCATCTC
CAATGGAACCCTTTTCCATTATATTCATGACCAAAGTGAAAAGTTCCCAAATTCTTGGGA
CAATCGCTTAAAAATTGCTACAGATGTTGCAGCTGCACTTGCCTACTTACATTCAGCAGC
TTCGATACCAATCTACCATAGAGATATCAAGTCTTCCAACATACTCCTGGATGATAAATA
TATAGCTAAAATATCAGACTTTGGGGCTTCAAGGTCCATCCCAACAGATAAAACTCACTT
GACCACATTAGTTCAGGGGACTTTTGGGTACTTGGATCCAGAATATTTCCAATCAAGCCA
ATTTACAGAAAAAAGTGATGTTT
>pac.W.Ch.158
TATGCTACTAGATGGAAGAATTGTAGCAATTAAGAAATCCAAAATAGTGAATGAGGGTCA
AATCGAACAGTTCATTAATGAGGTTGTCATACTTTCTCATATCAACCACAAGAACGTGGT
TAAATTGTTGGGTTGTTGTTTGGAGACAGAAGTTCCTTTACTTGTTTATGAGTTCATCTC
CAATGGAACCCTTTTCCATCACATCCATGACCAAAGTGAGGAATTCCAATGTTCATGGGA
CAATCGGTTAAGAATTGCTATAGAACTTGCAAATGCACTTGCCTATTTGCATTCAGCAGC
TTCCATACCAATCTATCATAGAGATATCAAGTCTACCAACATACTATTAGATAGTAAATA
TACAGCTAAAGTATCAGACTTTGGGATTTCAAGGTCCGTTCCAAACGATAAAACTCACTT
AACCACATTAGTCCAAGGGACCTTTGGATACTTGGATCCAGAGTATTTCCAATCAAGCCA
ATTTACAGAAAAAAAGTG
>pac.W.Ch.160
TATGCTACTAGATGGAAGAATTGTAGCAATTAAGAAATCCAAAATAGTGAATGAGGGTCA
AATCGAACAGTTCATTAATGAGGTTGTCATACTTTCTCATATCAACCACAAGAACGTGGT
TAAATTGTTGGGTTGTTGTTTGGAGACAGAAGTTCCTTTACTTGTTTATGAGTTCATCTC
CAATGGAACCCTTTTCCATCACATCCATGACCAAAGTGAGGAATTCCAATGTTCATGGGA
CAATCGGTTAAGAATTGCTATAGAACTTGCAAATGCACTTGCCTATTTGCATTCAGCAGC
TTCCATACCAATCTATCATAGAGATATCAAGTCTACCAACATACTATTAGATAGTAAATA
TACAGCTAAAGTATCAGACTTTGGGATTTCAAGGTCCGTTCCAAACGATAAAACTCACTT
AACCACATTAGTCCAAGGGACCTTTGGATACTTGGATCCAGAGTATTTCCAATCAAGTCA
ATTCACAGAGAAAAGTGATGTTT
>pac.W.Ch.162
AGTGTTGTCAGATGGAAGAATAATAGCCGTCAAGAAGTCCAAGGATGCTAATCTAATTCG
TCTATTCGTAAATGAGGCTATCATTCTCTCCAAGATTAATCACACAAACGTGGTAAAACT
GATAGGCTATTGCAAAGAAACTGAAGTTCCTTTATTAGTTTACGAATTTGTTATAAATGG
AACCCTAGCCCACCATCTCCATGAATTGGGTCTTAGGTCATCGTTGGGTTGGGGAGACCG
CTTAAAGATTGCTGCAGAAGTTGCAAATGCAATTGCCTGTATGCATTCTTGTAAAATCAT
ACATAGAGACATAAAATCTACCAACATACTGTTGGATGAAAATTTCACGGCCAAATTGTG
TGACTTTGGACTTTCAAGGACAGTCCCATCTGATCAAGATCACGTATCAACCAAGGAGTT
GTGGGGGACGGAGGGGTACGTGGACCCTGACTATATTCAGACAGGCCAACTCACTGGTAA
GAGTGATGTTT
>pac.W.Ch.164
AATTTTAGCAGACCACAGAATAGTGGCTGTTAAAAAATCCAAAATAGTGGATGAGAGCCA
AATTGAACAATTCATAAATGAAGTTGTTATTCTCTCTCAGATCAACCATAGGAATGTGGT
GAGACTTCTAGGATGTTGTTTGGAGACTGAGGTTCCTCTACTAATTTATGAGTTTATCAA
CAATGGAACCCTCTTGCACCATATCCATGATGAGAGTCATATATCCTCAATTTCTTGGGG
ATGTCGTTTGAGGATAGCTACAGAAACTGCGGAAGCACTTGCTTATCTACATTCTGCAGC
TTCTCCACCAATCATTCACAGGGATATTAAGTCTACTAACATACTCTTGGATGAAGATTA
CACGGCTAAAATAGCAGACTTTGGAGCTTCAAGGTTGGTTCCTCTTGATTACACACAATT
GACTACATTAGTACAAGGGACGTTGGGGTACTTGGACCCAGAATATTTTCATTCAAGTCA
ATTAACTGAGAAAAGTGATGTTT
>pac.BRI.L.2
TGAAGCCaCCAATGGCTTCCACAACGACAGCCTGGTTGGCTCTGGTGGCTTTGGGGATGT
GTACAAAGCCCAATTGAAGGATGGAAGCACTGTGGCAATCAAAAAACTAATCCATGTTAG
TGGACAAGGTGACCGGGAATTCACAGCAGAAATGGAGACCATTGGGAAGATTAAGCACCG
GAACCTTGTCCCCCTATTGGGCTACTGCAAGGTTGGGGAAGAACGGCTCTTGGTGTATGA
ATACATGAGGTTCGGTAGCTTAGAAGACATCCTACATGATAGGAGAAAAGCAGGGATCAA
GCTGAATTGGGTTGCAAGGAGGAAAATCGCCATTGGCGCAGCGAGAGGATTAGCATTCCT
CCACCACAATTGCATCCCTCATATCATTCACAGGGACATGAAGTCAAGCAACGTCCTGCT
CGATGAGAACTTGGAAGCTCGGGTGTCAGATTTTGGAATGGCAAGGTTGATGAGTGCAAT
GGACACCCACCTGAGTGTCAGCACACTCGCCGGCACACCGGGTTATGTCCCACCGGAATA
CTACCAGAGCTTCCGGTGTTCTACCAAGGGTGATGTCTACAGCTATGGGGTAGTCCTTCT
CGAGCTGCTAACCGGCAAGCAGCCCACGGACTCGGCAGATTTTGGTGACAACAACCTAGT
GGGATGGGTGAAGCAGCATGCAAGGCTGAGAATAACTGATGTGTTCGATCCTGAGCTCAT
GAAAGAGGAGCCAAACTTGGAAATGGAGCTACTAGAACACCTGAAGATTGCATGTGCCTG
TTTGGATGATCGATCGTGGCGTCGACCCACCATGATTCAAGTAAT

>pac.BRI.L.6
GGAAGCTTACGTTCGCGGATCTTCTTGAAGCCaCCAATGGCTTCCACAATGACAGCCTCA
TCGGCTCCGGTGGGTTTGGGGATGTGTACAAAGCTCAACTAAAGGATGGAAGCATTGTAG
CCATCAAGAAATTAATCCATGTAAGTGGACAAGGTGACAGGGAATTCACAGCTGAAATGG
AAACCATTGGTAAGATTAAGCACAGGAACCTTGTCCCCCTCTTGGGCTACTGCAAGGTAA
GGGAAGAACGGCTGTTGGTTTATGAGTACATGAGATTTGGAAGCTTAGATGACATTTTAC
ATGATAAAAGAAAAGCTGGGATCAAACTGAATTGGGCAGCCAGGAGGAAAATTGCTATTG
GTGCTGCGAGGGGATTAGCTTTCCTCCACCACAACTGCACCCCCCATATAATTCACAGAG
ACATGAAGTCCAGCAATGTCCTGCTTGATGGTAACTTGGAAGCTCGGGTGTCTGATTTCG
GAATGGCGAGGCTGATGAGCGCAATGGACACCCACCTGAGTGTCAGCACACTTGCCGGTA
CCCCAGGTTACGTCCCACCGGAATACTACCAGAGCTTCCGGTGCTCTACTAAAGGGGACG
TTTACAGCTACGGGGTAGTCCTGCTAGAGCTGCTAACCGGTAAGCTGCCAACAGACTCGA
CTGACTTTGGTGACAACAACCTAGTGGGATGGGTGAAGCAGCACGCAAAGCTCAAAATAA
GCGATGTGTTCGACCCTGAGATCATGAAAGAGGACCCAAGCTTGGAGGTGGAACTCTTAC
AGCACTTAAAGATCGCCTGTGCGTGCTTGAGCGAACGACCGAGTCGACGACCCTCGATGA
TACAAGTAATGGCGATGTTTAAGGAAATTCAAGCAG
>pac.BRL.B.8
TGCTGACAGCTTGATAGGATCTGGTGGGTTCGGTGAGGTCTACAAGGCCCAGCTAAAGGA
CGGATGTGTGGTGGCAATTAAGAAGCTGATTCATGTCACAGGTCAGGGAGACAGAGAATT
TACTGCAGAAATGGAAACAATAGGGAAGGTCAAGCACAGAAACCTTGTTCCATTGTTGGG
TTACTGCAAAATAGGAGAGGAGAGACTTCTTGTGTACGAGTACATGAAATGGGGAAGCTT
GGAGACAGTTCTTCATGACAGGACCAACGGAGGGGGCTCGAAGCTCAACTGGGCAGCAAG
AAAGAAGATTGCTACTGGGTCGGCAAGAGGACTTGCATTTCTTCACCATAATTGCATTCC
TCACATAATCCACAGGGACATGAAGTCTAGCAATGTTCTCCTTGATGAAAATTTGGAGGC
CCGGGTGTCAGATTTTGGCATGGCAAGGCTGATGAATGCCGTTGACACACACCTCAGCGT
AAGCACCCTCGCTGGGACACCGGGTTATGTTCCACCTGAATACTACCAGAGTTTCAGGTG
CACGACAAAAGGGGATGTGTACAGCTACGGTGTCATACTGCTAGAGCTTGTCTCAGGCAA
GAGACCGATTGACCCATCAGAATTCGGT
>pac.BRL.B.9
TGCTGACAGCTTGATAGGATCTGGTGGATTCGGTGAGGTCTACAAGGCCCAGCTGAAGGA
TGGGTGTGTTGTTGCAATCAAGAGGCTGATTCATGTCACAGGTCAGGGGGACAGAGAATT
TACTGCAGAAATGGAAACAATCGGGAAGATCAAACACAGAAACCTTGTTCCATTGTTGGG
TTACTGCAAGATTGGAGAGGAGAGGCTTCTTGTGTACGAGTACATGAAATGGGGAAGCTT
GGAGACAGTTCTTCATGACAGGGCCAAGGGAAGGGGCTCGAAGCTCGACTGGGCAGCAAG
AAAGAAAATTGCTGTTGGGTCGGCAAGAGGACTTGCATTTCTTCACCATAGCTGCATTCC
TCACATAATCCACAGGGACATGAAGTCGAGCAATGTTCTCCTAGATGAAAATTTAGAGGC
CCGGGTGTCTGATTTTGGCATGGCGAGGCTAATGAATGCCCTTGACACTCATCTCAGCGT
AAGCACCCTAGCAGGGACACCAGGTTATGTTCCACCTGAATACTACCAGAGTTTCGGGTG
CACAACAAAAGGGGACGTGTACAGTTACGGGGTCATACTGCTGGAGCTACTCTCAGGTAA
GAGACCAATTGACCCATCAGAATTTGGT
>pac.BRL.B.12
TGCTGACAGCTTGATAGGATCTGGTGGGTTCGGTGAGGTCTACAAGGCCCAGCTGAAGGA
CGGATGTGTGGTGGCAATTAAGAAGCTGATTCATGTCACAGGGCAGGGAGACAGAGAATT
TACTGCAGAAATGGAAACAATAGGGAAGATCAAGCACAGAAACCTTGTTCCATTGTTGGG
TTACTGCAAAATAGGAGAGGAGAGACTTCTTGTGTACGAGTACATGAAATGGGGAAGCTT
GGAGACAGTTCTTCATGACAGGGCCAAGGGAAGGGGCTCGAAGCTCGACTGGGCAGCAAG
AAAGAAAATTGCTGTTGGGTCGGCAAGAGGACTTGCATTCCTTCACCATAGCTGCATTCC
TCACATAATCCACAGGGACATGAAGTCGAGCAATGTTCTCCTAGATGAAAATTTAGAGGC
CCGGGTGTCTGATTTTGGCATGGCGAGGCTAATGAATGCCCTTGACACTCATCTCAGCGT
AAGCACCCTAGCAGGGACACCAGGTTATGCTCCACCTGAATACTACCAGAGTTTCAGGTG
CACAACAAAAGGGGACGTGTACAGTTACGGGGACATACTGCTGGAGCTACTCTCAGGTAA
GAGACCAATTGACCCATCAGAATTTGGT
>pac.BRL.B.14
TGCTGACAGCTTGATAGGATCTGGTGGGTTCGGTGAGGTCTACAAGGCCCAGCTGAAGGA
CGGATGTGTGGTGGCAATTAAGAAGCTGATTCATGTCACAGGTCAGGGGGACAGAGAATT
TACTGCAGAAATGGAAACAATCGGGAAGATCAAACACAGAAACCTTGTTCCATTGTTGGG
TTACTGCAAGGTTGGAGAGGAGAGGCTTCTTGTGTACGAGTACATGAAATGGGGAAGCTT
GGAGACAGTTCTTCATGACAGGGCCAAGGGAAGGGGCTCGAAGCTCGACTGGGCAGCAAG
AAAGAAAATTGCTGTTGGGTCGGCAAGAGGACTTGCATTTCTTCACCATAGCTGCATTCC
TCACATAATCCACAGGGACATGAAGTCGAGCAATGTTCTCCTAGATGAAAATTTAGGGGC
CCGGGTGTCTGATTTTGGCATGGCGAGGCTAATGAATGCCCTTGACACTCATCTCAGCGT
AAGCACCCTAGCAGGGACACCAGGTTATGTTCCACCTGAATACTACCAGAGTTTCAGGTG
CACAACAAAAGGGGACGTGTACAGTTACGGGGTCATACTGCTGGAGCTACTCTCAGGTAA
GAGACCAATTGACCCATCAGAATTTGGT
>pac.BRL2.102
AGCAAGCATGATTGGGTGCGGTGGGTTTGGAGAAGTGTTCAAGGCCACATTAAAAGACGG
CTCAAGTGTCGCGATTAAGAAGCTCATACGCCTGAGTTGCCAGGGCGATCGAGAATTCAT
GGCCGAGATGGAAACTCTAGGGAAAATCAAGCACAGGAATCTTGTTCCTCTACTGGGCTA
TTGCAAGATTGGGGAGGAGAGGCTCCTCGTCTACGAATTCATGGAGTTTGGAAGTCTCGA
AGAGATGCTCCATGGGAGAACAAGGGCTCGGGAAGGAAGGCGGATACTAACATGGGAGGA

ACGGAAGAAGATTGCAAGAGGGGCAGCAAAAGGGCTCTGTTTCCTGCACCACAATTGCAT
CCCACACATCATACACCGTGACATGAAGTCAAGCAACGTGCTTTTGGACCATGATATGGA
AGCAAGAGTGTCAGACTTTGGGATGGCAAGGCTCATAAGTGCCCTGGACACGCACCTCAG
CGTGAGCACACTCGCAGGCACACCTGGGTATGTCCCACCTGAATACTACCAGAGCTTCCG
CTGCACTGCCAAGGGAGATGTTTACTCATTCGGAGTCGTACTCTTGGAGCTCTTGACGGG
AAAGAGGCCTACGGACAAGGAGGATTTTGGAGATACCAATTTGGTTGGGTGGGTGAAGAT
GACGGTGAGGGAAGGGAGGGGGAAGGAAGTAATCGACCCGGAGCTGCTTTCGGCCATCAA
GGGCGGTGATGAAGATGAAGCTGAAGAGTTCAAGGAGATGGTGAGATACTTGGAGATAAC
GATGCAGTGCGTGGAAGATTTTCCTTCTAGGAGGCCAAACATGTTGCAGGTG
>pac.BRL2.108
AGCAAGCATGATTGGGTGCGGTGGGTTTGGAGAAGTGTCCAAGGCCACATTAAAAGACGG
CTCAAGTGTCGCGATTAAGAAGCTCATACGCCTGAGTTGCCAGGGCGATCGAGAATTCAT
GGCCGAGATGGAAACTCTAGGGAAAATCAAGCACAGGAATCTTGTTCCTCTACTGGGCTA
TTGCAAGATTGGGGAGGAGAGGCTCCTCGTCTACGAATTCATGGAGTTTGGAAGTCTCGA
AGAGATGCTCCATGGGAGAACAAGGGCTCGGGAAGGAAGGCGGATACTAACATGGGAGGA
ACGGAAGAAGATTGCAAGAGGGGCAGCAAAAGGGCTCTGTTTCCTGCACCACAATTGCAT
CCCACACATCATACACCGTGACATGAAGTCAAGCAACGTGCTTTTGGACCATGATATGGA
AGCAAGAGTGTCAGACTTTGGGATGGCAAGACTCATAAGTGCCCTGGACACGCACCTCAG
CGTGAGCACACTCGCAGGCACACCTGGGTATGTCCCACCTGAATACTACCAGAGCTTCCG
CTGCACTGCCAAGGGAGATGTTTACTCATTCGGAGTCGTACTCTTGGAGCTCTTGACGGG
AAAGAGGCCTACGGACAAGGAGGATTTTGGAGATACCAATTTGGTTGGGTGGGTGAAGAT
GACGGTGAGGGAAGGGAGGGGGAAGGAAGTAATCGACCCGGAGCTGCTTTCGGCCATCAA
GGGCGGTGATGAAGATGAAGCTGAAGAGTTCAAGGAGATGGTGAGATACTTGGAGATAAC
GATGCAGTGCGTGGAAGATTTTCCTTCTAGGAGGCCAAACATGTTGCAGGTG

aa sequences

>pac.x.5.1
GVLSDRTIGAIKVFNLEFLAAFKSFDAECEVMRNICHRNLVKIISSCSNTEFRALILEYM
PNGSLEKWLYSYNYYLDFMQRLDIMLDVASALEYLHHDYSTAVVHCDLKPSNVLLDEDMV
GHVCDFGIAKLLPENESTKQTKTLGTL
>pac.x.5.2
GILHQDKLPVAVKVLNLQERGASKSFMAECEILREIRHRNLLKILASCSTIDFQGNDFKA
LVFELMPNGSLERWLHPSTDGQHLLNYLCLGKRLDIAIDIASALDYLHHHNQTPIVHCDL
KPSNILLDDSMAAHMGDFGLAKFLSKDTNFSDKNETSSIAIKGSI
>pac.x.5.3
GILPRDELPVAVKVLNLQECGASKSFMAECEALRAIRHRNLLKILTSCSGVDFEGNDFKA
LVFELMHNGSLENWLHPTIDGRHLLNNLRFGQRLNIAIDVASALDYLHHHCQTPIVHRDI
KPSNVLLDDDMTAHVGDFGLAKFLSNPTNNSRKFESSSVALRGSI
>pac.x.5.4
GLLNQGETIVAVKVLNLQQYGSYKSFMAECESLRNIQHRNLVKVITSCSSIDFEGNDFKA
LVYEFMSNGSLERWLHPNAEDAQVEQRNLNLLQRLNIAIDVACALDYLHHNSKTPIVHCD
LKPSNVLLDDDMVAHVGDFGLSRFLPMTINNSSRSQTSSMGI
>pac.x.5.6
GMVHQDGRLVSVAVKVLNLQQQEASKTFMAECKALRNVRHRNLLKIITSCSSADFKGNEF
KALVLEFMPNGSLESWLHPRIDGQLQLRYLSISQRLNIAIDVALALDYLHNHCQIPIAHC
DLKPSNVLLNDDMTAHVGDFGLAKFLYRASNNCGEGQHSSAALKGTI
>pac.x.5.8
GILCQDEPPVAVKVLNLQEQGASKSFMAECEALREIRHRNLLKILTCCSSVDFEGIDFKA
LVYELMPNGSLENWLHQSTNGQHLNDLNFGQRLNIAMDVALALDYLHHHCQTPIVHCDLK
PSNVLLDDNMVAHLGDFGLAKILSMTSNYSSENETISIVIKGSI
>pac.x.5.9
GILPRDELPVAVKVLNLQEYGASKSFMAECEALRAIRHRNLLKILTSCSGVDFEGNDFKA
LVFELMHNGSLENWLHPTIDGQHLLNNLRFGQRLNIAIDVASALDYLHHHCQTPIVHRDL
KPSNVLLDDDMTAHVGDFGLAKFLSNPTNNSRKFESSSVALRGSI
>pac.x.5.12
GILRQDEQPIAVKVVNLQEQGASKSFMAECEALREIRHRNLLKILTSCSSVDFQGNDFKA
LVFELMPNGSLENWLHPKPNEQNQLNKLNLRQRLNISIDVASALDYLHHQCQTPIVHCDL
KPSNVLLDDDMTAHVGDFGLAKFLSNPTNNSRKFESSSVALRGSI
>pac.x.5.13
GNLPRDDLPVAVKVLNLQECGASKSFMAECEALRAIRHRNLVKILTSCSSVDFEGNDFKA
LVFELMHNGSLENWLHPTLDGRHLLNNLRFGQRLNIAIDVASALDYLHHHCQTPIVHCDL
KPSNVLLDDDMTAHVGDFGLAKFLFNPTNNSRKNESSSVALRGSI
>pac.x.5.14
GLLNQGETNVAVKVFNLPQHGASKSFMAECESLRNIRHRNLVKVITSCSSIDFAGNDFKA
LVYEFMSNGSLERWLYPNAEVAQVEQRNLNILQRLNIAIDVASALDYLHHNCKTPIIHCD
LKPSNILLDDDMVAHVGDFGLSRFLPMTINNSQSQTSSIGIKGSI
>pac.x.5.17

GILHQNELPIAVKVFNLLEHGASKSFMTECEALREIRHRNLLKILTSCSSTDFEGNDFKA
LVFVLMPNGSLENWLHQSTYGRHQNYLNFDQRLNIAIDVASALDYLHHQCQTPIVHSDLK
PSNVLLDDDMVAHVSDFGLAKFISKPTKYSGNNKTSSVVIKGSI
>pac.x.5.18
GILPRDDLPVAVKVLNLQECGASKSFMAECEALRAIRHRNLVKILTSCSSVDFEGNDFKA
LVFELMHNGSLENWLHPTLDGQHLLNNLRFGQRLNIAIDVASALDYLHHHCQTLIVHCDL
KPSNVLLDDDMTAHVGDFGLAKFISKPTNYFGKNDTSSSIAIKGSI
>pac.x.5.19
GILPRDDLPVAVKVLNLQECGASKSFMAECEALRAIRHRNLVKILTSCSSVDFEGNDFKA
LVFELMHNGSLENWLHPTLDGQHLLNNLRFGQRLNIALDVASALDYLQYHCQTPIVHCDL
KPSNVLLDDDMTAHVGDFGLAKILSKPTSYTGKNETSSLAIKGSI
>pac.x.5.26
GLLNQGETNVAVKVFNLPQHGASKSFMAECESLRNIRHRNLVKVITSCSSIDFAGNDFKA
LVYEFMSNGSLERWLYPNAEVAQVEQRNLNILQRLNIAIDVASALDYLHHNCKTPIIHCD
LKPSNILLDDDMVAHVGDFGLSRFLPMTINNSSRSQTSSIGIKGSI
>pac.x.5.39
ASLGGEKRILAIKKLVTSNIIQYPEDFDREVRILGKAMHPNLMTLKGYYWTPQLQLLISD
YAPNGSLHSKLHERSPSTPSLSWANRFKIVLGTAKGLAHLHQSFRPPIIHYNVKPSNILL
DENYNPKISDFGLARLLPKLDKHVINSRFQSAL
>pac.x.5.43
ASLGGEKRILAIKKLVTSNIIQYPEDFDREVRILGKAMHPNLMTLKGYYWTPQLQLLISD
YAPNGSLHSKLHERSPSAPSLSWANRFKIVLGTAKGLAHLHQSFRPPIIHYNVKPSNILL
DENYNPKISDFGLARLLPKLDKHVINSRFQSAL
>pac.x.6.102
YKGILHQDKLPVAVKVLNLQERGASKSFMAECEILREIRHRNLLKILASCSTIDFQGNDF
KALVFELMPNGSLERWLHPSTDGQHLLNYLCLGKRLDMAIDIASALDYLHHHNQTPIVHC
DLKPSNILLDDSMTAHMGDFGLAKFLSKDTNFSDKNETSSIAIKGSI
>pac.x.6.103
YKGILHRDESPIAVKVFNLLEHGASKSFMTECEALREIRHRNLLKILTSCSSTDFEGNDF
KALVFVLVPNGSLENWLHQSTYGRHQNYLNFDQRLNIAIDVASALDYLHHQCQTPIVHSD
LKPSNVLLDDGMVAHVSDFGLAKFISKPTKYSGNNKTSSVVIKGSI
>pac.x.6.104
YKGMVHQDGRLVSVAVKVLNLQQQEASKTFMAECKALRNVRHRNLLKIITSCSSADFKGN
EFKALVLEFMPNGSLESWLHPRIDGQLQLRYLSISQRLNIAIDVALALDYLHNHCQIPIA
HCDLKPSNVLLNDDMTAHVGDFGLAKFLSRATSNSGEGQHSSVAIKGTI
>pac.x.6.107
YKGIIHYDGKPAPVAVKVLNLQQRGASKTFMVECNALRKIRHRNLLKIITSCSSVDFKGN
DFKALVFEYMPSGSLESWLHPSIDEKHQSRYLNFSQRLDIAIDAAFALDYLKNHCPTPIV
HCDIKPSNVLLDDDMTAHVGDFGLAKTLSRASNNCGEGQHSSAALKGTI
>pac.x.6.108
YKGILCQDEQPVAIKVLILQERGASKSFMAKCEALREIQHRNLLKIFTSCSTVDFEGNFF
RAFVFEFLPNGSLENWLHPSTDGQHLLKNLSFSQRLNIAIDVAFTLDYLHHHCQTPIVHC
DLKPSNILLDDDMTTHVGDFGLAKFLSKPTNYSRKDETSSVAIKGSI
>pac.x.6.109
YKGIFHQDGRAILIAVKVLNLLQRKASKTFMAECKALRNIRHRNLLKILTSCSSIDFKGN
DFKALVFEFMPNRSLESWLHPSVNEHHQLRCLNFSQRLNVAIDVAFALDYLQNHCPTPIV
HCDLKPSNVLFDDDMTAHVGDFGLAKFLSMATDNSGESQYSSIAIKGTI
>pac.x.6.112
YKGMVHQDGRLVSVAVKVLNLQQQEASKTFMAECKALRNVRHRNLLKIITSCSSADFKGN
EFKALVLEFMPNGSLESWLHPRIDGQLQLRYLSISQRLNIAIDVALALDYLHNHCQIPIA
HCDLKPSNVLLNDDMTAHVGDFGLAKFLYRATSNSAEGQNSLATIKGTI
>pac.x.6.115
YKGILHQDGRSILVAVKVLNLQQRDGFKSFMTECKALRNIRHRNLVKILTSCSSIDFKDN
DFKALVFEFMPNGSLESWLHPSTEGHHQSRYLNFSQRLNIAIDVAFALDYLHNHCSTPIV
HCDLKPSNVLLDDDMTAHVGDFGLAKILSRATSNSGEGQHSSAALKGTI
>pac.x.6.123
YKGILHQDELFVAVKEFNLQEWGASKSFMAECEALREIRHQNLLKILTACSSIDIEGNDL
KALVSKLMPNGSLENWLHPNTYGGHLLNNLSFGKRLNIAIDVASALDYLHHHCQTPIVHR
DLKPSNVLLDDDMIAHVGDFGLAKFLSKLDNYSGKNETSSIAIKGSI
>pac.x.6.124
YKGILHQDKLPVAVKVLNLQERGASKSFMAECEILREIRHRNLLKILASCSTIDFQGNDF
KALVFELMPNGSLERWLHPSTDGQHLLNYLCLGKRLDIAIDIASALDYLHHHNQTPIVHC
DLKPSNILLDDSMTAHMGDFGLAKFLSKDTNFSDKNETSSIAIKGSI
>pac.x.6.129
YKGIFHQDGRAILIAVKVLNLLQRKASKTFMAECKALRNIRHRNLLKILTSCSSIDFKGN
DFKALVFEFMPNGSLESWLHPSVNEHHQLRCLNFSQRLNVAIDVAFALDYLQNHCPTPIV
HCDLKPSNVLFDDDMTAHVGDFGLAKFLSMATDNSGESQYSSIAIKGTI
>pac.x.7.4
GLLSDGTVSAVKVFNSEFQGVFKSFNVECEVYRNIRHRNLIKIISSCSNIEFRALILEYM
PNGSLEKWLYSQNYCLNVLQRLDIMIDVAFALEYLHHGYSTLVVHCDLKPTNILLDEDMV

GHVGDFGIAKLFAENASIVQTETLGTL
>pac.x6.52
KGTLSNNTEVAVKCVSHDSEQGLREFMAEISSMGRLQHKNLVHMKGWCRKGNELMLVYNY
MSNGSLNRWIFDEPKKLLGWKERRRVLVDVAEGLNYLHHGWDQVVLHRDIKSSNILLDSE
FRGRLGDFGLAKLYQHGKEPGTTRLVGTLGYLAPELVNVGATAA
>pac.x6.53
KGTLSNNTEVAVKCVSHDSEQGLREFMAEISSMGRLQHKNLVHMKGWCRKGNELMLVYNY
MSNGSLNRWIFDEPKKLLGWKERRRVLVDVAEGLDYLHHGWDQVVLHRDIKSSNILLDSE
FRGRLGGFGLAKLYQHGKEPGTTRLVGTLGYLAPELVNVGATAA
>pac.x6.54
KGTLSNNTEVAVKCVSHDSEQGLREFMAEISSMGRLQHKNLVHMKGWCRKGNELMLVYDY
MSNGSLNRWIFDEPKKLLGWKERRRVLVDVAEGLNYLHHGWDQVVLHRDIKSSNILLDSE
FRGRLGDFGLAKLYQHGKEPGTTRLVGTLGYLAPELVNVGATAA
>pac.x6.55
KGTLSNNTEVAVKCVSHDSEQGLREFMAEISSMGRLQHKNLVHMKGWCRKGNELMLVYDY
MSNGSLNRWIFDEPKKLLGWKERRRVLVDVAEGLNYLHHGWDQVVLHRDIKSSNILLDSE
FRGRLGDFGLAKLYQHGKEPGTTRLVGTLGYLAPELVNVGATAA
>pac.pt.4.7
VLKNKMTVAVKRSDPGGDQGLLEFQREIIILSSIRHRHLVSLIGYCDERSEMILVYEFME
KGTLRSHLYNSDLPSLSWKQRLDICIGSARGLHYLHTGVAGGIIHRDIKSTNILLDENYV
AKVADFGLSREVPVEHSRGGYVSTAVKGTFGYLDPEYFKRQQLTEKS
>pac.pt.4.8
VLRDNTKIAVKRCVPGSKQGLPEFQTEITVLSKIRHRHLVALVGYCEEQSEMILVYEYME
KGPLRNHLYGSELPPLSWKQRLEICIGSARGLHYLHTGSAQGIIHRDVKSTNILLDETYV
AKVADFGLSRSGPCMDETHVSTGVKGSFGYLDPEYFKRQQLTDKS
>pac.pt.4.11
TLEDGTKVAVKRGNPRSEQGLAEFRTEIEMLSKLRHRHLVSLIGYCDERSEMILVYEYMA
NGTLRSHLYGTDLPSLSWRQRLEICIGVARGLHYLHTEAAQSIIHRDVKSANILLNENFV
AKVADFGISKIVPALDQTHVSTVVKGTFGYLDPEYFRRQQLTEKS
>pac.pt.4.13
TLEDGTKVAVKRGNPRSGQGLTEFQTEIELLSKLRHRHLVSLIGYCNEGSEMILVYEYMA
NGTLRRHLCGADLPSLSWRQRLEICIGAAKGLHYLHTGAAQSIIHRDVKSANILLNENFV
AKVADFGISKIVPALDQTHVSTVVKGSFGYLDPEYFRRKQLTEKS
>pac.pt.4.14
VLRDNTKIAVKRCVPGSEQGVPEFQTEITVLSKIRHQHLVALIGYCEKQSEMILVYEYME
KGPLRNILYGSELPPLSWKQSLEICIGSARGLHYLHTGSAQGIIHRDVKSTNILLDENYV
TKVADFGLSRSGPCVDETHVSTGVKGSFGYLDPEYFKRQQLTDKS
>pac.pt.5.102
VLKNKMTVAVKRSDPGGDQGLLEFQREIIILSSIRHRHLVSLIGYCDERSEMILVYEFME
KGTLRSHLYNSDLPSLSWKQRLDICIGSARGLHYLHTGVAGGIIHRDIKSTNILLDENYV
AKVADFGLSREVPVEHSRGGYVSTAVKGTFGYLDPEYFRHQRLTEKSDVYSFGVVLL
>pac.pt.5.103
VLGDGTKVAVKRGNPLSQQGLAEFRTEIEMLSQFRHRHLVSLIGYCDERKEMILVYEYME
NGTLKSHLYGSNLPSLSWKQRLEICIGSARGLHYLHTGSTKAIIHRDVKSANILLDENLM
AKVADFGLSKTGPEIDQTHVSTAVKGSFGYLDPEYFRRQQLTEKSDVYSFGVVLF
>pac.pt.5.104
EIDDGTLVAIKRAHPQSQQGLREFETEIEMLSKLRHRHLVSMIGFCEEQNEMILVYEYMA
NGTLRSHLFGSDLPPLTWKQRLDVCIGAARGLHYLHTGAERGIIHRDVKTTNILLDENFV
AKMADFGLSKTGPALEHTHVSTAVKGSFGYLDPEYFRKQHLTEKSDVYSFGVVLF
>pac.pt.5.106
VLKNKMTVAVKRSDPGGDQGLLEFQREIIILSSIRHRHLVSLIGYCDERSEMILVYEFME
KGTLRSHLYNSDLPSLSWKQRLDICIGSARGLHYLHTGVAGGIIHRDIKSTNILLDENYV
AKVADFGLSREVPVEHSRGGYVSTAVKGTFGYLDPEYFRHQRLTEKSDVYSFGVVLL
>pac.pt.5.107
VMEDGTKVAVKRSNPLSQQGLAEFRTEIEMLSQFRHRHLVSLIGYCDEQKEMILVYEYME
NGTLNSHLYGSNFPSLSWKQRLEICIGSARGLHYLHTGFPKDIIHRDVKSANILLDENLM
AKVADFGLSKTGPGIDQTHVSTAVKGSFGYLDPEYFIMQRLTEKSDVYSFGVLLF
>pac.pt.5.110
KRSEPGRDQGLLEFQREIIILSNIRHRHLVSLIGFCDEQSEMILVCEFMEKGTLRSHLYN
SDLPSLSWKQRLDICIGSARGLHYLHTGVAGGIIHRDIKSTNILLDENYVAKVADFGLSR
EVPVEHSRGGYVSTAVKGTFGYLDPEYFRHQRLTEKSDVYSFGVVLL
>pac.pt.5.113
VLKNKMTVAVKRSDPGGDQGLLEFQREIIILSSIRHRHLVSLIGYCDERSEMILVYEFME
KGTLRSHLYNSDLPSLSWKQRLDICIGSARGLHYLHTGVAGGIIHRDIKSTNILLDENYV
AKVADFGLSREVPVEHSRGGYVSTAVKGTFGYLDPEYFRHQRLTEKSDVYSFGVVLL
>pac.pt.2.202
KGEIDDGTLVAIKRAHPQSQQGLREFETEIEMLSKLRHRHLVSMIGFCEEQNEMILVYEY
MANGTLRSHLFGGDLPPLTWKRRLDVCIGAARGLHYLHTGAERSTIHRDVKTTNILLDEN
FVAKMADFGLSKTGPSLDHTHVSTAVKGSFGYLDPEYFRRQQLTEKSDVYSFGVVLF
>pac.pt.2.203

KGVLRDGTKVAVKRGNPLSQQGFAEFWTEIEMLSQLRHRHLVSMIGYCDEQKEMILVYEY
MENGTLRNHLYGSELPILSWKQRLEICIESARGLHYLHTGSTKAIIHRDVKSANILLDEN
LMAKVADFGLSKAGPEIDQTHVSTAVKGSFGYLDPEYFRRQQLTEKSDVYSFGVVLF
>pac.pt.2.205
KGEIDDGTLVAIKRAHPQSQQGLREFETEIEMLSKLRHRHLVSMIGFCEEQNEMILVYEY
MANGTLRSHLFGNDLPPLTWKQRLDVCIGAARGLHYLHTGAERGIIHRDVKTTNILLDEN
FVAKMADSGLSKTGPALEHTHVSTAVKGSFGYLDPEYFRKQHLTEKSDVYSFGVVLF
>pac.pt.2.207
RGEIDDGTLAAIKRAHPQSEQGLAEFETEIEMLSKLRHRHLVSMIGFCEEQNEMILVYEY
MANGTLRSHLFGGDLPPLTWKRRLDVCIGAARGLHYLHTGAERSTIHRDVKTTNILLDEN
FVAKMADFGLSKTGPALEHTHVSTAVKGSFGYLDPEYFRKQHLTEKSDVYSFGVVLF
>pac.pt.2.209
KGVMEDGTKVAVKRSNPLSQQGLAEFRTEIEMLSQFRHRHLVSLIGYCDEQKEMILVYEY
MENGTLNSHLYGSNFPSLSWKQRLEICIGSARGLHYLHTGFPKDIIHRDVKSANILLDEN
LMAKVADFGLSKTGPGIDQTHVSTAVKGSFGYPDPEYFIMQRLTEKSDVYSFGVLLL
>pac.pt.2.210
RGEIDDGTLAAIKRAHPQSEQGLAEFETEIEMLSKLRHRHLVSMIGFCEEQNEMILVYEY
MANGTLRSHLFGGDLPPLTWRRRLDVCIGAARGLHYLHTGAERSTIHRDVKTTNILLDEN
FVAKMADFGLSKTGPSLDHTHVSTAVKGSFGYLDPEYFRRQQLTEKSDVYSFGVVLF
>pac.pt.10.301
LGELEDGTKLAIKRGNPRSEQGINEFQTEIQMLSKLRHRHLVSLIGYCDEQSEMILVYEY
MANGPLRDHIYGSTLPTLSWKQRLEICIGAARGLHYLHTGAAQGIIHRDVKTTNILLDEN
LVAKVSDFGLSKTAPTLEQTHVSTAVKGSFGYLDPEYFRRQQLTEKSDVYSFGVVLF
>pac.pt.10.306
KGEIDGASTKVAIKRGNPLSEQGVHEFQTEIEMLSKLRHRHLVSLIGYCEENCEMILVYD
YMAHGTLREHLYKTQKPPLPWRQRLEICIGAARGLHYLHTGAKHTIIHRDVKTTNILLDE
KWVAKVSDFGLSKTGPTLDHTHVSTVVKGSFGYLDPEYFRRQQLTDKSDVYSFGVVLF
>pac.pt.10.309
LGELEDGTKLAIKRGNPRSEQGINEFQTEIQMLSKLRHRHLVSLIGYCDEQSEMILVYEY
MANGPLRDHIYGSTLPTLSWKQRLEICIGAARGLHYLHTGAAQGIIHRDVKTTNILLDEN
LVAKVSDFGLSKTAPTLEQTHVSTAVKGSFGYLDPEYFRRQQLTEKSDVYSFGVVLF
>pac.pt.10.310
LGELEDGTKLAIKRGNPRSEQGINEFQTEIQMLSKLRHRHLVSLIGYCDEQSEMILVYEY
MANGPLRDHIYGSTLPTLSWKQRLEICIGAARGLHYLHTGAAQGIIHRDVKTTNILLDEN
LVAKVSDFGLSKTAPTLEQTHVSTAVKGSFGYLDPEYFRRQQLTEKSDVYSFGVVLF
>pac.ptd.5.2
VLKNKMTVAVKRSDPGGDQGLLEFQREIIILSSIRHRHLVSLIGYCDERSEMILVYEFME
KGTLRSHLYNSDLPSLSWKQRLDICIGSARGLHYLHTGVAGGIIHRDIKSTNILLDENYV
AKVADFGLSREVPVEHSRGGYVSTAVKGTFGYLDPEYFRHQRLTEKSDVYSFGVVLL
>pac.ptd.5.5
VLKNKMTVAVKRSDPGGDQGLLEFQREIIILSSIRHRHLVSLIGYCDERSEMILVYEFME
KGTLRSHLYNSDLPSLSWKQRLDICIGSARGLHYLHTGVAGGIIHRDIKSTNILLDENYV
AKVADFGLSREVPVEHSRGGYVSTAVKGTFGYLDPEYFRHQRLTEKSDVYSFGVVLL
>pac.ptd.8.21
KGVLKNKTTVAVKRSDPGGDQGLLEFQREIIILSSIRHRHLVPLIGYCDERSEMILVYEF
MEKGTLRSHLYNSDLPSLSWKQRLDICIGSARGLHYLHTGVAGGIIHRDIKSTNILLDEN
YVAKVADFGLSREVPVEHSRGGYVSTAVKGTFGYLDPEYFRHQRLTEKSDVYSFGVVLL
>pac.ptd.8.22
RGEIDDGTLAAIKRAHPQSEQGLAEFETEIEMLSKLRHRHLVSMIGFCEEQNEMILVYEY
MANGTLRSHLFGGDLPPLTWRRRLDVCIGAARGLHYLHTGAERSTIHRDVKTTNILLDEN
FVAKMADFGLSKTGPSLDHTHVSTTVKGSFGYLDPEYFRRQQLTEKSDVYSFGVVLF
>pac.ptd.8.23
KGVMEDGTKVAVKRSNPLSQQGLAEFRTEIEMLSQFRHRHLVSLIGYCDEQKEMILVYEY
MENGTLNSHLYGSNFPSLSWKQRLEICIGSARGLHYLHTGFPKAIIHRDVKSANILLDEN
LMAKVADFGLSKTGPGIDQTHVSTAVKGSFGYLDPEYFIMQRLTEKSDVYSFGVLLF
>pac.ptd.9.41
KGVVDGGLKVAIKRANPSSEQGVHEFQTEIEMLSKLRHRHLVSLIGYCEENEEMILVYDY
MAYGTLREHLYKSNKPTLSWKQRLEICIGAARGLHYLHTGAKYTIIHRDVKTTNILLDEN
WVAKVSDFGLSKTGPNLNQTHVSTVVKGSFGYLDPEYFRRQQLTEKSDVYSFGVVLF
>pac.Erf.2
QLRSGRHVAVKMLGKSKGNGQEFINEVATIGRIHHVNVVRLIGFCSNGSKRALIYEFMPN
GSLEKYTFSQEKEERLSLSWDKMYEIALGVARGIEYLHRGCDIQILHFDIKPHNILLDEN
FVPKISDFGLAKFYPINDNTITLTAARGT
>pac.Erf.3
TLDHGETIVAVKVLNLQQQGASKSFMAECEALRNIRHRNLVKILTICSSMDFKGNDFKAL
VFEFMPNGNLERWLHPETYHTQDELGNLNFLQRLNIAIDVASALEYLHHNCPTPIIHCDL
KPSNILLDNDMTAHVGDFGLSRLHLETSNRSSQNQTSSIGIRGS
>pac.Erf.4
NSSSRWKTSNICSEGVERSTTRSIQDFYGRMQSIEKYSTSKSSQDHNFLLSVDFMGNDFK
ALIFEFMTNGNLESWLHPNIDGQHQSRYLNISQRLDMAMDVASALNYLHNQCPTPIVHCD

LKPSNVLLDDDMSAHVGDFGLAKFLFRAPSDSGESQYSSVAIKGT
>pac.Erf.5
ILGNGETIVAVKVLNLQQQGASKSFMAECEALRNIRHRNLVKILTTCSSTDFEGNDFKAL
VFEFMPNGSLEKWLHPGADDAQDGLMNINLLQRLNIAIDVASALEYLHHHCQIPIIHCDL
KPNNILLDNDMTAHVGDFGLSRFLPEATNAFSQNHSSSIGIRGS
>pac.Erf.6
LLNQGETNVAVKVFNLPQHGASKSFMAECESLRNIRHRNLVKVITSCSSIDFAGNDFKAL
VYEFMSNGSLERWLYPNAEVAQVEQRNLNILQRLNTAIDVASALDYLHHNCKTPIIHCDL
KPNNILLDDDMVAHVGDFGLSRFLPMTINNSQSQTSSIGIKGS
>pac.Erf.7
IFHQDGRSVSVAVKVLNLQQREASKTFMAECKALRNIRHRNLLKILTSCSSVDFKGNDFK
ALVLEFMSNESLESWLHPNIDEHHQSRYLNFSQRLNIAIDVALALDYLHNNCPTPIVHCD
LKPSNVLLDDEMTAHVGDFGLAKFLSRATSNSGEGQHSSVAIKGT
>pac.Erf.9
QLRSGHHVAVKMLGKSKGNGQEFINEVATIGRIHHVNVVQLIGFCSNGSKRALIYEFMPN
GSLEKYTFSQEKEERLSLGWDKMYEIALGVARGIEYLHRGCDIQILHFDIKPHNILLDEN
FVPKISDFGLAKFYPINDNTITLTAARGT
>pac.Erf.10
ILGNGETIVAVKVLNLQQQGASKSFMAECEALRNIRHRNLVKILTTCSSTDFEGNDFKAL
VFEFMPNGSLEKWLHPGADDAQDGLMNINLLQRLNIAIDVASALEYLHHHCQIPIIHCDL
KPNNILLDNDMTAHVGDFGLSRFLPEATNAFSQNHSSSIGIRGS
>pac.Erf.11
MVHQDGRLVSVAVKVLNLQQQEASKTFMAECKALRNVRHRNLLKIITSCSSADFKGNEFK
ALVLEFMPNGSLESWLHPRIDGQLQLRYLSISQRLNIAIDVALALDYLHNHCQIPIAHCD
LKPSNVLLNDDTTAHVGDFGLAKFLYRATSNSAEGQNSLATIKGT
>pac.Erf.13
RLVDGRLVAVKVLNESKGKGDDFINEVTTIGRTNHHNIVRLLGFCSEGSKRALIYEFMSN
GSLEKFIYVDKPRMTQPLAWENLYQIAIGIAKGLEYLHRGCNTHILHLDIKPHNILLDEK
FCPKISDFGLAKQCLTGESAVSLHGARGT
>pac.Erf.14
VLREDEAVVAVKVLNLQQHGASKSFISECKTLTNIRHRNLVKILTACSSVDFRMNDFKAL
VFEFMSNGNLDEWLHPRIGGQHQLKSLSLIQRLNIAIDVASALEYLHFHCQIPIVHCDLK
PSNVLLDEDMTAHVGDFGLARFLFETSNISSKNQTISAGLKGS
>pac.Erf.2B.210
SLIKWPQRVVAVKRLENVVNGGEREFQNEMTSIGRTYHRNLVRLLGYCQDGHQRLLVYEY
MSKGSLADFLFKPDCPPSWDERVRIALDIARGILYLHEECETQIIHCDIKPQNILMDEYQ
CAKIADFGLAKLLKPDQTRTFTGIRGTRGYVAPEWHRNLPITVK
>pac.Erf.2B.211
ILSSSQKVVAVKRLENMVKEGENEFQNEMTTIGRTHHRNLVRLLGYCHEGTNRLLVYEYM
SNGSLADFLFKYERHPNWDERVGIALHIARGILYLHEECETQIIHCDIKPQNILIDEYRC
AKIADFGLAKLLKPNQSMTSTGIRGTRGYVAPEWHQNMPITVK
>pac.W.ArA.3
YAENQILGRGGYGTVYKGILADHRTVAVKKSKIVDQSQIEQFINEVVILSQINHRNVVRL
LGCCLETEVPLLIYEFINNGTLSHHIHDESHISSISWGCRLRIATETAEALAYLHSAASP
PIIHRDIKSTNILLDEDYTAKIADFGASRLVPLDYTQLTTLVQGTLGYLDPEYFHSSQLT
>pac.W.ArA.4
FSEKNLIGGGGFGVVYKGTLSDGTWVAVKRIIESDFHGDAEFCNEVEILSNLKHRNLVLL
RGCCMTDEESSKQRYLVYEYMPNGNLKDHLFLLDKQPLSWPQRKSIILDVAKGIAYLHYG
VKPAIYHRDIKPTNILLDADMKARVADFGLVKQSREGQSQLITRVAGTHGYLAPEYALYG
QLT
>pac.W.ArA.5
FSQKNLIGRGSFGVVYKGILPDGTWVAVKKITESDFQGDAEFCNEVEIISNLKHRNLVPL
RGCCVIDGDCDEGSGKQRYLVYEHMPYGNLEDHLFLSNPHSGIFNKQPLSWPQRKSIILD
VAKGIAYLHYGVKPAIYHRDIKPTNILLDADMKARVADFGLVKQSREGQSHLITKVAGTH
GYLAPEYALYGQLT
>pac.W.ArA.6
YAENQILGRGGYGTVYKGILADHRTVAVKKSKIVDQSQIEQFINEVVILSQINHRNVVRL
LGCCLETEVPLLIYEFINNGTLSHHIHDESHISSISWGCRLRIATETAEALAYLHSAASP
PIIHRDIKSTNILLDEDYTAKIADFGASRLVPLDHTQLTTLVQGTLGYLDPEYFHSSQLT
>pac.W.ArA.7
YAENRILGRGGYGTVYKGILADHRIVAVKKSKIVDESQIEQFINEVVILSQINHRNVVRL
LGCCLETEVPLLIYEFINNGTLFHHIHDEGHVSSISWGSRMRIAAETAGVLAYLHSAASI
PIIHRDIKSTNILLDEKYTAKVADFGASRLVPLDQTQLTTLVQGTLGYLDPEYFNSSQLT
>pac.W.ArA.8
YNENRILGQGSQGTVYKGMLPDGRIVAIKKSKLVNEGQIEQFINELAILSHINHRNVVKL
LGCCLETEVPLLVYEFISNGTLFYHIHEKSEEFLSSWDNRLRIATEVAGALAYLHSAASI
PIYHRDIKSTNILIDGKYTAKVSDFGISRSVPIDKTHLTTSVQGTFGYMDPEYFQSNQFT
>pac.W.ArA.9
YNENRILGQGGQGTVYKGMLPDGRIVAIKKSKIVNEGQIEQFINEVVILSHINHKNVVKL
LGCCLETEVPLLVYEFISNGTLFHHIHVQSEEFQCSWDNRLRIAIELANALAYLHSAASI

PIYHRDIKSTNILLDSKYTAKVSDFGISRSVPNDKTHLTTLVQGTFGYLDPEYFQSSQFT
>pac.W.ArA.11
YAENQILGRGGYGTVYKGILADHRTVAVKKSKIVDQSQIEQFINGVVILSQINHRNVVRL
LGCCLETEVPLLIYEFINNGTLSHHIHDESHISSISWGCRLRIATETAEALAYLHSAASP
PIIHRDIKSTNILLDEDYTAKIADFGASRLVPLDHTQLTTLVQGTLGYLDPEYFHSSQLT
>pac.W.ArA.12
FSQKNLIGRGSFGVVYKGILPDGTWVAVKKITESDFQGDAEFCNEVEIISNLKHRNLVPL
RGCCVIDGDCDEGSGKQRYLVYEHMPYGNLEDHLFLSNPRSGIFNKQPLSWPQRKSIILD
VAKGIAYLHYGVKPAIYHRDIKPTNILLDADMKARVADFGLVKQSREGQSHLITKVAGTH
GYLAPEYALYGQLT
>pac.W.ArA.14
YAENRILGRGGYGTVYKGILADHRIVAVKKSKIVDENQIEQFINEVVILSQINHRNVVRL
LGCCLETEVPLLIYEFINNGTLFHHIHDEGHVSSISWEIRLRIAAETAGALAYLHSAVST
PIIHRDIKSANILLDENYTSKIADFGASRLVPLDQTQLTTLVQGTLGYLDPEYFHSSQLT
>pac.W.ArB.305
NGFSEKQRLGTGAYGTVYAGKLHNVDWVAIKKIRHRDNDGIEQVMNEIKLLSSVSHPNLV
RLLGCCIEKGEQILVYEFMPNGTLSQHLQREISTGLPWMTRLTIATETAHAIAHLHSAIN
PPIFHRDIKSSNILLDYNYNSKVADFGLSRLGMTESSHISTAPQGTPGYLDPQYHQNFHL
SDKX
>pac.W.ArB.312
NGFSEKQRLGTGAYGTVYAGKLHNVDWVAIKKIRHRDNDGIEQVMNEIKLLSSVSHPNLV
RLLGCCIEKGEQILVYEFMPNGTLSQHLQREISTGLPWMTRLTIATETAHAIAHLHSAIN
PPIFHRDIKSSNILLDYNYNSKVADFGLSRLGMTESSHISTAPQGTPGYLDPQYHQNFHL
SDKX
>pac.W.VtA.101
TVYKGMLVDGMIVAIKKSKIVDEGQIEQFIYEVVMLSQINHRNVVKLLGCCLETEVPLLV
YEFISKGTLFHYIHDQSEEFPNSWDNRLKIATDVATALAYLHSAASMPIYHRDIKSSNIL
LDDKYIAKISDFGISRSIPTDKTHLTTLVQGTLGYLDPEYYQSSQFTEKX
>pac.W.VtA.102
TVYKGMLVDGTIVAIKKSKIVDESQIEQFINEVVMLSQINHRNVVKLLGCCLEAEVPLLV
YEFISNGTLFHYIHDQSEKFPNSWDNRLKIATDVAAALAYLHSAASIPIYHRDIKSSNIL
LDDKYIAKISDFGASRSIPTDKTHLTTLVQGTFGYLDPEYFQSNQFTEIX
>pac.W.VtA.105
TVYKGMLVDGMIVAIKKSKIVDEGQIEQFIYEVVMLSQINHRNVVKLLGCCLETEVPLLV
YEFISKGTLFHYIHDQSEEFPNSWDNRLKIATDVATALAYLHSAASMPISHRDIKSSNIL
LDDKYIAKISDFGISRSIPTDKTHLTTLVQGTLGYLDPEYYQSGQFTEKX
>pac.W.VtA.107
TVYKGMLHCGRIVAIKKAKLVDEGQVEQFINEVVILSQINHRNVVKLLGCCLETKVPLLV
YEFISNGTLFYHIHDQSEEFPISWVNCLRIATEVAGALAYLHSAASIPINHRDIKSTNIL
LDEKYNAKVSDFGISRSVATDKTHLTTTVQGTFGYFDPEYFQSSQFTEKX
>pac.W.VtA.108
TVYKGMLQDGRNVATKKSRIVDEGQIEQFINEVVILSQINHRNVVKLLGCCLETEVPLLV
YEFISNGTLFYHIHDQSEEFPISWVNCLRIATEVAGALAYLHSAASIPIYHRDIKSTNIL
LDDKCKAKVADFGTSRSVSVDKTHLTTLVQGTLGYLDPEYFQSSQFTDKX
>pac.W.VtA.109
TVYKGILEDHRIVAIKKSKLVDESQIEQFINEVVILSQINHRNVVKLLGCCLETEVPLLI
YEFINNGTLFHHIHDEGHVSSISWESRLRIAAETAGTLAYLHSATSIPIIHRDIKSTNIL
LDDNYTAKVADFGASRLVPLDQTQLTTLVQGTLGYLDPEYFISSQLIEKX
>pac.W.VtA.110
TVYKGMLLDGRIVAIKKSKIVNEGQIEQFINEVVILSHINHKNVVKLLGCCLETEVPLLV
YEFISNGTLFHHIHDQSEEFQCSWDNRLRIAIELANALAYLHSAASIPIYHRDIKSTNIL
LDSKYTAKVSDFGISRSVPNDKTHLTTLVQGTFGYLDPEYFQSSQFTEKX
>pac.W.VtA.113
TVYKGMLVDGMIVAIKKSKIVNEGQTEQFINELVILSHINHRNVVKLLGCCLETEVPLLV
YEFISNGTLFHHIHEKSEEFLSSWDNRLRIAIEVAGALAYLHSAASIPIYHRDIKSTNVL
IDGKYTAKVSDFGISRSVPIDKTHLTTSVQGTFGYMDPEYFQSNQFTEKX
>pac.W.VtB.201
HYNDNRILGQGGQGTVYKGMLQDGRIVAVKKATMVDEGQVEQFINEVVILSQINHRNVVK
LLGCCLETEVPLLVYEFISNGTLFYHIHDPTEEFVMSWDNRLRIATEVAGALAYLHSAAS
IPIYHRDVKSTNILLDDKHRSKVSDFGTARSVSIDKTHLTTIVQGTLGYLDPEYFQTSQF
TDKSDVYSFGVVLVELLTGEKPISLINSQERRNLVTHFIQLTKENRIFEILDDQVVEGRK
EEIEAVVNIAKRCLNFNGK
>pac.W.VtB.203
NYNENRILGQGGQGTVYKGMLHCGRIVAIKKAKLVDEGQVEQFINEVVILSQINHRNVVK
LLGCCLETEVPLLVYEFISNGTLFHHIHSQTEDFLMSWDNRLRIITEVAGALAYLHSSVS
MPIYHRDVKSTNILLDDKYRAKVSDFGTSRSVPVDKTHLTTLVQGTMGYLDPEYFQTSQF
TDKSDVYSFGVVLVELLTGEKPISLIGSQEKRNLVTYFIQSTKKNRLFEILDAQIVEGQK
EELEVVAGLAKRCVTLNGK
>pac.W.VtB.208
HYNENRILGQGGQGTVYKGMLHCGRIVAIKKAKLVDEGQVEQFINEVVILSQINHRNVVK

LLGCCLETEVPLLVYEFISNGTLFHHIHSQTEDFLMSWDNRLRIITEVAGALAYLHSSAS
MPIYHRDIKSTNILLDDKCKAKVADFGTSRSVSVDKTHLTTLVQGTLGYLDPEYFQSSQF
TDKSDVYSFGVVLVELLTGEKPISVFAQERRNLVTYFIQSIKENLLFEILDDRIIEGRKE
EIEAVASLAKRCLNLNGR
>pac.W.VtB.209
HFNENRILGQGGQGTVYKGMLVNGRIVAVKRSKIVNEGQIEQFINEVVILSHINHRNVVK
LLGCCLETEVPLLVYEFVSNGTLFHHIHDQSEEFLSSWDNRLRIAAEAAGALAYLHSTAS
IPIYHRDIKSTNILLDSKYTAKVSDFGISRSVPIDKTHLTTLVQGTFGYLDPEYFQSSQF
TEKSDVYSFGVVIVELLTGEKPISSVKSKEKMNLATHFLSSIRENCLFEILDAQVVKEDR
KEEIEIVANLAKRCLNLNGR
>pac.W.Ch.152
MLGDGRIVAVKKSKIVNECQIEQFINEVFILSHINHRNVVKLLGCCLETEVPLLVYEFIS
NGTLFHHIHDQSEEFLSSWDNRLRIAAEVAGTLAYLHSSASIPIYHKDIKSTNILLDGKY
TAKVADFGTSRSIPNDKTHLTTLVQGTFGYLDPEYFQSSQFTEKSDVX
>pac.W.Ch.156
ILEDHRIVAVKKSKLVDASQIEQFINEVVILSQINHRNVVRLLGCCLETEVPLLIYEFIS
NGTLFHHIHDEGHVSSISWEIRLRIATETAGTLAYLHSATSTPIINRDIKSTNILLDDNY
TAKVADFGASRLVPLDQTQLTTLVQGTLGYLDPEYFLSSQLTEKSDVX
>pac.W.Ch.157
MLVDGTIVAIKKSKIVDESQIEQFINEVVMLSQINHRNVVKLLGCCLEAEVPLLVYEFIS
NGTLFHYIHDQSEKFPNSWDNRLKIATDVAAALAYLHSAASIPIYHRDIKSSNILLDDKY
IAKISDFGASRSIPTDKTHLTTLVQGTFGYLDPEYFQSSQFTEKSDVX
>pac.W.Ch.158
MLLDGRIVAIKKSKIVNEGQIEQFINEVVILSHINHKNVVKLLGCCLETEVPLLVYEFIS
NGTLFHHIHDQSEEFQCSWDNRLRIAIELANALAYLHSAASIPIYHRDIKSTNILLDSKY
TAKVSDFGISRSVPNDKTHLTTLVQGTFGYLDPEYFQSSQFTEKK
>pac.W.Ch.160
MLLDGRIVAIKKSKIVNEGQIEQFINEVVILSHINHKNVVKLLGCCLETEVPLLVYEFIS
NGTLFHHIHDQSEEFQCSWDNRLRIAIELANALAYLHSAASIPIYHRDIKSTNILLDSKY
TAKVSDFGISRSVPNDKTHLTTLVQGTFGYLDPEYFQSSQFTEKSDVX
>pac.W.Ch.162
VLSDGRIIAVKKSKDANLIRLFVNEAIILSKINHTNVVKLIGYCKETEVPLLVYEFVING
TLAHHLHELGLRSSLGWGDRLKIAAEVANAIACMHSCKIIHRDIKSTNILLDENFTAKLC
DFGLSRTVPSDQDHVSTKELWGTEGYVDPDYIQTGQLTGKSDVX
>pac.W.Ch.164
ILADHRIVAVKKSKIVDESQIEQFINEVVILSQINHRNVVRLLGCCLETEVPLLIYEFIN
NGTLLHHIHDESHISSISWGCRLRIATETAEALAYLHSAASPPIIHRDIKSTNILLDEDY
TAKIADFGASRLVPLDYTQLTTLVQGTLGYLDPEYFHSSQLTEKSDVX
>pac.BRI.L.2
EATNGFHNDSLVGSGGFGDVYKAQLKDGSTVAIKKLIHVSGQGDREFTAEMETIGKIKHR
NLVPLLGYCKVGEERLLVYEYMRFGSLEDILHDRRKAGIKLNWVARRKIAIGAARGLAFL
HHNCIPHIIHRDMKSSNVLLDENLEARVSDFGMARLMSAMDTHLSVSTLAGTPGYVPPEY
YQSFRCSTKGDVYSYGVVLLELLTGKQPTDSADFGDNNLVGWVKQHARLRITDVFDPELM
KEEPNLEMELLEHLKIACACLDDRSWRRPTMIQV
>pac.BRI.L.6
KLTFADLLEATNGFHNDSLIGSGGFGDVYKAQLKDGSIVAIKKLIHVSGQGDREFTAEME
TIGKIKHRNLVPLLGYCKVREERLLVYEYMRFGSLDDILHDKRKAGIKLNWAARRKIAIG
AARGLAFLHHNCTPHIIHRDMKSSNVLLDGNLEARVSDFGMARLMSAMDTHLSVSTLAGT
PGYVPPEYYQSFRCSTKGDVYSYGVVLLELLTGKLPTDSTDFGDNNLVGWVKQHAKLKIS
DVFDPEIMKEDPSLEVELLQHLKIACACLSERPSRRPSMIQVMAMFKEIQA
>pac.BRL.B.8
ADSLIGSGGFGEVYKAQLKDGCVVAIKKLIHVTGQGDREFTAEMETIGKVKHRNLVPLLG
YCKIGEERLLVYEYMKWGSLETVLHDRTNGGGSKLNWAARKKIATGSARGLAFLHHNCIP
HIIHRDMKSSNVLLDENLEARVSDFGMARLMNAVDTHLSVSTLAGTPGYVPPEYYQSFRC
TTKGDVYSYGVILLELVSGKRPIDPSEFG
>pac.BRL.B.9
ADSLIGSGGFGEVYKAQLKDGCVVAIKRLIHVTGQGDREFTAEMETIGKIKHRNLVPLLG
YCKIGEERLLVYEYMKWGSLETVLHDRAKGRGSKLDWAARKKIAVGSARGLAFLHHSCIP
HIIHRDMKSSNVLLDENLEARVSDFGMARLMNALDTHLSVSTLAGTPGYVPPEYYQSFGC
TTKGDVYSYGVILLELLSGKRPIDPSEFG
>pac.BRL.B.12
ADSLIGSGGFGEVYKAQLKDGCVVAIKKLIHVTGQGDREFTAEMETIGKIKHRNLVPLLG
YCKIGEERLLVYEYMKWGSLETVLHDRAKGRGSKLDWAARKKIAVGSARGLAFLHHSCIP
HIIHRDMKSSNVLLDENLEARVSDFGMARLMNALDTHLSVSTLAGTPGYAPPEYYQSFRC
TTKGDVYSYGDILLELLSGKRPIDPSEFG
>pac.BRL.B.14
ADSLIGSGGFGEVYKAQLKDGCVVAIKKLIHVTGQGDREFTAEMETIGKIKHRNLVPLLG
YCKVGEERLLVYEYMKWGSLETVLHDRAKGRGSKLDWAARKKIAVGSARGLAFLHHSCIP
HIIHRDMKSSNVLLDENLGARVSDFGMARLMNALDTHLSVSTLAGTPGYVPPEYYQSFRC
TTKGDVYSYGVILLELLSGKRPIDPSEFG

>pac.BRL2.102
ASMIGCGGFGEVFKATLKDGSSVAIKKLIRLSCQGDREFMAEMETLGKIKHRNLVPLLGY
CKIGEERLLVYEFMEFGSLEEMLHGRTRAREGRRILTWEERKKIARGAAKGLCFLHHNCI
PHIIHRDMKSSNVLLDHDMEARVSDFGMARLISALDTHLSVSTLAGTPGYVPPEYYQSFR
CTAKGDVYSFGVVLLELLTGKRPTDKEDFGDTNLVGWVKMTVREGRGKEVIDPELLSAIK
GGDEDEAEEFKEMVRYLEITMQCVEDFPSRRPNMLQV
>pac.BRL2.108
ASMIGCGGFGEVSKATLKDGSSVAIKKLIRLSCQGDREFMAEMETLGKIKHRNLVPLLGY
CKIGEERLLVYEFMEFGSLEEMLHGRTRAREGRRILTWEERKKIARGAAKGLCFLHHNCI
PHIIHRDMKSSNVLLDHDMEARVSDFGMARLISALDTHLSVSTLAGTPGYVPPEYYQSFR
CTAKGDVYSFGVVLLELLTGKRPTDKEDFGDTNLVGWVKMTVREGRGKEVIDPELLSAIK
GGDEDEAEEFKEMVRYLEITMQCVEDFPSRRPNMLQV
