# Supplementary material for: Kinase domain-targeted isolation of defense-related receptor-like kinases (RLK/Pelle) in Platanus × acerifolia: phylogenetic and structural analysis
Source: BMC Res Notes. 2014 Dec 8;7:884. doi: 10.1186/1756-0500-7-884 (PMC4295470; doi:10.1186/1756-0500-7-884)
Supplement: Supplementary file 4 — Additional file 4: Reference sequences included in the phylogenies for comparison and to root the trees. Where RLK subfamilies or generic terms (i.e. Pto-like) are cited, all sequences reported in the source were tested in the phylogenies, except pseudogenes or putative proteins with truncated kinase domains. (PDF 23 KB) [file 13104_2014_3456_MOESM4_ESM.pdf]

Additional file 4. Reference sequences included in the phylogenies for comparison and to root the trees. Where RLK subfamilies or generic terms (i.e. Pto-like) are cited, all sequences reported in the source were tested in the phylogenies, except pseudogenes or putative proteins with truncated kinase domains.

**I - Reference sequences for phylogenies at the subfamily level**		
Proteins	Species	Source
CrRLK1L	*Arabidopsis* and rice	[50]
Pto and relatives	*Solanum pimpinellifolium*	[39]
WAK-like	*Arabidopsis*	[4, 49]
LRR X	*Arabidopsis*	[4]
LRR XII	*Arabidopsis*	[4]
Best Pac Blastp matches	*Arabidopsis*, poplar and rice	NCBI GenBank

**II - Reference sequences for phylogenetic investigation into the relations among Pto, Pto-like and CrRLK1L**		
Proteins	Species	Source
Best Pto Blastp matches (i.e. CrRLK1L)	*Arabidopsis*, Poplar, Castor bean, Grapevine, Rice	NCBI GenBank
Best Blastp matches obtained using wild/cultivated strawberry and bean Pto-like	*Fragaria vesca*, *Phaseolus vulgaris*	Phytozome<br><br>*F. vesca*:<br>mrna04250.1-v1.0-hybrid<br>mrna16583.1-v1.0-hybrid<br>mrna14010.1-v1.0-hybrid<br>mrna23363.1-v1.0-hybrid<br><br>*P. vulgaris*:<br>Phvul.003G239400.1<br>Phvul.004G038800.1<br>Phvul.007G188300.1
Pto and relatives	*Solanum pimpinellifolium*	[39]
Pto-like	Wild tomato species	[AF4.1]* [76]
Pto-like	*Capsicum* spp.	NCBI GenBank<br>Acc. Nos: AY316952, AY316954-67
Pto-like	*Solanum* spp.	[70]
Pto-like	*Nicotiana repanda*	[75]
Pto-like	*Phaseolus vulgaris*	[69]
Pto-like	Cultivated and wild strawberry	[74]
Pto-like	*Musa acuminata*	[73]
Whole CrRLK1L subfamily	*Arabidopsis* and rice	[50]
Whole CrRLK1L subfamily	Poplar	[5]
Whole CrRLK1L subfamily	Moss	NCBI GenBank, Acc. Nos:<br>XM_001757821, XM_001757822,<br>XM_001760648, XM_001760727,<br>XM_001779604
Whole CrRLK1L subfamily	Tomato	[6]
CrRLK1L-like	*Platanus* × *acerifolia*	This paper

III - Proteins used to root the trees		
Proteins	Species	Source
CTR1, Raf kinase family	*Arabidopsis*	[3], NCBI GenBank
MPK3, MAP kinase family	*Arabidopsis*	[3], NCBI GenBank
CPK7, CDPK family	*Arabidopsis*	[3], NCBI GenBank
Several other representatives of eukaryotic protein kinase families (ePKs)	*Many species*	[3]
APH(3')III (aminoglycoside 3'-phosphotransferase)	*Staphylococcus*	[3]

* References not reported in the text.

AF4.1 Rose LE, Langley CH, Bernal AJ, Michelmore RW: **Natural variation in the Pto disease resistance gene within species of wild tomato (Lycopersicon). I. Functional analysis of *Pto* alleles. Population genetics of Pto**. *Genetics*. 2005, **171:**345-357.
