# Supplementary material for: Kinase domain-targeted isolation of defense-related receptor-like kinases (RLK/Pelle) in Platanus × acerifolia: phylogenetic and structural analysis
Source: BMC Res Notes. 2014 Dec 8;7:884. doi: 10.1186/1756-0500-7-884 (PMC4295470; doi:10.1186/1756-0500-7-884)
Supplement: Supplementary file 5 — Additional file 5: The extracellular domain of Platanus × acerifolia ( Pac ) RLK/Pelles. Pairwise comparison between selected gene fragments obtained in this work, and the overlapping RLK/Pelle isotigs extracted from a 454 FLX dataset of a Platanus × acerifolia (Pac) transcriptome (Pilotti M., Brunetti A., Iacono M. and Pindo M. unpublished data). A Spanning regions and identity values. B The isotigs display a region 5′-upstream the kinase domain, which includes putative extracellular domains typical of the RLK/Pelle subfamilies dealt with in this work (output data from Pfam). This is a confirmation of the phylogenetic identification of the gene fragments. A SMART analysis predicted in a confident manner transmembrane domains in all the isotigs, which were located upstream the kinase domain and downstream the putative extracellular domains. (PDF 110 KB) [file 13104_2014_3456_MOESM5_ESM.pdf]

Additional file 5. The extracellular domain of *Platanus* × *acerifolia* (*Pac*) RLK/Pelles. Pairwise comparison between selected gene fragments obtained in this work, and the overlapping RLK/Pelle isotigs extracted from a 454 FLX dataset of a *Platanus* × *acerifolia* (*Pac*) transcriptome (Pilotti M., Brunetti A., Iacono M. and Pindo M. unpublished data). **A** Spanning regions and identity values. **B** The isotigs display a region 5'-upstream the kinase domain, which includes putative extracellular domains typical of the RLK/Pelle subfamilies dealt with in this work (output data from Pfam). This is a confirmation of the phylogenetic identification of the gene fragments. A SMART analysis predicted in a confident manner transmembrane domains in all the isotigs, which were located upstream the kinase domain and downstream the putative extracellular domains.

A

PCR gene fragments (length, nt)	Classification at the subfamily level	Isotigs KF208644-KF208653 (length, nt)	Spanning region	Identity score (nt)	Indel
pac.pt.10.306 (535)	CrRLK1L	1 (2322)	535	99.81	1 (1nt)
pac.ptd.5.2 (533)	CrRLK1L	2 (1707)	533	99.81	-
pac.pt.2.210 (533)	CrRLK1L	3 (2499)	533	100	-
pac.Erf.3 (493)	LRR XII	4 (2406)	493	89.05	1 (3nt)
pac.x.5.19 (496)	LRR XII	5 (2778)	496	91.13	-
pac.x.5.4 (487)	LRR XII	6 (3165)	487	93.43	-
pac.x.6.123 (502)	LRR XII	7 (1131)	502	88.25	1 (1nt)
pac.W.Ch.156 (503)	WAK-Like	8 (2283)	503	88.47	-
pac.BRI.L.2 (825)	LRR X-BRI1	9 (1071)	136 [a]	100	-
pac.BRL2.102 (832)	LRR X-BRL	10 (2109)	832	99.76	-

[a] pac.BRI.L2 overlapped the isotig in head-to-tail fashion

B

Pac (S1s/43) RLK/Pelle Isotigs	Classification	Domain with significant matches[1]	E-value	Domain with insignificant matches [a]	E-value
1	CrRLK1L	Malectin like (80-444)<br>Pkinase (574-772)	3.6e-64<br>3.2e-44	SKG6 (473-513)	0.2
2	CrRLK1L	Malectin like (2-132)<br>Pkinase (229-502)	1.1e-16<br>1.1e-46		
3	CrRLK1L	Malectin like (10-347)<br>Pkinase (498-768)	9.8e-54<br>7.8e-47		
4	LRR XII	LRR8 (291-351)<br>Pkinase (483-789)	1.4e-06<br>3.4e-43	LRR8 (1-56)<br>LRR4 2 copies (170-218)<br>LRR8 (221-279)	5.8e-05<br>9e-05<br>0.001
5	LRR XII	LRRNT 2 (53-92)<br>LRR6 (487-509)<br>LRR4, 2 copies (513-552)<br>LRR8 (561-620)<br>Pkinase (727-921)	1.1e-11<br>0.18<br>1.9e-06<br>4.9e-07<br>6.9e-33	LRR8 (120-180)<br>LRR8 (192-252)<br>LRR4 (313-365)<br>LRR8 (367-428)<br>LRR4 (440-477)	0.0003<br>9e-05<br>0.14<br>0.00044<br>0.06
6	LRR XII	LRRNT2 (47-87)<br>LRR8 (189-249)<br>LRR4 (534-579)<br>Pkinase (727-1027)	6e-12<br>2.5e-07<br>1e-06<br>3.2-40	LRR8 (118-177)<br>LRR8 (413-473)<br>LRR4, 2 copies (582-623)	6.7e-05<br>0.025<br>0.00045
7	LRR XII	LRR1 (13-34)<br>LRR8 (36-95)<br>Pkinase (202-373)	0.22<br>2.5e-10<br>5.1e-31		
8	WAK-Like	GUB WAK bind (46-157)<br>EGF-CA (312-352)<br>Pkinase (439-709)	7.2e-28<br>4e-07<br>1.5e-51	EGF3 (272-305)	0.054
9	LRR X-BRI1	LRR8 (120-178)	4.5e-07	LRR6 (1-24)<br>Pkinase (318-355)	0.099<br>0.011
10	LRR X-BRL	LRR4 (178-225)<br>Pkinase 406-689	9.3e-07<br>1.6e-43	LRR4 (14-61)<br>LRR4 (62-106)<br>PgaD (312-379)	4.5e-05<br>2.8e-06<br>0.012

[a] **:** domain names
Pkinase: Protein kinase
Malectin like: Carbohydrate-binding protein of the endoplasmic reticulum
LRR: Leucine rich repeat
LRRNT: Leucine rich repeat N-terminal
GUB WAK bind: Wall-associated receptor kinase galacturonan-binding
EGF-CA: Calcium-binding epidermal growth factor
EGF3: Epidermal growth factor
SKG6: Transmembrane alpha-helix
