# Supplementary material for: Kinase domain-targeted isolation of defense-related receptor-like kinases (RLK/Pelle) in Platanus × acerifolia: phylogenetic and structural analysis
Source: BMC Res Notes. 2014 Dec 8;7:884. doi: 10.1186/1756-0500-7-884 (PMC4295470; doi:10.1186/1756-0500-7-884)
Supplement: Supplementary file 6 — Additional file 6: Phylogenetic classification of RLK/Pelles of Platanus × acerifolia ( Pac ). The analyses were based on the Neighbor-joining method and show a general view of the relationship of the aa sequences of RLK/Pelles of Pac (pac, ♦ in black), Pto group (♦ in red) and other kinases. A. Contains representative sequences of the following species/groups: Pac, Pto group (red rhombus), Pto-like partial sequences from non-solanaceous species (white rhombus with a red outline), RLK/Pelle subfamilies of Arabidopsis[4], other families of the receptor kinase group – receptor tyrosine kinase (RTK) and Raf [3] - and other eukaryotic protein kinases (ePKs). The tree is rooted with a bacterial protein kinase [APH(3′)III also named aminoglycoside 3′-phosphotransferase]. B. Contains all Pac sequences reported in this work and their strict Arabidopsis homologs according to what emerged from A. A kinase domain region spanning subdomains I/II to the beginning of VIII, was used to infer the trees. See caption of Figure 1 and Methods for details on the representation of the trees. (PDF 90 KB) [file 13104_2014_3456_MOESM6_ESM.pdf]

Additional file 6. Phylogenetic classification of RLK/Pelles of *Platanus* × *acerifolia* (*Pac*). The analyses were based on the Neighbor-joining method and show a general view of the relationship of the aa sequences of RLK/Pelles of *Pac* (pac, ♦ in black), Pto group (♦ in red) and other kinases.

A. Contains representative sequences of the following species/groups: *Pac*, Pto group (red rhombus), Pto-like partial sequences from non-solanaceous species (white rhombus with a red outline), RLK/Pelle subfamilies of *Arabidopsis* [4], other families of the receptor kinase group – receptor tyrosine kinase (RTK) and Raf [3] - and other eukaryotic protein kinases (ePKs). The tree is rooted with a bacterial protein kinase [APH(3')III also named aminoglycoside 3'-phosphotransferase].

B. Contains all *Pac* sequences reported in this work and their strict *Arabidopsis* homologs according to what emerged from A.

A kinase domain region spanning subdomains I/II to the beginning of VIII, was used to infer the trees.

See caption of Figure 1 and Methods for details on the representation of the trees.

A

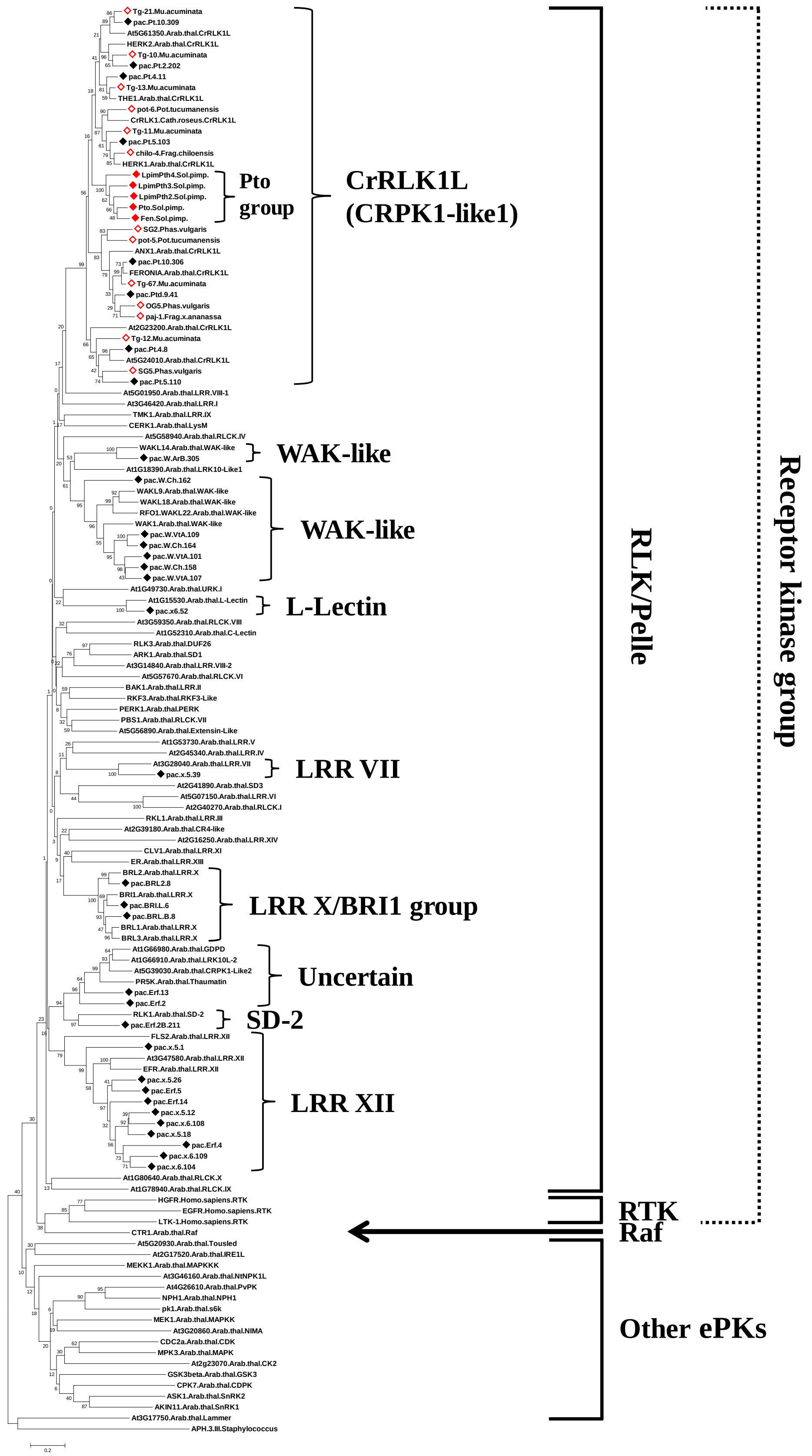

Tg-21.Mu.acuminata
pac.Pt.10.309
At5G61350.Arab.thal.CrRLK1L
HERK2.Arab.thal.CrRLK1L
Tg-10.Mu.acuminata
pac.Pt.2.202
pac.Pt.4.11
Tg-13.Mu.acuminata
THE1.Arab.thal.CrRLK1L
pot-6.Pot.tucumanensis
CrRLK1.Cath.roseus.CrRLK1L
Tg-11.Mu.acuminata
pac.Pt.5.103
chilo-4.Frag.chiloensis
HERK1.Arab.thal.CrRLK1L
LpimPth4.Sol.pimp.
LpimPth3.Sol.pimp.
LpimPth2.Sol.pimp.
Pto.Sol.pimp.
Fen.Sol.pimp.
SG2.Phas.vulgaris
pot-5.Pot.tucumanensis
ANX1.Arab.thal.CrRLK1L
pac.Pt.10.306
FERONIA.Arab.thal.CrRLK1L
Tg-67.Mu.acuminata
pac.Ptd.9.41
OG5.Phas.vulgaris
paj-1.Frag.x.ananassa
At2G23200.Arab.thal.CrRLK1L
Tg-12.Mu.acuminata
pac.Pt.4.8
At5G24010.Arab.thal.CrRLK1L
SG5.Phas.vulgaris
pac.Pt.5.110
At5G01950.Arab.thal.LRR.VIII-1
At3G46420.Arab.thal.LRR.I
TMK1.Arab.thal.LRR.IX
CERK1.Arab.thal.LysM
At5G58940.Arab.thal.RLCK.IV
WAKL14.Arab.thal.WAK-like
pac.W.ArB.305
At1G18390.Arab.thal.LRK10-Like1
pac.W.Ch.162
WAKL9.Arab.thal.WAK-like
WAKL18.Arab.thal.WAK-like
RFO1.WAKL22.Arab.thal.WAK-like
WAK1.Arab.thal.WAK-like
pac.W.VtA.109
pac.W.Ch.164
pac.W.VtA.101
pac.W.Ch.158
pac.W.VtA.107
At1G49730.Arab.thal.URK.I
At1G15530.Arab.thal.L-Lectin
pac.x6.52
At3G59350.Arab.thal.RLCK.VIII
At1G52310.Arab.thal.C-Lectin
RLK3.Arab.thal.DUF26
ARK1.Arab.thal.SD1
At3G14840.Arab.thal.LRR.VIII-2
At5G57670.Arab.thal.RLCK.VI
BAK1.Arab.thal.LRR.II
RKF3.Arab.thal.RKF3-Like
PERK1.Arab.thal.PERK
PBS1.Arab.thal.RLCK.VII
At5G56890.Arab.thal.Extensin-Like
At1G53730.Arab.thal.LRR.V
At2G45340.Arab.thal.LRR.IV
At3G28040.Arab.thal.LRR.VII
pac.x.5.39
At2G41890.Arab.thal.SD3
At5G07150.Arab.thal.LRR.VI
At2G40270.Arab.thal.RLCK.I
RKL1.Arab.thal.LRR.III
At2G39180.Arab.thal.CR4-like
At2G16250.Arab.thal.LRR.XIV
CLV1.Arab.thal.LRR.XI
ER.Arab.thal.LRR.XIII
BRL2.Arab.thal.LRR.X
pac.BRL2.8
BRI1.Arab.thal.LRR.X
pac.BRI.L.6
pac.BRL.B.8
BRL1.Arab.thal.LRR.X
BRL3.Arab.thal.LRR.X
At1G66980.Arab.thal.GDPD
At1G66910.Arab.thal.LRK10L-2
At5G39030.Arab.thal.CRPK1-Like2
PR5K.Arab.thal.Thaumatin
pac.Erf.13
pac.Erf.2
RLK1.Arab.thal.SD-2
pac.Erf.2B.211
FLS2.Arab.thal.LRR.XII
pac.x.5.1
At3G47580.Arab.thal.LRR.XII
EFR.Arab.thal.LRR.XII
pac.x.5.26
pac.Erf.5
pac.Erf.14
pac.x.5.12
pac.x.6.108
pac.x.5.18
pac.Erf.4
pac.x.6.109
pac.x.6.104
At1G80640.Arab.thal.RLCK.X
At1G78940.Arab.thal.RLCK.IX
HGFR.Homo.sapiens.RTK
EGFR.Homo.sapiens.RTK
LTK-1.Homo.sapiens.RTK
CTR1.Arab.thal.Raf
At5G20930.Arab.thal.Tousled
At2G17520.Arab.thal.IRE1L
MEKK1.Arab.thal.MAPKKK
At3G46160.Arab.thal.NtNPK1L
At4G26610.Arab.thal.PvPK
NPH1.Arab.thal.NPH1
pk1.Arab.thal.s6k
MEK1.Arab.thal.MAPKK
At3G20860.Arab.thal.NIMA
CDC2a.Arab.thal.CDK
MPK3.Arab.thal.MAPK
At2g23070.Arab.thal.CK2
GSK3beta.Arab.thal.GSK3
CPK7.Arab.thal.CDPK
ASK1.Arab.thal.SnRK2
AKIN11.Arab.thal.SnRK1
At3G17750.Arab.thal.Lammer
APH.3.III.Staphylococcus
0.2
Pto group
CrRLK1L (CRPK1-like1)
WAK-like
WAK-like
L-Lectin
LRR VII
LRR X/BRI1 group
Uncertain
SD-2
LRR XII
RLK/Pelle
RTK
Raf
Other ePKs
Receptor kinase group

B

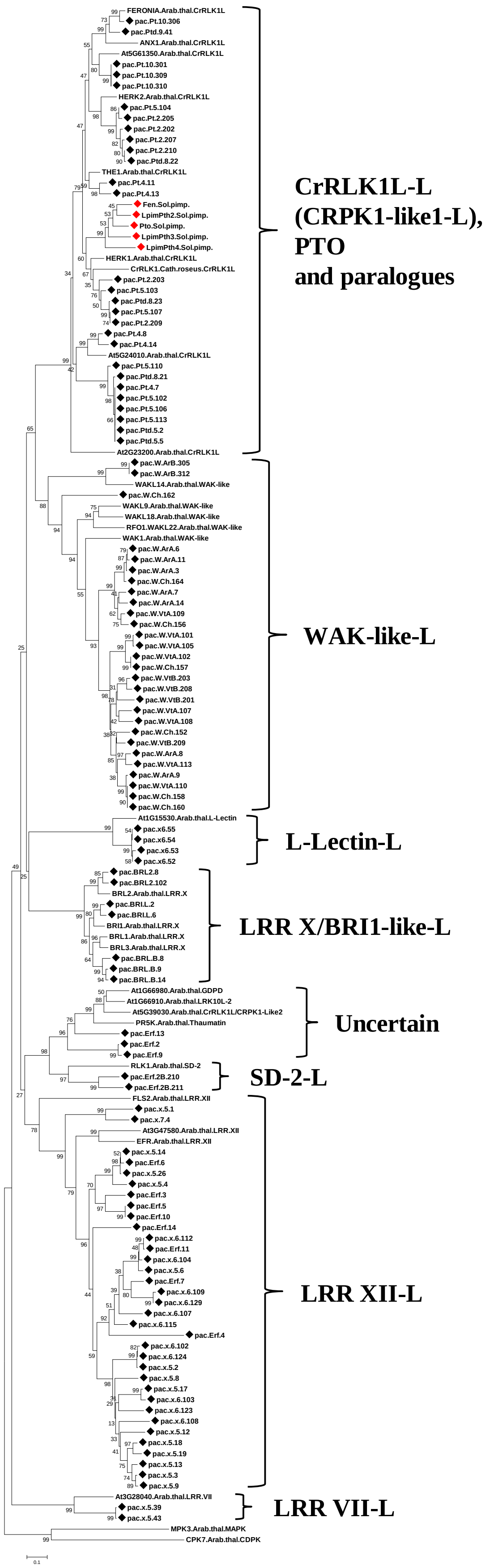
FERONIA.Arab.thal.CrRLK1L
pac.Pt.10.306
pac.Ptd.9.41
ANX1.Arab.thal.CrRLK1L
At5G61350.Arab.thal.CrRLK1L
pac.Pt.10.301
pac.Pt.10.309
pac.Pt.10.310
HERK2.Arab.thal.CrRLK1L
pac.Pt.5.104
pac.Pt.2.205
pac.Pt.2.202
pac.Pt.2.207
pac.Pt.2.210
pac.Ptd.8.22
THE1.Arab.thal.CrRLK1L
pac.Pt.4.11
pac.Pt.4.13
Fen.Sol.pimp.
LpimPth2.Sol.pimp.
Pto.Sol.pimp.
LpimPth3.Sol.pimp.
LpimPth4.Sol.pimp.
HERK1.Arab.thal.CrRLK1L
CrRLK1.Cath.roseus.CrRLK1L
pac.Pt.2.203
pac.Pt.5.103
pac.Ptd.8.23
pac.Pt.5.107
pac.Pt.2.209
pac.Pt.4.8
pac.Pt.4.14
At5G24010.Arab.thal.CrRLK1L
pac.Pt.5.110
pac.Ptd.8.21
pac.Pt.4.7
pac.Pt.5.102
pac.Pt.5.106
pac.Pt.5.113
pac.Ptd.5.2
pac.Ptd.5.5
At2G23200.Arab.thal.CrRLK1L
CrRLK1L-L
(CRPK1-like1-L),
PTO
and paralogues
pac.W.ArB.305
pac.W.ArB.312
WAKL14.Arab.thal.WAK-like
pac.W.Ch.162
WAKL9.Arab.thal.WAK-like
WAKL18.Arab.thal.WAK-like
RFO1.WAKL22.Arab.thal.WAK-like
WAK1.Arab.thal.WAK-like
pac.W.ArA.6
pac.W.ArA.11
pac.W.ArA.3
pac.W.Ch.164
pac.W.ArA.7
pac.W.ArA.14
pac.W.VtA.109
pac.W.Ch.156
pac.W.VtA.101
pac.W.VtA.105
pac.W.VtA.102
pac.W.Ch.157
pac.W.VtB.203
pac.W.VtB.208
pac.W.VtB.201
pac.W.VtA.107
pac.W.VtA.108
pac.W.Ch.152
pac.W.VtB.209
pac.W.ArA.8
pac.W.VtA.113
pac.W.ArA.9
pac.W.VtA.110
pac.W.Ch.158
pac.W.Ch.160
WAK-like-L
At1G15530.Arab.thal.L-Lectin
pac.x6.55
pac.x6.54
pac.x6.53
pac.x6.52
L-Lectin-L
pac.BRL2.8
pac.BRL2.102
BRL2.Arab.thal.LRR.X
pac.BRI.L.2
pac.BRI.L.6
BRI1.Arab.thal.LRR.X
BRL1.Arab.thal.LRR.X
BRL3.Arab.thal.LRR.X
pac.BRL.B.8
pac.BRL.B.9
pac.BRL.B.14
LRR X/BRI1-like-L
At1G66980.Arab.thal.GDPD
At1G66910.Arab.thal.LRK10L-2
At5G39030.Arab.thal.CrRLK1L/CRPK1-Like2
PR5K.Arab.thal.Thaumatin
pac.Erf.13
pac.Erf.2
pac.Erf.9
Uncertain
RLK1.Arab.thal.SD-2
pac.Erf.2B.210
pac.Erf.2B.211
SD-2-L
FLS2.Arab.thal.LRR.XII
pac.x.5.1
pac.x.7.4
At3G47580.Arab.thal.LRR.XII
EFR.Arab.thal.LRR.XII
pac.x.5.14
pac.Erf.6
pac.x.5.26
pac.x.5.4
pac.Erf.3
pac.Erf.5
pac.Erf.10
pac.Erf.14
pac.x.6.112
pac.Erf.11
pac.x.6.104
pac.x.5.6
pac.Erf.7
pac.x.6.109
pac.x.6.129
pac.x.6.107
pac.x.6.115
pac.Erf.4
pac.x.6.102
pac.x.6.124
pac.x.5.2
pac.x.5.8
pac.x.5.17
pac.x.6.103
pac.x.6.123
pac.x.6.108
pac.x.5.12
pac.x.5.18
pac.x.5.19
pac.x.5.13
pac.x.5.3
pac.x.5.9
LRR XII-L
At3G28040.Arab.thal.LRR.VII
pac.x.5.39
pac.x.5.43
LRR VII-L
MPK3.Arab.thal.MAPK
CPK7.Arab.thal.CDPK
0.1
