# Supplementary material for: Kinase domain-targeted isolation of defense-related receptor-like kinases (RLK/Pelle) in Platanus × acerifolia: phylogenetic and structural analysis
Source: BMC Res Notes. 2014 Dec 8;7:884. doi: 10.1186/1756-0500-7-884 (PMC4295470; doi:10.1186/1756-0500-7-884)
Supplement: Supplementary file 7 — Additional file 7: The CrRLK1L-L of Platanus × acerifolia ( Pac ). Neighbor-joining analysis to compare CrRLK1L-L of Pac (pac, ♦), with the complete CrRLK1L subfamily of Arabidopsis and rice; Pto and paralogs of Solanum pimpinellifolium were also included (red rhombus); The subdivision into CRPK1-like 1 and CRPK1-like 2 is from Shiu and Bleecker [4]. aa sequences were used to infer the tree. Analysis was based on the kinase domain region spanning subdomain I through to IX. See caption of Figure 1 for details on the representation of the tree. Arrows indicate Arabidopsis CrRLK1L which were functionally characterised. (PDF 47 KB) [file 13104_2014_3456_MOESM7_ESM.pdf]

Additional file 7. The CrRLK1L-L of *Platanus* × *acerifolia* (*Pac*). Neighbor-joining analysis to compare CrRLK1L-L of *Pac* (pac, ♦), with the complete CrRLK1L subfamily of *Arabidopsis* and rice; Pto and paralogs of *Solanum pimpinellifolium* were also included (red rhombus); The subdivision into CRPK1-like 1 and CRPK1-like 2 is from Shiu and Bleecker [4]. aa sequences were used to infer the tree. Analysis was based on the kinase domain region spanning subdomain I through to IX. See caption of Figure 1 for details on the representation of the tree. Arrows indicate *Arabidopsis* CrRLK1L which were functionally characterised.

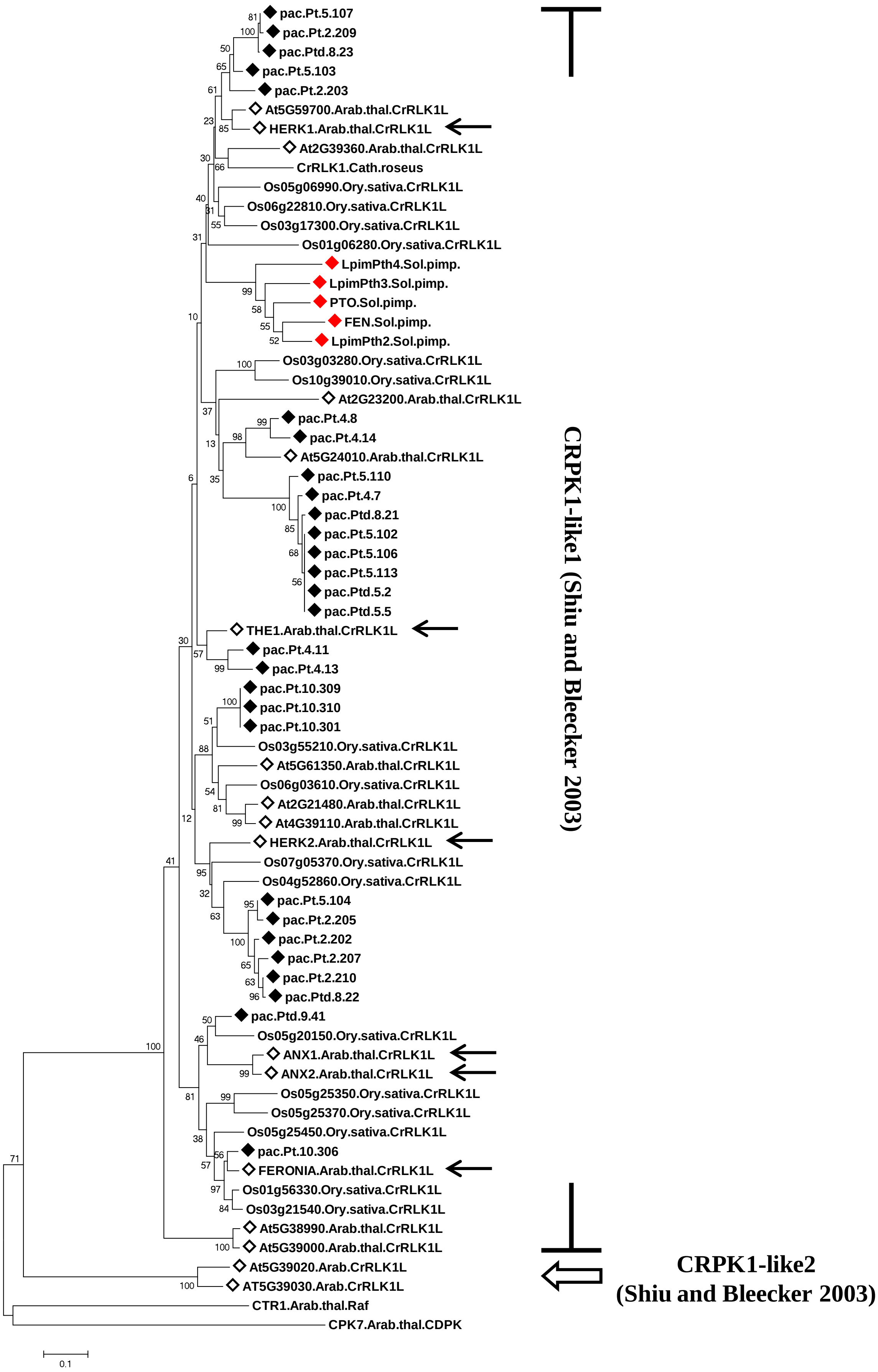
pac.Pt.5.107
pac.Pt.2.209
pac.Ptd.8.23
pac.Pt.5.103
pac.Pt.2.203
At5G59700.Arab.thal.CrRLK1L
HERK1.Arab.thal.CrRLK1L
At2G39360.Arab.thal.CrRLK1L
CrRLK1.Cath.roseus
Os05g06990.Ory.sativa.CrRLK1L
Os06g22810.Ory.sativa.CrRLK1L
Os03g17300.Ory.sativa.CrRLK1L
Os01g06280.Ory.sativa.CrRLK1L
LpimPth4.Sol.pimp.
LpimPth3.Sol.pimp.
PTO.Sol.pimp.
FEN.Sol.pimp.
LpimPth2.Sol.pimp.
Os03g03280.Ory.sativa.CrRLK1L
Os10g39010.Ory.sativa.CrRLK1L
At2G23200.Arab.thal.CrRLK1L
pac.Pt.4.8
pac.Pt.4.14
At5G24010.Arab.thal.CrRLK1L
pac.Pt.5.110
pac.Pt.4.7
pac.Ptd.8.21
pac.Pt.5.102
pac.Pt.5.106
pac.Pt.5.113
pac.Ptd.5.2
pac.Ptd.5.5
THE1.Arab.thal.CrRLK1L
pac.Pt.4.11
pac.Pt.4.13
pac.Pt.10.309
pac.Pt.10.310
pac.Pt.10.301
Os03g55210.Ory.sativa.CrRLK1L
At5G61350.Arab.thal.CrRLK1L
Os06g03610.Ory.sativa.CrRLK1L
At2G21480.Arab.thal.CrRLK1L
At4G39110.Arab.thal.CrRLK1L
HERK2.Arab.thal.CrRLK1L
Os07g05370.Ory.sativa.CrRLK1L
Os04g52860.Ory.sativa.CrRLK1L
pac.Pt.5.104
pac.Pt.2.205
pac.Pt.2.202
pac.Pt.2.207
pac.Pt.2.210
pac.Ptd.8.22
pac.Ptd.9.41
Os05g20150.Ory.sativa.CrRLK1L
ANX1.Arab.thal.CrRLK1L
ANX2.Arab.thal.CrRLK1L
Os05g25350.Ory.sativa.CrRLK1L
Os05g25370.Ory.sativa.CrRLK1L
Os05g25450.Ory.sativa.CrRLK1L
pac.Pt.10.306
FERONIA.Arab.thal.CrRLK1L
Os01g56330.Ory.sativa.CrRLK1L
Os03g21540.Ory.sativa.CrRLK1L
At5G38990.Arab.thal.CrRLK1L
At5G39000.Arab.thal.CrRLK1L
At5G39020.Arab.CrRLK1L
AT5G39030.Arab.CrRLK1L
CTR1.Arab.thal.Raf
CPK7.Arab.thal.CDPK
0.1
CRPK1-like1 (Shiu and Bleecker 2003)
CRPK1-like2
(Shiu and Bleecker 2003)
