# Supplementary material for: Kinase domain-targeted isolation of defense-related receptor-like kinases (RLK/Pelle) in Platanus × acerifolia: phylogenetic and structural analysis
Source: BMC Res Notes. 2014 Dec 8;7:884. doi: 10.1186/1756-0500-7-884 (PMC4295470; doi:10.1186/1756-0500-7-884)

Additional file 8. Unwanted RLK/Pelles of *Platanus* × *acerifolia* (*Pac*). Sequences were obtained with primers aimed at isolating members of LRR XII subfamily. **A** Neighbor-joining tree based on the aa sequences of *Pac* (pac, ♦), the nearest RLK/Pelle members of *Arabidopsis* (◊) and other unknown, homologous sequences of rice and poplar. Analysis was based on the kinase domain region spanning subdomains I/II to the beginning of VIII. See caption of Figure 1 for details on the representation of the tree. **B** Multiple alignment of RD motif (shaded grey), non-RD motif (shaded black) and an atypical variant of the non-RD-Asp (shaded black). **C** Phylogenetic analysis was ambiguous in determining the homology relationship of pac.Erf.2, pac.Erf.9 and pac.Erf.13 with the *Arabidopsis* RLK/Pelle subfamilies GDPD, LRK10L-2, CRPK1-like2 (i.e. CrRLK1L) and Thaumatin. The table thus shows the identity and similarity values and number of gaps derived from the pairwise comparison of these sequences with one *Arabidopsis* relative for each subfamily which, in preliminary analyses, were the nearest sequences.

C

Plane tree genes<br>• Lenght, aa<br>• Subdomain spanning	Arabidopsis homologs	aa id. (%)	aa sim. (%)	Gaps (%)
pac.Erf.13<br>• 149<br>• I/II→VIII	At1G66910.LRK10L-2	61.5	74.3	3/148 (2.0)
	PR5K.Thaumatin	58.7	73.3	4/150 (2.7)
	At1G66980.GDPD	62.2	76.4	3/148 (2)
	At5G39030.CRPK1-like-2	60.1	74.3	3/148 (2)
pac.Erf.2<br>• 149<br>• I/II→VIII	At1G66910.LRK10L-2	56.1	75.7	3/148 (2.0)
	PR5K.Thaumatin	55.5	75.3	3/146 (2.1)
	At1G66980.GDPD	54.1	73.6	3/148 (2)
	At5G39030.CRPK1-like-2	54	72.7	7/150 (4.7)
pac.Erf.9<br>• 149<br>• I/II→VIII	At1G66910.LRK10L-2	55.4	75.0	3/148 (2.0)
	PR5K.Thaumatin	54.8	74.7	3/146 (2.1)
	At1G66980.GDPD	53.4	73	3/148 (2.0)
	At5G39030.CRPK1-like-2	51.4	70.9	3/148 (2.0)

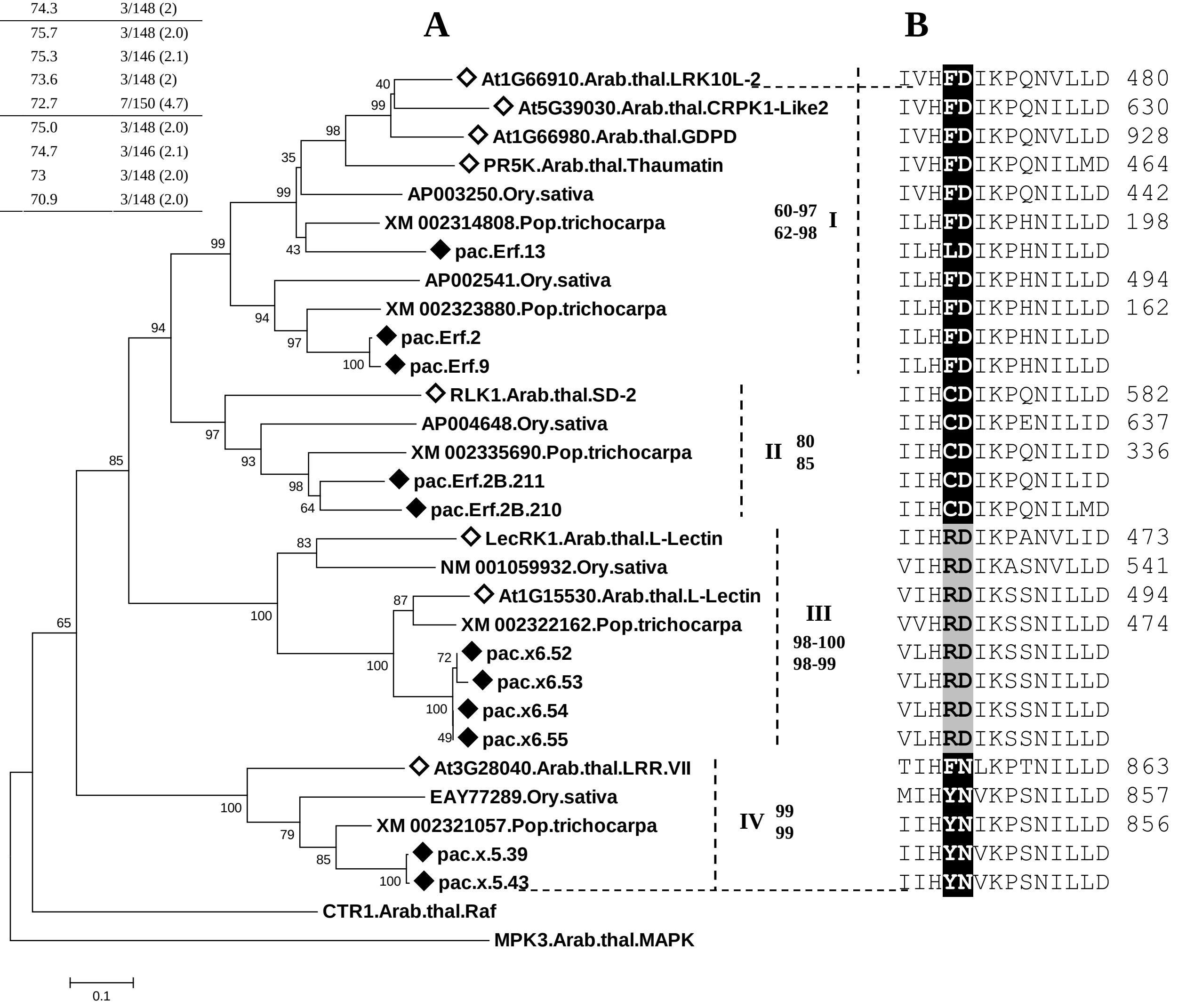

Supplement: Supplementary file 8 — Additional file 8: Unwanted RLK/Pelles of Platanus × acerifolia ( Pac ). Sequences were obtained with primers aimed at isolating members of LRR XII subfamily. A Neighbor-joining tree based on the aa sequences of Pac (pac, ♦), the nearest RLK/Pelle members of Arabidopsis (◊) and other unknown, homologous sequences of rice and poplar. Analysis was based on the kinase domain region spanning subdomains I/II to the beginning of VIII. See caption of Figure 1 for details on the representation of the tree. B Multiple alignment of RD motif (shaded grey), non-RD motif (shaded black) and an atypical variant of the non-RD-Asp (shaded black). C Phylogenetic analysis was ambiguous in determining the homology relationship of pac.Erf.2, pac.Erf.9 and pac.Erf.13 with the Arabidopsis RLK/Pelle subfamilies GDPD, LRK10L-2, CRPK1-like2 (i.e. CrRLK1L) and Thaumatin. The table thus shows the identity and similarity values and number of gaps derived from the pairwise comparison of these sequences with one Arabidopsis relative for each subfamily which, in preliminary analyses, were the nearest sequences. (PDF 172 KB) [file 13104_2014_3456_MOESM8_ESM.pdf]
