# Supplementary material for: Kinase domain-targeted isolation of defense-related receptor-like kinases (RLK/Pelle) in Platanus × acerifolia: phylogenetic and structural analysis
Source: BMC Res Notes. 2014 Dec 8;7:884. doi: 10.1186/1756-0500-7-884 (PMC4295470; doi:10.1186/1756-0500-7-884)
Supplement: Supplementary file 9 — Additional file 9: Wak1-clade-specific amino acid motif helps to identify close homologs in Platanus × acerifolia ( Pac ). Amino acidic residues (shaded black) conserved among sequences of Wak1-headed clade of Arabidopsis (RLK/Pelle subfamily WAK-like) and of the II phylogenetic clade of Wak-like-L of Pac (refer to phylogeny of Figure 3). This motif suggests a genetic correlation between the two sequence groups. (PDF 130 KB) [file 13104_2014_3456_MOESM9_ESM.pdf]

Additional file 9. Wak1-clade-specific amino acid motif helps to identify close homologs in *Platanus* × *acerifolia* (*Pac*). Amino acidic residues (shaded black) conserved among sequences of Wak1-headed clade of *Arabidopsis* (RLK/Pelle subfamily WAK-like) and of the II phylogenetic clade of Wak-like-L of *Pac* (refer to phylogeny of Figure 3). This motif suggests a genetic correlation between the two sequence groups.

Clade	Sequence			
WAK1 clade	WAK1.Arab.thal.Wak-like	TVYKGILPDNS 432.......	GTLFDHLH-G--SMIDS--SL-TWEHRLK 510.......	LHSSASIPIIHRDIKTANI 540
	WAK3.Arab.thal.Wak-like	TVYKGILPDNT 438	GTLFDHLH-G--SIFDS--SL-TWEHRLR 516	LHSSASIPIIHRDIKTANI 546
	WAKL16.Arab.thal.Wak-like	TVYKGILPDNS 131	GSLFDHLH-G--SMFVS--SL-TWEHRLE 209	LHSGASIPIIHRDIKTENI 239
	WAK5.Arab.thal.Wak-like	TVYKGILQDNS 431	GTLFDHLH-G--SMFDS--SL-TWEHRLR 509	LHSYASIPIIHRDVKTANI 539
	WAK2.Arab.thal.Wak-like	TVYKGILPDNS 427	GTLFDHLH-G--SLYDS--SL-TWEHRLR 505	LHSSASIPIIHRDIKTANI 535
	WAK4.Arab.thal.Wak-like	TVYKGILPDNS 433	GTLFDHLH-G--SMFDS--SL-TWEHRLR 511	LHSSASIPIIHRDIKTANI 541
II clade	pac.W.VtA.109	TVYKGILEDHR	GTLFHHIH-D--EGHVS--SI-SWESRLR	LHSATSIPIIHRDIKSTNI
	pac.W.Ch.156	-----ILEDHR	GTLFHHIH-D--EGHVS--SI-SWEIRLR	LHSATSTPIINRDIKSTNI
	pac.W.ArA.6	TVYKGILADHR	GTLSHHIH-D--ESHIS--SI-SWGCRLR	LHSAASPPIIHRDIKSTNI
	pac.W.ArA.11	TVYKGILADHR	GTLSHHIH-D--ESHIS--SI-SWGCRLR	LHSAASPPIIHRDIKSTNI
	pac.W.ArA.3	TVYKGILADHR	GTLSHHIH-D--ESHIS--SI-SWGCRLR	LHSAASPPIIHRDIKSTNI
	pac.W.Ch.164	-----ILADHR	GTLLHHIH-D--ESHIS--SI-SWGCRLR	LHSAASPPIIHRDIKSTNI
	pac.W.ArA.7	TVYKGILADHR	GTLFHHIH-D--EGHVS--SI-SWGSRMR	LHSAASIPIIHRDIKSTNI
	pac.W.ArA.14	TVYKGILADHR	GTLFHHIH-D--EGHVS--SI-SWEIRLR	LHSAVSTPIIHRDIKSANI
RFO1 clade	WAKL9.Arab.thal.Wak-like	TVYKGMLVDGR 470	GNLFEHLH-D--EFDEN--IMATWNIRLR 549	LHSSASSPIYHRDVKSTNI 579
	WAKL10.Arab.thal.Wak-like	TVYKGMLVDGR 456	GNLFEHLH-D--DSDDY--TMTTWEVRLR 535	LHSAASSPIYHRDIKSTNI 565
	WAKL1.Arab.thal.Wak-like	TVYKGMLVDGS 452	GDLFKRLH-D--ESDDY--TM-TWEVRLR 530	MHSAASFPIFHRDIKTTNI 560
	WAKL5.Arab.thal.Wak-like	TVYKGMMVDGK 435	GDMFKRLH-D--ESDDY--AM-TWEVRLR 513	MHSAASFPIYHRDIKTTNI 543
	WAKL6.Arab.thal.Wak-like	TVYKGMLAEGR 455	GDLFKRLH-EKSESNDY--TM-TWEVRLR 535	MHSAASIPIYHRDIKTTNI 565
	WAKL2.Arab.thal.Wak-like	TVYKGMLVDGR 439	GDLCKRLH-D--ESDDY--TM-TWEVRLH 517	LHSAASFPIYHRDIKTTNI 547
	WAKL4.Arab.thal.Wak-like	TVYKGMLVDGR 465	GDLCKRLR-D--ECDDY--IM-TWEVRLH 543	LHSAASFPIYHRDIKTTNI 573
	RFO1.WAKL22.Arab.thal.Wak-like	TVYKGMLVDGR 444	GDLFKRLH-H--DSDDY--TM-TWDVRLR 522	LHSAASTPVYHRDVKTTNI 552
	WAKL8.Arab.thal.Wak-like	TVYKGMLEDGM 413	RNLFDHLH-N--PSEDF--PM-SWEVRLC 491	LHSAVSIPIYHRDVKSTNI 521
	WAKL17.Arab.thal.Wak-like	TVYKGMLVDGR 467	GNLFKHIHEE--EADDY--TM-IWGMRLR 546	LHSAASSPIYHRDIKSTNI 576
	WAKL18.Arab.thal.Wak-like	TVYKGMLVDGR 476	GNLFKHIHEE--ESDDY--TM-LWGMRLR 555	LHSSASSPIYHRDIKSTNI 585
	WAKL11.Arab.thal.Wak-like	TVYKGMLVDGR 474	GNLFQHIH-E--ESDDY--TK-TWGMRLR 552	LHSAASSPIYHRDIKSTNI 582
	WAKL13.Arab.thal.Wak-like	TVYKGMLVDGR 477	GNLFQHLH-E--EFDDY--TA-LWGVRMR 555	LHTAACSPIYHRDIKSTNI 585
I clade	pac.W.VtA.107	TVYKGMLHCGR	GTLFYHIH-D--QSEEF--PI-SWVNCLR	LHSAASIPINHRDIKSTNI
	pac.W.VtB.203	TVYKGMLHCGR	GTLFHHIH-S--QTEDF--LM-SWDNRLR	LHSSVSMPIYHRDVKSTNI
	pac.W.VtB.208	TVYKGMLHCGR	GTLFHHIH-S--QTEDF--LM-SWDNRLR	LHSSASMPIYHRDIKSTNI
	pac.W.VtB.201	TVYKGMLQDGR	GTLFYHIH-D--PTEEF--VM-SWDNRLR	LHSAASIPIYHRDVKSTNI
	pac.W.VtA.108	TVYKGMLQDGR	GTLFYHIH-D--QSEEF--PI-SWVNCLR	LHSAASIPIYHRDIKSTNI
	pac.W.VtA.101	TVYKGMLVDGM	GTLFHYIH-D--QSEEF--PN-SWDNRLK	LHSAASMPIYHRDIKSSNI
	pac.W.VtA.105	TVYKGMLVDGM	GTLFHYIH-D--QSEEF--PN-SWDNRLK	LHSAASMPISHRDIKSSNI
	pac.W.VtA.102	TVYKGMLVDGT	GTLFHYIH-D--QSEKF--PN-SWDNRLK	LHSAASIPIYHRDIKSSNI
	pac.W.Ch.157	-----MLVDGT	GTLFHYIH-D--QSEKF--PN-SWDNRLK	LHSAASIPIYHRDIKSSNI
	pac.W.ArA.8	TVYKGMLPDGR	GTLFYHIH-E--KSEEF--LS-SWDNRLR	LHSAASIPIYHRDIKSTNI
	pac.W.VtA.113	TVYKGMLVDGM	GTLFHHIH-E--KSEEF--LS-SWDNRLR	LHSAASIPIYHRDIKSTNV
	pac.W.Ch.158	-----MLLDGR	GTLFHHIH-D--QSEEF--QC-SWDNRLR	LHSAASIPIYHRDIKSTNI
	pac.W.Ch.160	-----MLLDGR	GTLFHHIH-D--QSEEF--QC-SWDNRLR	LHSAASIPIYHRDIKSTNI
	pac.W.VtA.110	TVYKGMLLDGR	GTLFHHIH-D--QSEEF--QC-SWDNRLR	LHSAASIPIYHRDIKSTNI
	pac.W.ArA.9	TVYKGMLPDGR	GTLFHHIH-V--QSEEF--QC-SWDNRLR	LHSAASIPIYHRDIKSTNI
	pac.W.VtB.209	TVYKGMLVNGR	GTLFHHIH-D--QSEEF--LS-SWDNRLR	LHSTASIPIYHRDIKSTNI
	pac.W.Ch.152	-----MLGDGR	GTLFHHIH-D--QSEEF--LS-SWDNRLR	LHSSASIPIYHKDIKSTNI
III clade →	pac.W.Ch.162	-----VLSDGR	GTLAHHLH-E--LGLRS--SL-GWGDRLK	MHSCK---IIHRDIKSTNI
	WAKL15.Arab.thal.Wak-like	EVFKGNLDDGT 377	GTLFEHIYGGGGGGGGLYDHL-PLRRRLM 460	LHSSSSPPIYHRDVKSSNI 490
	WAKL20.Arab.thal.Wak-like	EVFKAVLEDGT 386	GTLFEHLH---GSSDRTWKPL-TWRRRLQ 466	LHSAAQPPIYHRDVKSSNI 496
IV clade	pac.W.ArB.305	TVYAGKLHNVD	GTLSQHLQ-R--EIST---GL-PWMTRLT	LHSAINPPIFHRDIKSSNI
	pac.W.ArB.312	TVYAGKLHNVD	GTLSQHLQ-R--EIST---GL-PWMTRLT	LHSAINPPIFHRDIKSSNI
	WAKL14.Arab.thal.Wak-like	TVYRGKLQNDE 371	GTLSEHLQ-R--DRGS---GL-PWTLRLT 448	LHSSMNPPIYHRDIKSTNI 478
	WAKL21.Arab.thal.Wak-like	TVYAGEFPNSS 337	GTLYQHLQ-H--ERGQP--PL-SWQLRLA 415	LHSSVNPPIYHRDIKSSNI 445
