# Supplementary material for: Kinase domain-targeted isolation of defense-related receptor-like kinases (RLK/Pelle) in Platanus × acerifolia: phylogenetic and structural analysis
Source: BMC Res Notes. 2014 Dec 8;7:884. doi: 10.1186/1756-0500-7-884 (PMC4295470; doi:10.1186/1756-0500-7-884)
Supplement: Supplementary file 10 — Additional file 10: Classification of Pac sequences based on maximum-likelihood (ML) phylogenetic analyses. Analyses were performed on the same dataset alignments used for crucial Neighbor-joining trees presented in this work (aa sequences). A1 and A2 match with Additional file 6B (all Pac sequences compared with homologous Arabidopsis RLK Pelles); B matches with Additional file 7 (Arabidopsis and rice CrRLK1L vs Pac CrRLK1L-L); C matches with Figure 2 (Arabidopsis LRR XII vs Pac LRR XII-L); D matches with Figure 3 (Arabidopsis WAK-like vs Pac WAK-like-L); E with Figure 4 (Arabidopsis LRR X vs Pac LRR X-BRI1-L group). Editing matches that of counterpart figures. All trees were inferred with Mega 6 except the one represented in A2 that was TREE PUZZLE-implemented. With regard to Mega 6 analyses, the percentage of trees in which the associated sequences clustered together in the bootstrap test (1,000 replicates) is shown for each node; trees are drawn to scale in the number of substitutions per site (scale at the bottom). TREE-PUZZLE analysis was conducted with 50,000 puzzling steps and quartet puzzling support values are shown for each node. (PDF 161 KB) [file 13104_2014_3456_MOESM10_ESM.pdf]

Additional file 10. Classification of *Pac* sequences based on maximum-likelihood (ML) phylogenetic analyses. Analyses were performed on the same dataset alignments used for crucial Neighbor-joining trees presented in this work (aa sequences). A1 and A2 match with Additional file 6B (all *Pac* sequences compared with homologous *Arabidopsis* RLK Pelles); B matches with Additional file 7 (*Arabidopsis* and rice CrRLK1L vs *Pac* CrRLK1L-L); C matches with Fig. 2 (*Arabidopsis* LRR XII vs *Pac* LRR XII-L); D matches with Fig. 3 (*Arabidopsis* WAK-like vs *Pac* WAK-like-L); E with Fig. 4 (*Arabidopsis* LRR X vs *Pac* LRR X-BRI1-L group). Editing matches that of counterpart figures.

All trees were inferred with Mega 6 except the one represented in A2 that was TREE PUZZLE-implemented. With regard to Mega 6 analyses, the percentage of trees in which the associated sequences clustered together in the bootstrap test (1,000 replicates) is shown for each node; trees are drawn to scale in the number of substitutions per site (scale at the bottom). TREE-PUZZLE analysis was conducted with 50,000 puzzling steps and quartet puzzling support values are shown for each node.

A1

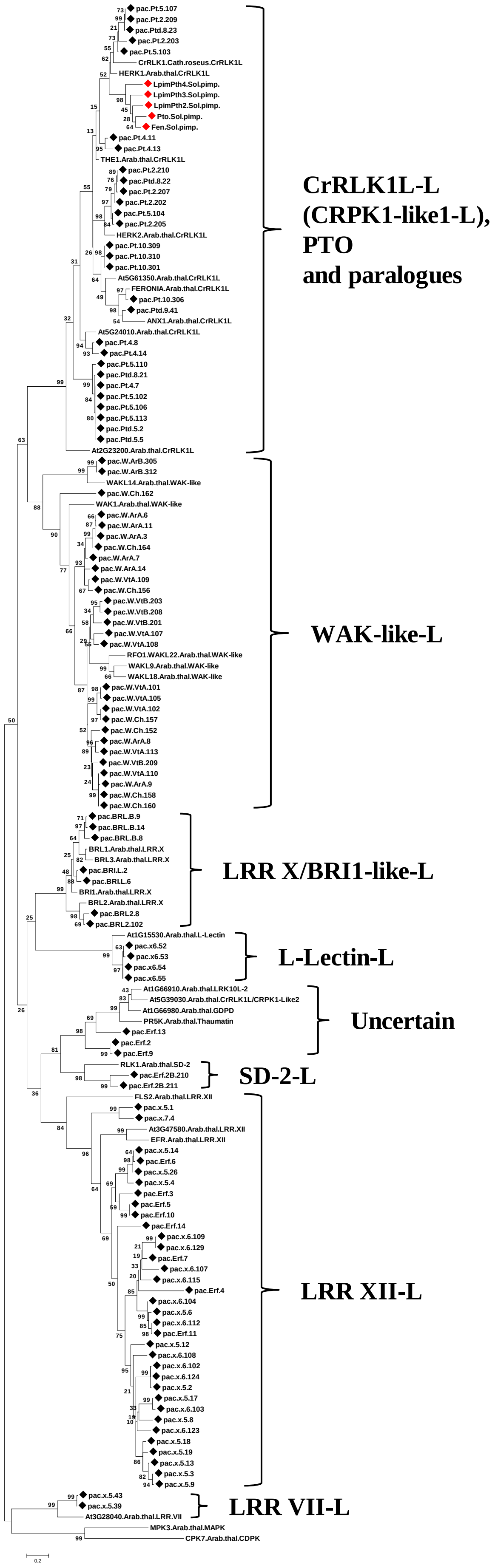
pac.Pt.5.107
pac.Pt.2.209
pac.Ptd.8.23
pac.Pt.2.203
pac.Pt.5.103
CrRLK1.Cath.roseus.CrRLK1L
HERK1.Arab.thal.CrRLK1L
LpimPth4.Sol.pimp.
LpimPth3.Sol.pimp.
LpimPth2.Sol.pimp.
Pto.Sol.pimp.
Fen.Sol.pimp.
pac.Pt.4.11
pac.Pt.4.13
THE1.Arab.thal.CrRLK1L
pac.Pt.2.210
pac.Ptd.8.22
pac.Pt.2.207
pac.Pt.2.202
pac.Pt.5.104
pac.Pt.2.205
HERK2.Arab.thal.CrRLK1L
pac.Pt.10.309
pac.Pt.10.310
pac.Pt.10.301
At5G61350.Arab.thal.CrRLK1L
FERONIA.Arab.thal.CrRLK1L
pac.Pt.10.306
pac.Ptd.9.41
ANX1.Arab.thal.CrRLK1L
At5G24010.Arab.thal.CrRLK1L
pac.Pt.4.8
pac.Pt.4.14
pac.Pt.5.110
pac.Ptd.8.21
pac.Pt.4.7
pac.Pt.5.102
pac.Pt.5.106
pac.Pt.5.113
pac.Ptd.5.2
pac.Ptd.5.5
At2G23200.Arab.thal.CrRLK1L
CrRLK1L-L
(CRPK1-like1-L),
PTO
and paralogues
pac.W.ArB.305
pac.W.ArB.312
WAKL14.Arab.thal.WAK-like
pac.W.Ch.162
WAK1.Arab.thal.WAK-like
pac.W.ArA.6
pac.W.ArA.11
pac.W.ArA.3
pac.W.Ch.164
pac.W.ArA.7
pac.W.ArA.14
pac.W.VtA.109
pac.W.Ch.156
pac.W.VtB.203
pac.W.VtB.208
pac.W.VtB.201
pac.W.VtA.107
pac.W.VtA.108
RFO1.WAKL22.Arab.thal.WAK-like
WAKL9.Arab.thal.WAK-like
WAKL18.Arab.thal.WAK-like
pac.W.VtA.101
pac.W.VtA.105
pac.W.VtA.102
pac.W.Ch.157
pac.W.Ch.152
pac.W.ArA.8
pac.W.VtA.113
pac.W.VtB.209
pac.W.VtA.110
pac.W.ArA.9
pac.W.Ch.158
pac.W.Ch.160
WAK-like-L
pac.BRL.B.9
pac.BRL.B.14
pac.BRL.B.8
BRL1.Arab.thal.LRR.X
BRL3.Arab.thal.LRR.X
pac.BRI.L.2
pac.BRI.L.6
BRI1.Arab.thal.LRR.X
BRL2.Arab.thal.LRR.X
pac.BRL2.8
pac.BRL2.102
LRR X/BRI1-like-L
At1G15530.Arab.thal.L-Lectin
pac.x6.52
pac.x6.53
pac.x6.54
pac.x6.55
L-Lectin-L
At1G66910.Arab.thal.LRK10L-2
At5G39030.Arab.thal.CrRLK1L/CRPK1-Like2
At1G66980.Arab.thal.GDPD
PR5K.Arab.thal.Thaumatin
pac.Erf.13
pac.Erf.2
pac.Erf.9
Uncertain
RLK1.Arab.thal.SD-2
pac.Erf.2B.210
pac.Erf.2B.211
SD-2-L
FLS2.Arab.thal.LRR.XII
pac.x.5.1
pac.x.7.4
At3G47580.Arab.thal.LRR.XII
EFR.Arab.thal.LRR.XII
pac.x.5.14
pac.Erf.6
pac.x.5.26
pac.x.5.4
pac.Erf.3
pac.Erf.5
pac.Erf.10
pac.Erf.14
pac.x.6.109
pac.x.6.129
pac.Erf.7
pac.x.6.107
pac.x.6.115
pac.Erf.4
pac.x.6.104
pac.x.5.6
pac.x.6.112
pac.Erf.11
pac.x.5.12
pac.x.6.108
pac.x.6.102
pac.x.6.124
pac.x.5.2
pac.x.5.17
pac.x.6.103
pac.x.5.8
pac.x.6.123
pac.x.5.18
pac.x.5.19
pac.x.5.13
pac.x.5.3
pac.x.5.9
LRR XII-L
pac.x.5.43
pac.x.5.39
At3G28040.Arab.thal.LRR.VII
LRR VII-L
MPK3.Arab.thal.MAPK
CPK7.Arab.thal.CDPK
0.2

A2

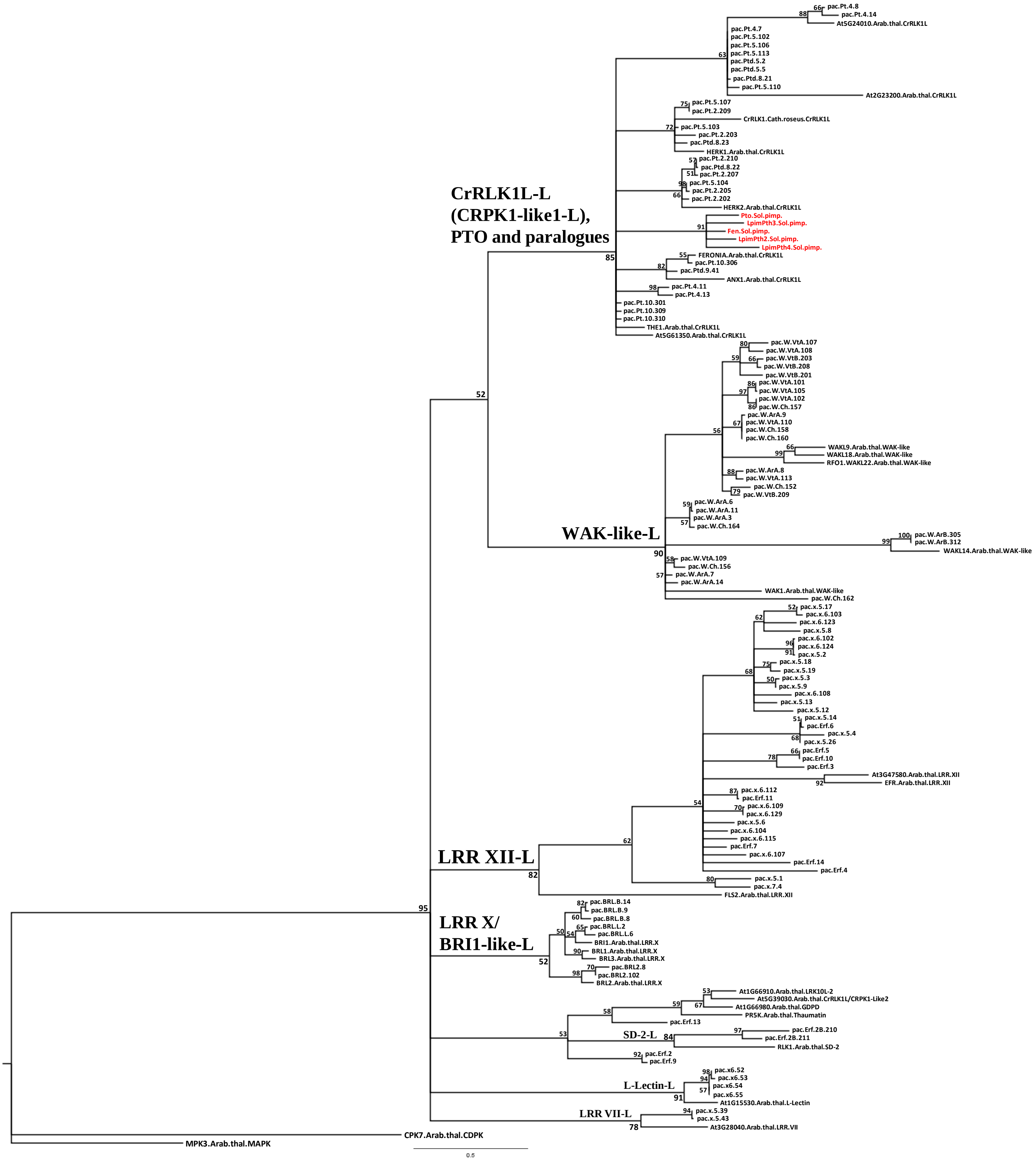

CrRLK1L-L
(CRPK1-like1-L),
PTO and paralogues
WAK-like-L
LRR XII-L
LRR X/
BRI1-like-L
SD-2-L
L-Lectin-L
LRR VII-L
pac.Pt.4.8
pac.Pt.4.14
At5G24010.Arab.thal.CrRLK1L
pac.Pt.4.7
pac.Pt.5.102
pac.Pt.5.106
pac.Pt.5.113
pac.Ptd.5.2
pac.Ptd.5.5
pac.Ptd.8.21
pac.Pt.5.110
At2G23200.Arab.thal.CrRLK1L
pac.Pt.5.107
pac.Pt.2.209
CrRLK1.Cath.roseus.CrRLK1L
pac.Pt.5.103
pac.Pt.2.203
pac.Ptd.8.23
HERK1.Arab.thal.CrRLK1L
pac.Pt.2.210
pac.Ptd.8.22
pac.Pt.2.207
pac.Pt.5.104
pac.Pt.2.205
pac.Pt.2.202
HERK2.Arab.thal.CrRLK1L
Pto.Sol.pimp.
LpimPth3.Sol.pimp.
Fen.Sol.pimp.
LpimPth2.Sol.pimp.
LpimPth4.Sol.pimp.
FERONIA.Arab.thal.CrRLK1L
pac.Pt.10.306
pac.Ptd.9.41
ANX1.Arab.thal.CrRLK1L
pac.Pt.4.11
pac.Pt.4.13
pac.Pt.10.301
pac.Pt.10.309
pac.Pt.10.310
THE1.Arab.thal.CrRLK1L
At5G61350.Arab.thal.CrRLK1L
pac.W.VtA.107
pac.W.VtA.108
pac.W.VtB.203
pac.W.VtB.208
pac.W.VtB.201
pac.W.VtA.101
pac.W.VtA.105
pac.W.VtA.102
pac.W.Ch.157
pac.W.ArA.9
pac.W.VtA.110
pac.W.Ch.158
pac.W.Ch.160
WAKL9.Arab.thal.WAK-like
WAKL18.Arab.thal.WAK-like
RFO1.WAKL22.Arab.thal.WAK-like
pac.W.ArA.8
pac.W.VtA.113
pac.W.Ch.152
pac.W.VtB.209
pac.W.ArA.6
pac.W.ArA.11
pac.W.ArA.3
pac.W.Ch.164
pac.W.ArB.305
pac.W.ArB.312
WAKL14.Arab.thal.WAK-like
pac.W.VtA.109
pac.W.Ch.156
pac.W.ArA.7
pac.W.ArA.14
WAK1.Arab.thal.WAK-like
pac.W.Ch.162
pac.x.5.17
pac.x.6.103
pac.x.6.123
pac.x.5.8
pac.x.6.102
pac.x.6.124
pac.x.5.2
pac.x.5.18
pac.x.5.19
pac.x.5.3
pac.x.5.9
pac.x.6.108
pac.x.5.13
pac.x.5.12
pac.x.5.14
pac.Erf.6
pac.x.5.4
pac.x.5.26
pac.Erf.5
pac.Erf.10
pac.Erf.3
At3G47580.Arab.thal.LRR.XII
EFR.Arab.thal.LRR.XII
pac.x.6.112
pac.Erf.11
pac.x.6.109
pac.x.6.129
pac.x.5.6
pac.x.6.104
pac.x.6.115
pac.Erf.7
pac.x.6.107
pac.Erf.14
pac.Erf.4
pac.x.5.1
pac.x.7.4
FLS2.Arab.thal.LRR.XII
pac.BRL.B.14
pac.BRL.B.9
pac.BRL.B.8
pac.BRL.L.2
pac.BRL.L.6
BRI1.Arab.thal.LRR.X
BRL1.Arab.thal.LRR.X
BRL3.Arab.thal.LRR.X
pac.BRL2.8
pac.BRL2.102
BRL2.Arab.thal.LRR.X
At1G66910.Arab.thal.LRK10L-2
At5G39030.Arab.thal.CrRLK1L/CRPK1-Like2
At1G66980.Arab.thal.GDPD
PR5K.Arab.thal.Thaumatin
pac.Erf.13
pac.Erf.2B.210
pac.Erf.2B.211
RLK1.Arab.thal.SD-2
pac.Erf.2
pac.Erf.9
pac.x6.52
pac.x6.53
pac.x6.54
pac.x6.55
At1G15530.Arab.thal.L-Lectin
pac.x.5.39
pac.x.5.43
At3G28040.Arab.thal.LRR.VII
CPK7.Arab.thal.CDPK
MPK3.Arab.thal.MAPK
0.5

B

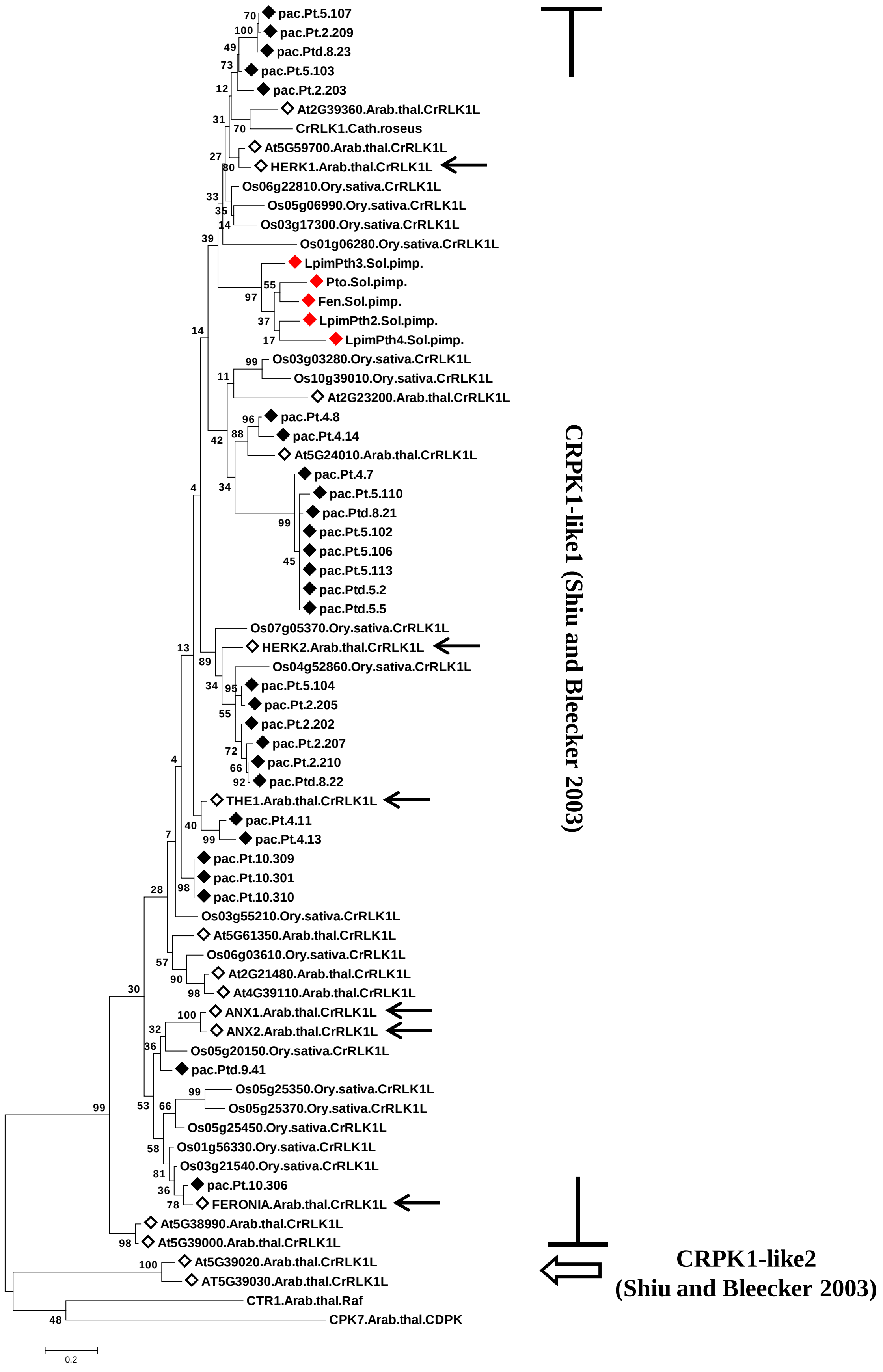

pac.Pt.5.107
pac.Pt.2.209
pac.Ptd.8.23
pac.Pt.5.103
pac.Pt.2.203
At2G39360.Arab.thal.CrRLK1L
CrRLK1.Cath.roseus
At5G59700.Arab.thal.CrRLK1L
HERK1.Arab.thal.CrRLK1L
Os06g22810.Ory.sativa.CrRLK1L
Os05g06990.Ory.sativa.CrRLK1L
Os03g17300.Ory.sativa.CrRLK1L
Os01g06280.Ory.sativa.CrRLK1L
LpimPth3.Sol.pimp.
Pto.Sol.pimp.
Fen.Sol.pimp.
LpimPth2.Sol.pimp.
LpimPth4.Sol.pimp.
Os03g03280.Ory.sativa.CrRLK1L
Os10g39010.Ory.sativa.CrRLK1L
At2G23200.Arab.thal.CrRLK1L
pac.Pt.4.8
pac.Pt.4.14
At5G24010.Arab.thal.CrRLK1L
pac.Pt.4.7
pac.Pt.5.110
pac.Ptd.8.21
pac.Pt.5.102
pac.Pt.5.106
pac.Pt.5.113
pac.Ptd.5.2
pac.Ptd.5.5
Os07g05370.Ory.sativa.CrRLK1L
HERK2.Arab.thal.CrRLK1L
Os04g52860.Ory.sativa.CrRLK1L
pac.Pt.5.104
pac.Pt.2.205
pac.Pt.2.202
pac.Pt.2.207
pac.Pt.2.210
pac.Ptd.8.22
THE1.Arab.thal.CrRLK1L
pac.Pt.4.11
pac.Pt.4.13
pac.Pt.10.309
pac.Pt.10.301
pac.Pt.10.310
Os03g55210.Ory.sativa.CrRLK1L
At5G61350.Arab.thal.CrRLK1L
Os06g03610.Ory.sativa.CrRLK1L
At2G21480.Arab.thal.CrRLK1L
At4G39110.Arab.thal.CrRLK1L
ANX1.Arab.thal.CrRLK1L
ANX2.Arab.thal.CrRLK1L
Os05g20150.Ory.sativa.CrRLK1L
pac.Ptd.9.41
Os05g25350.Ory.sativa.CrRLK1L
Os05g25370.Ory.sativa.CrRLK1L
Os05g25450.Ory.sativa.CrRLK1L
Os01g56330.Ory.sativa.CrRLK1L
Os03g21540.Ory.sativa.CrRLK1L
pac.Pt.10.306
FERONIA.Arab.thal.CrRLK1L
At5G38990.Arab.thal.CrRLK1L
At5G39000.Arab.thal.CrRLK1L
At5G39020.Arab.thal.CrRLK1L
AT5G39030.Arab.thal.CrRLK1L
CTR1.Arab.thal.Raf
CPK7.Arab.thal.CDPK
0.2
CRPK1-like1 (Shiu and Bleecker 2003)
CRPK1-like2
(Shiu and Bleecker 2003)

C

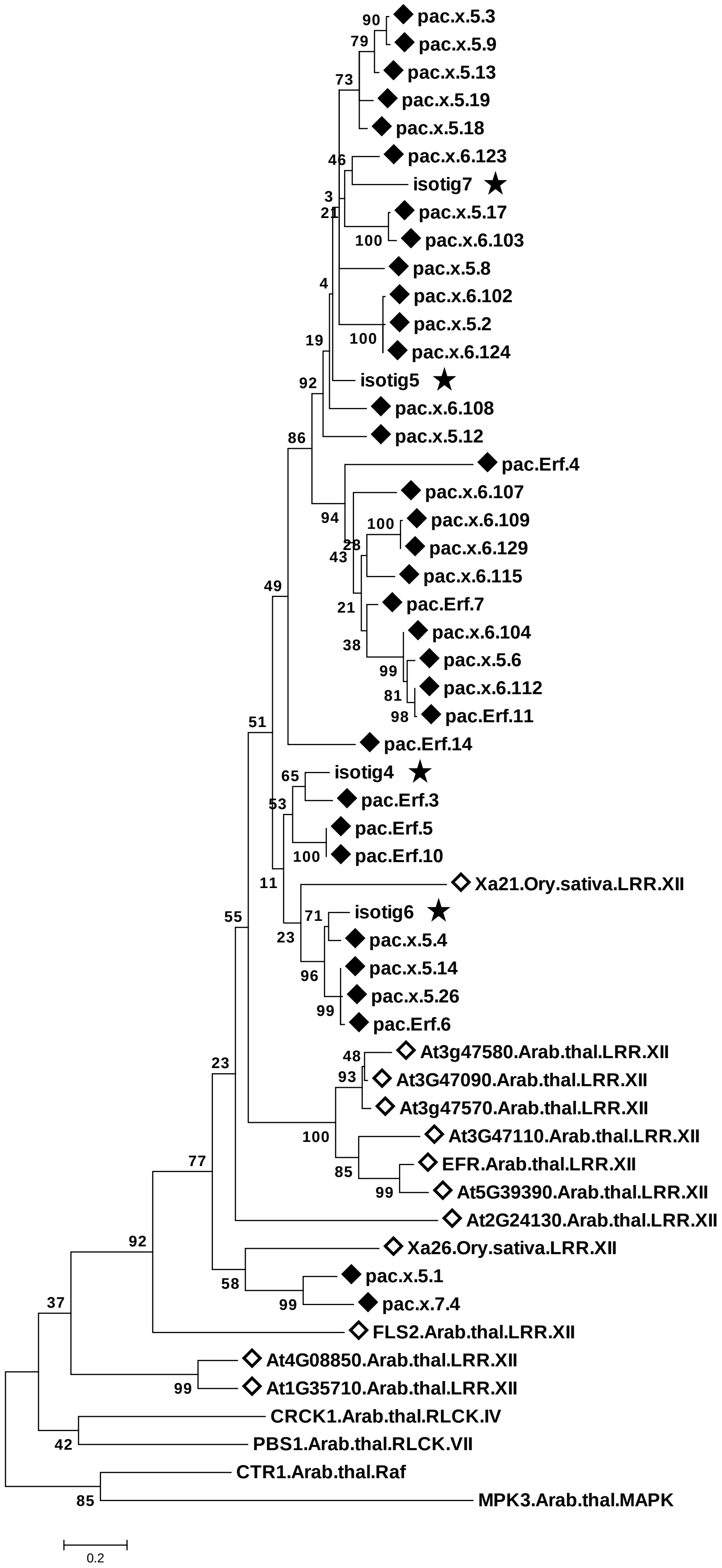
pac.x.5.3
pac.x.5.9
pac.x.5.13
pac.x.5.19
pac.x.5.18
pac.x.6.123
isotig7
pac.x.5.17
pac.x.6.103
pac.x.5.8
pac.x.6.102
pac.x.5.2
pac.x.6.124
isotig5
pac.x.6.108
pac.x.5.12
pac.Erf.4
pac.x.6.107
pac.x.6.109
pac.x.6.129
pac.x.6.115
pac.Erf.7
pac.x.6.104
pac.x.5.6
pac.x.6.112
pac.Erf.11
pac.Erf.14
isotig4
pac.Erf.3
pac.Erf.5
pac.Erf.10
Xa21.Ory.sativa.LRR.XII
isotig6
pac.x.5.4
pac.x.5.14
pac.x.5.26
pac.Erf.6
At3g47580.Arab.thal.LRR.XII
At3G47090.Arab.thal.LRR.XII
At3g47570.Arab.thal.LRR.XII
At3G47110.Arab.thal.LRR.XII
EFR.Arab.thal.LRR.XII
At5G39390.Arab.thal.LRR.XII
At2G24130.Arab.thal.LRR.XII
Xa26.Ory.sativa.LRR.XII
pac.x.5.1
pac.x.7.4
FLS2.Arab.thal.LRR.XII
At4G08850.Arab.thal.LRR.XII
At1G35710.Arab.thal.LRR.XII
CRCK1.Arab.thal.RLCK.IV
PBS1.Arab.thal.RLCK.VII
CTR1.Arab.thal.Raf
MPK3.Arab.thal.MAPK
0.2

D

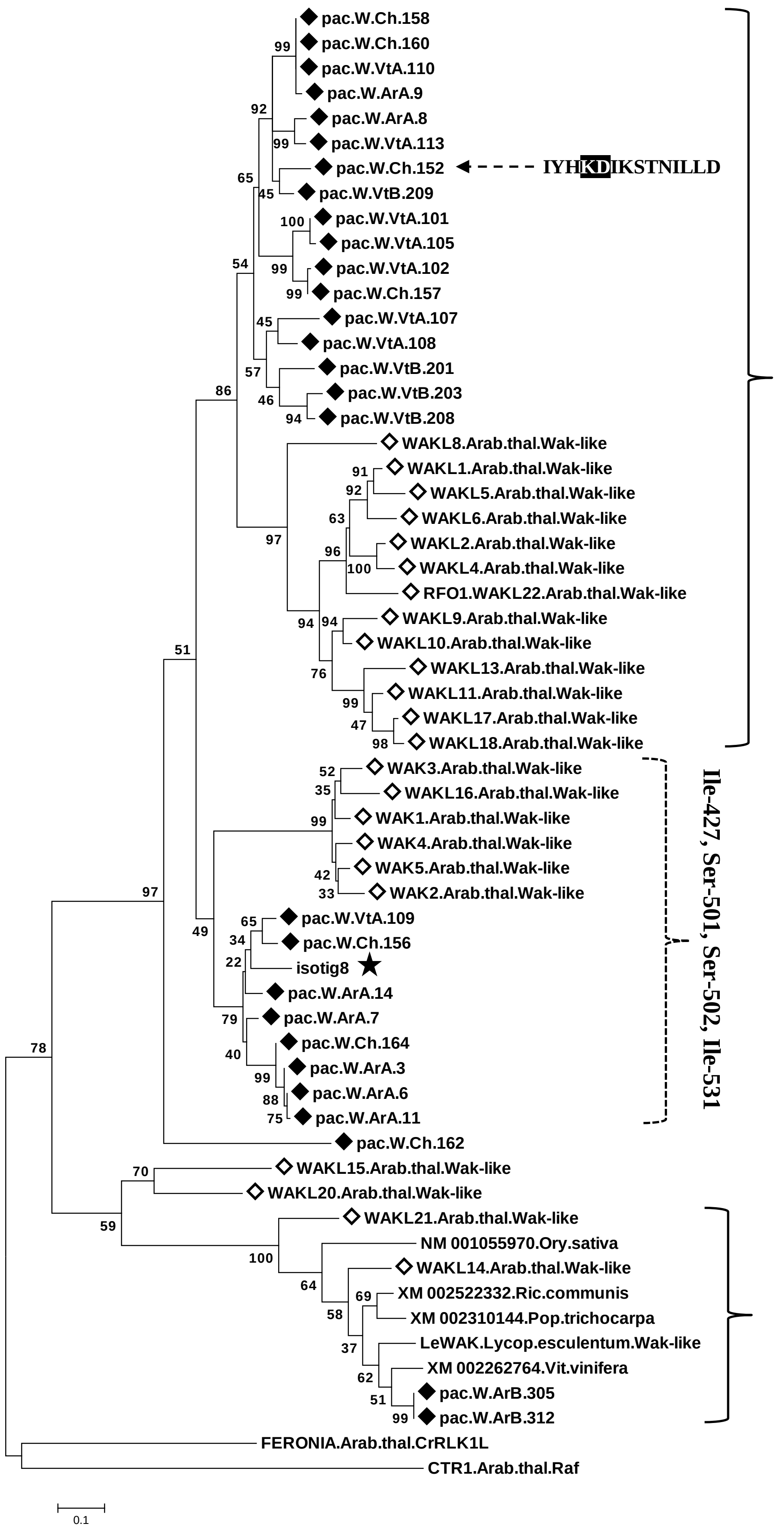

pac.W.Ch.158
pac.W.Ch.160
pac.W.VtA.110
pac.W.ArA.9
pac.W.ArA.8
pac.W.VtA.113
pac.W.Ch.152
IYHKDIKSTNILLD
pac.W.VtB.209
pac.W.VtA.101
pac.W.VtA.105
pac.W.VtA.102
pac.W.Ch.157
pac.W.VtA.107
pac.W.VtA.108
pac.W.VtB.201
pac.W.VtB.203
pac.W.VtB.208
WAKL8.Arab.thal.Wak-like
WAKL1.Arab.thal.Wak-like
WAKL5.Arab.thal.Wak-like
WAKL6.Arab.thal.Wak-like
WAKL2.Arab.thal.Wak-like
WAKL4.Arab.thal.Wak-like
RFO1.WAKL22.Arab.thal.Wak-like
WAKL9.Arab.thal.Wak-like
WAKL10.Arab.thal.Wak-like
WAKL13.Arab.thal.Wak-like
WAKL11.Arab.thal.Wak-like
WAKL17.Arab.thal.Wak-like
WAKL18.Arab.thal.Wak-like
WAK3.Arab.thal.Wak-like
WAKL16.Arab.thal.Wak-like
WAK1.Arab.thal.Wak-like
WAK4.Arab.thal.Wak-like
WAK5.Arab.thal.Wak-like
WAK2.Arab.thal.Wak-like
pac.W.VtA.109
pac.W.Ch.156
isotig8
pac.W.ArA.14
pac.W.ArA.7
pac.W.Ch.164
pac.W.ArA.3
pac.W.ArA.6
pac.W.ArA.11
pac.W.Ch.162
Ile-427, Ser-501, Ser-502, Ile-531
WAKL15.Arab.thal.Wak-like
WAKL20.Arab.thal.Wak-like
WAKL21.Arab.thal.Wak-like
NM 001055970.Ory.sativa
WAKL14.Arab.thal.Wak-like
XM 002522332.Ric.communis
XM 002310144.Pop.trichocarpa
LeWAK.Lycop.esculentum.Wak-like
XM 002262764.Vit.vinifera
pac.W.ArB.305
pac.W.ArB.312
FERONIA.Arab.thal.CrRLK1L
CTR1.Arab.thal.Raf
0.1

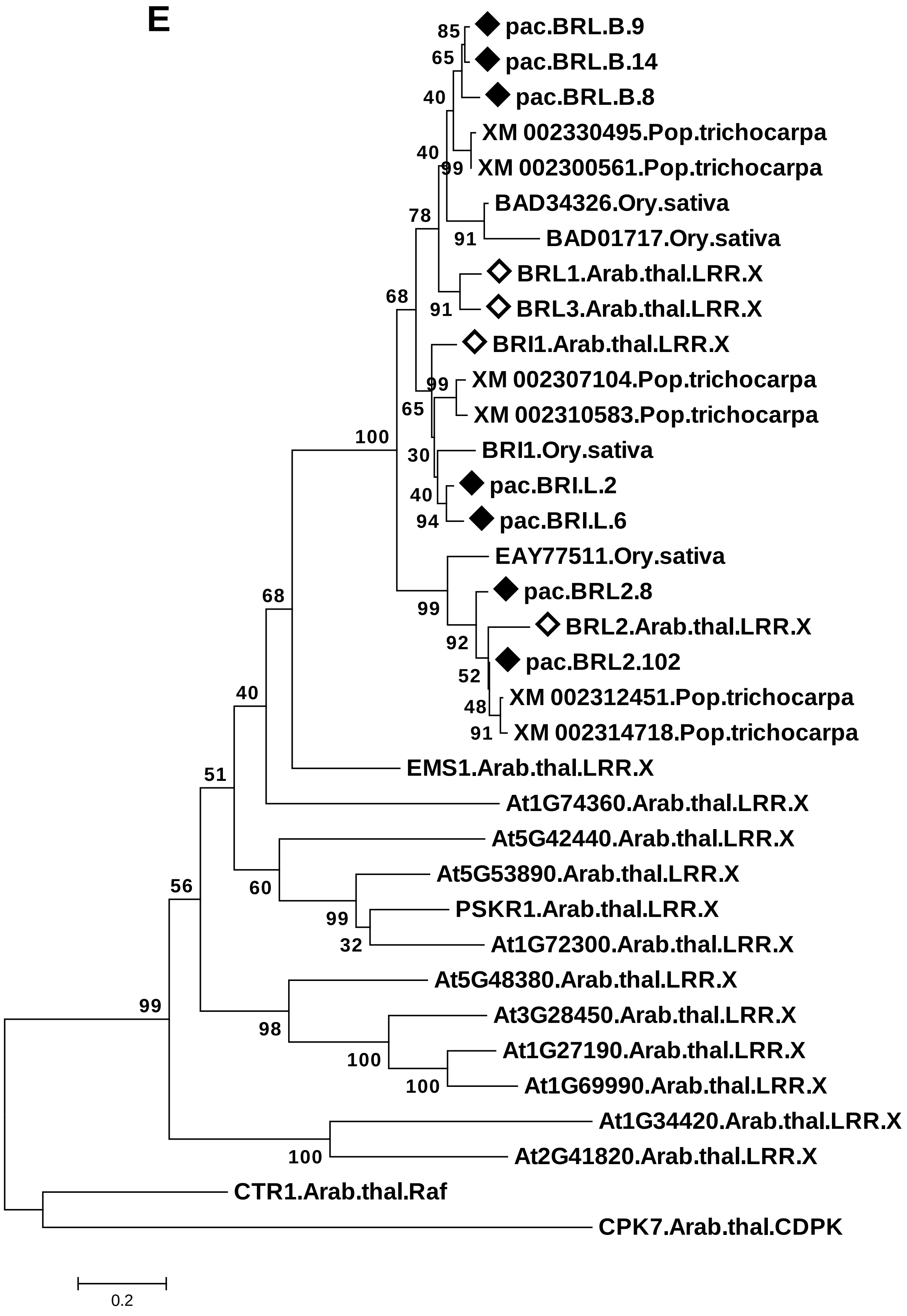
E
pac.BRL.B.9
pac.BRL.B.14
pac.BRL.B.8
XM 002330495.Pop.trichocarpa
XM 002300561.Pop.trichocarpa
BAD34326.Ory.sativa
BAD01717.Ory.sativa
BRL1.Arab.thal.LRR.X
BRL3.Arab.thal.LRR.X
BRI1.Arab.thal.LRR.X
XM 002307104.Pop.trichocarpa
XM 002310583.Pop.trichocarpa
BRI1.Ory.sativa
pac.BRI.L.2
pac.BRI.L.6
EAY77511.Ory.sativa
pac.BRL2.8
BRL2.Arab.thal.LRR.X
pac.BRL2.102
XM 002312451.Pop.trichocarpa
XM 002314718.Pop.trichocarpa
EMS1.Arab.thal.LRR.X
At1G74360.Arab.thal.LRR.X
At5G42440.Arab.thal.LRR.X
At5G53890.Arab.thal.LRR.X
PSKR1.Arab.thal.LRR.X
At1G72300.Arab.thal.LRR.X
At5G48380.Arab.thal.LRR.X
At3G28450.Arab.thal.LRR.X
At1G27190.Arab.thal.LRR.X
At1G69990.Arab.thal.LRR.X
At1G34420.Arab.thal.LRR.X
At2G41820.Arab.thal.LRR.X
CTR1.Arab.thal.Raf
CPK7.Arab.thal.CDPK
85
65
40
40
99
78
91
68
91
99
65
100
30
40
94
99
92
52
48
91
68
40
51
60
99
32
56
98
100
100
99
100
0.2
