# Supplementary material for: Kinase domain-targeted isolation of defense-related receptor-like kinases (RLK/Pelle) in Platanus × acerifolia: phylogenetic and structural analysis
Source: BMC Res Notes. 2014 Dec 8;7:884. doi: 10.1186/1756-0500-7-884 (PMC4295470; doi:10.1186/1756-0500-7-884)
Supplement: Supplementary file 11 — Additional file 11: Phylogenetic relationship between Pto (−like) ( sensu strictu and sensu latu ) and the subfamily CrRLK1L by two methods of maximum-likelihood analysis. Analysis was performed on the same dataset alignment used for the analysis presented in Figure 5. The following aa sequences were included: Pto locus and solanaceous Pto-like; CrRLK1L of Arabidopsis, rice and Catharanthus roseus; Pto best blastp matches (namely CrRLK1L, based on domain composition) of Arabidopsis, rice, grapevine, poplar and castor bean; Pto-like partial sequences (namely CrRLK1L-like, i.e. Pto-like sensu latu) of non-solanaceous species (i.e. Phaselus vulgaris, cultivated and wild strawberry and Musa acuminata); best blastp matches from F. vesca and P. vulgaris (namely CrRLK1L) obtained using Pto-like partial sequences as query. Representative Pac sequences obtained with Pto-primer (CrRLK1L-like) were also included (pac). The arrows indicate solanaceous so-called Pto-like sequences, which clustered within a CrRLK1L-headed clade. The star indicates a CrRLK1L-like of castor bean lacking the extracellular domain typical of the subfamily. A: tree obtained with Mega 6; the percentage of trees in which the associated sequences clustered together in the bootstrap test (1,000 replicates) is shown for each node; trees are drawn to scale in the number of substitutions per site (scale at the bottom). B: tree obtained with TREE-PUZZLE; analysis was conducted with 50,000 puzzling steps and quartet puzzling support values are shown for each node. See Methods for details. (PDF 115 KB) [file 13104_2014_3456_MOESM11_ESM.pdf]

Additional file 11. Phylogenetic relationship between Pto (-like) (*sensu strictu* and *sensu latu*) and the subfamily CrRLK1L by two methods of maximum-likelihood analysis. Analysis was performed on the same dataset alignment used for the analysis presented in Fig. 5. The following aa sequences were included: Pto locus and solanaceous Pto-like; CrRLK1L of *Arabidopsis*, rice and *Catharanthus roseus*; Pto best blastp matches (namely CrRLK1L, based on domain composition) of *Arabidopsis*, rice, grapevine, poplar and castor bean; Pto-like partial sequences (namely CrRLK1L-like, i.e. Pto-like *sensu latu*) of non-solanaceous species (i.e. *Phaselus vulgaris*, cultivated and wild strawberry and *Musa acuminata*); best blastp matches from *F. vesca* and *P. vulgaris* (namely CrRLK1L) obtained using Pto-like partial sequences as query. Representative *Pac* sequences obtained with *Pto*-primer (CrRLK1L-like) were also included (pac). The arrows indicate solanaceous *so-called* Pto-like sequences, which clustered within a CrRLK1L-headed clade. The star indicates a CrRLK1L-like of castor bean lacking the extracellular domain typical of the subfamily.

A: tree obtained with Mega 6; the percentage of trees in which the associated sequences clustered together in the bootstrap test (1,000 replicates) is shown for each node; trees are drawn to scale in the number of substitutions per site (scale at the bottom).

B: tree obtained with TREE-PUZZLE; analysis was conducted with 50,000 puzzling steps and quartet puzzling support values are shown for each node.

See Methods for details.

A

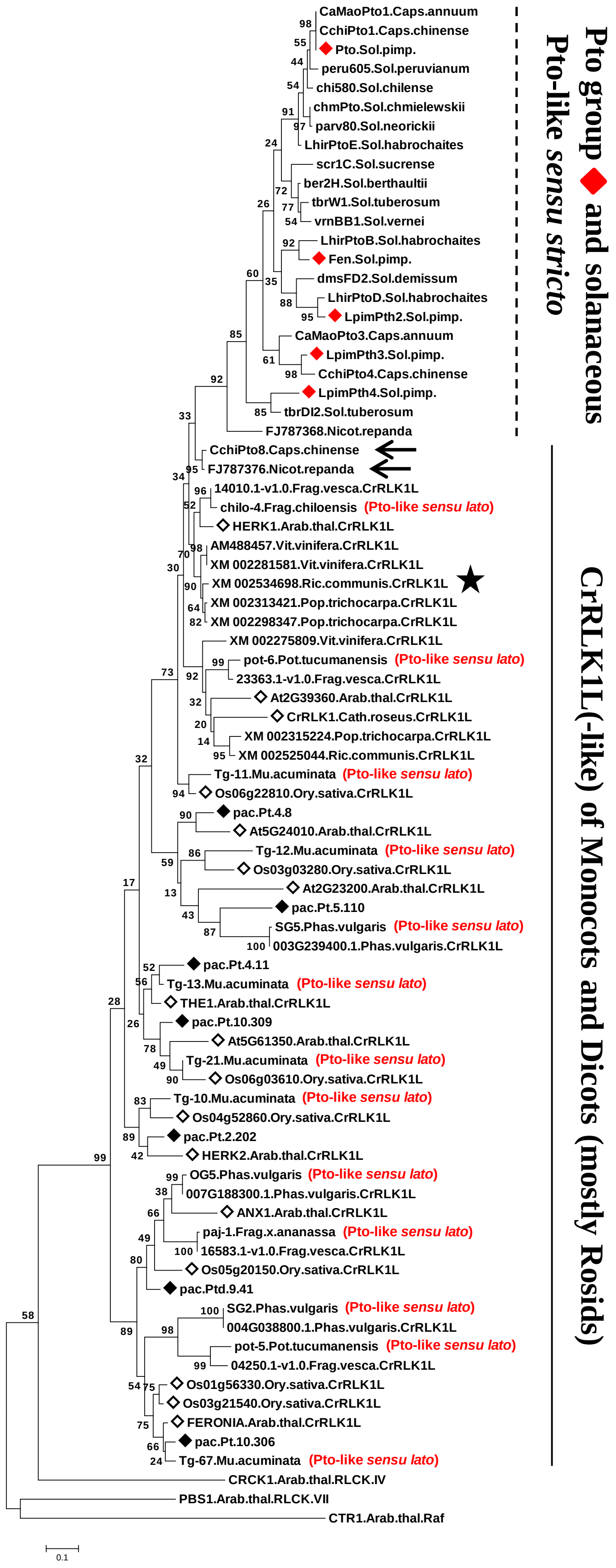

CaMaoPto1.Caps.annuum
CchiPto1.Caps.chinense
Pto.Sol.pimp.
peru605.Sol.peruvianum
chi580.Sol.chilense
chmPto.Sol.chmielewskii
parv80.Sol.neorickii
LhirPtoE.Sol.habrochaites
scr1C.Sol.sucrense
ber2H.Sol.berthaultii
tbrW1.Sol.tuberosum
vrnBB1.Sol.vernei
LhirPtoB.Sol.habrochaites
Fen.Sol.pimp.
dmsFD2.Sol.demissum
LhirPtoD.Sol.habrochaites
LpimPth2.Sol.pimp.
CaMaoPto3.Caps.annuum
LpimPth3.Sol.pimp.
CchiPto4.Caps.chinense
LpimPth4.Sol.pimp.
tbrDI2.Sol.tuberosum
FJ787368.Nicot.repanda
CchiPto8.Caps.chinense
FJ787376.Nicot.repanda
14010.1-v1.0.Frag.vesca.CrRLK1L
chilo-4.Frag.chiloensis (Pto-like sensu lato)
HERK1.Arab.thal.CrRLK1L
AM488457.Vit.vinifera.CrRLK1L
XM 002281581.Vit.vinifera.CrRLK1L
XM 002534698.Ric.communis.CrRLK1L
XM 002313421.Pop.trichocarpa.CrRLK1L
XM 002298347.Pop.trichocarpa.CrRLK1L
XM 002275809.Vit.vinifera.CrRLK1L
pot-6.Pot.tucumanensis (Pto-like sensu lato)
23363.1-v1.0.Frag.vesca.CrRLK1L
At2G39360.Arab.thal.CrRLK1L
CrRLK1.Cath.roseus.CrRLK1L
XM 002315224.Pop.trichocarpa.CrRLK1L
XM 002525044.Ric.communis.CrRLK1L
Tg-11.Mu.acuminata (Pto-like sensu lato)
Os06g22810.Ory.sativa.CrRLK1L
pac.Pt.4.8
At5G24010.Arab.thal.CrRLK1L
Tg-12.Mu.acuminata (Pto-like sensu lato)
Os03g03280.Ory.sativa.CrRLK1L
At2G23200.Arab.thal.CrRLK1L
pac.Pt.5.110
SG5.Phas.vulgaris (Pto-like sensu lato)
003G239400.1.Phas.vulgaris.CrRLK1L
pac.Pt.4.11
Tg-13.Mu.acuminata (Pto-like sensu lato)
THE1.Arab.thal.CrRLK1L
pac.Pt.10.309
At5G61350.Arab.thal.CrRLK1L
Tg-21.Mu.acuminata (Pto-like sensu lato)
Os06g03610.Ory.sativa.CrRLK1L
Tg-10.Mu.acuminata (Pto-like sensu lato)
Os04g52860.Ory.sativa.CrRLK1L
pac.Pt.2.202
HERK2.Arab.thal.CrRLK1L
OG5.Phas.vulgaris (Pto-like sensu lato)
007G188300.1.Phas.vulgaris.CrRLK1L
ANX1.Arab.thal.CrRLK1L
paj-1.Frag.x.ananassa (Pto-like sensu lato)
16583.1-v1.0.Frag.vesca.CrRLK1L
Os05g20150.Ory.sativa.CrRLK1L
pac.Ptd.9.41
SG2.Phas.vulgaris (Pto-like sensu lato)
004G038800.1.Phas.vulgaris.CrRLK1L
pot-5.Pot.tucumanensis (Pto-like sensu lato)
04250.1-v1.0.Frag.vesca.CrRLK1L
Os01g56330.Ory.sativa.CrRLK1L
Os03g21540.Ory.sativa.CrRLK1L
FERONIA.Arab.thal.CrRLK1L
pac.Pt.10.306
Tg-67.Mu.acuminata (Pto-like sensu lato)
CRCK1.Arab.thal.RLCK.IV
PBS1.Arab.thal.RLCK.VII
CTR1.Arab.thal.Raf
0.1
Pto group and solanaceous
Pto-like sensu stricto
CrRLK1L(-like) of Monocots and Dicots (mostly Rosids)

B

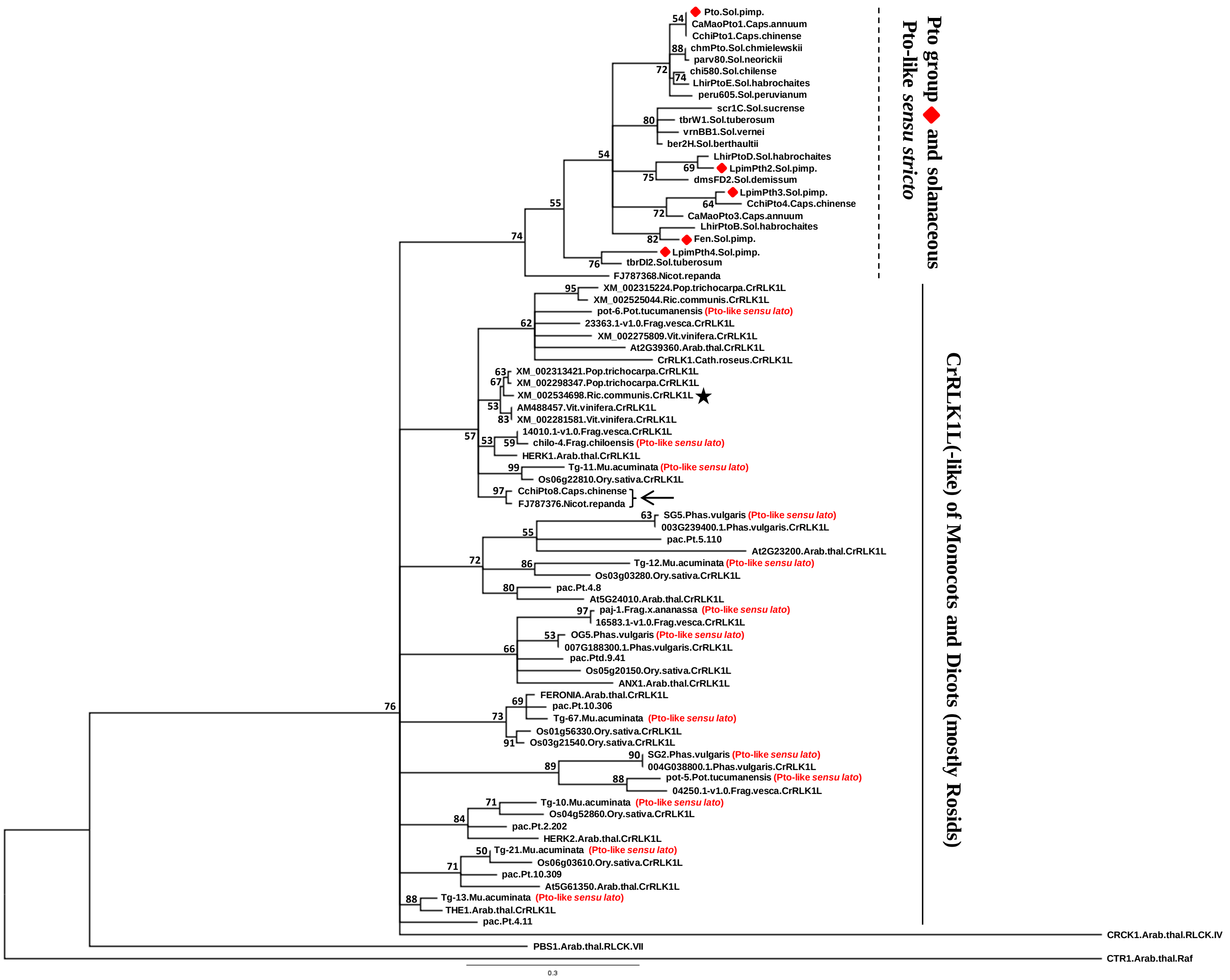

Pto group and solanaceous
Pto-like sensu stricto
CrRLK1L(-like) of Monocots and Dicots (mostly Rosids)
Pto.Sol.pimp.
CaMaoPto1.Caps.annuum
CchiPto1.Caps.chinense
chmPto.Sol.chmielewskii
parv80.Sol.neorickii
chi580.Sol.chilense
LhirPtoE.Sol.habrochaites
peru605.Sol.peruvianum
scr1C.Sol.sucrense
tbrW1.Sol.tuberosum
vrnBB1.Sol.vernei
ber2H.Sol.berthaultii
LhirPtoD.Sol.habrochaites
LpimPth2.Sol.pimp.
dmsFD2.Sol.demissum
LpimPth3.Sol.pimp.
CchiPto4.Caps.chinense
CaMaoPto3.Caps.annuum
LhirPtoB.Sol.habrochaites
Fen.Sol.pimp.
LpimPth4.Sol.pimp.
tbrDl2.Sol.tuberosum
FJ787368.Nicot.repanda
XM_002315224.Pop.trichocarpa.CrRLK1L
XM_002525044.Ric.communis.CrRLK1L
pot-6.Pot.tucumanensis (Pto-like sensu lato)
23363.1-v1.0.Frag.vesca.CrRLK1L
XM_002275809.Vit.vinifera.CrRLK1L
At2G39360.Arab.thal.CrRLK1L
CrRLK1.Cath.roseus.CrRLK1L
XM_002313421.Pop.trichocarpa.CrRLK1L
XM_002298347.Pop.trichocarpa.CrRLK1L
XM_002534698.Ric.communis.CrRLK1L
AM488457.Vit.vinifera.CrRLK1L
XM_002281581.Vit.vinifera.CrRLK1L
14010.1-v1.0.Frag.vesca.CrRLK1L
chilo-4.Frag.chiloensis (Pto-like sensu lato)
HERK1.Arab.thal.CrRLK1L
Tg-11.Mu.acuminata (Pto-like sensu lato)
Os06g22810.Ory.sativa.CrRLK1L
CchiPto8.Caps.chinense
FJ787376.Nicot.repanda
SG5.Phas.vulgaris (Pto-like sensu lato)
003G239400.1.Phas.vulgaris.CrRLK1L
pac.Pt.5.110
At2G23200.Arab.thal.CrRLK1L
Tg-12.Mu.acuminata (Pto-like sensu lato)
Os03g03280.Ory.sativa.CrRLK1L
pac.Pt.4.8
At5G24010.Arab.thal.CrRLK1L
paj-1.Frag.x.ananassa (Pto-like sensu lato)
16583.1-v1.0.Frag.vesca.CrRLK1L
OG5.Phas.vulgaris (Pto-like sensu lato)
007G188300.1.Phas.vulgaris.CrRLK1L
pac.Ptd.9.41
Os05g20150.Ory.sativa.CrRLK1L
ANX1.Arab.thal.CrRLK1L
FERONIA.Arab.thal.CrRLK1L
pac.Pt.10.306
Tg-67.Mu.acuminata (Pto-like sensu lato)
Os01g56330.Ory.sativa.CrRLK1L
Os03g21540.Ory.sativa.CrRLK1L
SG2.Phas.vulgaris (Pto-like sensu lato)
004G038800.1.Phas.vulgaris.CrRLK1L
pot-5.Pot.tucumanensis (Pto-like sensu lato)
04250.1-v1.0.Frag.vesca.CrRLK1L
Tg-10.Mu.acuminata (Pto-like sensu lato)
Os04g52860.Ory.sativa.CrRLK1L
pac.Pt.2.202
HERK2.Arab.thal.CrRLK1L
Tg-21.Mu.acuminata (Pto-like sensu lato)
Os06g03610.Ory.sativa.CrRLK1L
pac.Pt.10.309
At5G61350.Arab.thal.CrRLK1L
Tg-13.Mu.acuminata (Pto-like sensu lato)
THE1.Arab.thal.CrRLK1L
pac.Pt.4.11
CRCK1.Arab.thal.RLCK.IV
PBS1.Arab.thal.RLCK.VII
CTR1.Arab.thal.Raf
0.3
