# Supplementary material for: Kinase domain-targeted isolation of defense-related receptor-like kinases (RLK/Pelle) in Platanus × acerifolia: phylogenetic and structural analysis
Source: BMC Res Notes. 2014 Dec 8;7:884. doi: 10.1186/1756-0500-7-884 (PMC4295470; doi:10.1186/1756-0500-7-884)
Supplement: Supplementary file 12 — Additional file 12: Phylogenetic analyses (NJ and ML) to compare the complete CrRLK1L subfamily (CRPK1-like 1) of different plant species and Pto group. We included the complete CRPK1-like 1 aa sequence set of Arabidopsis, poplar, tomato (Solanum lycopersicum), rice and moss, and Pto and paralogs of Solanum pimpinellifolium (see Additional file 4 for sequence sources). The NJ tree is presented in A, the ML trees, Mega6 and TREE-PUZZLE-implemented, are presented in B and C respectively. Analyses were based on the complete kinase domain region. With regard to Mega 6, the percentage of trees in which the associated sequences clustered together in the bootstrap test (1,000 replicates) is shown for each node; trees are drawn to scale in the number of substitutions per site (scale at the bottom). With regard to TREE-PUZZLE analysis was conducted with 50,000 puzzling steps and quartet puzzling support values are shown for each node. See Methods for additional details. (PDF 220 KB) [file 13104_2014_3456_MOESM12_ESM.pdf]

Additional file 12. Phylogenetic analyses (NJ and ML) to compare the complete CrRLK1L subfamily (CRPK1-like 1) of different plant species and Pto group. We included the complete CRPK1-like 1 aa sequence set of *Arabidopsis*, poplar, tomato (*Solanum lycopersicum*), rice and moss, and Pto and paralogs of *Solanum pimpinellifolium* (see additional file 4 for sequence sources). The NJ tree (Mega 6) is presented in A, the ML trees, Mega6 and TREE-PUZZLE-implemented, are presented respectively in B and C.

Analyses were based on the complete kinase domain region. With regard to Mega 6, the percentage of trees in which the associated sequences clustered together in the bootstrap test (1,000 replicates) is shown for each node; trees are drawn to scale in the number of substitutions per site (scale at the bottom). With regard to TREE-PUZZLE, analysis was conducted with 50,000 puzzling steps and quartet puzzling support values are shown for each node. See Methods for additional details.

Introduction

Note that CRPK1-like 1 set of *S. lycopersicum* included the Pto-like paralogs, confirming results by Sakamoto et al. [6]. Brackets with roman numerals indicates highly bootstrapped multi-species clades confirming that, frequently, in CrRLK1L, ortholgs are more similar than paralogs [5, 50]. Specifically: we identified the clades in the NJ tree (A) and visualized them with a bracket, then we marked the corresponding clades in the ML trees (Mega 6 and TREE-PUZZLE-implemented, i.e. B and C respectively). In B and C dotted brackets mean that the correspondence with clades in the NJ tree is somewhat rough. Note that, in general, the multi-species phylogenetic clades are fully conserved in the three analyses.

However the major focus of this additional file is that, differently from multi-species clades, Pto and paralogs of *S. pimpinellifolium* and *S. lycopersicum* (♦ in red) represent a solanaceous-specific CrRLK1L-like clade. The phylogenetic vicinity of Pto group to CrRLK1L subfamily has been also shown in Figure 1 and 5, and in Additional files 6, 7, 10, 11 and is evident, though not discussed by the authors, in the phylogenetic tree of tomato RLK/Pelles by Sakamoto et al. [6]. The phylogenies also shows the expansion of the subfamily in vascular plants, compared with moss.

A

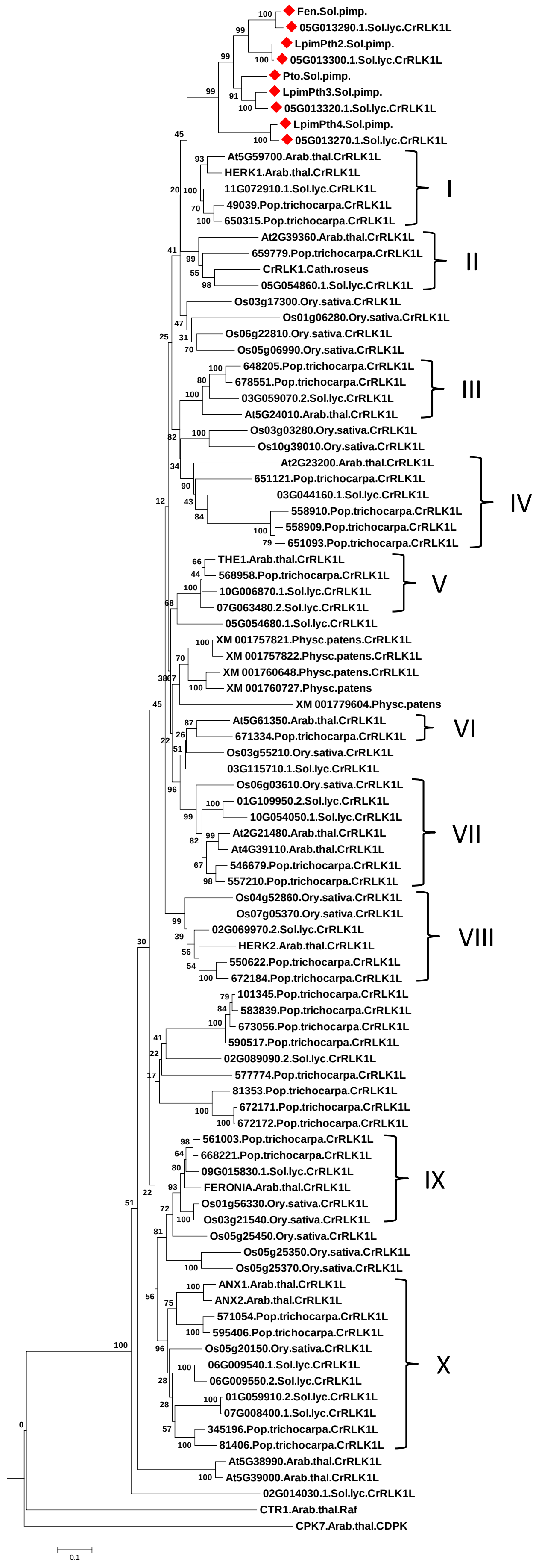

Fen.Sol.pimp.
05G013290.1.Sol.lyc.CrRLK1L
LpimPth2.Sol.pimp.
05G013300.1.Sol.lyc.CrRLK1L
Pto.Sol.pimp.
LpimPth3.Sol.pimp.
05G013320.1.Sol.lyc.CrRLK1L
LpimPth4.Sol.pimp.
05G013270.1.Sol.lyc.CrRLK1L
At5G59700.Arab.thal.CrRLK1L
HERK1.Arab.thal.CrRLK1L
11G072910.1.Sol.lyc.CrRLK1L
49039.Pop.trichocarpa.CrRLK1L
650315.Pop.trichocarpa.CrRLK1L
I
At2G39360.Arab.thal.CrRLK1L
659779.Pop.trichocarpa.CrRLK1L
CrRLK1.Cath.roseus
05G054860.1.Sol.lyc.CrRLK1L
II
Os03g17300.Ory.sativa.CrRLK1L
Os01g06280.Ory.sativa.CrRLK1L
Os06g22810.Ory.sativa.CrRLK1L
Os05g06990.Ory.sativa.CrRLK1L
648205.Pop.trichocarpa.CrRLK1L
678551.Pop.trichocarpa.CrRLK1L
03G059070.2.Sol.lyc.CrRLK1L
At5G24010.Arab.thal.CrRLK1L
III
Os03g03280.Ory.sativa.CrRLK1L
Os10g39010.Ory.sativa.CrRLK1L
At2G23200.Arab.thal.CrRLK1L
651121.Pop.trichocarpa.CrRLK1L
03G044160.1.Sol.lyc.CrRLK1L
558910.Pop.trichocarpa.CrRLK1L
558909.Pop.trichocarpa.CrRLK1L
651093.Pop.trichocarpa.CrRLK1L
IV
THE1.Arab.thal.CrRLK1L
568958.Pop.trichocarpa.CrRLK1L
10G006870.1.Sol.lyc.CrRLK1L
07G063480.2.Sol.lyc.CrRLK1L
V
05G054680.1.Sol.lyc.CrRLK1L
XM 001757821.Physc.patens.CrRLK1L
XM 001757822.Physc.patens.CrRLK1L
XM 001760648.Physc.patens.CrRLK1L
XM 001760727.Physc.patens
XM 001779604.Physc.patens
At5G61350.Arab.thal.CrRLK1L
671334.Pop.trichocarpa.CrRLK1L
VI
Os03g55210.Ory.sativa.CrRLK1L
03G115710.1.Sol.lyc.CrRLK1L
Os06g03610.Ory.sativa.CrRLK1L
01G109950.2.Sol.lyc.CrRLK1L
10G054050.1.Sol.lyc.CrRLK1L
At2G21480.Arab.thal.CrRLK1L
At4G39110.Arab.thal.CrRLK1L
546679.Pop.trichocarpa.CrRLK1L
557210.Pop.trichocarpa.CrRLK1L
VII
Os04g52860.Ory.sativa.CrRLK1L
Os07g05370.Ory.sativa.CrRLK1L
02G069970.2.Sol.lyc.CrRLK1L
HERK2.Arab.thal.CrRLK1L
550622.Pop.trichocarpa.CrRLK1L
672184.Pop.trichocarpa.CrRLK1L
VIII
101345.Pop.trichocarpa.CrRLK1L
583839.Pop.trichocarpa.CrRLK1L
673056.Pop.trichocarpa.CrRLK1L
590517.Pop.trichocarpa.CrRLK1L
02G089090.2.Sol.lyc.CrRLK1L
577774.Pop.trichocarpa.CrRLK1L
81353.Pop.trichocarpa.CrRLK1L
672171.Pop.trichocarpa.CrRLK1L
672172.Pop.trichocarpa.CrRLK1L
561003.Pop.trichocarpa.CrRLK1L
668221.Pop.trichocarpa.CrRLK1L
09G015830.1.Sol.lyc.CrRLK1L
FERONIA.Arab.thal.CrRLK1L
Os01g56330.Ory.sativa.CrRLK1L
Os03g21540.Ory.sativa.CrRLK1L
IX
Os05g25450.Ory.sativa.CrRLK1L
Os05g25350.Ory.sativa.CrRLK1L
Os05g25370.Ory.sativa.CrRLK1L
ANX1.Arab.thal.CrRLK1L
ANX2.Arab.thal.CrRLK1L
571054.Pop.trichocarpa.CrRLK1L
595406.Pop.trichocarpa.CrRLK1L
Os05g20150.Ory.sativa.CrRLK1L
06G009540.1.Sol.lyc.CrRLK1L
06G009550.2.Sol.lyc.CrRLK1L
01G059910.2.Sol.lyc.CrRLK1L
07G008400.1.Sol.lyc.CrRLK1L
345196.Pop.trichocarpa.CrRLK1L
81406.Pop.trichocarpa.CrRLK1L
X
At5G38990.Arab.thal.CrRLK1L
At5G39000.Arab.thal.CrRLK1L
02G014030.1.Sol.lyc.CrRLK1L
CTR1.Arab.thal.Raf
CPK7.Arab.thal.CDPK
0.1

B

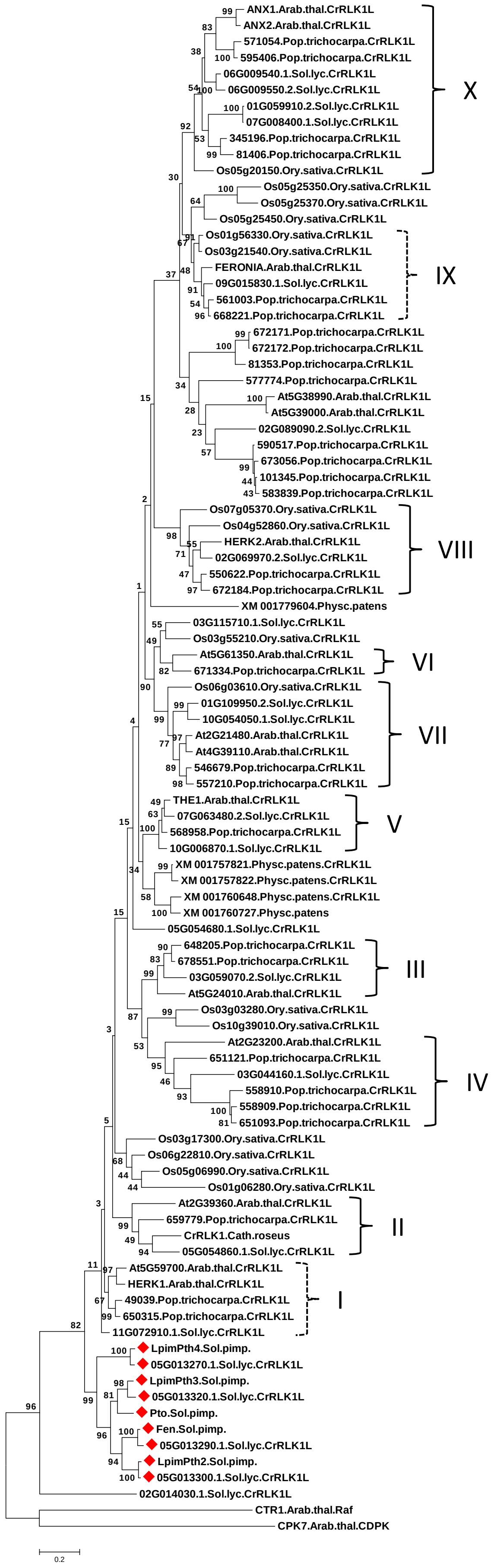

ANX1.Arab.thal.CrRLK1L
ANX2.Arab.thal.CrRLK1L
571054.Pop.trichocarpa.CrRLK1L
595406.Pop.trichocarpa.CrRLK1L
06G009540.1.Sol.lyc.CrRLK1L
06G009550.2.Sol.lyc.CrRLK1L
01G059910.2.Sol.lyc.CrRLK1L
07G008400.1.Sol.lyc.CrRLK1L
345196.Pop.trichocarpa.CrRLK1L
81406.Pop.trichocarpa.CrRLK1L
Os05g20150.Ory.sativa.CrRLK1L
X
Os05g25350.Ory.sativa.CrRLK1L
Os05g25370.Ory.sativa.CrRLK1L
Os05g25450.Ory.sativa.CrRLK1L
Os01g56330.Ory.sativa.CrRLK1L
Os03g21540.Ory.sativa.CrRLK1L
FERONIA.Arab.thal.CrRLK1L
09G015830.1.Sol.lyc.CrRLK1L
561003.Pop.trichocarpa.CrRLK1L
668221.Pop.trichocarpa.CrRLK1L
IX
672171.Pop.trichocarpa.CrRLK1L
672172.Pop.trichocarpa.CrRLK1L
81353.Pop.trichocarpa.CrRLK1L
577774.Pop.trichocarpa.CrRLK1L
At5G38990.Arab.thal.CrRLK1L
At5G39000.Arab.thal.CrRLK1L
02G089090.2.Sol.lyc.CrRLK1L
590517.Pop.trichocarpa.CrRLK1L
673056.Pop.trichocarpa.CrRLK1L
101345.Pop.trichocarpa.CrRLK1L
583839.Pop.trichocarpa.CrRLK1L
Os07g05370.Ory.sativa.CrRLK1L
Os04g52860.Ory.sativa.CrRLK1L
HERK2.Arab.thal.CrRLK1L
02G069970.2.Sol.lyc.CrRLK1L
550622.Pop.trichocarpa.CrRLK1L
672184.Pop.trichocarpa.CrRLK1L
VIII
XM 001779604.Physc.patens
03G115710.1.Sol.lyc.CrRLK1L
Os03g55210.Ory.sativa.CrRLK1L
At5G61350.Arab.thal.CrRLK1L
671334.Pop.trichocarpa.CrRLK1L
VI
Os06g03610.Ory.sativa.CrRLK1L
01G109950.2.Sol.lyc.CrRLK1L
10G054050.1.Sol.lyc.CrRLK1L
At2G21480.Arab.thal.CrRLK1L
At4G39110.Arab.thal.CrRLK1L
546679.Pop.trichocarpa.CrRLK1L
557210.Pop.trichocarpa.CrRLK1L
VII
THE1.Arab.thal.CrRLK1L
07G063480.2.Sol.lyc.CrRLK1L
568958.Pop.trichocarpa.CrRLK1L
10G006870.1.Sol.lyc.CrRLK1L
V
XM 001757821.Physc.patens.CrRLK1L
XM 001757822.Physc.patens.CrRLK1L
XM 001760648.Physc.patens.CrRLK1L
XM 001760727.Physc.patens
05G054680.1.Sol.lyc.CrRLK1L
648205.Pop.trichocarpa.CrRLK1L
678551.Pop.trichocarpa.CrRLK1L
03G059070.2.Sol.lyc.CrRLK1L
At5G24010.Arab.thal.CrRLK1L
III
Os03g03280.Ory.sativa.CrRLK1L
Os10g39010.Ory.sativa.CrRLK1L
At2G23200.Arab.thal.CrRLK1L
651121.Pop.trichocarpa.CrRLK1L
03G044160.1.Sol.lyc.CrRLK1L
558910.Pop.trichocarpa.CrRLK1L
558909.Pop.trichocarpa.CrRLK1L
651093.Pop.trichocarpa.CrRLK1L
IV
Os03g17300.Ory.sativa.CrRLK1L
Os06g22810.Ory.sativa.CrRLK1L
Os05g06990.Ory.sativa.CrRLK1L
Os01g06280.Ory.sativa.CrRLK1L
At2G39360.Arab.thal.CrRLK1L
659779.Pop.trichocarpa.CrRLK1L
CrRLK1.Cath.roseus
05G054860.1.Sol.lyc.CrRLK1L
II
At5G59700.Arab.thal.CrRLK1L
HERK1.Arab.thal.CrRLK1L
49039.Pop.trichocarpa.CrRLK1L
650315.Pop.trichocarpa.CrRLK1L
11G072910.1.Sol.lyc.CrRLK1L
I
LpimPth4.Sol.pimp.
05G013270.1.Sol.lyc.CrRLK1L
LpimPth3.Sol.pimp.
05G013320.1.Sol.lyc.CrRLK1L
Pto.Sol.pimp.
Fen.Sol.pimp.
05G013290.1.Sol.lyc.CrRLK1L
LpimPth2.Sol.pimp.
05G013300.1.Sol.lyc.CrRLK1L
02G014030.1.Sol.lyc.CrRLK1L
CTR1.Arab.thal.Raf
CPK7.Arab.thal.CDPK
0.2

C

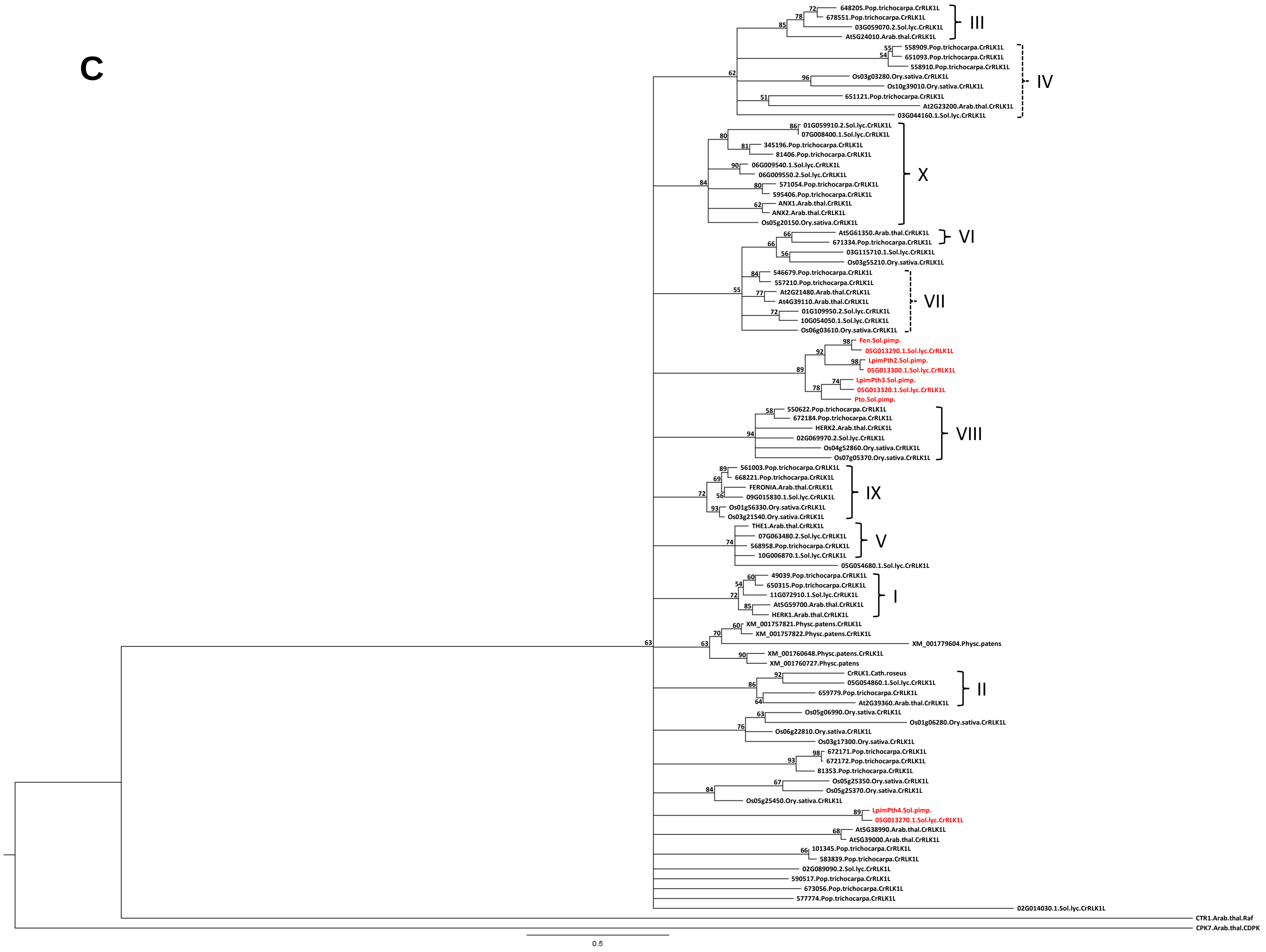
648205.Pop.trichocarpa.CrRLK1L
678551.Pop.trichocarpa.CrRLK1L
03G059070.2.Sol.lyc.CrRLK1L
At5G24010.Arab.thal.CrRLK1L
III
558909.Pop.trichocarpa.CrRLK1L
651093.Pop.trichocarpa.CrRLK1L
558910.Pop.trichocarpa.CrRLK1L
Os03g03280.Ory.sativa.CrRLK1L
Os10g39010.Ory.sativa.CrRLK1L
651121.Pop.trichocarpa.CrRLK1L
At2G23200.Arab.thal.CrRLK1L
03G044160.1.Sol.lyc.CrRLK1L
IV
01G059910.2.Sol.lyc.CrRLK1L
07G008400.1.Sol.lyc.CrRLK1L
345196.Pop.trichocarpa.CrRLK1L
81406.Pop.trichocarpa.CrRLK1L
06G009540.1.Sol.lyc.CrRLK1L
06G009550.2.Sol.lyc.CrRLK1L
571054.Pop.trichocarpa.CrRLK1L
595406.Pop.trichocarpa.CrRLK1L
ANX1.Arab.thal.CrRLK1L
ANX2.Arab.thal.CrRLK1L
Os05g20150.Ory.sativa.CrRLK1L
X
At5G61350.Arab.thal.CrRLK1L
671334.Pop.trichocarpa.CrRLK1L
VI
03G115710.1.Sol.lyc.CrRLK1L
Os03g55210.Ory.sativa.CrRLK1L
546679.Pop.trichocarpa.CrRLK1L
557210.Pop.trichocarpa.CrRLK1L
At2G21480.Arab.thal.CrRLK1L
At4G39110.Arab.thal.CrRLK1L
01G109950.2.Sol.lyc.CrRLK1L
10G054050.1.Sol.lyc.CrRLK1L
Os06g03610.Ory.sativa.CrRLK1L
VII
Fen.Sol.pimp.
05G013290.1.Sol.lyc.CrRLK1L
LpimPth2.Sol.pimp.
05G013300.1.Sol.lyc.CrRLK1L
LpimPth3.Sol.pimp.
05G013320.1.Sol.lyc.CrRLK1L
Pto.Sol.pimp.
550622.Pop.trichocarpa.CrRLK1L
672184.Pop.trichocarpa.CrRLK1L
HERK2.Arab.thal.CrRLK1L
02G069970.2.Sol.lyc.CrRLK1L
Os04g52860.Ory.sativa.CrRLK1L
Os07g05370.Ory.sativa.CrRLK1L
VIII
561003.Pop.trichocarpa.CrRLK1L
668221.Pop.trichocarpa.CrRLK1L
FERONIA.Arab.thal.CrRLK1L
09G015830.1.Sol.lyc.CrRLK1L
Os01g56330.Ory.sativa.CrRLK1L
Os03g21540.Ory.sativa.CrRLK1L
IX
THE1.Arab.thal.CrRLK1L
07G063480.2.Sol.lyc.CrRLK1L
568958.Pop.trichocarpa.CrRLK1L
10G006870.1.Sol.lyc.CrRLK1L
V
05G054680.1.Sol.lyc.CrRLK1L
49039.Pop.trichocarpa.CrRLK1L
650315.Pop.trichocarpa.CrRLK1L
11G072910.1.Sol.lyc.CrRLK1L
At5G59700.Arab.thal.CrRLK1L
HERK1.Arab.thal.CrRLK1L
I
XM_001757821.Physc.patens.CrRLK1L
XM_001757822.Physc.patens.CrRLK1L
XM_001779604.Physc.patens
XM_001760648.Physc.patens.CrRLK1L
XM_001760727.Physc.patens
CrRLK1.Cath.roseus
05G054860.1.Sol.lyc.CrRLK1L
659779.Pop.trichocarpa.CrRLK1L
At2G39360.Arab.thal.CrRLK1L
II
Os05g06990.Ory.sativa.CrRLK1L
Os01g06280.Ory.sativa.CrRLK1L
Os06g22810.Ory.sativa.CrRLK1L
Os03g17300.Ory.sativa.CrRLK1L
672171.Pop.trichocarpa.CrRLK1L
672172.Pop.trichocarpa.CrRLK1L
81353.Pop.trichocarpa.CrRLK1L
Os05g25350.Ory.sativa.CrRLK1L
Os05g25370.Ory.sativa.CrRLK1L
Os05g25450.Ory.sativa.CrRLK1L
LpimPth4.Sol.pimp.
05G013270.1.Sol.lyc.CrRLK1L
At5G38990.Arab.thal.CrRLK1L
At5G39000.Arab.thal.CrRLK1L
101345.Pop.trichocarpa.CrRLK1L
583839.Pop.trichocarpa.CrRLK1L
02G089090.2.Sol.lyc.CrRLK1L
590517.Pop.trichocarpa.CrRLK1L
673056.Pop.trichocarpa.CrRLK1L
577774.Pop.trichocarpa.CrRLK1L
02G014030.1.Sol.lyc.CrRLK1L
CTR1.Arab.thal.Raf
CPK7.Arab.thal.CDPK
0.5
