# Supplementary material for: Kinase domain-targeted isolation of defense-related receptor-like kinases (RLK/Pelle) in Platanus × acerifolia: phylogenetic and structural analysis
Source: BMC Res Notes. 2014 Dec 8;7:884. doi: 10.1186/1756-0500-7-884 (PMC4295470; doi:10.1186/1756-0500-7-884)
Supplement: Supplementary file 13 — Additional file 13: The molecular role of some invariant residues of the kinase domain. Substitutions in Platanus × acerifolia (Pac) sequences (atypical and non-RD motifs) are listed. (PDF 82 KB) [file 13104_2014_3456_MOESM13_ESM.pdf]

Additional file 13. The molecular role of some invariant residues of the kinase domain. Substitutions in *Platanus* × *acerifolia* (*Pac*) sequences (atypical and non-RD motifs) are listed.

Invariant residues substituted in some RLK/Pelles of *Pac*	Role for kinase catalytic activity [a]	*Pac* RLK/Pelles	and their substitutions [b]
Lys(K) – subdomain II<br>Glu(E) - subdomain III	Lys is stabilized and appropriately oriented by the ionic interaction with the invariant Glu of subdomain III. Lys thus makes crucial contact with ATP phosphates contributing to ATP positioning for phosphotransfer [54, 55, 84, 85]. In addition, the integrity of the Lys-Glu ion pair is crucial for correct conformational coupling of the N-terminal lobe (in which ATP binding occurs) with the activation segment of the C-terminal lobe (which provides a platform for phosphorilation and substrate binding) [AF13.1*]. In tomato Pto, experimental substitution of the invariant Lys abolished kinase activity [AF13.2*, AF13.3*, 58]	pac.Erf.4, LRR-XII-L<br>pac.W.ArA.11, WAK-like-L<br>pac.Erf.4, LRR-XII-L<br>pac.x.6.108, LRR-XII-L	K(II)→E<br>E(III)→G<br>E(III)→R<br>E(III)→K
Asp(D) – subdomain VIB	The RD-Asp is the catalytic base. It is involved in crucial interactions with the phospho-site of the substrate in order to promote the transfer of the γ-phosphate of ATP [54, 55, 85].<br>In tomato Pto, experimental substitution of the invariant Asp abolished kinase activity [AF13.3*, 58].	pac.x.5.39, LRR-VII-L<br>pac.x.5.43, LRR-VII-L	D(VIB) → N<br>D(VIB) → N
Arg(R) - subdomain VIB (Immediately preceding the invariant Asp)	The RD-Arg is crucial in that, as a positively charged residue, it inhibits catalysis by the negatively charged RD-Asp. Phosphorilation of Ser/Thr residues in the activation segment, generates phosphoamino acid(s) which contact the RD-Arg and neutralize its charge, thus enabling phosphotransfer by orientating the catalytic residue towards the phospho-site of the substrate [54, 85].	39 RLK/Pelles, for the most part LRR-XII-L	R(VIB) → C [c]

[a] The kinase catalytic domain of the eukaryotic protein kinase superfamily has been divided into 11 subdomains, defined as regions containing characteristic patterns of conserved residues of which twelve were considered nearly invariant throughout the whole superfamily. These subdomains confer the basic core two-lobed structure of the kinase domain and are essential for catalytic activity [AF13.4*, 84, 106]. Kinase activity is required at different steps for receptor kinase activation and the fulfilment of their function. A basic mechanism relies on autophosphorylation in the activation segment which i) stabilizes it for substrate binding, and ii) activates the catalytic residue for transphosphorylation [AF13.5*, AF13.6*, 55, 57, 62, 84, 85].
However, in the kinase superfamily of both animal and plant lineages, substitutions of the invariant residues are common and characterize kinase-defective domains which therefore signal using phosphorylation-independent mechanisms and for this reason are called atypical kinases [53]. *Arabidopsis* SUB (STRUBBELIG) [AF13.7*], tomato TARK1 (Tomato Atypical Receptor-like Kinases 1) [AF13.8*] and maize MARK (Maize Atypical Receptor-Kinase) [AF13.9*], are three representative plant atypical kinases which have been studied at a functional level.
Analyses of *Arabidopsis* kinome revealed that 20% of RLK/Pelles are putatively enzymatically inactive proteins [53]. The atypical motifs of *Pac* RLK/Pelles are the most frequent substitutions shared by the atypical kinases irrespective of the organism (animal or plant) and of the subfamily membership [53]. Specifically, substitutions of the conserved Glu were shared with tomato TARK1 [AF13.8*] and maize MARK [AF13.9*].

[b] Roman numerals in brackets indicate the kinase subdomain in which the residue is located.

[c] Cysteine (C) was the most frequent substitution of RD-Arg

* References not reported in the text

AF13.5 Adams JA: **Activation loop phosphorylation and catalysis in protein kinases: Is there functional evidence for the autoinhibitor model?** *Biochemistry* 2003, **42:**601–607.

AF13.7 Chevalier D, Batoux M, Fulton L, Pfister K, Yadav RK, Schellenberg M, Schneitz K: **STRUBBELIG defines a receptor kinase-mediated signaling pathway regulating organ development in Arabidopsis**. Proc Natl Acad Sci 2005, **102:**9074-9079.

AF13.4 Hanks SK, Quinn AM, Hunter T: **The protein kinase family: conserved features and deduced phylogeny of the catalytic domains**. *Science* 1988, **24:**42-52.

AF13.1 Jeffrey PD, Russo AA, Polyak K, Gibbs E, Hurwitz J, Massagué J, Pavletich NP: **Mechanism of CDK activation revealed by the structure of a cyclinA-CDK2 complex.** *Nature* 1995, **376:**313-320.

AF13.6 Johnson KL, Ingram GC: **Sending the right signals: regulating receptor kinase activity.** *Curr Opin Plant Biol* 2005, **8:**648-656.

AF13.8 Kim JG, Li X, Roden A, Taylor KW, Aakre CD, Su B, Lalonde S, Kirik A, Chen Y, Baranage G, McLane H, Martin GB, Mudgett MB: **Xanthomonas T3S effector XopN suppresses PAMP-triggered immunity and interacts with a tomato atypical receptor-like kinase and TFT1**. *Plant Cell* 2009, **21:**1305–1323.

AF13.9 Llompart B, Castells E, Rio A, Roca R, Ferrando A, Stiefel V, Puigdomenech P, Casacuberta JM. **The direct activation of MIK, a germinal center kinase (GCK)-like kinase, by MARK, a maize atypical receptor kinase, suggests a new mechanism for signaling through kinase-dead receptors.** *J Biol Chem* 2003, **278:**48105–48111.

AF13.3 Rathjen JP, Chang JH, Staskawicz BJ, Michelmore RW: **Constitutively active *Pto* induces a Prf-dependent hypersensitive response in the absence of *avrPto*.** *EMBO J* 1999, **18:**3232–3240.

AF13.2 Zhou J-M, Loh Y-T, Bressan RA, Martin GB: **The tomato gene *Pti1* encodes a serine-threonine kinase that is phosphorylated by Pto and is involved in the hypersensitive response.** *Cell* 1995, **83:**925–935.
