# Supplementary material for: Kinase domain-targeted isolation of defense-related receptor-like kinases (RLK/Pelle) in Platanus × acerifolia: phylogenetic and structural analysis
Source: BMC Res Notes. 2014 Dec 8;7:884. doi: 10.1186/1756-0500-7-884 (PMC4295470; doi:10.1186/1756-0500-7-884)

Additional file 14. Serine, threonine and tyrosine phosphorilation specificity motifs of RLK/Pelles of *Platanus* × *acerifolia* (*Pac*) and *Arabidopsis*. The motifs are compared with phosphorilation specificity motifs of protein kinase previously inferred [42]. Consensus was determined using MEME (a motif-based sequence analysis tool).

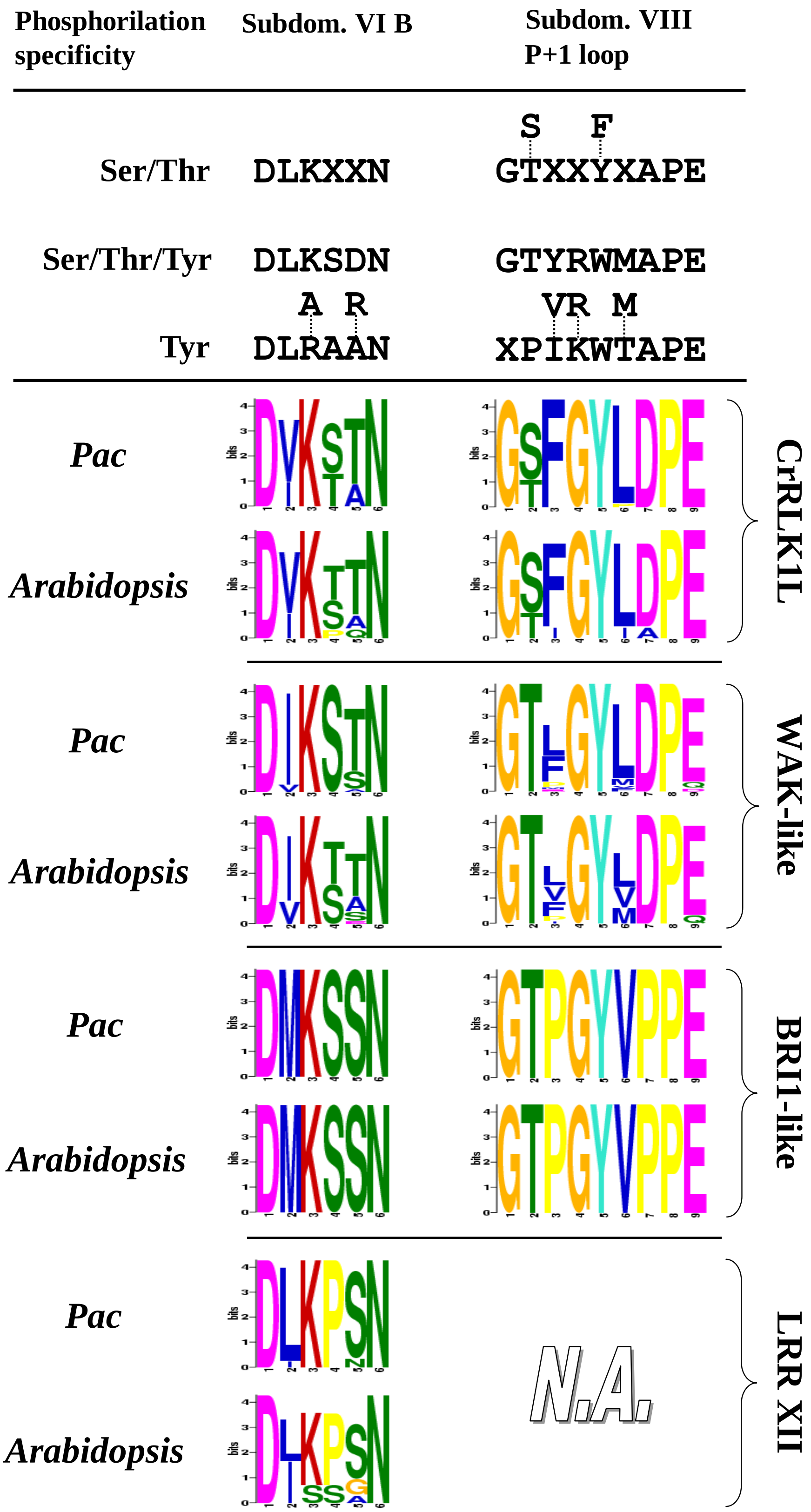

Supplement: Supplementary file 14 — Additional file 14: Serine, threonine and tyrosine phosphorilation specificity motifs of RLK/Pelles of Platanus × acerifolia ( Pac ) and Arabidopsis . The motifs are compared with phosphorilation specificity motifs of protein kinase previously inferred [42]. Consensus was determined using MEME (a motif-based sequence analysis tool). (PDF 128 KB) [file 13104_2014_3456_MOESM14_ESM.pdf]
