# Supplementary material for: Kinase domain-targeted isolation of defense-related receptor-like kinases (RLK/Pelle) in Platanus × acerifolia: phylogenetic and structural analysis
Source: BMC Res Notes. 2014 Dec 8;7:884. doi: 10.1186/1756-0500-7-884 (PMC4295470; doi:10.1186/1756-0500-7-884)
Supplement: Supplementary file 15 — Additional file 15: Crucial residues of the activation segments of Pto and BRI1. The relevant literature is cited in the text. (PDF 24 KB) [file 13104_2014_3456_MOESM15_ESM.pdf]

**Additional file 15. Crucial residues of the activation segments of Pto and BRI1.** The relevant literature is cited in the text.

| Pto residues [a] Functions | T190 | T195 | S198 | | T199 | V201 | K202 | G203 | T204 | L205 | G206 | Y207 | I208 | D209 | Y212 | F213 | I214 | K215 |
|---|---|---|---|---|---|---|---|---|---|---|---|---|---|---|---|---|---|---|
| Pto/avrPto interaction | | | | | + | | + | | + | + | | + | | + | | | | |
| Pto/avrPtoB interaction | | | | | + | | | | | + | | | | | | + | | + |
| Elicitation of hypersensitive response | | | + | | + | | | | | | | | | | | | | |
| Negative regulator of the resistance response | + | + | | | + | + | | + | + | + | + | + | + | | | | | |
| Autophosphorilation site | | | + | | + | | | | | | | | | | | | | |
| **BRI1 residues [a] Functions** | | T1039 | S1042 | S1044 | T1045 | | | | T1049 | | | Y1052 | | | Y1057 | | | |
| Auto/trans phosphorilation | | + | + | + | + | | | | + | | | | | | | | | |
| Required for BRI1 signalling | | + | + | + | + | | | | + | | | | | | | | | |
| Essential for kinase activity | | | | | | | | | | | | + | | | + | | | |

[a] Residues of Pto and BRI1 which have corresponding positions in the activation segment are reported in the same columns.
