# Supplementary material for: Kinase domain-targeted isolation of defense-related receptor-like kinases (RLK/Pelle) in Platanus × acerifolia: phylogenetic and structural analysis
Source: BMC Res Notes. 2014 Dec 8;7:884. doi: 10.1186/1756-0500-7-884 (PMC4295470; doi:10.1186/1756-0500-7-884)
Supplement: Supplementary file 16 — Additional file 16: Important residues of CrRLK1L located in the kinase domain region spanning subdomains VIB to VIII. The alignment shows the functional consensus derived from the Pto and BRI1 activation segment (subdomains VII and VIII) (Figure 6, Additional file 15) and its conservation in CrRLK1L(−L) subfamilies of Arabidopsis and Platanus × acerifolia (Pac). Black-shaded residues define the Pto functional consensus and the identities with CrRLK1L(−L). BRI1 consensus residues shared with Pto consensus motif, are shaded black, the residue exclusive of BRI1 consensus is shaded grey. Shaded-grey gaps have been inserted in order to match sequences with BRI1 consensus. The figure also shows the RD/non-RD motif and the regions that are useful to infer the ser/thr, tyr and the dual phosphorylation specificities of protein kinases [42] (Additional file 14) [red-shaded residues (subdomain VIb) and the P + 1 loop (subdomain VIII)]. (PDF 166 KB) [file 13104_2014_3456_MOESM16_ESM.pdf]

Additional file 16. Important residues of CrRLK1L located in the kinase domain region spanning subdomains VIB to VIII. The alignment shows the functional consensus derived from the Pto and BRI1 activation segment (subdomains VII and VIII) (Figure 6, Additional file 15) and its conservation in CrRLK1L(-L) subfamilies of *Arabidopsis* and *Platanus* × *acerifolia* (*Pac*). Black-shaded residues define the Pto functional consensus and the identities with CrRLK1L(-L). BRI1 consensus residues shared with Pto consensus motif, are shaded black, the residue exclusive of BRI1 consensus is shaded grey. Shaded-grey gaps have been inserted in order to match sequences with BRI1 consensus. The figure also shows the RD/non-RD motif and the regions that are useful to infer the ser/thr, tyr and the dual phosphorylation specificities of protein kinases [42] (Additional file 14) [red-shaded residues (subdomain VIb) and the P+1 loop (subdomain VIII)].

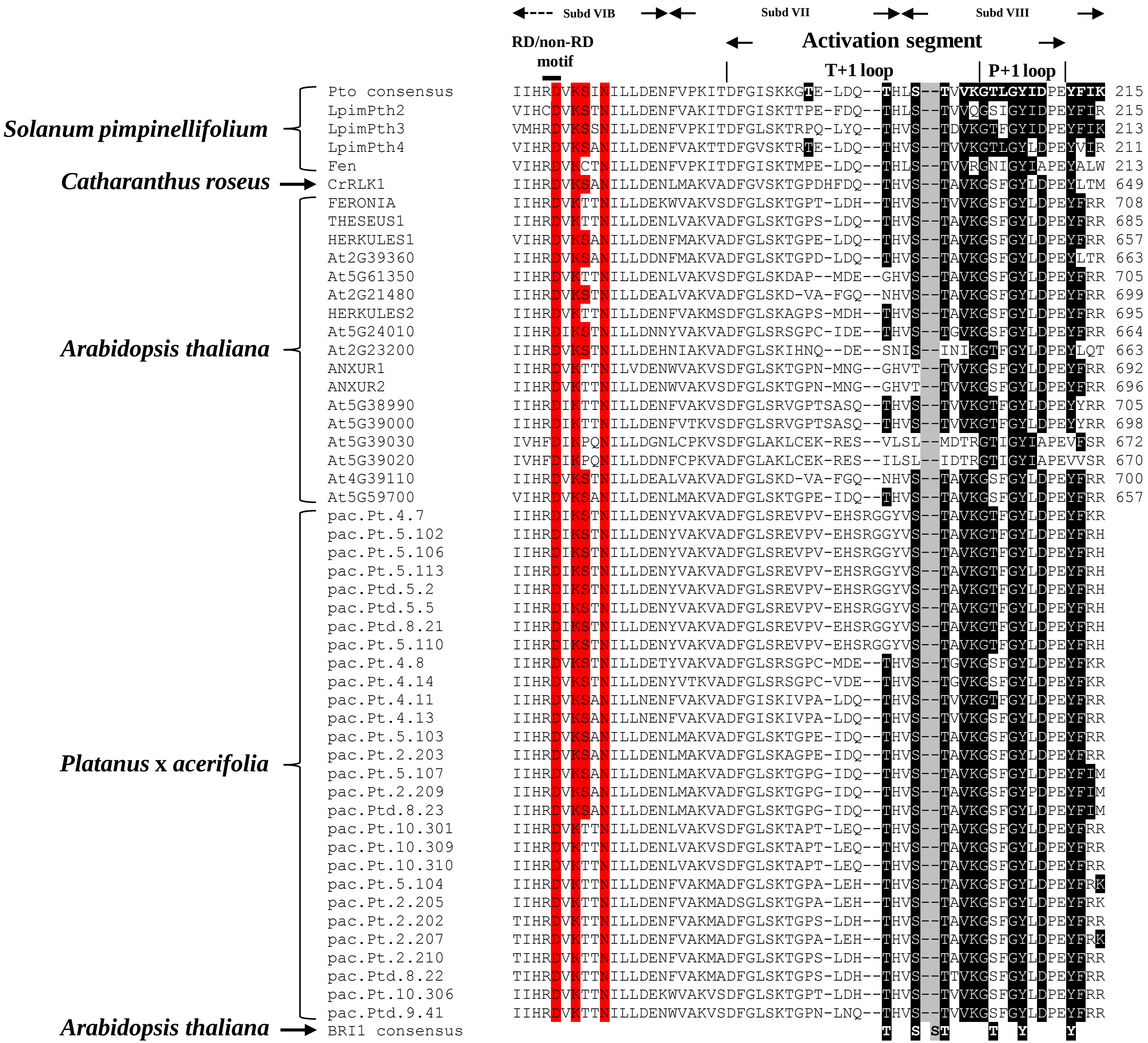

Subd VIB
Subd VII
Subd VIII
RD/non-RD motif
Activation segment
T+1 loop
P+1 loop
Solanum pimpinellifolium
Catharanthus roseus
Arabidopsis thaliana
Platanus x acerifolia
Arabidopsis thaliana
Pto consensus IIHRDVKSINILLDENFVPKITDFGISKKGTE-LDQ--THLS--TVVKGTLGYIDPEYFIK 215
LpimPth2 VIHCDVKSTNILLDENFVAKITDFGISKTTPE-FDQ--THLS--TVVQGSIGYIDPEYFIR 215
LpimPth3 VMHRDVKSSNILLDENFVPKITDFGLSKTRPQ-LYQ--THVS--TDVKGTFGYIDPEYFIK 213
LpimPth4 VIHRDVKSANILLDENFVAKTTDFGVSKTRTE-LDQ--THVS--TVVKGTLGYLDPEYVIR 211
Fen VIHRDVKCTNILLDENFVPKITDFGISKTMPE-LDQ--THLS--TVVRGNIGYIAPEYALW 213
CrRLK1 IIHRDVKSANILLDENLMAKVADFGVSKTGPDHFDQ--THVS--TAVKGSFGYLDPEYLTM 649
FERONIA IIHRDVKTTNILLDEKWVAKVSDFGLSKTGPT-LDH--THVS--TVVKGSFGYLDPEYFRR 708
THESEUS1 IIHRDVKTTNILLDENLVAKVADFGLSKTGPS-LDQ--THVS--TAVKGSFGYLDPEYFRR 685
HERKULES1 VIHRDVKSANILLDENFMAKVADFGLSKTGPE-LDQ--THVS--TAVKGSFGYLDPEYFRR 657
At2G39360 IIHRDVKSANILLDDNFMAKVADFGLSKTGPD-LDQ--THVS--TAVKGSFGYLDPEYLTR 663
At5G61350 IIHRDVKTTNILLDENLVAKVSDFGLSKDAP--MDE--GHVS--TAVKGSFGYLDPEYFRR 705
At2G21480 IIHRDVKSTNILLDEALVAKVADFGLSKD-VA-FGQ--NHVS--TAVKGSFGYLDPEYFRR 699
HERKULES2 IIHRDVKTTNILLDENFVAKMSDFGLSKAGPS-MDH--THVS--TAVKGSFGYLDPEYFRR 695
At5G24010 IIHRDIKSTNILLDNNYVAKVADFGLSRSGPC-IDE--THVS--TGVKGSFGYLDPEYFRR 664
At2G23200 IIHRDVKSTNILLDEHNIAKVADFGLSKIHNQ--DE--SNIS--INIKGTFGYLDPEYLQT 663
ANXUR1 IIHRDVKTTNILVDENWVAKVSDFGLSKTGPN-MNG--GHVT--TVVKGSFGYLDPEYFRR 692
ANXUR2 IIHRDVKTTNILLDENWVAKVSDFGLSKTGPN-MNG--GHVT--TVVKGSFGYLDPEYFRR 696
At5G38990 IIHRDIKTTNILLDENFVAKVSDFGLSRVGPTSASQ--THVS--TVVKGTFGYLDPEYYRR 705
At5G39000 IIHRDIKTTNILLDENFVTKVSDFGLSRVGPTSASQ--THVS--TVVKGTFGYLDPEYYRR 698
At5G39030 IVHFDIKPQNILLDGNLCPKVSDFGLAKLCEK-RES--VLSL--MDTRGTIGYIAPEVFSR 672
At5G39020 IVHFDIKPQNILLDDNFCPKVADFGLAKLCEK-RES--ILSL--IDTRGTIGYIAPEVVSR 670
At4G39110 IIHRDVKSTNILLDEALVAKVADFGLSKD-VA-FGQ--NHVS--TAVKGSFGYLDPEYFRR 700
At5G59700 VIHRDVKSANILLDENLMAKVADFGLSKTGPE-IDQ--THVS--TAVKGSFGYLDPEYFRR 657
pac.Pt.4.7 IIHRDIKSTNILLDENYVAKVADFGLSREVPV-EHSRGGYVS--TAVKGTFGYLDPEYFKR
pac.Pt.5.102 IIHRDIKSTNILLDENYVAKVADFGLSREVPV-EHSRGGYVS--TAVKGTFGYLDPEYFRH
pac.Pt.5.106 IIHRDIKSTNILLDENYVAKVADFGLSREVPV-EHSRGGYVS--TAVKGTFGYLDPEYFRH
pac.Pt.5.113 IIHRDIKSTNILLDENYVAKVADFGLSREVPV-EHSRGGYVS--TAVKGTFGYLDPEYFRH
pac.Ptd.5.2 IIHRDIKSTNILLDENYVAKVADFGLSREVPV-EHSRGGYVS--TAVKGTFGYLDPEYFRH
pac.Ptd.5.5 IIHRDIKSTNILLDENYVAKVADFGLSREVPV-EHSRGGYVS--TAVKGTFGYLDPEYFRH
pac.Ptd.8.21 IIHRDIKSTNILLDENYVAKVADFGLSREVPV-EHSRGGYVS--TAVKGTFGYLDPEYFRH
pac.Pt.5.110 IIHRDIKSTNILLDENYVAKVADFGLSREVPV-EHSRGGYVS--TAVKGTFGYLDPEYFRH
pac.Pt.4.8 IIHRDVKSTNILLDETYVAKVADFGLSRSGPC-MDE--THVS--TGVKGSFGYLDPEYFKR
pac.Pt.4.14 IIHRDVKSTNILLDENYVTKVADFGLSRSGPC-VDE--THVS--TGVKGSFGYLDPEYFKR
pac.Pt.4.11 IIHRDVKSANILLNENFVAKVADFGISKIVPA-LDQ--THVS--TVVKGTFGYLDPEYFRR
pac.Pt.4.13 IIHRDVKSANILLNENFVAKVADFGISKIVPA-LDQ--THVS--TVVKGSFGYLDPEYFRR
pac.Pt.5.103 IIHRDVKSANILLDENLMAKVADFGLSKTGPE-IDQ--THVS--TAVKGSFGYLDPEYFRR
pac.Pt.2.203 IIHRDVKSANILLDENLMAKVADFGLSKAGPE-IDQ--THVS--TAVKGSFGYLDPEYFRR
pac.Pt.5.107 IIHRDVKSANILLDENLMAKVADFGLSKTGPG-IDQ--THVS--TAVKGSFGYLDPEYFIM
pac.Pt.2.209 IIHRDVKSANILLDENLMAKVADFGLSKTGPG-IDQ--THVS--TAVKGSFGYPDPEYFIM
pac.Ptd.8.23 IIHRDVKSANILLDENLMAKVADFGLSKTGPG-IDQ--THVS--TAVKGSFGYLDPEYFIM
pac.Pt.10.301 IIHRDVKTTNILLDENLVAKVSDFGLSKTAPT-LEQ--THVS--TAVKGSFGYLDPEYFRR
pac.Pt.10.309 IIHRDVKTTNILLDENLVAKVSDFGLSKTAPT-LEQ--THVS--TAVKGSFGYLDPEYFRR
pac.Pt.10.310 IIHRDVKTTNILLDENLVAKVSDFGLSKTAPT-LEQ--THVS--TAVKGSFGYLDPEYFRR
pac.Pt.5.104 IIHRDVKTTNILLDENFVAKMADFGLSKTGPA-LEH--THVS--TAVKGSFGYLDPEYFRK
pac.Pt.2.205 IIHRDVKTTNILLDENFVAKMADSGLSKTGPA-LEH--THVS--TAVKGSFGYLDPEYFRK
pac.Pt.2.202 TIHRDVKTTNILLDENFVAKMADFGLSKTGPS-LDH--THVS--TAVKGSFGYLDPEYFRR
pac.Pt.2.207 TIHRDVKTTNILLDENFVAKMADFGLSKTGPA-LEH--THVS--TAVKGSFGYLDPEYFRK
pac.Pt.2.210 TIHRDVKTTNILLDENFVAKMADFGLSKTGPS-LDH--THVS--TAVKGSFGYLDPEYFRR
pac.Ptd.8.22 TIHRDVKTTNILLDENFVAKMADFGLSKTGPS-LDH--THVS--TTVKGSFGYLDPEYFRR
pac.Pt.10.306 IIHRDVKTTNILLDEKWVAKVSDFGLSKTGPT-LDH--THVS--TVVKGSFGYLDPEYFRR
pac.Ptd.9.41 IIHRDVKTTNILLDENWVAKVSDFGLSKTGPN-LNQ--THVS--TVVKGSFGYLDPEYFRR
BRI1 consensus T S ST T Y Y
