# Supplementary material for: Kinase domain-targeted isolation of defense-related receptor-like kinases (RLK/Pelle) in Platanus × acerifolia: phylogenetic and structural analysis
Source: BMC Res Notes. 2014 Dec 8;7:884. doi: 10.1186/1756-0500-7-884 (PMC4295470; doi:10.1186/1756-0500-7-884)
Supplement: Supplementary file 17 — Additional file 17: Important residues of Wak-like located in the kinase domain region spanning subdomains VIB to VIII. The alignment shows the functional consensus derived from the Pto and BRI1 activation segment (subdomains VII and VIII) (Figure 6, Additional file 15) and its conservation in the WAK-like(−L) subfamilies of Arabidopsis and Platanus × acerifolia (Pac). Black-shaded residues define the Pto functional consensus and the identities with WAK-like(−L). BRI1 consensus residues shared with Pto consensus motif, are shaded black, the residue exclusive of BRI1 consensus is shaded grey. Shaded-grey gaps have been inserted in order to match sequences with BRI1 consensus. The figure also shows the RD and non-RD motif and the regions that are useful to infer the ser/thr, tyr and the dual phosphorylation specificities of protein kinases [42] (Additional file 14) [red-shaded residues (subdomain VIb) and the P + 1 loop (subdomain VIII)]. (PDF 165 KB) [file 13104_2014_3456_MOESM17_ESM.pdf]

Additional file 17. Important residues of Wak-like located in the kinase domain region spanning subdomains VIB to VIII. The alignment shows the functional consensus derived from the Pto and BRI1 activation segment (subdomains VII and VIII) (Figure 6, Additional file 15) and its conservation in the WAK-like(-L) subfamilies of *Arabidopsis* and *Platanus* × *acerifolia* (*Pac*). Black-shaded residues define the Pto functional consensus and the identities with WAK-like(-L). BRI1 consensus residues shared with Pto consensus motif, are shaded black, the residue exclusive of BRI1 consensus is shaded grey. Shaded-grey gaps have been inserted in order to match sequences with BRI1 consensus. The figure also shows the RD and non-RD motif and the regions that are useful to infer the ser/thr, tyr and the dual phosphorylation specificities of protein kinases [42] (Additional file 14) [red-shaded residues (subdomain VIb) and the P+1 loop (subdomain VIII)].

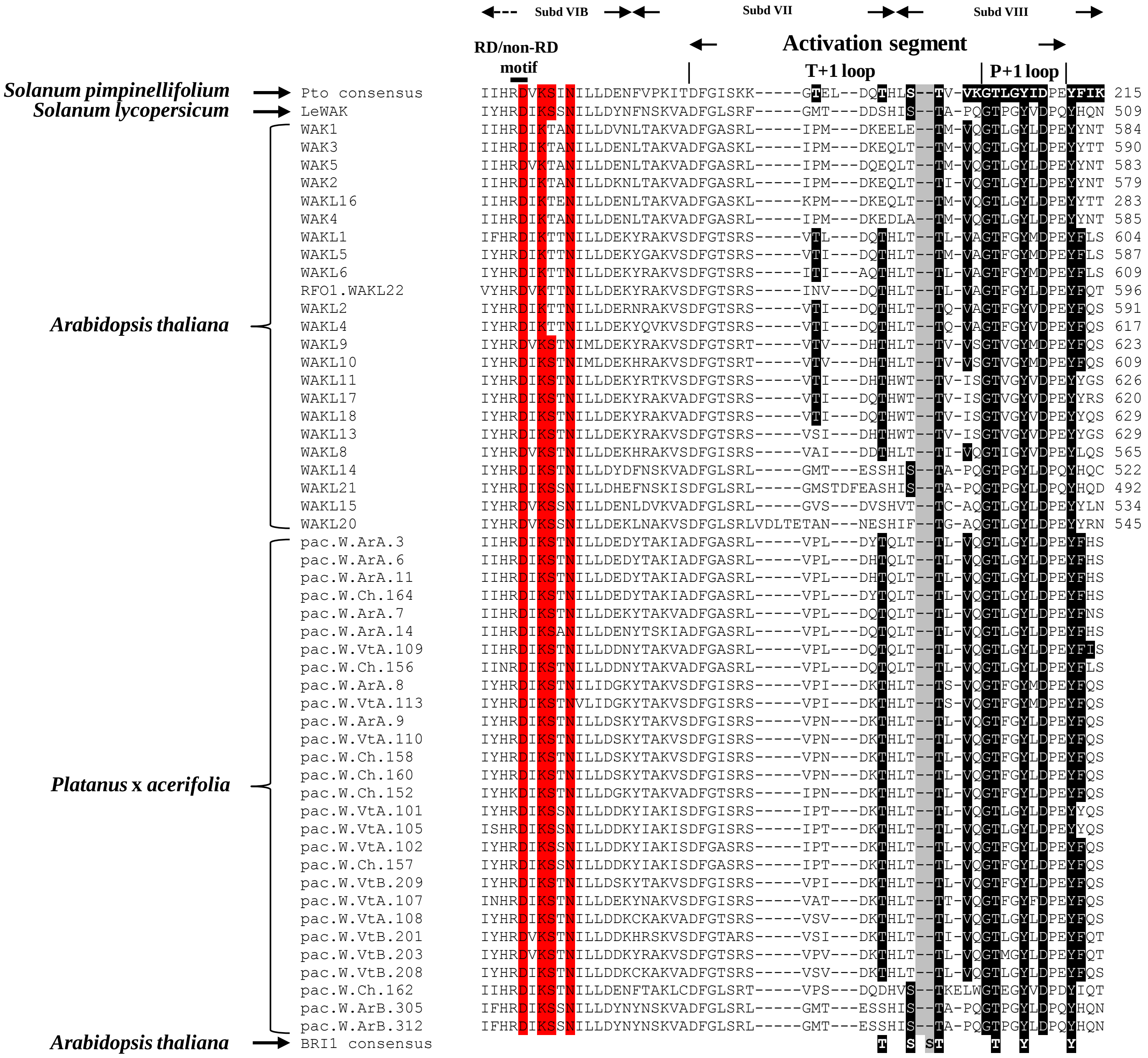

Subd VIB
Subd VII
Subd VIII
Activation segment
RD/non-RD motif
T+1 loop
P+1 loop
Solanum pimpinellifolium
Solanum lycopersicum
Arabidopsis thaliana
Platanus x acerifolia
Arabidopsis thaliana
Pto consensus IIHRDVKSINILLDENFVPKITDFGISKK-----GTEL--DQTHLS--TV-VKGTLGYIDPEYFIK 215
LeWAK IYHRDIKSSNILLDYNFNSKVADFGLSRF-----GMT---DDSHIS--TA-PQGTPGYVDPQYHQN 509
WAK1 IIHRDIKTANILLDVNLTAKVADFGASRL-----IPM---DKEELE--TM-VQGTLGYLDPEYYNT 584
WAK3 IIHRDIKTANILLDENLTAKVADFGASKL-----IPM---DKEQLT--TM-VQGTLGYLDPEYYTT 590
WAK5 IIHRDVKTANILLDENLTAKVADFGASRL-----IPM---DQEQLT--TM-VQGTLGYLDPEYYNT 583
WAK2 IIHRDIKTANILLDKNLTAKVADFGASRL-----IPM---DKEQLT--TI-VQGTLGYLDPEYYNT 579
WAKL16 IIHRDIKTENILLDENLTAKVADFGASKL-----KPM---DKEQLT--TM-VQGTLGYLDPEYYTT 283
WAK4 IIHRDIKTANILLDENLTAKVADFGASRL-----IPM---DKEDLA--TM-VQGTLGYLDPEYYNT 585
WAKL1 IFHRDIKTTNILLDEKYRAKVSDFGTSRS-----VTL---DQTHLT--TL-VAGTFGYMDPEYFLS 604
WAKL5 IYHRDIKTTNILLDEKYGAKVSDFGTSRS-----VTI---DQTHLT--TM-VAGTFGYMDPEYFLS 587
WAKL6 IYHRDIKTTNILLDEKYRAKVSDFGTSRS-----ITI---AQTHLT--TL-VAGTFGYMDPEYFLS 609
RFO1.WAKL22 VYHRDVKTTNILLDEKYRAKVSDFGTSRS-----INV---DQTHLT--TL-VAGTFGYLDPEYFQT 596
WAKL2 IYHRDIKTTNILLDERNRAKVSDFGTSRS-----VTI---DQTHLT--TQ-VAGTFGYVDPEYFQS 591
WAKL4 IYHRDIKTTNILLDEKYQVKVSDFGTSRS-----VTI---DQTHLT--TQ-VAGTFGYVDPEYFQS 617
WAKL9 IYHRDVKSTNIMLDEKYRAKVSDFGTSRT-----VTV---DHTHLT--TV-VSGTVGYMDPEYFQS 623
WAKL10 IYHRDIKSTNIMLDEKHRAKVSDFGTSRT-----VTV---DHTHLT--TV-VSGTVGYMDPEYFQS 609
WAKL11 IYHRDIKSTNILLDEKYRTKVSDFGTSRS-----VTI---DHTHWT--TV-ISGTVGYVDPEYYGS 626
WAKL17 IYHRDIKSTNILLDEKYRAKVADFGTSRS-----VTI---DQTHWT--TV-ISGTVGYVDPEYYRS 620
WAKL18 IYHRDIKSTNILLDEKYRAKVADFGTSRS-----VTI---DQTHWT--TV-ISGTVGYVDPEYYQS 629
WAKL13 IYHRDIKSTNILLDEKYRAKVSDFGTSRS-----VSI---DHTHWT--TV-ISGTVGYVDPEYYGS 629
WAKL8 IYHRDVKSTNILLDEKHRAKVSDFGISRS-----VAI---DDTHLT--TI-VQGTIGYVDPEYLQS 565
WAKL14 IYHRDIKSTNILLDYDFNSKVADFGLSRL-----GMT---ESSHIS--TA-PQGTPGYLDPQYHQC 522
WAKL21 IYHRDIKSSNILLDHEFNSKISDFGLSRL-----GMSTDFEASHIS--TA-PQGTPGYLDPQYHQD 492
WAKL15 IYHRDVKSSNILLDENLDVKVADFGLSRL-----GVS---DVSHVT--TC-AQGTLGYLDPEYYLN 534
WAKL20 IYHRDVKSSNILLDEKLNAKVSDFGLSRLVDLTETAN---NESHIF--TG-AQGTLGYLDPEYYRN 545
pac.W.ArA.3 IIHRDIKSTNILLDEDYTAKIADFGASRL-----VPL---DYTQLT--TL-VQGTLGYLDPEYFHS
pac.W.ArA.6 IIHRDIKSTNILLDEDYTAKIADFGASRL-----VPL---DHTQLT--TL-VQGTLGYLDPEYFHS
pac.W.ArA.11 IIHRDIKSTNILLDEDYTAKIADFGASRL-----VPL---DHTQLT--TL-VQGTLGYLDPEYFHS
pac.W.Ch.164 IIHRDIKSTNILLDEDYTAKIADFGASRL-----VPL---DYTQLT--TL-VQGTLGYLDPEYFHS
pac.W.ArA.7 IIHRDIKSTNILLDEKYTAKVADFGASRL-----VPL---DQTQLT--TL-VQGTLGYLDPEYFNS
pac.W.ArA.14 IIHRDIKSANILLDENYTSKIADFGASRL-----VPL---DQTQLT--TL-VQGTLGYLDPEYFHS
pac.W.VtA.109 IIHRDIKSTNILLDDNYTAKVADFGASRL-----VPL---DQTQLT--TL-VQGTLGYLDPEYFIS
pac.W.Ch.156 IINRDIKSTNILLDDNYTAKVADFGASRL-----VPL---DQTQLT--TL-VQGTLGYLDPEYFLS
pac.W.ArA.8 IYHRDIKSTNILIDGKYTAKVSDFGISRS-----VPI---DKTHLT--TS-VQGTFGYMDPEYFQS
pac.W.VtA.113 IYHRDIKSTNVLIDGKYTAKVSDFGISRS-----VPI---DKTHLT--TS-VQGTFGYMDPEYFQS
pac.W.ArA.9 IYHRDIKSTNILLDSKYTAKVSDFGISRS-----VPN---DKTHLT--TL-VQGTFGYLDPEYFQS
pac.W.VtA.110 IYHRDIKSTNILLDSKYTAKVSDFGISRS-----VPN---DKTHLT--TL-VQGTFGYLDPEYFQS
pac.W.Ch.158 IYHRDIKSTNILLDSKYTAKVSDFGISRS-----VPN---DKTHLT--TL-VQGTFGYLDPEYFQS
pac.W.Ch.160 IYHRDIKSTNILLDSKYTAKVSDFGISRS-----VPN---DKTHLT--TL-VQGTFGYLDPEYFQS
pac.W.Ch.152 IYHKDIKSTNILLDGKYTAKVADFGTSRS-----IPN---DKTHLT--TL-VQGTFGYLDPEYFQS
pac.W.VtA.101 IYHRDIKSSNILLDDKYIAKISDFGISRS-----IPT---DKTHLT--TL-VQGTLGYLDPEYYQS
pac.W.VtA.105 ISHRDIKSSNILLDDKYIAKISDFGISRS-----IPT---DKTHLT--TL-VQGTLGYLDPEYYQS
pac.W.VtA.102 IYHRDIKSSNILLDDKYIAKISDFGASRS-----IPT---DKTHLT--TL-VQGTFGYLDPEYFQS
pac.W.Ch.157 IYHRDIKSSNILLDDKYIAKISDFGASRS-----IPT---DKTHLT--TL-VQGTFGYLDPEYFQS
pac.W.VtB.209 IYHRDIKSTNILLDSKYTAKVSDFGISRS-----VPI---DKTHLT--TL-VQGTFGYLDPEYFQS
pac.W.VtA.107 INHRDIKSTNILLDEKYNAKVSDFGISRS-----VAT---DKTHLT--TT-VQGTFGYFDPEYFQS
pac.W.VtA.108 IYHRDIKSTNILLDDKCKAKVADFGTSRS-----VSV---DKTHLT--TL-VQGTLGYLDPEYFQS
pac.W.VtB.201 IYHRDVKSTNILLDDKHRSKVSDFGTARS-----VSI---DKTHLT--TI-VQGTLGYLDPEYFQT
pac.W.VtB.203 IYHRDVKSTNILLDDKYRAKVSDFGTSRS-----VPV---DKTHLT--TL-VQGTMGYLDPEYFQT
pac.W.VtB.208 IYHRDIKSTNILLDDKCKAKVADFGTSRS-----VSV---DKTHLT--TL-VQGTLGYLDPEYFQS
pac.W.Ch.162 IIHRDIKSTNILLDENFTAKLCDFGLSRT-----VPS---DQDHVS--TKELWGTEGYVDPDYIQT
pac.W.ArB.305 IFHRDIKSSNILLDYNYNSKVADFGLSRL-----GMT---ESSHIS--TA-PQGTPGYLDPQYHQN
pac.W.ArB.312 IFHRDIKSSNILLDYNYNSKVADFGLSRL-----GMT---ESSHIS--TA-PQGTPGYLDPQYHQN
BRI1 consensus T S ST T Y Y
