# Supplementary material for: Kinase domain-targeted isolation of defense-related receptor-like kinases (RLK/Pelle) in Platanus × acerifolia: phylogenetic and structural analysis
Source: BMC Res Notes. 2014 Dec 8;7:884. doi: 10.1186/1756-0500-7-884 (PMC4295470; doi:10.1186/1756-0500-7-884)
Supplement: Supplementary file 18 — Additional file 18: Important residues of LRR X-BRI1 group located in the kinase domain region spanning subdomains VIB to VIII. The alignment shows the functional consensus derived from the Pto and BRI1 activation segment (subdomains VII and VIII) (Figure 6, Additional file 15) and its conservation in the LRR X-BRI1(−L) group of Arabidopsis and Platanus × acerifolia (Pac). Black-shaded residues define the Pto functional consensus and the identities with LRR X-BRI1(−L). BRI1 consensus residues shared with Pto consensus motif, are shaded black, the residue exclusive of BRI1 consensus is shaded grey. The figure also shows the RD motif and the regions that are useful to infer the ser/thr, tyr and the dual phosphorylation specificities of protein kinases [42] (Additional file 14) [red-shaded residues (subdomain VIb) and the P + 1 loop (subdomain VIII)]. (PDF 169 KB) [file 13104_2014_3456_MOESM18_ESM.pdf]

Additional file 18. Important residues of LRR X-BRI1 group located in the kinase domain region spanning subdomains VIB to VIII. The alignment shows the functional consensus derived from the Pto and BRI1 activation segment (subdomains VII and VIII) (Figure 6, Additional file 15) and its conservation in the LRR X-BRI1(-L) group of *Arabidopsis* and *Platanus* × *acerifolia* (*Pac*). Black-shaded residues define the Pto functional consensus and the identities with LRR X-BRI1(-L). BRI1 consensus residues shared with Pto consensus motif, are shaded black, the residue exclusive of BRI1 consensus is shaded grey. The figure also shows the RD motif and the regions that are useful to infer the ser/thr, tyr and the dual phosphorylation specificities of protein kinases [42] (Additional file 14) [red-shaded residues (subdomain VIb) and the P+1 loop (subdomain VIII)].

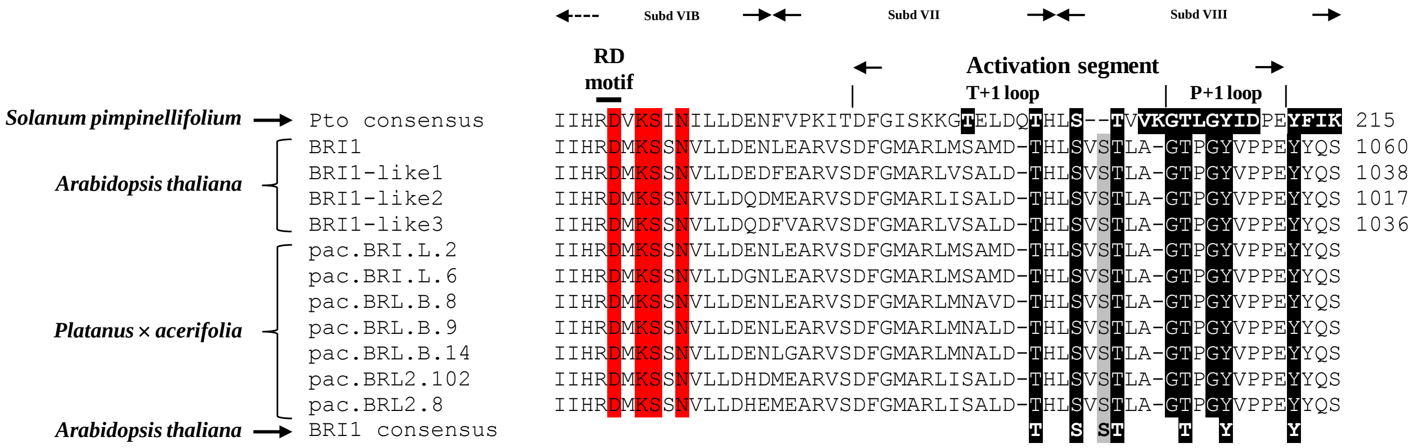

Subd VIB
Subd VII
Subd VIII
RD motif
Activation segment
T+1 loop
P+1 loop
Solanum pimpinellifolium
Pto consensus IIHRDVKSINILLDENFVPKITDFGISKKGTELDQTHLS--TVVKGTLGYIDPEYFIK 215
Arabidopsis thaliana
BRI1 IIHRDMKSSNVLLDENLEARVSDFGMARLMSAMD-THLSVSTLA-GTPGYVPPEYYQS 1060
BRI1-like1 IIHRDMKSSNVLLDEDFEARVSDFGMARLVSALD-THLSVSTLA-GTPGYVPPEYYQS 1038
BRI1-like2 IIHRDMKSSNVLLDQDMEARVSDFGMARLISALD-THLSVSTLA-GTPGYVPPEYYQS 1017
BRI1-like3 IIHRDMKSSNVLLDQDFVARVSDFGMARLVSALD-THLSVSTLA-GTPGYVPPEYYQS 1036
Platanus × acerifolia
pac.BRI.L.2 IIHRDMKSSNVLLDENLEARVSDFGMARLMSAMD-THLSVSTLA-GTPGYVPPEYYQS
pac.BRI.L.6 IIHRDMKSSNVLLDGNLEARVSDFGMARLMSAMD-THLSVSTLA-GTPGYVPPEYYQS
pac.BRL.B.8 IIHRDMKSSNVLLDENLEARVSDFGMARLMNAVD-THLSVSTLA-GTPGYVPPEYYQS
pac.BRL.B.9 IIHRDMKSSNVLLDENLEARVSDFGMARLMNALD-THLSVSTLA-GTPGYVPPEYYQS
pac.BRL.B.14 IIHRDMKSSNVLLDENLGARVSDFGMARLMNALD-THLSVSTLA-GTPGYVPPEYYQS
pac.BRL2.102 IIHRDMKSSNVLLDHDMEARVSDFGMARLISALD-THLSVSTLA-GTPGYVPPEYYQS
pac.BRL2.8 IIHRDMKSSNVLLDHEMEARVSDFGMARLISALD-THLSVSTLA-GTPGYVPPEYYQS
Arabidopsis thaliana
BRI1 consensus T S ST T Y Y
