# Supplementary material for: Kinase domain-targeted isolation of defense-related receptor-like kinases (RLK/Pelle) in Platanus × acerifolia: phylogenetic and structural analysis
Source: BMC Res Notes. 2014 Dec 8;7:884. doi: 10.1186/1756-0500-7-884 (PMC4295470; doi:10.1186/1756-0500-7-884)
Supplement: Supplementary file 19 — Additional file 19: Ka/Ks values obtained for each window in the sliding window analysis of Platanus × acerifolia ( Pac ) LRR XII-L. CrRLK1L-L of Pac and LRR XII of Arabidopsis are included for comparison. (PDF 61 KB) [file 13104_2014_3456_MOESM19_ESM.pdf]

Additional file 19. Ka/Ks values obtained for each window in the sliding window analysis of *Platanus* × *acerifolia* (*Pac*) LRR XII-L. CrRLK1L-L of Pac and LRR XII of *Arabidopsis* are included for comparison.

Sequence Groups [a]	*Pac* I LRR XII-L			*Pac* II LRR XII-L			*Pac* IV+V LRR XII-L			*Arabidopsis* [b] LRR XII			*Pac* CrRLK1L-L		
window [c]	Mean	count > 1	max. value	Mean	count > 1	max. value	Mean	count > 1	max. value	Mean	count > 1	max. value	Mean	count > 1	max. value
1	0.50	8	1.36	0.96	15	1.9	0.56	4	1.08	0.17	0	0.57	0.04	0	0.49
2	0.39	6	1.92	0.66	5	1.26	0.22	0	0.51	0.12	0	0.35	0.03	0	0.42
3	0.18	0	0.42	0.41	6	1.19	0.13	0	0.26	0.11	0	0.21	0.04	0	0.42
4	0.13	0	0.31	0.30	0	0.91	0.10	0	0.30	0.09	0	0.20	0.03	0	0.40
5	0.12	0	0.3	0.22	0	0.68	0.05	0	0.13	0.04	0	0.11	0.05	0	0.34
6	0.18	0	0.63	0.10	0	0.25	0.09	0	0.10	0.08	0	0.18	0.06	0	0.32
7	0.25	0	0.68	0.17	0	0.3	0.13	0	0.21	0.06	0	0.19	0.05	0	0.33
8	0.24	0	0.9	0.16	0	0.3	0.14	0	0.30	0.06	0	0.20	0.04	0	0.32
9	0.28	0	0.94	0.15	0	0.29	0.17	0	0.39	0.13	0	0.38	0.06	0	0.64
10	0.39	3	3	0.16	0	0.75	0.13	0	0.27	0.15	0	0.32	0.05	1	1.16
11	0.49	3	6.61	0.24	1	1.1	0.15	0	0.44	0.13	0	0.29	0.06	0	0.99
12	0.57	1	3.07	0.35	0	0.71	0.21	0	0.38	0.18	0	0.36	0.07	1	1.03
13	0.84	25	3.03	0.96	12	3.83	0.23	0	0.97	0.34	0	0.85	0.07	1	1.12
14	0.91	33	3.18	0.78	5	2.52	0.23	0	0.98	0.22	0	0.43	0.07	1	1.46
15	0.79	21	2.44	0.50	1	1.64	0.27	0	0.91	0.23	0	0.50	0.05	1	1.04
16	0.71	23	2.51	0.40	0	0.64	0.15	0	0.37	0.14	0	0.47	0.03	0	0.20
17	0.45	6	4.72	0.35	0	0.71	0.11	0	0.26	0.20	0	0.45	0.03	0	0.32
18	0.30	0	0.98	0.32	0	0.81	0.15	0	0.34	0.13	0	0.22	0.03	0	0.92
19	0.25	3	1.33	0.19	0	0.94	0.11	0	0.22	0.18	0	0.37	0.03	0	0.42
20	0.48	11	4.62	0.25	0	0.64	0.13	0	0.47	0.11	0	0.26	0.04	0	0.40
21	0.27	4	3.03	0.30	0	0.45	0.29	0	0.92	0.16	0	0.53	0.04	0	0.46
22	0.24	2	1.21	0.28	0	0.36	0.82	3	2.42	0.17	0	0.29	0.03	0	0.19
23	0.18	0	1	0.36	1	1.04	0.59	0	1.00	0.09	0	0.22	0.06	0	0.20
24	0.18	1	1.12	0.35	0	0.99	0.34	1	1.23	0.06	0	0.11	0.16	0	0.36
25	0.26	2	1.03	0.28	0	0.81	0.23	0	0.47	0.06	0	0.19	0.12	0	0.33
26	0.47	8	2.25	0.57	8	2.12	0.18	0	0.43	0.10	0	0.42	0.09	0	0.31
27	0.39	4	1.76	0.49	4	2.07	0.21	0	0.59	0.06	0	0.48	N.A.	N.A.	N.A.
28	0.63	14	2.65	0.61	8	1.95	0.24	0	0.42	0.04	0	0.45	N.A.	N.A.	N.A.
29	0.45	2	2.46	0.77	10	3.9	N.A. [d]	N.A.	N.A.	0.05	0	0.32	N.A.	N.A.	N.A.

[a] I, II and IV+V refer to sequences of phylogenetic clades of LRR XII-L depicted in Figure 2. [b] Results of the comparison between AT4G08850 and AT1G35710 of *Arabidopsis* are not shown as they were analysed separately (see the text). For this couple, values for each window were similar to those obtained in the other pairwise comparisons (i.e. in the range: 0.01-0.33). [c] The numbers of the CrRLK1L-L windows do not precisely correspond to those of the other gene groups because of the presence of multiple gaps in the alignment between LRR XII and CrRLK1L.
[d] N.A. = Not available.
