# Supplementary material for: Kinase domain-targeted isolation of defense-related receptor-like kinases (RLK/Pelle) in Platanus × acerifolia: phylogenetic and structural analysis
Source: BMC Res Notes. 2014 Dec 8;7:884. doi: 10.1186/1756-0500-7-884 (PMC4295470; doi:10.1186/1756-0500-7-884)
Supplement: Supplementary file 20 — Additional file 20: Amino acid variability at the kinase domain positions subject to non-synonymous variation. Analysis focuses on phylogenetic clades of LRR-XII-L of Platanus × acerifolia (Pac) and LRR-XII of Arabidopsis (refer Figure 2). The three distinct tables present the data regarding the sharing of the variable positions among four, three and two distinct group of sequences. (PDF 183 KB) [file 13104_2014_3456_MOESM20_ESM.pdf]

Additional file 20. Amino acid variability at the kinase domain positions subject to non-synonymous variation. Analysis focuses on phylogenetic clades of LRR-XII-L of *Platanus* × *acerifolia* (*Pac*) and LRR-XII of *Arabidopsis* (refer to Figure 2). The three distinct tables present the data regarding the sharing of the variable positions among four, three and two distinct group of sequences.

Positions [a]	Amino acid variation [b]									
**1**	I clade	**I**	N							
	II clade	**I**		M						
	IV/V clade	**I**			**L**	T				
	Arabidopsis				**L**		F			
**4**	I clade	**Q**	R							
	II clade	**Q**		Y						
	IV/V clade	**Q**			N	H				
	Arabidopsis						P	L	T	S
**10**	I clade	**P**	F							
	II clade	**P**		**L**	S	T				
	IV/V clade						N	**I**		
	Arabidopsis			**L**				**I**	A	
**19**	I clade	**L**	**Q**							
	II clade	**L**	**Q**							
	IV/V clade		**Q**	P						
	Arabidopsis	**L**	**Q**		C					
**21**	I clade	**H**	W	C	**Y**	**Q**	**R**			
	II clade					**Q**	**R**			
	IV/V clade	**H**			**Y**	**Q**				
	Arabidopsis	**H**					**R**			
**46**	I clade	**L**	F							
	II clade	**L**		**I**						
	IV/V clade	**L**		**I**						
	Arabidopsis	**L**		**I**	Q	V				
**52**	I clade	**T**	**I**	**V**						
	II clade		**I**	**V**	A					
	IV/V clade	**T**	**I**			M				
	Arabidopsis	**T**	**I**				L	S		
**55**	I clade	**E**	**Q**							
	II clade			**K**	M					
	IV/V clade	**E**		**K**		A				
	Arabidopsis	**E**	**Q**							
**79**	I clade	**S**	**N**	T	K					
	II clade	**S**	**N**			R				
	IV/V clade		**N**				G	**E**		
	Arabidopsis							**E**	D	

**83**	I clade	Y	N	D					
	II clade		N	D	E				
	IV/V clade			D		V	H		
	Arabidopsis				E			R	S
**84**	I clade	G	E						
	II clade	G	E						
	IV/V clade			A	T				
	Arabidopsis			A	T	V	I		
**86**	I clade	H	N						
	II clade	H		L					
	IV/V clade				V	D			
	Arabidopsis		N			D	R		
**93**	I clade	F	L						
	II clade	F		I					
	IV/V clade	F	L	I					
	Arabidopsis	F	L		P				
**113**	I clade	Q	H						
	II clade	Q	H	N					
	IV/V clade		H	N					
	Arabidopsis		H		Y				
**148**	I clade	S	F						
	II clade	S	F	Y					
	IV/V clade				P	L			
	Arabidopsis			Y		L			
**149**	I clade	K	N	M					
	II clade			M	R				
	IV/V clade			M		E			
	Arabidopsis	K	N						
**153**	I clade	Y	N	F					
	II clade		N		D				
	IV/V clade		N			A	R		
	Arabidopsis				D			E	
**157**	I clade	N	F	D					
	II clade				S	G			
	IV/V clade	N			S				
	Arabidopsis	N					I		
**159**	I clade	T	S						
	II clade			Y	H				
	IV/V clade	T	S						
	Arabidopsis					F	L		

[a] Numbering of the positions refers to Fig. 8.
[b] Aligning and black shading highlight amino acid identity among the different sequence groups.

Positions [a]	Amino acid variation [b]									
**3**	I clade	H	**P**	R	C					
	IV/V clade					N	**G**	D		
	Arabidopsis		**P**				**G**		Q	L
**13**	I clade	**V**	**I**							
	II clade	**V**		L						
	Arabidopsis	**V**	**I**							
**24**	II clade	**S**	F							
	IV/V clade	**S**		Y						
	Arabidopsis				T	M	A			
**33**	I clade	**A**	I							
	IV/V clade	**A**		**S**						
	Arabidopsis	**A**		**S**	T					
**36**	I clade	E	A							
	II clade			N	K					
	Arabidopsis					G	D			
**43**	I clade	**L**	**V**							
	II clade	**L**	**V**							
	Arabidopsis		**V**	A						
**48**	I clade	**S**	**A**	C						
	IV/V clade	**S**			T	**I**				
	Arabidopsis		**A**			**I**	V			
**58**	I clade	**D**	F							
	II clade	**D**		**E**						
	Arabidopsis	**D**		**E**						
**64**	I clade	**F**	S	**Y**						
	II clade	**F**			L					
	IV/V clade	**F**		**Y**						
**66**	I clade	L	**F**							
	II clade		**F**	**Y**						
	Arabidopsis		**F**	**Y**						
**68**	I clade	**P**	H							
	II clade	**P**		**S**	T					
	IV/V clade	**P**		**S**						
**71**	II clade	**S**	**N**							
	IV/V clade	**S**	**N**							
	Arabidopsis	**S**	**N**							
**74**	I clade	N	**R**							
	IV/V clade		**R**	**K**						
	Arabidopsis			**K**	M					
**82**	I clade	**T**	L	**I**	P					
	II clade	**T**		**I**		V				
	IV/V clade						E	D	Y	

**85**	I clade	**R**	**G**	**Q**				
	II clade			**Q**	**H**	K		
	Arabidopsis	**R**	**G**		**H**		N	
**88**	II clade	**L**	**S**					
	IV/V clade	**L**		Q				
	Arabidopsis		**S**		P			
**90**	I clade	**Y**	N	K	D			
	II clade	**Y**				C		
	Arabidopsis						S	T
**92**	I clade	**N**	**S**	R	C			
	II clade	**N**	**S**					
	Arabidopsis					T	G	
**99**	I clade	**I**	**M**					
	II clade	**I**	**M**	V				
	IV/V clade	**I**			T			
**105**	I clade	**S**	**F**	**L**				
	II clade	**S**	**F**	**L**				
	IV/V clade	**S**			C			
**108**	II clade	**D**	N					
	IV/V clade	**D**		**E**				
	Arabidopsis	**D**		**E**	V			
**116**	II clade	**T**	**I**					
	IV/V clade	**T**	**I**					
	Arabidopsis			D	E	N		
**123**	I clade	**L**	**I**					
	II clade	**L**	**I**					
	Arabidopsis	**L**	**I**					
**128**	I clade	**V**	**I**					
	IV/V clade	**V**	**I**					
	Arabidopsis	**V**	**I**					
**135**	I clade	**V**	I	**T**	A			
	II clade			**T**		S		
	IV/V clade	**V**		**T**				
**146**	I clade	**F**	**I**					
	II clade	**F**	**I**	T				
	IV/V clade	**F**			L			
**150**	I clade	P	L	**T**	D			
	IV/V clade			**T**		A		
	Arabidopsis						Y	F
**151**	I clade	**T**	**S**	D				
	II clade	**T**	**S**		P			
	IV/V clade	**T**	**S**			I		

**152**	I clade	K	N	S					
	II clade		N	S	D				
	Arabidopsis	K				R	E	Q	
**154**	I clade	S	F	T					
	II clade	S			C				
	Arabidopsis	S		T					
**156**	I clade	N	K	E					
	IV/V clade				R	Q			
	Arabidopsis						L	F	H

[a] Numbering of the positions refers to Fig. 8.
[b] Aligning and black shading highlight amino acid identity among the different sequence groups.

Positions [a]	Amino acid variation [b]					
**5**	I clade	N	D			
	Arabidopsis			E	K	
**6**	I clade	E	D	K		
	Arabidopsis	E		K	S	N
**11**	I clade	I	V			
	II clade	I	V	F		
**16**	I clade	F	L	V		
	IV/V clade	F	L			
**18**	II clade	L	V			
	Arabidopsis	L		M		
**23**	II clade	A	G			
	IV/V clade	A		S		
**29**	I clade	T	A			
	II clade	T	A	V		
**37**	II clade	I	V			
	Arabidopsis	I		T		
**38**	I clade	R	Q			
	IV/V clade	R	Q			
**54**	I clade	F	I			
	Arabidopsis	F		S		
**63**	II clade	V	I			
	Arabidopsis	V	I			
**67**	I clade	M	L	V		
	Arabidopsis	M	L			

**69**	II clade	N	S					
	Arabidopsis	N		K				
**77**	IV/V clade	Y	H					
	Arabidopsis		H	Q				
**78**	I clade	Q	P					
	Arabidopsis		P	L				
**89**	I clade	N	K					
	IV/V clade			R	M	G		
**94**	I clade	D	G	R	S			
	Arabidopsis					A	L	F
**95**	I clade	Q	K					
	Arabidopsis			E	A			
**98**	I clade	N	D					
	II clade	N	D					
**100**	I clade	A	S					
	Arabidopsis	A		V				
**101**	I clade	I	L	M				
	II clade	I		M				
**103**	I clade	V	I					
	II clade	V		A				
**106**	I clade	A	T					
	Arabidopsis	A		V				
**111**	I clade	H	Q					
	II clade	H	Q	K				
**112**	I clade	H	Y					
	Arabidopsis			V	T			
**114**	I clade	C	N					
	IV/V clade	C		S				
**115**	II clade	P	S	Q				
	IV/V clade	P		Q	K			
**119**	II clade	V	A					
	IV/V clade	V		I				
**131**	II clade	D	N					
	Arabidopsis	D		E				
**132**	IV/V clade	D	N					
	Arabidopsis	D		K				
**133**	I clade	D	G	N	S			
	II clade	D				E		

**147**	I clade	L	I			
	IV/V clade	L		H		
**155**	I clade	G	R	S	D	
	II clade	G				A
**158**	I clade	K	E	D		
	IV/V clade				Q	H
**162**	II clade	I	A	V		
	IV/V clade				I	M
**163**	I clade	V	A			
	II clade		A	T		
**164**	I clade	I	L			
	II clade	I	L			

[a] Numbering of the positions refers to Fig. 8.
[b] Aligning and black shading highlight amino acid identity among the different sequence groups.
